# Post-discharge suicide prediction among US veterans using natural language processing-enriched social and behavioral determinants of health

**DOI:** 10.1038/s44184-025-00120-2

**Published:** 2025-02-22

**Authors:** Avijit Mitra, Kun Chen, Weisong Liu, Ronald C. Kessler, Hong Yu

**Affiliations:** 1 Manning College of Information and Computer Sciences, University of Massachusetts Amherst, Amherst, MA USA; 2 Department of Statistics, University of Connecticut, Storrs, CT USA; 3 Center for Population Health, University of Connecticut Health Center, Farmington, CT USA; 4 Miner School of Computer and Information Sciences, University of Massachusetts Lowell, Lowell, MA USA; 5 Center for Biomedical and Health Research in Data Sciences, University of Massachusetts Lowell, Lowell, MA USA; 6 Department of Health Care Policy, Harvard Medical School, Boston, MA USA; 7 Center for Healthcare Organization & Implementation Research, Veterans Affairs Bedford Healthcare System, Bedford, MA USA

**Keywords:** Machine learning, Predictive markers

## Abstract

Despite the established association between social and behavioral determinants of health (SBDH) and suicide risk, SBDHs from unstructured electronic health record notes for suicide prediction remain underutilized. This study investigates the impact of SBDH identified from both structured and unstructured data utilizing a natural language processing (NLP) system on suicide prediction at 7, 30, 90, and 180 days post-discharge. Using data from 2,987,006 US Veterans between 1 October 2009, and 30 September 2015, we designed a case-control study demonstrating that structured and NLP-extracted SBDH significantly enhance distinct prediction models’ performance. For example, the random forest model improved its 180-day post-discharge prediction with an area under the receiver operating characteristic curve increase from 83.57% to 84.25% (95% CI = 0.63%–0.98%, *p* val < 0.001) and area under the precision-recall curve increase from 57.38% to 59.87% (95% CI = 3.86%–4.82%, *p* val < 0.001) after integrating NLP-extracted SBDH. These findings underscore the potential of NLP-extracted SBDH in advancing suicide prediction.

## Introduction

Suicide has consistently ranked among the primary causes of mortality in the US for decades, with a substantial 35.6% increase from 2000 to 2021^1^. In 2021 alone, suicide accounted for 48,183 fatalities in the US^1^, while the global toll surpassed 700,000^2,3^. Existing data indicates a higher suicide rate among Veterans than non-veteran adults over the last decade and notably, Veterans are experiencing a more pronounced increase of suicide risk^4^. Prior studies found that 80% of suicide victims were in contact with their primary care providers in the year preceding their death, and within the same timeframe, 25.7–31% had sought mental health care^5,6^. This puts healthcare providers in a unique position to contribute, and a better predictive tool may assist them in mitigating the prospective risk of suicidal events.

Social and behavioral determinants of health (SBDH) encompass factors such as socioeconomic status, access to healthy food, education, housing etc. that wield strong influence over an individual’s health outcomes^7^. Prior studies established strong relationships between SBDHs and suicidal behaviors^8–12^. For example, social disruptions (e.g., relationship dissolution, financial insecurity, legal problems, and exposure to childhood adversity) exhibit significant associations with suicidal behaviors^8,12–15^. However, leveraging SBDHs for predicting suicide has presented challenges, primarily due to the limitations in structured data sources, such as ICD codes, for capturing comprehensive and reliable SBDH information. Unstructured clinical notes, enriched with detailed SBDH information, can play a vital role in this regard^12,16^.

The increasing use of Electronic Health Records (EHR) in the US has stimulated efforts to identify patients at suicide risk using EHR data. This has resulted in data mining and machine learning approaches to predict suicidal behavior and suicide mortality among patients in large healthcare systems^17,18^. While most of the existing work on suicide risk assessment using SBDH has focused on structured data sources, unstructured EHR notes represent a relatively untapped data source that can be accessed relatively inexpensively. With the advent of advanced natural language processing (NLP) techniques, there are large opportunities to automate SBDH extraction from EHR notes to augment the structured data, aiding healthcare providers with a more holistic view of a patient’s overall health status and suicide risk^19,20^.

The US Department of Veterans Affairs (VA) operates the largest integrated healthcare network in the country, with a national EHR system used by >1200 medical centers and clinics^21^. With great public concern about the health of Veterans, the VA presents a unique opportunity to fully leverage its data for the exploration of suicide-related predictive modeling. In this study, we conducted the first retrospective case-control study to examine the impact of both structured and NLP-extracted (from unstructured notes) SBDH on suicide death among Veterans. We evaluated three architecturally distinct suicide prediction models across multiple prediction windows.

## Methods

### Data source and study design

In this study, we used inpatient and outpatient discharge data from the US Department of VA Veteran Health Administration (VHA) Corporate Data Warehouse. We included all discharges from outpatient emergency room and inpatient care between October 1, 2009 (start of Fiscal Year [FY] 2010) and September 30, 2015 (end of FY 2015), and following recent studies^22–24^, the unit of analysis was chosen to be hospital discharge. Our study protocol was approved by the institutional review board of VA Bedford Health Care. The Transparent Reporting of a Multivariable Prediction Model for Individual Prognosis or Diagnosis (TRIPOD)^25^ reporting guidelines were followed.

Cases were defined as discharges followed by deaths from suicide (according to National Death Index^26^ with International Classification of Diseases (ICD), Tenth Revision, codes X60–X84, Y87.0, and/or U03 as underlying cause of death) in the next D days (‘prediction window’). From each discharge, we established a 2-year retrospective ‘observation window’ to aggregate all relevant information for prediction. Each case was randomly matched, without replacement, to 5 discharges that were not followed by suicide in the prediction window (controls), on discharge type and date (±1 year). Our discharge inclusion criteria include (1) at least one diagnosis or procedure record within the observation window, (2) patients at least 18 years old with no conflicting date of birth (DOB) information, and (3) discharges at least D days before the study end date (September 30, 2015).

We analyzed 4 prediction windows 7, 30, 90, and 180 days, resulting in 4 cohorts. For each discharge, our task was to predict death by suicide within the prediction window, given all data from the observation window. We put aside the discharges from FY 2015 as the hold-out test data and used remaining discharges for training.

### Predictor construction

We categorized all predictors into four groups: demographics, codes, suicide behavioral (SB) information, and SBDH. The demographic predictors contain patients’ race, sex, age, and marital status. Codes include diagnosis codes, procedure codes, medication codes. Since diagnoses and procedure codes are hierarchical, encoding all of them may lead to overfitting. We therefore used the single-level Clinical Classification Software (CCS)^27^ to categorize them. This led to 283 categories for diagnosis codes and 248 categories for procedure codes. We also categorized medication codes following VA National Formulary^28^ (VANF) drug classification. More details are available in Supplementary Note 1. SB information includes suicide attempt (SA) and ideation (SI), obtained using the phenotype algorithm available through the VA’s Centralized Interactive Phenomics Resource (CIPHER)^29^.

SBDHs were identified from structured data using ICD-9 and VHA stop codes (structured SBDH), and clinical notes using NLP (NLP-extracted SBDH). Structured SBDHs included 6 factors—social or familial problems, employment or financial problems, housing instability, legal problems, violence, and non-specific psychosocial needs. NLP-extracted SBDHs were obtained from unstructured clinical notes using a transformer-based^30^ NLP system^12^, and comprised 12 factors—social isolation, job or financial insecurity, housing instability, legal problems, barriers to care, violence, transition of care, food insecurity, substance abuse, psychiatric symptoms, pain, and patient disability. SBDHs were extracted from the following 9 note types—emergency department notes, nursing assessments, primary care notes, hospital admission notes, inpatient progress notes, pain management, mental health notes, social worker notes, and discharge summaries. To assess the impact of SBDH from both sources, we combined them to create 13 distinct SBDH factors (Supplementary Note 2). We binarized each SBDH factor following the methods outlined by Mitra et al.^12^. In addition to individual-level SBDHs mentioned above, we also included neighborhood-level socioeconomic variable—area deprivation index (ADI)^31^ which represents the socioeconomic status of a patient’s neighborhood. ADI includes state and national-level rankings of neighborhoods based on socioeconomic disadvantages. A higher ADI indicates a lower socioeconomic status. We linked each patient’s EHR data to the ADI database via their address zip code and discharge quarter of the calendar year to identify the corresponding national-level ranking and included that as a predictor.

We extracted all predictors from the observation window except diagnosis codes, SB, and SBDH (excluding ADI). Diagnosis codes were extracted only from the discharge day as this yielded the best performance in our initial experiments. To capture prior documented SA and SI, we extracted SB data from any time before the current discharge date. Furthermore, we varied the timeframe for SBDH to investigate how their proximity to discharge affects subsequent suicide. We chose 7 (a week), 30 (a month), 90 (3 months), 180 (6 months), 365 (1 year), and 730 days (2 years) as candidate time frames. To provide the model a sense of time-variability, we also used SBDH predictors extracted from all six time windows simultaneously.

In summary, we considered 619 candidate predictors (Table 1): 4 demographic variables, 283 diagnoses codes variables, 248 procedures codes variables, 50 medication codes variables, 2 SB variables, 6 structured SBDH variables, 12 NLP-extracted SBDH variables, 13 combined SBDH variables, and 1 ADI variable. Demographic and ADI variables were categorical, whereas the remaining predictors were constructed as binary variables—indicating the absence or presence.

**Table 1:** List of predictors considered in our study

Predictor type	Description	Source
Demographic variables	Race, gender, age, and marital status	Structured data
SB variables	SA and SI	Structured data (using VA CIPHER’s phenotype algorithm)
Diagnoses codes variables	ICD-9 diagnoses codes grouped into 283 categories using CCS v2015	Structured data (ICD-9 diagnoses codes)
Procedure codes variables	Procedure codes grouped into 248 categories using CCS v2015 and CCS v2022.1	Structured data (ICD-9 procedure codes and CPT codes)
Medication codes variables	VANF classes grouped into 50 categories (Supplementary Note 1)	Structured data (VANF classes)
ADI	ADI database linked to the EHR data to obtain national-level ranking	Structured data (census/survey)
Structured SBDH	6 SBDHs extracted from structured data	Structured data (ICD-9 and VHA stop codes)
NLP-extracted SBDH	12 SBDHs extracted from 9 types of clinical notes using an NLP system^12^	Unstructured EHR notes

### Predictor screening

Predictor screening was performed on the binary features of diagnoses, procedure, and medication codes. First, we removed any of these predictors with a low prevalence of less than 1%. Next, for each remaining predictor, we fit a univariate logistic regression model of suicide death on the predictor and the demographic variables. We evaluated the *p* values of the predictors from these univariate models and used the Benjamini-Hochberg procedure^32^ to control the false discovery rate (FDR) at 10%. Only predictors with an adjusted *p* value smaller than 0.1 were used as candidate predictors to build the predictive models. Our two-stage screening reduced 87.4%, 78.51%, 75.77% and 71.41% of the predictors for the case-control cohorts with 7, 30, 90, and 180-day prediction windows respectively. Prior works suggest that predictor screening can help with noise reduction and substantially improve out-of-sample model performance^33,34^. SBDH variables were excluded from the screening stage as the focus of this work is on analyzing their impact on the prediction of suicide.

### Statistical analyses

We employed three different machine learning (ML) methods for predictive modeling, namely, elastic net logistic regression (ENL), random forest (RF), and multilayer perceptron (MLP). For the ENL and RF models, we used 10-fold cross-validation on the training data and performed grid searches over a wide range of hyperparameters to select the best models. For MLP, we used a 2-layer feed-forward network with ReLU^35^ as the activation function. To tune the hyperparameters of MLP, we set apart 20% of the training data as the validation set. As our cohorts had a case-control ratio of 1:5, we used cost-sensitive learning^36^ for all models to mitigate data imbalance. For ENL and RF, we averaged all metrics over 10 folds. For MLP, we averaged the model performance over three runs with different seeds. We experimented with different combinations of predictors, as shown in Tables 2 and 3. For SBDH, we experimented with the following combinations: structured SBDH, NLP-extracted SBDH, combined SBDH, structured SBDH + ADI, NLP-extracted SBDH + ADI, and Combined SBDH + ADI.

**Table 2:** Performance of different predictive models across different prediction windows for the best predictor configuration

Model	Risk group size, P	Sensitivity (SD), %	Specificity (SD), %	PPV (SD), %	Adjusted PPV (SD), %
Prediction window = 7					
ENL	0.05	16.42 (0.58)	97.48 (0.12)	57.67 (2.03)	0.03 (0.00)
	0.10	29.27 (0.83)	94.06 (0.17)	50.80 (1.44)	0.02 (0.00)
	0.20	48.74 (1.85)	86.07 (0.39)	42.30 (1.61)	0.02 (0.00)
	0.60	88.68 (0.46)	46.03 (0.10)	25.60 (0.13)	0.01 (0.00)
RF	0.05	12.98 (1.03)	96.75 (0.22)	45.58 (3.63)	0.02 (0.00)
	0.10	24.97 (0.73)	93.16 (0.15)	43.33 (1.26)	0.02 (0.00)
	0.20	47.22 (1.62)	85.76 (0.34)	40.98 (1.41)	0.02 (0.00)
	0.60	88.41 (1.27)	45.98 (0.26)	25.53 (0.37)	0.01 (0.00)
MLP	0.05	15.89 (0.54)	97.36 (0.11)	55.81 (1.90)	0.03 (0.00)
	0.10	27.15 (1.43)	93.62 (0.30)	47.13 (2.48)	0.02 (0.00)
	0.20	48.57 (3.30)	86.04 (0.69)	42.15 (2.87)	0.02 (0.00)
	0.60	82.78 (1.87)	44.80 (0.39)	23.90 (0.54)	0.01 (0.00)
Prediction window = 30					
ENL	0.05	18.59 (0.48)	97.83 (0.10)	64.00 (1.66)	0.12 (0.01)
	0.10	32.68 (0.57)	94.72 (0.12)	56.26 (0.98)	0.09 (0.00)
	0.20	53.86 (0.69)	87.04 (0.14)	46.37 (0.59)	0.06 (0.00)
	0.60	89.39 (0.32)	46.11 (0.07)	25.65 (0.09)	0.02 (0.00)
RF	0.05	15.28 (0.82)	97.14 (0.17)	52.61 (2.81)	0.07 (0.01)
	0.10	28.28 (0.79)	93.80 (0.16)	48.70 (1.36)	0.06 (0.00)
	0.20	51.52 (1.15)	86.55 (0.24)	44.35 (0.99)	0.05 (0.00)
	0.60	88.06 (0.87)	45.84 (0.18)	25.27 (0.25)	0.02 (0.00)
MLP	0.05	15.40 (0.74)	97.16 (0.15)	53.04 (2.56)	0.08 (0.01)
	0.10	26.09 (1.14)	93.35 (0.24)	44.93 (1.96)	0.05 (0.00)
	0.20	40.32 (1.02)	84.23 (0.21)	34.71 (0.88)	0.04 (0.00)
	0.60	79.88 (1.33)	44.14 (0.28)	22.92 (0.38)	0.02 (0.00)
Prediction window = 90					
ENL	0.05	21.86 (0.16)	98.28 (0.03)	71.12 (0.53)	0.38 (0.01)
	0.10	38.19 (0.43)	95.48 (0.08)	62.13 (0.71)	0.25 (0.01)
	0.20	56.74 (0.50)	87.14 (0.10)	46.15 (0.41)	0.13 (0.00)
	0.60	90.25 (0.49)	45.89 (0.10)	24.47 (0.13)	0.05 (0.00)
RF	0.05	23.20 (0.28)	98.54 (0.05)	75.48 (0.91)	0.47 (0.02)
	0.10	38.46 (0.47)	95.53 (0.09)	62.56 (0.76)	0.26 (0.01)
	0.20	58.24 (0.48)	87.43 (0.09)	47.37 (0.39)	0.14 (0.00)
	0.60	92.31 (0.55)	46.29 (0.11)	25.03 (0.15)	0.05 (0.00)
MLP	0.05	20.80 (0.27)	98.07 (0.05)	67.68 (0.86)	0.32 (0.01)
	0.10	33.44 (0.97)	94.56 (0.19)	54.40 (1.58)	0.18 (0.01)
	0.20	51.38 (1.93)	86.10 (0.37)	41.79 (1.57)	0.11 (0.01)
	0.60	88.09 (1.57)	45.47 (0.31)	23.89 (0.43)	0.05 (0.00)
Prediction window = 180					
ENL	0.05	23.20 (0.28)	98.54 (0.05)	75.48 (0.91)	0.47 (0.02)
	0.10	37.54 (0.22)	95.46 (0.04)	62.01 (0.36)	0.41 (0.01)
	0.20	56.31 (0.15)	87.19 (0.03)	46.45 (0.12)	0.22 (0.00)
	0.60	92.00 (0.21)	46.33 (0.04)	25.28 (0.06)	0.08 (0.00)
RF	0.05	24.58 (0.29)	98.88 (0.06)	81.21 (0.97)	1.07 (0.07)
	0.10	41.14 (0.41)	96.17 (0.08)	67.95 (0.67)	0.53 (0.02)
	0.20	60.86 (0.57)	88.09 (0.11)	50.21 (0.47)	0.25 (0.00)
	0.60	92.36 (0.58)	46.40 (0.11)	25.38 (0.16)	0.08 (0.00)
MLP	0.05	19.82 (1.44)	97.94 (0.28)	65.48 (4.74)	0.48 (0.10)
	0.10	33.29 (1.46)	94.62 (0.29)	54.99 (2.41)	0.31 (0.03)
	0.20	50.77 (1.06)	86.09 (0.21)	41.88 (0.87)	0.18 (0.01)
	0.60	87.61 (0.34)	45.46 (0.07)	24.07 (0.09)	0.08 (0.00)

*PPV* positive predictive value, *ENL* elastic net logistic regression, *RF* random forest, *MLP* multilayer perceptron.

**Table 3:** Performance of different predictive models, including ensembled systems, across different prediction windows

Prediction window	Models	ROC AUC, %	PR AUC, %
7	ENL	77.75	41.76
	RF	76.66	38.40
	MLP	75.59	42.87
	Ensemble (ENL + RF)	78.46	41.78
	Ensemble (ENL + RF + MLP)	78.39	43.72
30	ENL	78.96	46.94
	RF	78.50	43.48
	MLP	72.87	40.97
	Ensemble (ENL + RF)	79.91	48.51
	Ensemble (ENL + RF + MLP)	78.74	46.47
90	ENL	80.98	52.81
	RF	83.27	55.88
	MLP	79.37	48.55
	Ensemble (ENL + RF)	82.90	56.45
	Ensemble (ENL + RF + MLP)	82.94	55.53
180	ENL	82.94	53.52
	RF	84.36	60.67
	MLP	79.31	48.65
	Ensemble (ENL + RF)	84.23	58.75
	Ensemble (ENL + RF + MLP)	84.12	58.09

*ROC AUC* area under the receiver operator characteristic curve, PR *AUC* area under the precision-recall curve.

To evaluate the models’ predictive performance on the test data, we examined various performance metrics on the test data, including the area under the receiver operator characteristic curve (ROC AUC), area under the precision recall curve (PR AUC), sensitivity, specificity, and positive predictive value (PPV). Since suicide is a rare event, we calculated sensitivity, specificity, and PPV for different risk group sizes. A risk group size P for a predictive model indicates the fraction of the test set with the highest risk for suicide, as identified by the model. Following prior studies^22,37^ and our data statistics, we included 0.05, 0.10, 0.20, and 0.60 as different values for P. As this is a case-control study, we also reported adjusted PPV^38^ with the general population. PPV denotes the probability of predicted high-risk patients with suicide death. The measurement of PPV is important as this indicates the chances of saving patients’ lives with interventions. We remark that any given operating point on the ROC curve (i.e., a particular sensitivity–specificity pair) from the case-control study remains valid with the general population, given that the selected controls are representative of the non-suicide population. However, for the same specificity, the fraction of subjects labeled “high-risk” in the general population may be notably smaller than in the case-control sample.

In addition, we conducted calibration analysis (measured integration calibration index [ICI]^39^) and measured predictor importance using the Kernel SHAP (Shapley Additive Explanations) method^40^ for the best predictor configuration. For each model, we chose PR AUC to select the best hyperparameter configuration. All analyses used Python 3.8, ENL and RF were implemented using scikit-learn^41^ 0.23.1, and MLP was implemented using PyTorch^42^ 1.5.1.

## Results

### Prevalence of suicide

**Fig. 1:**
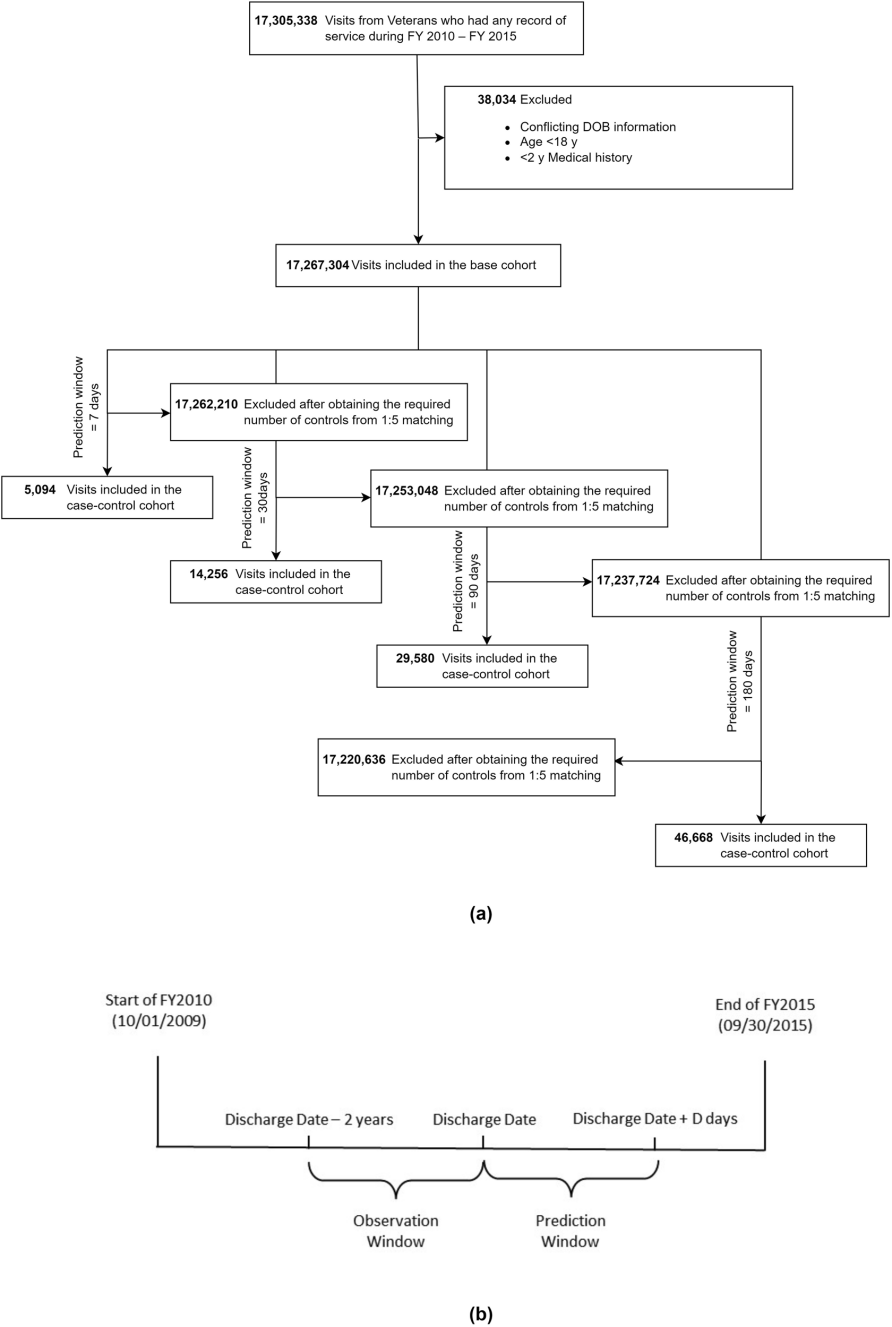
An overview of our study design. **a** Construction of the cohort and **b** the study timeline; *D* = {7, 30, 90, 180} (in days).

Out of 17,267,304 discharges from 2,987,006 Veterans (Fig. 1), 17,210,996 (99.67%) were eligible to be considered for the 7-day prediction with 849 cases, amounting to 0.005% suicide rate at the discharge level. At the patient level, the suicide rate within 7 days of discharge was 0.03%, with 849 suicide deaths from 2,703,173 patients. Similarly, the suicide rates within 180 days of discharge were 0.05% at the discharge level and 0.27% at the patient level. In summary, the 4 case-control cohorts for prediction windows 7, 30, 90, and 180 days consisted of 5094 (849 cases and 4245 controls), 14,256 (2376 cases and 11,880 controls), 29,580 (4930 cases and 24,650 controls) and 46,668 discharges (7778 cases and 38,890 controls) respectively. More details are available in Supplementary Note 3.

### Overall model performance

**Fig. 2:**
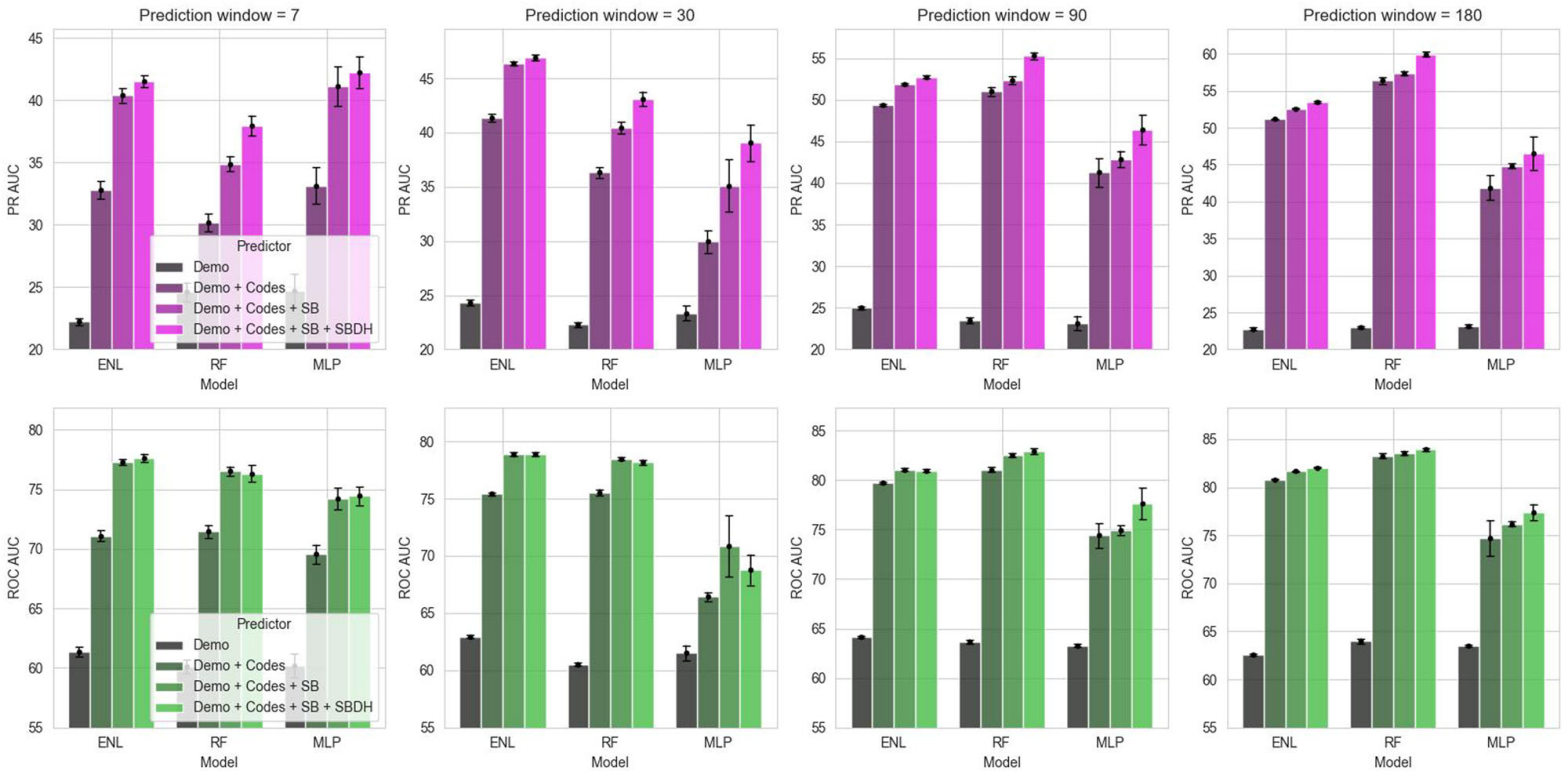
Performance of different predictive models across different prediction windows. ROC AUC area under the receiver operator characteristic curve, PR AUC area under the precision recall curve, SD standard deviation, Demo demographic variables, SB suicidal behaviors—attempt and ideation, Codes diagnosis, procedure, and medication codes, SBDH social and behavioral determinants of health, ENL elastic net logistic regression, RF random forest, MLP multilayer perceptron.

With ‘SBDH’ as predictors, we only reported results for the combinations that yielded the best PR AUC scores. We noticed incremental improvements across almost all models and prediction windows as we added a new predictor group. Adding codes and SB information always improved the AUC scores (Fig. 2). A similar trend can also be observed with SBDHs. For example, adding codes to demographic predictors improved the PR AUC for ENL by 47.66% (95% CI = 45.49–49.82, *p* val <0.001) in the 7-day prediction window. Adding SB predictors improved the PR AUC further by 23.13% (95% CI = 22.21–24.05, *p* val <0.001), and adding SBDH predictors yielded another 2.78% improvements (95% CI = 2.38–3.19, *p* val <0.001). The performance gain from SBDH predictors was consistent across all models and predictor windows. For instance, we observed 8.79% (95% CI = 7.01–10.57, *p* val <0.001), 6.55% (95% CI = 5.84–7.26, *p* val <0.001), 5.63% (95% CI = 5.01–6.25, *p* val <0.001), and 4.47% (95% CI = 4.02–4.92, *p* val <0.001) improvements in PR AUC for RF across 7, 30, 90 and 180-day prediction windows respectively after including SBDH. It is worth noting that the best SBDH setting for PR AUC did not always yield the best ROC AUC score.

ENL achieved the best AUC scores for the 7 and 30-day prediction windows, except MLP attaining the best PR AUC in the 7-day prediction window. In contrast, RF achieved the best AUCs across 90 and 180-day prediction windows. In general, models for the shortest prediction window (7 days) had the lowest ROC AUCs (74.44%–77.65%), and as prediction windows got longer, the models performed better with the highest ROC AUCs (77.39%–83.94%) obtained for the longest prediction window (180 days). PR AUC scores demonstrated a similar trend. AUC scores were almost always higher among outpatient ED discharges than inpatient discharges (Supplementary Table 5).

Across all prediction windows with the best predictor configuration, these models detected 12.98% to 24.58% of all deaths from suicide at the 5% risk tier or 96.75–98.88% specificity (Table 2). This means that even considering only 5% of the discharges with the highest model-assigned suicide risk, a suicide intervention program based on these models can capture 12.98–24.58% of patient discharges where the patients would otherwise die by suicide. Increasing the risk group size or reducing the specificity can help capture even more discharges, for example, 24.97%–41.14% at a 10% risk group size or 93.16–96.17% specificity. ENL demonstrated the highest sensitivity and specificity across all risk tiers for shorter prediction windows (7 and 30 days), whereas RF outperformed other models for longer prediction windows (90 and 180 days). PPVs and adjusted PPVs increase as the prediction window increases and the risk group size decreases. We obtained the highest adjusted PPV of 1.07% for the RF model with SBDH (0.91% without SBDH) over the 180-day prediction window at 98.88% specificity. This suggests that at 98.88% specificity, patients from 1.07% of discharges would die by suicide within 180 days of their hospital discharges in the absence of any additional intervention program.

We also present results for a subset of the test set consisting of discharges from patients not present in the training data (Supplementary Tables 6 and 7). This evaluation was conducted to assess model performance only on previously unseen patients. The results show a performance trend similar to the complete test set, with incremental improvements observed as new predictor groups are added. However, the AUC scores are slightly lower for longer prediction windows, and the previously observed monotonic relationship between prediction window length and AUC is no longer apparent. It is important to note that patients who died by suicide tend to have a higher number of discharges, resulting in a lower percentage of case patients in this test subset for longer prediction windows compared to the complete test set (e.g., 12.8% case patients in the subset vs. 16.8% in the full test set for the 180-day prediction window).

### Impact of NLP-extracted predictors

In this study, we used an NLP system to extract SBDH from clinical notes. We compared our NLP-extracted SBDHs with structured and combined SBDHs. Supplementary Table 8 outlines all the SBDH combinations that yielded the best performance for each model at a specific prediction window. In 50% of the settings (6 out of 12), NLP-extracted SBDHs appeared as the best choice, whereas structured SBDH performed better in four settings. For example, ENL and RF achieved 2.78% (95% CI = 2.38–3.19, *p* val <0.001) and 8.79% (95% CI = 7.01–10.57, *p* val <0.001) improvements in PR AUC after including NLP-extracted SBDH as predictors for the shortest (7-day) prediction window (Fig. 2 and Supplementary Table 5). For the longest (180-day) prediction window, the improvements were 0.70% (95% CI = 0.65–0.74, *p* val <0.001) and 4.34% (95% CI = 3.86–4.82, *p* val <0.001) respectively (Supplementary Table 5). For MLP, the inclusion of SBDH predictors did not always yield statistically significant gains. In most settings, ADI was found to be helpful.

### Calibration and predictor importance

Out of the three models, RF is the best-calibrated model (Supplementary Fig. 1). The addition of SBDH predictors marginally improved ICI for ENL and MLP but did not yield any improvement for RF. We also measured predictor importance using Kernel SHAP method (Supplementary Fig. 3). Based on SHAP values, we identified predictors that pushed a model towards making positive predictions (suicide death) and predictors that did the opposite. We named them positive and negative predictors, respectively. Upon examining the top 30 positive predictors, we found that SA, SI, and the age group 79 or higher are the most common predictors across different models and prediction windows. In contrast, black race, female gender, and age 50–59 were the most consistent negative predictors in the top 30. Among diagnoses predictors, ‘Administrative/social admission’ (conditions related to SDOH, suicidality, and administrative encounters; underlying ICD codes that overlapped with structured SBDH were removed, more details in Supplementary Note 1), ‘COPD’, ‘alcohol-related disorders’, and ‘anxiety disorders’ were the most common positive predictors. As for procedure categories, ‘anesthesia’ was a common positive predictor, whereas ‘cardiac stress tests’ was a common negative predictor. Among medications, ‘sedative hypnotics’ was a prominent positive predictor and ‘antidepressants’ was a common negative predictor. Among SBDHs, ‘Social isolation’ (NLP-extracted) and ‘violence’ (structured) were two of the most common positive predictors. We would like to emphasize that SHAP values do not indicate risk or protective factors; rather, they help rank predictors according to their usefulness for a task (suicide prediction) with respect to a model (ENL, RF, or MLP).

### Ensemble learning

Ensembling is a popular technique for aggregating multiple models’ predictions to improve system robustness. Among various aggregator functions such as linear averaging, majority voting, boosting, etc., we chose linear averaging for our study. First, for each model, we averaged the prediction probabilities over all folds/runs, and then, we averaged them over different models. We did this for the two best models (ENL and RF) and all three models. The results are shown in Table 3. We found that ensembling ENL and RF improved the AUC scores over the best single models for 7, 30, and 90-days prediction windows. However, the performance did not improve for the 180 days prediction window. Comparatively, ensembling all models was only helpful for prediction window 7. Overall, the RF model is still better calibrated than the ensembled systems (Supplementary Fig. 2).

## Discussion

To the authors’ knowledge, this is the first case-control study to examine the roles of NLP-extracted SBDHs in predicting suicide among US Veterans. We found that models with SBDH predictors outperformed models without SBDH in AUCs. For example, the RF model with no SBDH achieved an ROC AUC of 83.57% and a PR AUC of 57.38% in the 180-day prediction window. After adding ADI and NLP-extracted SBDH (timeframe = 730 days), the AUCs increased to 84.25% (0.81% improvement, 95% CI = 0.63–0.98, *p* val <0.001) and 59.87% (4.34% improvement, 95% CI = 3.86–4.82, *p* val <0.001) respectively. Moreover, when compared with structured SBDH, NLP-extracted SBDHs yielded competitive or better performance in most situations.

Our findings suggest that SBDH improved performance for all cases, with ROC AUC improvements going up to 3.86% and PR AUC improvements up to 11.21%. This is consistent with prior studies^22,24^ where multiple SBDH factors were identified as important predictors for suicide after discharge from VA psychiatric hospitalization. However, they lacked a robust deep-learning-based SBDH extraction system from clinical notes. Our results also showed that all models benefited from including NLP-extracted SBDHs in combination with other SDBHs or alone (Supplementary Table 5). This highlights the merit of harnessing clinical notes through NLP to enrich SBDH information for improved predictive modeling. Although adding SBDH predictors improved sensitivity, specificity, and PPV in most settings, the improvements were not always statistically significant. This may be attributed to model hyperparameters being tuned based on PR AUC.

Our work showed that near-term prediction of suicide death is more challenging than longer-time predictions; as such, all models performed the worst with 7-day prediction window, and the performance generally improved as the prediction window increased. This may partly stem from the lack of adequate samples in shorter prediction windows, making it more challenging for any model to map the predictors to suicide. Other studies suggested that a larger number of suicides over longer windows increases predictive models’ statistical power^22,24^. They found that models built to predict suicide over longer windows outperform models built to predict over shorter windows when applied to those shorter windows.

To assess the significance of the predictors, we ranked them using their SHAP values (Supplementary Fig. 3). We discovered that records of prior SA and SI were among the most influential predictors for death by suicide across all prediction windows. SA is a well-established risk factor for suicide^3,43^, with data showing that one out of every 100 attempt survivors dies from suicide within the first year, a risk approximately 100 times higher than that of the general population^44^. Furthermore, the risk of suicide can extend up to 32 years following an attempt^45^. A systematic review of 90 studies found a 6.7% suicide completion rate and a 23% non-fatal attempt rate^46^. Among demographic predictors, ‘divorced’ marital status and ‘white’ race emerged as positive predictors in most settings, while ‘married’ marital status, ‘female’ sex, and ‘black’ race were identified as negative predictors. Recent reports on both the general US population^47^ and Veterans^4^ showed a higher suicide rate in males than females and in individuals identifying as white compared to those identifying as black. Moreover, a meta-analysis of 36 studies found that divorced individuals face a higher suicide risk compared to non-married individuals, while married individuals have a lower risk than their non-married counterparts^48^.

Among the top 20 positive predictors (Supplementary Fig. 3), we found ‘social isolation’ and ‘violence’ as two of the most common SBDH predictors. Prior studies demonstrated a higher association between social isolation and elevated suicide risk^12,49–52^, while exposure to violence is also a well-established risk factor for suicidality^12,53,54^. However, it is worth noting that many SBDH factors may serve as risk markers rather than causal risk factors^55^. For instance, according to SHAP values, ‘housing instability’ and ‘employment or financial problems’ were identified as negative SBDH predictors in certain prediction windows for most of the models despite both homelessness and unemployment being associated with increased suicidality^8,12,56,57^. This apparent contradiction may be explained by several factors. First, patients experiencing housing instability or financial difficulties were simultaneously exposed to a range of other risk factors, leading to subadditive multivariate interactions within the models^22^. Second, patients experiencing such SBDH factors may have a higher likelihood of hospitalization compared to those without, leading to lower suicide risk among hospitalized patients with these SBDH conditions, especially for psychiatric inpatient discharges^24^. Third, there might exist more rigorous post-discharge intervention processes targeting these SBDH conditions. Each of these possibilities calls for further investigation to obtain a more comprehensive understanding of the relationship between SBDH and suicide death.

Using NLP to extract clinically relevant information from EHR notes is not new. Datta et al. reviewed 78 studies that utilized NLP to extract cancer-related information^58^. Mitra et al.^12^ developed a deep-learning-based NLP system to extract eight social determinants of health from EHR notes and found significant associations between those and suicide among US Veterans. Bhanu et al. designed an NLP system to extract SB information from EHR notes^59^. Many other works also used NLP systems to detect suicidality in EHR notes^60–63^. However, ours is the first case-control study to incorporate NLP-extracted SBDHs as predictors for suicide death prediction.

Although predicting suicidal behavior has been an active area of research^17,22,33,64,65^, our study differs in the addition of NLP-extracted SBDH as predictors to analyze their impact on a diverse set of models’ predictive performance. Despite many existing studies on the prediction of suicide, integrating their findings into existing healthcare systems poses a multitude of challenges, such as lack of logistics support at the deployment centers, risk-benefit tradeoff, cost-effectiveness, a sense of false reassurance^22^, and generalizability, among others. Moreover, a systematic review of 17 suicide prediction studies found that all predictive models suffer from low PPV, regardless of the population distribution or risk tier^66^, thus, making suicide prediction a challenging task.^24^

A low PPV or high false positive in suicide prediction models can cause significant ethical, psychological, and resource-related challenges in clinical settings. Such systems might make patients feel emotional distress, loss of trust, and stigmatization, while healthcare systems may face unnecessary interventions, increased costs, and strained resources. Ethical dilemmas alongside legal and liability concerns highlight the need for improving model specificity, PPV and using predictive tools as aids rather than definitive decision-makers. However, Kessler et al. showed that predictive models have positive net benefit (NB) across plausible ranges of the PPV distribution^24^.

Our study has several limitations. Firstly, the VA population’s demographic composition differs from that of the overall US population. VHA also lacks community-based hospitalizations and, sometimes, ED visit data. Nonetheless, research utilizing VHA data has informed non-VA facilities in implementing enhanced clinical practices^67–69^. Additionally, our study employed no VA-exclusive predictors, allowing for the extraction of the same predictors from EHRs at non-VA facilities for customized prediction models. Secondly, generalization of our findings across time might be tricky due to factors such as the adoption of ICD-10 codes, healthcare-related changes due to the COVID-19 pandemic, recent changes in VHA EHR systems, etc. We will investigate the effects of SBDH in a post-pandemic cohort in our next work. Thirdly, our analysis focused solely on outpatient emergency and inpatient care discharges. Expanding to include other hospital settings could enhance our comprehension of SBDHs’ impact on suicide. We leave this for our future work. Fourthly, we restricted the observation window to 2 years to incorporate relatively current SBDHs but extending it to encompass historical SBDHs may enhance model predictions, a subject we will explore in future research. Fifthly, several studies suggest the use of NB instead of AUCs or PPV as a better measure to evaluate and design suicide predictive models^24,70^. NB explicitly measures the benefits of intervening with true positives against the cost of intervening with false positives. We leave this as future work. Lastly, we utilized the ADI, available only at the census tract block group level; however, we plan to investigate the recently proposed social vulnerability metric^71^ as an alternative in future studies.

Ours is the first large-scale study to use NLP-extracted SBDH information from unstructured EHR data to predict suicide among Veterans. We showed that incorporating NLP-extracted SBDH exhibited improved predictive performance across different models and prediction windows. Consequently, integrating NLP-extracted SBDH into structured EHR data holds a promising avenue for the advancement of a more effective suicide prevention system.

## Supplementary information

Supplementary Information

## Data Availability

The VHA EHR data are available under restricted access for Veterans' privacy and data security laws, access can be obtained by relevant approvals through VA Informatics and Computing Infrastructure (VINCI) (contact: VINCI@va.gov). Individuals who wish to use this data for research purposes must fulfill the research credentialing requirements as outlined by the VA Office of Research and Development.
