# Supplementary material for: Post-discharge suicide prediction among US veterans using natural language processing-enriched social and behavioral determinants of health
Source: Npj Ment Health Res. 2025 Feb 22;4:8. doi: 10.1038/s44184-025-00120-2 (PMC11846906; doi:10.1038/s44184-025-00120-2)
Supplement: Supplementary file 1 — Supplementary Information [file 44184_2025_120_MOESM1_ESM.docx]

Contents

[**Supplementary Note 1: Categorization of Diagnoses, Procedure and Medication Codes** 2](#_Toc182049044)

[**Diagnoses Categories** 2](#_Toc182049045)

[**Procedure Categories** 44](#_Toc182049046)

[**Medication Categories** 106](#_Toc182049047)

[**Supplementary Note 2: Combining Structured and NLP-extracted SBDH** 107](#_Toc182049048)

[**Supplementary Note 3: Data Statistics** 107](#_Toc182049049)

[**Supplementary Note 4: Results** 111](#_Toc182049050)

[**Supplementary Note 5: Impact of NLP-extracted Predictors** 118](#_Toc182049051)

[**Supplementary Note 6: Calibration and Predictor Importance** 119](#_Toc182049052)

[References 123](#_Toc182049053)

# **Supplementary Note 1: Categorization of Diagnoses, Procedure and Medication Codes**

We used the single-level Clinical Classification Software (CCS) ^[[1]](#footnote-1)^ to categorize diagnosis and procedure codes. Following CCS v2015, we grouped all ICD-9 diagnoses codes into 283 classes (eTable 1). ICD-9 codes that were used to extract SBDHs were removed before this grouping to avoid redundancy. CCS was also used to group ICD-9 Procedure codes (CCS v2015) into 231 categories and the Current Procedure Terminology (CPT) codes (CCS v2022.1) into 245 categories. Most categories between these two sets of codes overlap and therefore, the union of them was considered to create 248 distinct procedure categories (eTable 2). A similar process was adopted to categorize medications. After removing all missing values, central nervous system medications were grouped into 19 categories^1^, and other medications were grouped into 31 categories based on VA National Formulary^2^ (VANF) drug classification (eTable 3). Specifically, we categorized each prescribed medication based on the first two characters of its VA class.

## **Diagnoses Categories**

| CCS Category | CCS Category Label | ICD-9 Codes |
| --- | --- | --- |
| 1 | Tuberculosis | 01000, 01001, 01002, 01003, 01004, 01005, 01006, 01010, 01011, 01012, 01013, 01014, 01015, 01016, 01080, 01081, 01082, 01083, 01084, 01085, 01086, 01090, 01091, 01092, 01093, 01094, 01095, 01096, 01100, 01101, 01102, 01103, 01104, 01105, 01106, 01110, 01111, 01112, 01113, 01114, 01115, 01116, 01120, 01121, 01122, 01123, 01124, 01125, 01126, 01130, 01131, 01132, 01133, 01134, 01135, 01136, 01140, 01141, 01142, 01143, 01144, 01145, 01146, 01150, 01151, 01152, 01153, 01154, 01155, 01156, 01160, 01161, 01162, 01163, 01164, 01165, 01166, 01170, 01171, 01172, 01173, 01174, 01175, 01176, 01180, 01181, 01182, 01183, 01184, 01185, 01186, 01190, 01191, 01192, 01193, 01194, 01195, 01196, 01200, 01201, 01202, 01203, 01204, 01205, 01206, 01210, 01211, 01212, 01213, 01214, 01215, 01216, 01220, 01221, 01222, 01223, 01224, 01225, 01226, 01230, 01231, 01232, 01233, 01234, 01235, 01236, 01280, 01281, 01282, 01283, 01284, 01285, 01286, 01300, 01301, 01302, 01303, 01304, 01305, 01306, 01310, 01311, 01312, 01313, 01314, 01315, 01316, 01320, 01321, 01322, 01323, 01324, 01325, 01326, 01330, 01331, 01332, 01333, 01334, 01335, 01336, 01340, 01341, 01342, 01343, 01344, 01345, 01346, 01350, 01351, 01352, 01353, 01354, 01355, 01356, 01360, 01361, 01362, 01363, 01364, 01365, 01366, 01380, 01381, 01382, 01383, 01384, 01385, 01386, 01390, 01391, 01392, 01393, 01394, 01395, 01396, 01400, 01401, 01402, 01403, 01404, 01405, 01406, 01480, 01481, 01482, 01483, 01484, 01485, 01486, 01500, 01501, 01502, 01503, 01504, 01505, 01506, 01510, 01511, 01512, 01513, 01514, 01515, 01516, 01520, 01521, 01522, 01523, 01524, 01525, 01526, 01550, 01551, 01552, 01553, 01554, 01555, 01556, 01560, 01561, 01562, 01563, 01564, 01565, 01566, 01570, 01571, 01572, 01573, 01574, 01575, 01576, 01580, 01581, 01582, 01583, 01584, 01585, 01586, 01590, 01591, 01592, 01593, 01594, 01595, 01596, 01600, 01601, 01602, 01603, 01604, 01605, 01606, 01610, 01611, 01612, 01613, 01614, 01615, 01616, 01620, 01621, 01622, 01623, 01624, 01625, 01626, 01630, 01631, 01632, 01633, 01634, 01635, 01636, 01640, 01641, 01642, 01643, 01644, 01645, 01646, 01650, 01651, 01652, 01653, 01654, 01655, 01656, 01660, 01661, 01662, 01663, 01664, 01665, 01666, 01670, 01671, 01672, 01673, 01674, 01675, 01676, 01690, 01691, 01692, 01693, 01694, 01695, 01696, 01700, 01701, 01702, 01703, 01704, 01705, 01706, 01710, 01711, 01712, 01713, 01714, 01715, 01716, 01720, 01721, 01722, 01723, 01724, 01725, 01726, 01730, 01731, 01732, 01733, 01734, 01735, 01736, 01740, 01741, 01742, 01743, 01744, 01745, 01746, 01750, 01751, 01752, 01753, 01754, 01755, 01756, 01760, 01761, 01762, 01763, 01764, 01765, 01766, 01770, 01771, 01772, 01773, 01774, 01775, 01776, 01780, 01781, 01782, 01783, 01784, 01785, 01786, 01790, 01791, 01792, 01793, 01794, 01795, 01796, 01800, 01801, 01802, 01803, 01804, 01805, 01806, 01880, 01881, 01882, 01883, 01884, 01885, 01886, 01890, 01891, 01892, 01893, 01894, 01895, 01896, 1370, 1371, 1372, 1373, 1374, V1201 |
| 2 | Septicemia (except in labor) | 0031, 0202, 0223, 0362, 0380, 0381, 03810, 03811, 03812, 03819, 0382, 0383, 03840, 03841, 03842, 03843, 03844, 03849, 0388, 0389, 0545, 449, 77181, 7907, 99591, 99592 |
| 3 | Bacterial infection; unspecified site | 0200, 0208, 0209, 0218, 0219, 0228, 0229, 0230, 0231, 0232, 0233, 0238, 0239, 024, 025, 0260, 0269, 0270, 0271, 0272, 0278, 0279, 0300, 0301, 0302, 0303, 0308, 0309, 0312, 0318, 0319, 03289, 0329, 0330, 0331, 0338, 0339, 0341, 0363, 03681, 03689, 0369, 037, 0392, 0393, 0394, 0398, 0399, 0400, 0401, 0402, 0403, 04042, 04081, 04082, 04089, 0410, 04100, 04101, 04102, 04103, 04104, 04105, 04109, 0411, 04110, 04111, 04112, 04119, 0412, 0413, 0414, 04141, 04142, 04143, 04149, 0415, 0416, 0417, 0418, 04181, 04182, 04183, 04184, 04185, 04186, 04189, 0419, 390, 3929, 7953, 79531, 79539, V090, V091, V092, V093, V094, V0950, V0951, V096, V0970, V0971, V0980, V0981, V0990, V0991, V1204 |
| 4 | Mycoses | 1100, 1101, 1102, 1103, 1104, 1105, 1106, 1108, 1109, 1110, 1111, 1112, 1113, 1118, 1119, 1120, 1121, 1122, 1123, 1125, 11282, 11284, 11285, 11289, 1129, 1141, 1143, 1149, 11500, 11509, 11510, 11519, 11590, 11599, 1160, 1161, 1162, 1170, 1171, 1172, 1173, 1174, 1175, 1176, 1177, 1178, 1179, 118 |
| 5 | HIV infection | 042, 0420, 0421, 0422, 0429, 0430, 0431, 0432, 0433, 0439, 0440, 0449, 07953, 27910, 27919, 79571, 7958, V08 |
| 6 | Hepatitis | 0700, 0701, 0702, 07020, 07021, 07022, 07023, 0703, 07030, 07031, 07032, 07033, 0704, 07041, 07042, 07043, 07044, 07049, 0705, 07051, 07052, 07053, 07054, 07059, 0706, 07070, 07071, 0709, 07271, 57140, 57141, 57142, 57149, 5731, 5732, 5733 |
| 7 | Viral infection | 0500, 0501, 0502, 0509, 0510, 05101, 05102, 0511, 0512, 0519, 0522, 0527, 0528, 0529, 05310, 05311, 05312, 05313, 05314, 05319, 05371, 05379, 0538, 0539, 0540, 05410, 05411, 05412, 05413, 05419, 0542, 0546, 05471, 05473, 05474, 05479, 0548, 0549, 05579, 0558, 0559, 05600, 05609, 05679, 0568, 0569, 0570, 0578, 0579, 05810, 05811, 05812, 05881, 05882, 05889, 05900, 05901, 05909, 05910, 05911, 05912, 05919, 05920, 05921, 05922, 0598, 0599, 0600, 0601, 0609, 061, 0650, 0651, 0652, 0653, 0654, 0658, 0659, 0660, 0661, 0663, 0664, 06640, 06642, 06649, 0668, 0669, 071, 0720, 0723, 07272, 07279, 0728, 0729, 0737, 0738, 0739, 0740, 0741, 0743, 0748, 075, 0780, 0781, 07810, 07811, 07812, 07819, 0782, 0783, 0784, 0785, 0786, 0787, 07881, 07882, 07888, 07889, 0790, 0791, 0792, 0793, 0794, 07950, 07951, 07952, 07959, 0796, 0798, 07981, 07982, 07983, 07988, 07989, 0799, 07998, 07999, 7908 |
| 8 | Other infections; including parasitic | 080, 0810, 0811, 0812, 0819, 0820, 0821, 0822, 0823, 08240, 08241, 08249, 0828, 0829, 0831, 0832, 0838, 0839, 0840, 0841, 0842, 0843, 0844, 0845, 0846, 0847, 0848, 0849, 0850, 0851, 0852, 0853, 0854, 0855, 0859, 0860, 0861, 0862, 0863, 0864, 0865, 0869, 0870, 0871, 0879, 0880, 0888, 08881, 08882, 08889, 0889, 1000, 10089, 1009, 101, 1020, 1021, 1022, 1023, 1024, 1025, 1026, 1027, 1028, 1029, 1030, 1031, 1032, 1033, 1039, 1040, 1048, 1049, 1200, 1201, 1202, 1203, 1208, 1209, 1210, 1211, 1212, 1213, 1214, 1215, 1216, 1218, 1219, 1220, 1221, 1222, 1223, 1224, 1225, 1226, 1227, 1228, 1229, 1230, 1231, 1232, 1233, 1234, 1235, 1236, 1238, 1239, 124, 1250, 1251, 1252, 1253, 1254, 1255, 1256, 1257, 1259, 1260, 1261, 1262, 1263, 1268, 1269, 1270, 1271, 1272, 1273, 1274, 1275, 1276, 1277, 1278, 1279, 1280, 1281, 1288, 1289, 129, 1305, 1307, 1308, 1309, 13100, 13101, 13102, 13103, 13109, 1318, 1319, 1320, 1321, 1322, 1323, 1329, 1330, 1338, 1339, 1340, 1341, 1342, 1348, 1349, 135, 1360, 1361, 1362, 13621, 13629, 1364, 1365, 1368, 1369, 1398, V120, V1200, V1203, V1209 |
| 9 | Sexually transmitted infections (not HIV or hepatitis) | 0900, 0901, 0902, 0903, 09040, 09041, 09042, 09049, 0905, 0906, 0907, 0909, 0910, 0911, 0912, 0913, 0914, 09150, 09151, 09152, 09161, 09162, 09169, 0917, 09181, 09182, 09189, 0919, 0920, 0929, 0930, 0931, 09320, 09321, 09322, 09323, 09324, 09381, 09382, 09389, 0939, 0940, 0941, 0942, 0943, 09481, 09482, 09483, 09484, 09485, 09486, 09487, 09489, 0949, 0950, 0951, 0952, 0953, 0954, 0955, 0956, 0957, 0958, 0959, 096, 0970, 0971, 0979, 0980, 09810, 09811, 09812, 09813, 09814, 09815, 09816, 09817, 09819, 0982, 09830, 09831, 09832, 09833, 09834, 09835, 09836, 09837, 09839, 09840, 09841, 09842, 09843, 09849, 09850, 09851, 09852, 09853, 09859, 0986, 0987, 09881, 09882, 09883, 09884, 09885, 09886, 09889, 0990, 0991, 0992, 0993, 0994, 09940, 09941, 09949, 09950, 09951, 09952, 09953, 09954, 09955, 09956, 09959, 0998, 0999, 79505, 79515, 79519, 79675, 79679 |
| 10 | Immunizations and screening for infectious disease | 7955, 79551, 79552, 7956, 79579, V010, V011, V012, V013, V014, V015, V016, V017, V0171, V0179, V018, V0181, V0182, V0183, V0184, V0189, V019, V020, V021, V022, V023, V024, V025, V0251, V0252, V0253, V0254, V0259, V026, V0260, V0261, V0262, V0269, V027, V028, V029, V030, V031, V032, V033, V034, V035, V036, V037, V038, V0381, V0382, V0389, V039, V040, V041, V042, V043, V044, V045, V046, V047, V048, V0481, V0482, V0489, V050, V051, V052, V053, V054, V058, V059, V060, V061, V062, V063, V064, V065, V066, V068, V069, V286, V712, V7182, V7183, V730, V731, V732, V733, V734, V735, V736, V738, V7381, V7388, V7389, V739, V7398, V7399, V740, V741, V742, V743, V744, V745, V746, V748, V749, V750, V751, V752, V753, V754, V755, V756, V757, V758, V759 |
| 11 | Cancer of head and neck | 1400, 1401, 1403, 1404, 1405, 1406, 1408, 1409, 1410, 1411, 1412, 1413, 1414, 1415, 1416, 1418, 1419, 1420, 1421, 1422, 1428, 1429, 1430, 1431, 1438, 1439, 1440, 1441, 1448, 1449, 1450, 1451, 1452, 1453, 1454, 1455, 1456, 1458, 1459, 1460, 1461, 1462, 1463, 1464, 1465, 1466, 1467, 1468, 1469, 1470, 1471, 1472, 1473, 1478, 1479, 1480, 1481, 1482, 1483, 1488, 1489, 1490, 1491, 1498, 1499, 1600, 1601, 1602, 1603, 1604, 1605, 1608, 1609, 1610, 1611, 1612, 1613, 1618, 1619, 1950, 2300, 2310, V1001, V1002, V1021 |
| 12 | Cancer of esophagus | 1500, 1501, 1502, 1503, 1504, 1505, 1508, 1509, 2301, V1003 |
| 13 | Cancer of stomach | 1510, 1511, 1512, 1513, 1514, 1515, 1516, 1518, 1519, 20923, 2302, V1004 |
| 14 | Cancer of colon | 1530, 1531, 1532, 1533, 1534, 1535, 1536, 1537, 1538, 1539, 1590, 20910, 20911, 20912, 20913, 20914, 20915, 20916, 2303, V1005 |
| 15 | Cancer of rectum and anus | 1540, 1541, 1542, 1543, 1548, 20917, 2304, 2305, 2306, 79670, 79671, 79672, 79673, 79674, 79676, V1006 |
| 16 | Cancer of liver and intrahepatic bile duct | 1550, 1551, 1552, 2308, V1007 |
| 17 | Cancer of pancreas | 1570, 1571, 1572, 1573, 1574, 1578, 1579 |
| 18 | Cancer of other GI organs; peritoneum | 1520, 1521, 1522, 1523, 1528, 1529, 1560, 1561, 1562, 1568, 1569, 1580, 1588, 1589, 1591, 1598, 1599, 20900, 20901, 20902, 20903, 2307, 2309, V1000, V1009 |
| 19 | Cancer of bronchus; lung | 1622, 1623, 1624, 1625, 1628, 1629, 20921, 2312, V1011 |
| 20 | Cancer; other respiratory and intrathoracic | 1620, 1630, 1631, 1638, 1639, 1650, 1658, 1659, 2311, 2318, 2319, V1012, V1020, V1022 |
| 21 | Cancer of bone and connective tissue | 1700, 1701, 1702, 1703, 1704, 1705, 1706, 1707, 1708, 1709, 1710, 1712, 1713, 1714, 1715, 1716, 1717, 1718, 1719 |
| 22 | Melanomas of skin | 1720, 1721, 1722, 1723, 1724, 1725, 1726, 1727, 1728, 1729, V1082 |
| 23 | Other non-epithelial cancer of skin | 1730, 17300, 17301, 17302, 17309, 1731, 17310, 17311, 17312, 17319, 1732, 17320, 17321, 17322, 17329, 1733, 17330, 17331, 17332, 17339, 1734, 17340, 17341, 17342, 17349, 1735, 17350, 17351, 17352, 17359, 1736, 17360, 17361, 17362, 17369, 1737, 17370, 17371, 17372, 17379, 1738, 17380, 17381, 17382, 17389, 1739, 17390, 17391, 17392, 17399, 20931, 20932, 20933, 20934, 20935, 20936, 2320, 2321, 2322, 2323, 2324, 2325, 2326, 2327, 2328, 2329, V1083 |
| 24 | Cancer of breast | 1740, 1741, 1742, 1743, 1744, 1745, 1746, 1748, 1749, 1750, 1759, 2330, V103 |
| 25 | Cancer of uterus | 179, 1820, 1821, 1828, 2332, V1042 |
| 26 | Cancer of cervix | 1800, 1801, 1808, 1809, 2331, 7950, 79501, 79502, 79503, 79504, 79506, V1041 |
| 27 | Cancer of ovary | 1830, V1043 |
| 28 | Cancer of other female genital organs | 181, 1832, 1833, 1834, 1835, 1838, 1839, 1840, 1841, 1842, 1843, 1844, 1848, 1849, 2333, 23330, 23331, 23332, 23339, 79516, V1040, V1044 |
| 29 | Cancer of prostate | 185, 2334, V1046 |
| 30 | Cancer of testis | 1860, 1869, V1047 |
| 31 | Cancer of other male genital organs | 1871, 1872, 1873, 1874, 1875, 1876, 1877, 1878, 1879, 2335, 2336, V1045, V1048, V1049 |
| 32 | Cancer of bladder | 1880, 1881, 1882, 1883, 1884, 1885, 1886, 1887, 1888, 1889, 2337, V1051 |
| 33 | Cancer of kidney and renal pelvis | 1890, 1891, 20924, V1052, V1053 |
| 34 | Cancer of other urinary organs | 1892, 1893, 1894, 1898, 1899, 2339, V1050, V1059 |
| 35 | Cancer of brain and nervous system | 1910, 1911, 1912, 1913, 1914, 1915, 1916, 1917, 1918, 1919, 1920, 1921, 1922, 1923, 1928, 1929, V1085, V1086 |
| 36 | Cancer of thyroid | 193, 25802, 25803, V1087 |
| 37 | Hodgkin`s disease | 20100, 20101, 20102, 20103, 20104, 20105, 20106, 20107, 20108, 20110, 20111, 20112, 20113, 20114, 20115, 20116, 20117, 20118, 20120, 20121, 20122, 20123, 20124, 20125, 20126, 20127, 20128, 20140, 20141, 20142, 20143, 20144, 20145, 20146, 20147, 20148, 20150, 20151, 20152, 20153, 20154, 20155, 20156, 20157, 20158, 20160, 20161, 20162, 20163, 20164, 20165, 20166, 20167, 20168, 20170, 20171, 20172, 20173, 20174, 20175, 20176, 20177, 20178, 20190, 20191, 20192, 20193, 20194, 20195, 20196, 20197, 20198, V1072 |
| 38 | Non-Hodgkin`s lymphoma | 20000, 20001, 20002, 20003, 20004, 20005, 20006, 20007, 20008, 20010, 20011, 20012, 20013, 20014, 20015, 20016, 20017, 20018, 20020, 20021, 20022, 20023, 20024, 20025, 20026, 20027, 20028, 20030, 20031, 20032, 20033, 20034, 20035, 20036, 20037, 20038, 20040, 20041, 20042, 20043, 20044, 20045, 20046, 20047, 20048, 20050, 20051, 20052, 20053, 20054, 20055, 20056, 20057, 20058, 20060, 20061, 20062, 20063, 20064, 20065, 20066, 20067, 20068, 20070, 20071, 20072, 20073, 20074, 20075, 20076, 20077, 20078, 20080, 20081, 20082, 20083, 20084, 20085, 20086, 20087, 20088, 20200, 20201, 20202, 20203, 20204, 20205, 20206, 20207, 20208, 20210, 20211, 20212, 20213, 20214, 20215, 20216, 20217, 20218, 20220, 20221, 20222, 20223, 20224, 20225, 20226, 20227, 20228, 20270, 20271, 20272, 20273, 20274, 20275, 20276, 20277, 20278, 20280, 20281, 20282, 20283, 20284, 20285, 20286, 20287, 20288, 20290, 20291, 20292, 20293, 20294, 20295, 20296, 20297, 20298, V1071, V1079 |
| 39 | Leukemias | 20240, 20241, 20242, 20243, 20244, 20245, 20246, 20247, 20248, 2031, 20310, 20311, 20312, 2040, 20400, 20401, 20402, 2041, 20410, 20411, 20412, 2042, 20420, 20421, 20422, 2048, 20480, 20481, 20482, 2049, 20490, 20491, 20492, 2050, 20500, 20501, 20502, 2051, 20510, 20511, 20512, 2052, 20520, 20521, 20522, 2053, 20530, 20531, 20532, 2058, 20580, 20581, 20582, 2059, 20590, 20591, 20592, 2060, 20600, 20601, 20602, 2061, 20610, 20611, 20612, 2062, 20620, 20621, 20622, 2068, 20680, 20681, 20682, 2069, 20690, 20691, 20692, 2070, 20700, 20701, 20702, 2071, 20710, 20711, 20712, 2072, 20720, 20721, 20722, 2078, 20780, 20781, 20782, 2080, 20800, 20801, 20802, 2081, 20810, 20811, 20812, 2082, 20820, 20821, 20822, 2088, 20880, 20881, 20882, 2089, 20890, 20891, 20892, V1060, V1061, V1062, V1063, V1069 |
| 40 | Multiple myeloma | 2030, 20300, 20301, 20302, 2038, 20380, 20381, 20382 |
| 41 | Cancer; other and unspecified primary | 1640, 1641, 1642, 1643, 1648, 1649, 1760, 1761, 1762, 1763, 1764, 1765, 1768, 1769, 1900, 1901, 1902, 1903, 1904, 1905, 1906, 1907, 1908, 1909, 1940, 1941, 1943, 1944, 1945, 1946, 1948, 1949, 1951, 1952, 1953, 1954, 1955, 1958, 20230, 20231, 20232, 20233, 20234, 20235, 20236, 20237, 20238, 20250, 20251, 20252, 20253, 20254, 20255, 20256, 20257, 20258, 20260, 20261, 20262, 20263, 20264, 20265, 20266, 20267, 20268, 20922, 20925, 20926, 20927, 2340, 2348, 2349, 7951, 79510, 79511, 79512, 79513, 79514, V1029, V1081, V1084, V1088, V1089, V109, V1090, V1091, V711 |
| 42 | Secondary malignancies | 1960, 1961, 1962, 1963, 1965, 1966, 1968, 1969, 1970, 1971, 1972, 1973, 1974, 1975, 1976, 1977, 1978, 1980, 1981, 1982, 1983, 1984, 1985, 1986, 1987, 19881, 19882, 19889, 20971, 20972, 20973, 20974, 51181, 78951 |
| 43 | Malignant neoplasm without specification of site | 1990, 1991, 1992, 20920, 20929, 20930, 20970, 20975, 20979 |
| 44 | Neoplasms of unspecified nature or uncertain behavior | 2350, 2351, 2352, 2353, 2354, 2355, 2356, 2357, 2358, 2359, 2360, 2361, 2362, 2363, 2364, 2365, 2366, 2367, 23690, 23691, 23699, 2370, 2371, 2372, 2373, 2374, 2375, 2376, 2377, 23770, 23771, 23772, 23773, 23779, 2379, 2380, 2381, 2382, 2383, 2384, 2385, 2386, 2387, 23871, 23872, 23873, 23874, 23875, 23876, 23877, 23879, 2388, 2389, 2390, 2391, 2392, 2393, 2394, 2395, 2396, 2397, 2398, 23981, 23989, 2399 |
| 45 | Maintenance chemotherapy; radiotherapy | V580, V581, V5811, V5812, V661, V662, V671, V672 |
| 46 | Benign neoplasm of uterus | 2180, 2181, 2182, 2189, 2190, 2191, 2198, 2199 |
| 47 | Other and unspecified benign neoplasm | 20940, 20941, 20942, 20943, 20950, 20951, 20952, 20953, 20954, 20955, 20956, 20957, 20960, 20961, 20962, 20963, 20964, 20965, 20966, 20967, 20969, 2100, 2101, 2102, 2103, 2104, 2105, 2106, 2107, 2108, 2109, 2110, 2111, 2112, 2113, 2114, 2115, 2116, 2117, 2118, 2119, 2120, 2121, 2122, 2123, 2124, 2125, 2126, 2127, 2128, 2129, 2130, 2131, 2132, 2133, 2134, 2135, 2136, 2137, 2138, 2139, 2140, 2141, 2142, 2143, 2144, 2148, 2149, 2150, 2152, 2153, 2154, 2155, 2156, 2157, 2158, 2159, 2160, 2161, 2162, 2163, 2164, 2165, 2166, 2167, 2168, 2169, 217, 220, 2210, 2211, 2212, 2218, 2219, 2220, 2221, 2222, 2223, 2224, 2228, 2229, 2230, 2231, 2232, 2233, 22381, 22389, 2239, 2240, 2241, 2242, 2243, 2244, 2245, 2246, 2247, 2248, 2249, 2250, 2251, 2252, 2253, 2254, 2258, 2259, 226, 2270, 2271, 2273, 2274, 2275, 2276, 2278, 2279, 22800, 22801, 22802, 22803, 22804, 22809, 2281, 2290, 2298, 2299, V1272 |
| 48 | Thyroid disorders | 2400, 2409, 2410, 2411, 2419, 24200, 24201, 24210, 24211, 24220, 24221, 24230, 24231, 24240, 24241, 24280, 24281, 24290, 24291, 243, 2440, 2441, 2442, 2443, 2448, 2449, 2450, 2451, 2452, 2453, 2454, 2458, 2459, 2460, 2461, 2462, 2463, 2468, 2469, 7945 |
| 49 | Diabetes mellitus without complication | 24900, 25000, 25001, 7902, 79021, 79022, 79029, 7915, 7916, V4585, V5391, V6546 |
| 50 | Diabetes mellitus with complications | 24901, 24910, 24911, 24920, 24921, 24930, 24931, 24940, 24941, 24950, 24951, 24960, 24961, 24970, 24971, 24980, 24981, 24990, 24991, 25002, 25003, 25010, 25011, 25012, 25013, 25020, 25021, 25022, 25023, 25030, 25031, 25032, 25033, 25040, 25041, 25042, 25043, 25050, 25051, 25052, 25053, 25060, 25061, 25062, 25063, 25070, 25071, 25072, 25073, 25080, 25081, 25082, 25083, 25090, 25091, 25092, 25093 |
| 51 | Other endocrine disorders | 2510, 2511, 2512, 2513, 2514, 2515, 2518, 2519, 2520, 25200, 25201, 25202, 25208, 2521, 2528, 2529, 2530, 2531, 2532, 2533, 2534, 2535, 2536, 2537, 2538, 2539, 2540, 2541, 2548, 2549, 2550, 2551, 25510, 25511, 25512, 25513, 25514, 2552, 2553, 2554, 25541, 25542, 2555, 2556, 2558, 2559, 2560, 2561, 2562, 2563, 2564, 2568, 2569, 2570, 2571, 2572, 2578, 2579, 2580, 25801, 2581, 2588, 2589, 2590, 2591, 2592, 2593, 2594, 2595, 25950, 25951, 25952, 2598, 2599, 7946 |
| 52 | Nutritional deficiencies | 260, 261, 262, 2630, 2631, 2632, 2638, 2639, 2640, 2641, 2642, 2643, 2644, 2645, 2646, 2647, 2648, 2649, 2650, 2651, 2652, 2660, 2661, 2662, 2669, 267, 2680, 2681, 2682, 2689, 2690, 2691, 2692, 2693, 2698, 2699, 7994, V121 |
| 53 | Disorders of lipid metabolism | 2720, 2721, 2722, 2723, 2724 |
| 54 | Gout and other crystal arthropathies | 2740, 27400, 27401, 27402, 27403, 27410, 27411, 27419, 27481, 27482, 27489, 2749, 71210, 71211, 71212, 71213, 71214, 71215, 71216, 71217, 71218, 71219, 71220, 71221, 71222, 71223, 71224, 71225, 71226, 71227, 71228, 71229, 71230, 71231, 71232, 71233, 71234, 71235, 71236, 71237, 71238, 71239, 71280, 71281, 71282, 71283, 71284, 71285, 71286, 71287, 71288, 71289, 71290, 71291, 71292, 71293, 71294, 71295, 71296, 71297, 71298, 71299 |
| 55 | Fluid and electrolyte disorders | 2760, 2761, 2762, 2763, 2764, 2765, 27650, 27651, 27652, 2766, 27669, 2767, 2768, 2769, 9951 |
| 56 | Cystic fibrosis | 27700, 27701, 27702, 27703, 27709 |
| 57 | Immunity disorders | 27900, 27901, 27902, 27903, 27904, 27905, 27906, 27909, 27911, 27912, 27913, 2792, 2793, 2794, 27941, 27949, 2798, 2799 |
| 58 | Other nutritional; endocrine; and metabolic disorders | 2700, 2701, 2702, 2703, 2704, 2705, 2706, 2707, 2708, 2709, 2710, 2711, 2712, 2713, 2714, 2718, 2719, 2725, 2726, 2727, 2728, 2729, 2730, 2731, 2732, 2733, 2734, 2738, 2739, 2750, 27501, 27502, 27503, 27509, 2751, 2752, 2753, 2754, 27540, 27541, 27542, 27549, 2755, 2758, 2759, 2771, 2772, 2773, 27730, 27731, 27739, 2774, 2775, 2776, 2777, 2778, 27781, 27782, 27784, 27785, 27786, 27787, 27789, 2779, 2780, 27800, 27801, 27802, 27803, 2781, 2782, 2783, 2784, 2788, 7831, 7832, 78321, 78322, 7833, 7834, 78340, 78341, 78342, 78343, 7835, 7837, 7839, 79391, 7947, 7957, V122, V1221, V1229, V850, V8521, V8522, V8523, V8524, V8525, V8530, V8531, V8532, V8533, V8534, V8535, V8536, V8537, V8538, V8539, V854, V8541, V8542, V8543, V8544, V8545, V8551, V8553, V8554 |
| 59 | Deficiency and other anemia | 2800, 2801, 2808, 2809, 2810, 2811, 2812, 2813, 2814, 2818, 2819, 2820, 2821, 2822, 2823, 2824, 28240, 28243, 28244, 28245, 28246, 28247, 28249, 2827, 2828, 2829, 2830, 2831, 28310, 28311, 28319, 2832, 2839, 2840, 28401, 28409, 2841, 28411, 28412, 28419, 2842, 2848, 28481, 28489, 2849, 2850, 28521, 28522, 28529, 2858, 2859 |
| 60 | Acute posthemorrhagic anemia | 2851 |
| 61 | Sickle cell anemia | 28241, 28242, 2825, 28260, 28261, 28262, 28263, 28264, 28268, 28269 |
| 62 | Coagulation and hemorrhagic disorders | 2860, 2861, 2862, 2863, 2864, 2865, 28652, 28653, 28659, 2866, 2867, 2869, 2870, 2871, 2872, 2873, 28730, 28731, 28732, 28733, 28739, 2874, 28749, 2875, 2878, 2879, 28981, 28982, 28984, 7827 |
| 63 | Diseases of white blood cells | 2880, 28800, 28801, 28802, 28803, 28804, 28809, 2881, 2882, 2883, 2884, 28850, 28851, 28859, 28860, 28861, 28862, 28863, 28864, 28865, 28866, 28869, 2888, 2889, 28953 |
| 64 | Other hematologic conditions | 2890, 2894, 28950, 28951, 28952, 28959, 2896, 2897, 2898, 28983, 28989, 2899, 7900, 79001, 79009, V123, V582 |
| 76 | Meningitis (except that caused by tuberculosis or sexually transmitted disease) | 00321, 0360, 0470, 0471, 0478, 0479, 0490, 0491, 0530, 05472, 0721, 10081, 11283, 1142, 11501, 11511, 11591, 3200, 3201, 3202, 3203, 3207, 3208, 32081, 32082, 32089, 3209, 3210, 3211, 3212, 3213, 3214, 3218, 3220, 3221, 3222, 3229 |
| 77 | Encephalitis (except that caused by tuberculosis or sexually transmitted disease) | 0361, 0462, 0498, 0499, 0520, 0543, 0550, 05601, 05821, 05829, 0620, 0621, 0622, 0623, 0624, 0625, 0628, 0629, 0630, 0631, 0632, 0638, 0639, 064, 0662, 06641, 0722, 1300, 1390, 3230, 32301, 32302, 3231, 3232, 3234, 32341, 32342, 3235, 32351, 32352, 3236, 32361, 32362, 32363, 3237, 32371, 32372, 3238, 32381, 32382, 3239, 34120, 34121, 34122 |
| 78 | Other CNS infection and poliomyelitis | 04500, 04501, 04502, 04503, 04510, 04511, 04512, 04513, 04520, 04521, 04522, 04523, 04590, 04591, 04592, 04593, 0460, 0461, 04611, 04619, 0463, 04671, 04672, 04679, 0468, 0469, 048, 138, 3240, 3241, 3249, 326, V1202 |
| 79 | Parkinson`s disease | 3320 |
| 80 | Multiple sclerosis | 340 |
| 81 | Other hereditary and degenerative nervous system conditions | 3300, 3301, 3302, 3303, 3308, 3309, 3313, 3314, 3315, 3316, 3317, 33181, 33189, 3319, 3330, 3331, 3332, 3333, 3334, 3335, 3336, 3337, 33371, 33372, 33379, 33381, 33382, 33383, 33384, 33385, 33389, 33390, 33391, 33393, 33394, 33399, 3340, 3341, 3342, 3343, 3344, 3348, 3349, 3350, 33510, 33511, 33519, 33520, 33521, 33522, 33523, 33524, 33529, 3358, 3359, 3360, 3361, 3362, 3363, 3368, 3369, 3370, 33700, 33701, 33709, 3371, 3373, 3379 |
| 82 | Paralysis | 3420, 34200, 34201, 34202, 3421, 34210, 34211, 34212, 34280, 34281, 34282, 3429, 34290, 34291, 34292, 3430, 3431, 3432, 3433, 3434, 3438, 3439, 3440, 34400, 34401, 34402, 34403, 34404, 34409, 3441, 3442, 3443, 34430, 34431, 34432, 3444, 34440, 34441, 34442, 3445, 34460, 3448, 34481, 34489, 3449, 78072, 7814 |
| 83 | Epilepsy; convulsions | 3450, 34500, 34501, 3451, 34510, 34511, 3452, 3453, 3454, 34540, 34541, 3455, 34550, 34551, 3456, 34560, 34561, 3457, 34570, 34571, 3458, 34580, 34581, 3459, 34590, 34591, 7803, 78031, 78032, 78033, 78039 |
| 84 | Headache; including migraine | 33900, 33901, 33902, 33903, 33904, 33905, 33909, 33910, 33911, 33912, 33920, 33921, 33922, 3393, 33941, 33942, 33943, 33944, 33981, 33982, 33983, 33984, 33985, 33989, 3460, 34600, 34601, 34602, 34603, 3461, 34610, 34611, 34612, 34613, 3462, 34620, 34621, 34622, 34623, 34630, 34631, 34632, 34633, 34640, 34641, 34642, 34643, 34650, 34651, 34652, 34653, 34670, 34671, 34672, 34673, 3468, 34680, 34681, 34682, 34683, 3469, 34690, 34691, 34692, 34693, 7840 |
| 85 | Coma; stupor; and brain damage | 3481, 7800, 78001, 78003, 78009 |
| 86 | Cataract | 36600, 36601, 36602, 36603, 36604, 36609, 36610, 36611, 36612, 36613, 36614, 36615, 36616, 36617, 36618, 36619, 36620, 36621, 36622, 36623, 36630, 36631, 36632, 36633, 36634, 36641, 36642, 36643, 36644, 36645, 36646, 36650, 36651, 36652, 36653, 3668, 3669, V431 |
| 87 | Retinal detachments; defects; vascular occlusion; and retinopathy | 36100, 36101, 36102, 36103, 36104, 36105, 36106, 36107, 36110, 36111, 36112, 36113, 36114, 36119, 3612, 36130, 36131, 36132, 36133, 36181, 36189, 3619, 36201, 36202, 36203, 36204, 36205, 36206, 36207, 36210, 36211, 36212, 36213, 36214, 36215, 36216, 36217, 36218, 36220, 36221, 36222, 36223, 36224, 36225, 36226, 36227, 36229, 36230, 36231, 36232, 36233, 36234, 36235, 36236, 36237, 36240, 36241, 36242, 36243, 36250, 36251, 36252, 36253, 36254, 36255, 36256, 36257, 36260, 36261, 36262, 36263, 36264, 36265, 36266, 36270, 36271, 36272, 36273, 36274, 36275, 36276, 36277, 36281, 36282, 36283, 36284, 36285, 36289, 3629 |
| 88 | Glaucoma | 36500, 36501, 36502, 36503, 36504, 36505, 36506, 36510, 36511, 36512, 36513, 36514, 36515, 36520, 36521, 36522, 36523, 36524, 36531, 36532, 36541, 36542, 36543, 36544, 36551, 36552, 36559, 36560, 36561, 36562, 36563, 36564, 36565, 36570, 36571, 36572, 36573, 36574, 36581, 36582, 36583, 36589, 3659 |
| 89 | Blindness and vision defects | 3670, 3671, 36720, 36721, 36722, 36731, 36732, 3674, 36751, 36752, 36753, 36781, 36789, 3679, 36800, 36801, 36802, 36803, 36810, 36811, 36812, 36813, 36814, 36815, 36816, 3682, 36830, 36831, 36832, 36833, 36834, 36840, 36841, 36842, 36843, 36844, 36845, 36846, 36847, 36851, 36852, 36853, 36854, 36855, 36859, 36860, 36861, 36862, 36863, 36869, 3688, 3689, 36900, 36901, 36902, 36903, 36904, 36905, 36906, 36907, 36908, 36910, 36911, 36912, 36913, 36914, 36915, 36916, 36917, 36918, 36920, 36921, 36922, 36923, 36924, 36925, 3693, 3694, 36960, 36961, 36962, 36963, 36964, 36965, 36966, 36967, 36968, 36969, 36970, 36971, 36972, 36973, 36974, 36975, 36976, 3698, 3699, V410 |
| 90 | Inflammation; infection of eye (except that caused by tuberculosis or sexually transmitted disease) | 0213, 03281, 05320, 05321, 05322, 05329, 05440, 05441, 05442, 05443, 05444, 05449, 05571, 0760, 0761, 0769, 0770, 0771, 0772, 0773, 0774, 0778, 0779, 07798, 07799, 11502, 11512, 11592, 1301, 1302, 1391, 36000, 36001, 36002, 36003, 36004, 36011, 36012, 36013, 36014, 36019, 36300, 36301, 36303, 36304, 36305, 36306, 36307, 36308, 36310, 36311, 36312, 36313, 36314, 36315, 36320, 36321, 36322, 36400, 36401, 36402, 36403, 36404, 36405, 36410, 36411, 36421, 36422, 36423, 36424, 3643, 37020, 37021, 37022, 37023, 37024, 37031, 37032, 37033, 37034, 37035, 37040, 37044, 37049, 37050, 37052, 37054, 37055, 37059, 3708, 3709, 37200, 37201, 37202, 37203, 37204, 37205, 37206, 37210, 37211, 37212, 37213, 37214, 37215, 37220, 37221, 37222, 37230, 37231, 37233, 37239, 37300, 37301, 37302, 37311, 37312, 37313, 37331, 37332, 37333, 37334, 3734, 3735, 3736, 3738, 3739, 37500, 37501, 37502, 37503, 37530, 37531, 37532, 37533, 37541, 37542, 37543, 37600, 37601, 37602, 37603, 37604, 37610, 37611, 37612, 37613, 37730, 37731, 37732, 37733, 37734, 37739, 37900, 37901, 37902, 37903, 37904, 37905, 37906, 37907, 37909, 37960, 37961, 37962, 37963 |
| 91 | Other eye disorders | 36020, 36021, 36023, 36024, 36029, 36030, 36031, 36032, 36033, 36034, 36040, 36041, 36042, 36043, 36044, 36050, 36051, 36052, 36053, 36054, 36055, 36059, 36060, 36061, 36062, 36063, 36064, 36065, 36069, 36081, 36089, 3609, 36330, 36331, 36332, 36333, 36334, 36335, 36340, 36341, 36342, 36343, 36350, 36351, 36352, 36353, 36354, 36355, 36356, 36357, 36361, 36362, 36363, 36370, 36371, 36372, 3638, 3639, 36441, 36442, 36451, 36452, 36453, 36454, 36455, 36456, 36457, 36459, 36460, 36461, 36462, 36463, 36464, 36470, 36471, 36472, 36473, 36474, 36475, 36476, 36477, 3648, 36481, 36482, 36489, 3649, 37000, 37001, 37002, 37003, 37004, 37005, 37006, 37007, 37060, 37061, 37062, 37063, 37064, 37100, 37101, 37102, 37103, 37104, 37105, 37110, 37111, 37112, 37113, 37114, 37115, 37116, 37120, 37121, 37122, 37123, 37124, 37130, 37131, 37132, 37133, 37140, 37141, 37142, 37143, 37144, 37145, 37146, 37148, 37149, 37150, 37151, 37152, 37153, 37154, 37155, 37156, 37157, 37158, 37160, 37161, 37162, 37170, 37171, 37172, 37173, 37181, 37182, 37189, 3719, 37234, 37240, 37241, 37242, 37243, 37244, 37245, 37250, 37251, 37252, 37253, 37254, 37255, 37256, 37261, 37262, 37263, 37264, 37271, 37272, 37273, 37274, 37275, 3728, 37281, 37289, 3729, 3732, 37400, 37401, 37402, 37403, 37404, 37405, 37410, 37411, 37412, 37413, 37414, 37420, 37421, 37422, 37423, 37430, 37431, 37432, 37433, 37434, 37441, 37443, 37444, 37445, 37446, 37450, 37451, 37452, 37453, 37454, 37455, 37456, 37481, 37482, 37483, 37484, 37485, 37486, 37487, 37489, 3749, 37511, 37512, 37513, 37514, 37515, 37516, 37520, 37521, 37522, 37551, 37552, 37553, 37554, 37555, 37556, 37557, 37561, 37569, 37581, 37589, 3759, 37621, 37622, 37630, 37631, 37632, 37633, 37634, 37635, 37636, 37640, 37641, 37642, 37643, 37644, 37645, 37646, 37647, 37650, 37651, 37652, 3766, 37681, 37682, 37689, 3769, 37700, 37701, 37702, 37703, 37704, 37710, 37711, 37712, 37713, 37714, 37715, 37716, 37721, 37722, 37723, 37724, 37741, 37742, 37743, 37749, 37751, 37752, 37753, 37754, 37761, 37762, 37763, 37771, 37772, 37773, 37775, 3779, 37800, 37801, 37802, 37803, 37804, 37805, 37806, 37807, 37808, 37810, 37811, 37812, 37813, 37814, 37815, 37816, 37817, 37818, 37820, 37821, 37822, 37823, 37824, 37830, 37831, 37832, 37833, 37834, 37835, 37840, 37841, 37842, 37843, 37844, 37845, 37850, 37851, 37852, 37853, 37854, 37855, 37856, 37860, 37861, 37862, 37863, 37871, 37872, 37873, 37881, 37882, 37883, 37884, 37885, 37886, 37887, 3789, 37911, 37912, 37913, 37914, 37915, 37916, 37919, 37921, 37922, 37923, 37924, 37925, 37926, 37927, 37929, 37931, 37932, 37933, 37934, 37939, 37940, 37941, 37942, 37943, 37945, 37946, 37949, 37950, 37951, 37952, 37953, 37954, 37955, 37956, 37957, 37958, 37959, 3798, 37990, 37991, 37992, 37993, 37999, 78193, V411, V425, V430, V456, V4561, V4569, V522, V531, V720 |
| 92 | Otitis media and related conditions | 0552, 38100, 38101, 38102, 38103, 38104, 38105, 38106, 38110, 38119, 38120, 38129, 3813, 3814, 38150, 38151, 38152, 38160, 38161, 38162, 38163, 3817, 38181, 38189, 3819, 38200, 38201, 38202, 3821, 3822, 3823, 3824, 3829, 38300, 38301, 38302, 3831, 38320, 38321, 38322, 38330, 38331, 38332, 38333, 38381, 38389, 3839, 38420, 38421, 38422, 38423, 38424, 38425, 38481, 38482, 3849, 38500, 38501, 38502, 38503, 38509, 38510, 38511, 38512, 38513, 38519, 38521, 38522, 38523, 38524, 3870, 3871, 3872, 3878, 3879 |
| 93 | Conditions associated with dizziness or vertigo | 38600, 38601, 38602, 38603, 38604, 38610, 38611, 38612, 38619, 3862, 38630, 38631, 38632, 38633, 38634, 38635, 38640, 38641, 38642, 38643, 38648, 38650, 38651, 38652, 38653, 38654, 38655, 38656, 38658, 3868, 3869, 7804 |
| 94 | Other ear and sense organ disorders | 38000, 38001, 38002, 38003, 38010, 38011, 38012, 38013, 38014, 38015, 38016, 38021, 38022, 38023, 38030, 38031, 38032, 38039, 3804, 38050, 38051, 38052, 38053, 38081, 38089, 3809, 38400, 38401, 38409, 3841, 38530, 38531, 38532, 38533, 38535, 38582, 38583, 38589, 3859, 38800, 38801, 38802, 38810, 38811, 38812, 3882, 38830, 38831, 38832, 38840, 38841, 38842, 38843, 38844, 38845, 3885, 38860, 38861, 38869, 38870, 38871, 38872, 3888, 3889, 38900, 38901, 38902, 38903, 38904, 38905, 38906, 38908, 38910, 38911, 38912, 38913, 38914, 38915, 38916, 38917, 38918, 3892, 38920, 38921, 38922, 3897, 3898, 3899, V412, V413, V4985, V532, V721, V7211, V7212, V7219 |
| 95 | Other nervous system disorders | 325, 32702, 32715, 32730, 32731, 32732, 32733, 32734, 32735, 32736, 32737, 32739, 32753, 33183, 3321, 33720, 33721, 33722, 33729, 3380, 33811, 33812, 33818, 33819, 33821, 33822, 33828, 33829, 3383, 3384, 3410, 3411, 3418, 3419, 34461, 347, 34700, 34701, 34710, 34711, 3480, 3482, 3483, 34830, 34831, 34839, 3484, 3485, 3488, 34881, 34882, 34889, 3489, 3492, 34981, 34982, 34989, 3499, 3501, 3502, 3508, 3509, 3510, 3511, 3518, 3519, 3520, 3521, 3522, 3523, 3524, 3525, 3526, 3529, 3530, 3531, 3532, 3533, 3534, 3535, 3536, 3538, 3539, 3540, 3541, 3542, 3543, 3544, 3545, 3548, 3549, 3550, 3551, 3552, 3553, 3554, 3555, 3556, 3557, 35571, 35579, 3558, 3559, 3560, 3561, 3562, 3563, 3564, 3568, 3569, 3570, 3571, 3572, 3573, 3574, 3576, 3577, 3578, 35781, 35782, 35789, 3579, 3580, 35800, 35801, 3581, 3582, 35830, 35831, 35839, 3588, 3589, 3590, 3591, 3592, 35921, 35922, 35923, 35924, 35929, 3593, 3594, 3595, 3596, 35971, 35979, 3598, 35981, 35989, 3599, 7810, 7811, 7812, 7813, 7817, 7818, 7820, 7843, 7845, 78451, 78452, 78459, 78460, 78461, 78469, 7920, 7930, 79400, 79401, 79402, 79409, 79410, 79411, 79412, 79413, 79414, 79415, 79416, 79417, 79419, 7961, 79951, 79952, 79953, 79954, 79955, 79959, V124, V1240, V1241, V1242, V1249, V415, V452, V484, V485, V493, V530, V5301, V5302, V5309 |
| 96 | Heart valve disorders | 3940, 3941, 3942, 3949, 3950, 3951, 3952, 3959, 3960, 3961, 3962, 3963, 3968, 3969, 3970, 3971, 3979, 4240, 4241, 4242, 4243, 42490, 42491, 42499, 7852, 7853, V422, V433 |
| 97 | Peri-; endo-; and myocarditis; cardiomyopathy (except that caused by tuberculosis or sexually transmitted disease) | 03282, 03640, 03641, 03642, 03643, 07420, 07421, 07422, 07423, 11281, 11503, 11504, 11513, 11514, 11593, 11594, 1303, 3910, 3911, 3912, 3918, 3919, 3920, 393, 3980, 39890, 39899, 4200, 42090, 42091, 42099, 4210, 4211, 4219, 4220, 42290, 42291, 42292, 42293, 42299, 4230, 4231, 4232, 4233, 4238, 4239, 4250, 4251, 42511, 42518, 4252, 4253, 4254, 4257, 4258, 4259, 4290 |
| 98 | Essential hypertension | 4011, 4019 |
| 99 | Hypertension with complications and secondary hypertension | 4010, 40200, 40201, 40210, 40211, 40290, 40291, 4030, 40300, 40301, 4031, 40310, 40311, 4039, 40390, 40391, 4040, 40400, 40401, 40402, 40403, 4041, 40410, 40411, 40412, 40413, 4049, 40490, 40491, 40492, 40493, 40501, 40509, 40511, 40519, 40591, 40599, 4372 |
| 100 | Acute myocardial infarction | 4100, 41000, 41001, 41002, 4101, 41010, 41011, 41012, 4102, 41020, 41021, 41022, 4103, 41030, 41031, 41032, 4104, 41040, 41041, 41042, 4105, 41050, 41051, 41052, 4106, 41060, 41061, 41062, 4107, 41070, 41071, 41072, 4108, 41080, 41081, 41082, 4109, 41090, 41091, 41092 |
| 101 | Coronary atherosclerosis and other heart disease | 4110, 4111, 4118, 41181, 41189, 412, 4130, 4131, 4139, 4140, 41400, 41401, 41406, 4142, 4143, 4144, 4148, 4149, V4581, V4582 |
| 102 | Nonspecific chest pain | 78650, 78651, 78659 |
| 103 | Pulmonary heart disease | 4150, 4151, 41512, 41513, 41519, 4160, 4161, 4162, 4168, 4169, 4170, 4171, 4178, 4179, V1255 |
| 104 | Other and ill-defined heart disease | 41410, 41411, 41412, 41419, 4291, 4292, 4293, 4295, 4296, 42971, 42979, 42981, 42982, 42983, 42989, 4299 |
| 105 | Conduction disorders | 4260, 42610, 42611, 42612, 42613, 4262, 4263, 4264, 42650, 42651, 42652, 42653, 42654, 4266, 4267, 42681, 42682, 42689, 4269, V450, V4500, V4501, V4502, V4509, V533, V5331, V5332, V5339 |
| 106 | Cardiac dysrhythmias | 4270, 4271, 4272, 42731, 42732, 42760, 42761, 42769, 42781, 42789, 4279, 7850, 7851 |
| 107 | Cardiac arrest and ventricular fibrillation | 42741, 42742, 4275 |
| 108 | Congestive heart failure; nonhypertensive | 39891, 4280, 4281, 42820, 42821, 42822, 42823, 42830, 42831, 42832, 42833, 42840, 42841, 42842, 42843, 4289 |
| 109 | Acute cerebrovascular disease | 34660, 34661, 34662, 34663, 430, 431, 4320, 4321, 4329, 43301, 43311, 43321, 43331, 43381, 43391, 4340, 43400, 43401, 4341, 43410, 43411, 4349, 43490, 43491, 436 |
| 110 | Occlusion or stenosis of precerebral arteries | 4330, 43300, 4331, 43310, 4332, 43320, 4333, 43330, 4338, 43380, 4339, 43390 |
| 111 | Other and ill-defined cerebrovascular disease | 4370, 4371, 4373, 4374, 4375, 4376, 4377, 4378, 4379 |
| 112 | Transient cerebral ischemia | 4350, 4351, 4352, 4353, 4358, 4359 |
| 113 | Late effects of cerebrovascular disease | 438, 4380, 43810, 43811, 43812, 43813, 43814, 43819, 43820, 43821, 43822, 43830, 43831, 43832, 43840, 43841, 43842, 43850, 43851, 43852, 43853, 4386, 4387, 43881, 43882, 43883, 43884, 43885, 43889, 4389 |
| 114 | Peripheral and visceral atherosclerosis | 4400, 4401, 4402, 44020, 44021, 44022, 44023, 44029, 4404, 4408, 4409, 4439, 5570, 5571, 5579 |
| 115 | Aortic; peripheral; and visceral artery aneurysms | 4410, 44100, 44101, 44102, 44103, 4411, 4412, 4413, 4414, 4415, 4416, 4417, 4419, 4420, 4421, 4422, 4423, 44281, 44282, 44283, 44284, 44289, 4429, 44321, 44322, 44323, 44324, 44329, 44770, 44771, 44772, 44773 |
| 116 | Aortic and peripheral arterial embolism or thrombosis | 4440, 44401, 44409, 4441, 44421, 44422, 44481, 44489, 4449, 44501, 44502, 44581, 44589 |
| 117 | Other circulatory disease | 4430, 4431, 44381, 44382, 44389, 4460, 4461, 4462, 44620, 44621, 44629, 4463, 4464, 4465, 4466, 4467, 4470, 4471, 4472, 4473, 4474, 4475, 4476, 4478, 4479, 4480, 4481, 4489, 4580, 4581, 4588, 4589, 4590, 45989, 4599, 7859, 79430, 79431, 79439, 7962, V125, V1250, V1253, V1254, V1259, V151, V421, V432, V4321, V4322, V434, V717 |
| 118 | Phlebitis; thrombophlebitis and thromboembolism | 4510, 45111, 45119, 4512, 45181, 45182, 45183, 45184, 45189, 4519, 452, 4530, 4531, 4532, 4533, 45340, 45341, 45342, 45350, 45351, 45352, 4536, 45371, 45372, 45373, 45374, 45375, 45376, 45377, 45379, 4538, 45381, 45382, 45383, 45384, 45385, 45386, 45387, 45389, 4539, V1251, V1252 |
| 119 | Varicose veins of lower extremity | 4540, 4541, 4542, 4548, 4549 |
| 120 | Hemorrhoids | 4550, 4551, 4552, 4553, 4554, 4555, 4556, 4557, 4558, 4559 |
| 121 | Other diseases of veins and lymphatics | 4563, 4564, 4565, 4566, 4568, 4570, 4571, 4572, 4578, 4579, 4591, 45910, 45911, 45912, 45913, 45919, 4592, 45930, 45931, 45932, 45933, 45939, 45981 |
| 122 | Pneumonia (except that caused by tuberculosis or sexually transmitted disease) | 00322, 0203, 0204, 0205, 0212, 0221, 0310, 0391, 0521, 0551, 0730, 0830, 1124, 1140, 1144, 1145, 11505, 11515, 11595, 1304, 1363, 4800, 4801, 4802, 4803, 4808, 4809, 481, 4820, 4821, 4822, 4823, 48230, 48231, 48232, 48239, 4824, 48240, 48241, 48242, 48249, 4828, 48281, 48282, 48283, 48284, 48289, 4829, 483, 4830, 4831, 4838, 4841, 4843, 4845, 4846, 4847, 4848, 485, 486, 5130, 5171 |
| 123 | Influenza | 4870, 4871, 4878, 488, 4880, 48801, 48802, 48809, 4881, 48811, 48812, 48819, 48881, 48882, 48889 |
| 124 | Acute and chronic tonsillitis | 463, 4740, 47400, 47401, 47402, 47410, 47411, 47412, 4742, 4748, 4749, 475 |
| 125 | Acute bronchitis | 4660, 4661, 46611, 46619 |
| 126 | Other upper respiratory infections | 0320, 0321, 0322, 0323, 0340, 460, 4610, 4611, 4612, 4613, 4618, 4619, 462, 4640, 46400, 46401, 46410, 46411, 46420, 46421, 46430, 46431, 4644, 46450, 46451, 4650, 4658, 4659, 4730, 4731, 4732, 4733, 4738, 4739, 78491 |
| 127 | Chronic obstructive pulmonary disease and bronchiectasis | 490, 4910, 4911, 4912, 49120, 49121, 49122, 4918, 4919, 4920, 4928, 494, 4940, 4941, 496 |
| 128 | Asthma | 49300, 49301, 49302, 49310, 49311, 49312, 49320, 49321, 49322, 49381, 49382, 49390, 49391, 49392 |
| 129 | Aspiration pneumonitis; food/vomitus | 5070 |
| 130 | Pleurisy; pneumothorax; pulmonary collapse | 5100, 5109, 5110, 5111, 5118, 51189, 5119, 5120, 5128, 51281, 51282, 51283, 51284, 51289, 5180, 5181, 5182 |
| 131 | Respiratory failure; insufficiency; arrest (adult) | 5173, 5185, 51851, 51852, 51853, 51881, 51882, 51883, 51884, 7991, V461, V4611, V4612, V4613, V4614, V462 |
| 132 | Lung disease due to external agents | 4950, 4951, 4952, 4953, 4954, 4955, 4956, 4957, 4958, 4959, 500, 501, 502, 503, 504, 505, 5060, 5061, 5062, 5063, 5064, 5069, 5071, 5078, 5080, 5081, 5082, 5088, 5089 |
| 133 | Other lower respiratory disease | 5131, 514, 515, 5160, 5161, 5162, 5163, 51630, 51631, 51632, 51633, 51634, 51635, 51636, 51637, 5164, 5165, 51661, 51662, 51663, 51664, 51669, 5168, 5169, 5172, 5178, 5183, 5184, 51889, 5194, 5198, 5199, 7825, 78600, 78601, 78602, 78603, 78604, 78605, 78606, 78607, 78609, 7862, 7863, 78630, 78631, 78639, 7864, 78652, 7866, 7867, 7868, 7869, 7931, 79311, 79319, 7942, V126, V1260, V1261, V1269, V426 |
| 134 | Other upper respiratory disease | 470, 4710, 4711, 4718, 4719, 4720, 4721, 4722, 4760, 4761, 4770, 4772, 4778, 4779, 4780, 4781, 47811, 47819, 47820, 47821, 47822, 47824, 47825, 47826, 47829, 47830, 47831, 47832, 47833, 47834, 4784, 4785, 4786, 47870, 47871, 47874, 47875, 47879, 4788, 4789, 5191, 51911, 51919, 5192, 5193, 7841, 78440, 78441, 78442, 78443, 78444, 78449, 7847, 7848, 7849, 78499, 7861, V414, V440, V550 |
| 135 | Intestinal infection | 0010, 0011, 0019, 0020, 0021, 0022, 0023, 0029, 0030, 00320, 00329, 0038, 0039, 0040, 0041, 0042, 0043, 0048, 0049, 0050, 0051, 0052, 0053, 0054, 0058, 00581, 00589, 0059, 0060, 0061, 0062, 0063, 0064, 0065, 0066, 0068, 0069, 0070, 0071, 0072, 0073, 0074, 0075, 0078, 0079, 0080, 00800, 00801, 00802, 00803, 00804, 00809, 0081, 0082, 0083, 00841, 00842, 00843, 00844, 00845, 00846, 00847, 00849, 0085, 0086, 00861, 00862, 00863, 00864, 00865, 00866, 00867, 00869, 0088, 0090, 0091, 0092, 0093, 0211, 0222 |
| 136 | Disorders of teeth and jaw | 5200, 5201, 5202, 5203, 5204, 5205, 5206, 5207, 5208, 5209, 5210, 52100, 52101, 52102, 52103, 52104, 52105, 52106, 52107, 52108, 52109, 5211, 52110, 52111, 52112, 52113, 52114, 52115, 5212, 52120, 52121, 52122, 52123, 52124, 52125, 5213, 52130, 52131, 52132, 52133, 52134, 52135, 5214, 52140, 52141, 52142, 52149, 5215, 5216, 5217, 5218, 52181, 52189, 5219, 5220, 5221, 5222, 5223, 5224, 5225, 5226, 5227, 5228, 5229, 5230, 52300, 52301, 5231, 52310, 52311, 5232, 52320, 52321, 52322, 52323, 52324, 52325, 5233, 52330, 52331, 52332, 52333, 5234, 52340, 52341, 52342, 5235, 5236, 5238, 5239, 5240, 52400, 52401, 52402, 52403, 52404, 52405, 52406, 52407, 52409, 5241, 52410, 52411, 52412, 52419, 5242, 52420, 52421, 52422, 52423, 52424, 52425, 52426, 52427, 52428, 52429, 5243, 52430, 52431, 52432, 52433, 52434, 52435, 52436, 52437, 52439, 5244, 5245, 52450, 52451, 52452, 52453, 52454, 52455, 52456, 52457, 52459, 5246, 52460, 52461, 52462, 52463, 52464, 52469, 52470, 52471, 52472, 52473, 52474, 52475, 52476, 52479, 5248, 52481, 52482, 52489, 5249, 5250, 5251, 52510, 52511, 52512, 52513, 52519, 5252, 52520, 52521, 52522, 52523, 52524, 52525, 52526, 5253, 52540, 52541, 52542, 52543, 52544, 52550, 52551, 52552, 52553, 52554, 52560, 52561, 52562, 52563, 52564, 52565, 52566, 52567, 52569, 52571, 52572, 52573, 52579, 5258, 5259, 5260, 5261, 5262, 5263, 5264, 5265, 52661, 52662, 52663, 52669, 52681, 52689, 5269, 78492, V523, V534, V585, V722 |
| 137 | Diseases of mouth; excluding dental | 5270, 5271, 5272, 5273, 5274, 5275, 5276, 5277, 5278, 5279, 5280, 52800, 52809, 5281, 5282, 5283, 5284, 5285, 5286, 5287, 52871, 52872, 52879, 5288, 5289, 5290, 5291, 5292, 5293, 5294, 5295, 5296, 5298, 5299, 7924 |
| 138 | Esophageal disorders | 4561, 45621, 5300, 5301, 53010, 53011, 53012, 53013, 53019, 5302, 53020, 53021, 5303, 5304, 5305, 5306, 5308, 53081, 53083, 53084, 53085, 53089, 5309 |
| 139 | Gastroduodenal ulcer (except hemorrhage) | 53110, 53111, 53130, 53131, 53150, 53151, 53170, 53171, 53190, 53191, 53210, 53211, 53230, 53231, 53250, 53251, 53270, 53271, 53290, 53291, 53310, 53311, 53330, 53331, 53350, 53351, 53370, 53371, 53390, 53391, 53410, 53411, 53430, 53431, 53450, 53451, 53470, 53471, 53490, 53491, V1271 |
| 140 | Gastritis and duodenitis | 5350, 53500, 53501, 5351, 53510, 53511, 5352, 53520, 53521, 5354, 53540, 53541, 5355, 53550, 53551, 5356, 53560, 53561, 53570, 53571 |
| 141 | Other disorders of stomach and duodenum | 5360, 5361, 5362, 5363, 5368, 5369, 5370, 5371, 5372, 5373, 5374, 5375, 5376, 53781, 53782, 53783, 53784, 53789, 5379 |
| 142 | Appendicitis and other appendiceal conditions | 5400, 5401, 5409, 541, 542, 5430, 5439 |
| 143 | Abdominal hernia | 55000, 55001, 55002, 55003, 55010, 55011, 55012, 55013, 55090, 55091, 55092, 55093, 55100, 55101, 55102, 55103, 5511, 55120, 55121, 55129, 5513, 5518, 5519, 55200, 55201, 55202, 55203, 5521, 55220, 55221, 55229, 5523, 5528, 5529, 55300, 55301, 55302, 55303, 5531, 55320, 55321, 55329, 5533, 5538, 5539 |
| 144 | Regional enteritis and ulcerative colitis | 5550, 5551, 5552, 5559, 556, 5560, 5561, 5562, 5563, 5564, 5565, 5566, 5568, 5569 |
| 145 | Intestinal obstruction without hernia | 5600, 5601, 5602, 56030, 56031, 56032, 56039, 56081, 56089, 5609 |
| 146 | Diverticulosis and diverticulitis | 56200, 56201, 56202, 56203, 56210, 56211, 56212, 56213 |
| 147 | Anal and rectal conditions | 5646, 5650, 5651, 566, 5690, 5691, 5692, 56941, 56942, 56943, 56944, 56949 |
| 148 | Peritonitis and intestinal abscess | 03283, 5670, 5671, 5672, 56721, 56722, 56723, 56729, 56738, 56739, 5678, 56781, 56782, 56789, 5679, 5695 |
| 149 | Biliary tract disease | 57400, 57401, 57410, 57411, 57420, 57421, 57430, 57431, 57440, 57441, 57450, 57451, 57460, 57461, 57470, 57471, 57480, 57481, 57490, 57491, 5750, 5751, 57510, 57511, 57512, 5752, 5753, 5754, 5755, 5756, 5758, 5759, 5760, 5761, 5762, 5763, 5764, 5765, 5768, 5769, 7933 |
| 151 | Other liver diseases | 570, 5715, 5716, 5718, 5719, 5720, 5721, 5722, 5723, 5724, 5728, 5730, 5734, 5735, 5738, 5739, 7824, 7891, 7895, 78959, 7904, 7905, 7948, V427 |
| 152 | Pancreatic disorders (not diabetes) | 5770, 5771, 5772, 5778, 5779, 5794 |
| 153 | Gastrointestinal hemorrhage | 4560, 45620, 5307, 53082, 53100, 53101, 53120, 53121, 53140, 53141, 53160, 53161, 53200, 53201, 53220, 53221, 53240, 53241, 53260, 53261, 53300, 53301, 53320, 53321, 53340, 53341, 53360, 53361, 53400, 53401, 53420, 53421, 53440, 53441, 53460, 53461, 5693, 5780, 5781, 5789 |
| 154 | Noninfectious gastroenteritis | 55841, 55842, 5589 |
| 155 | Other gastrointestinal disorders | 538, 5581, 5582, 5640, 56400, 56401, 56402, 56409, 5641, 5645, 5647, 5648, 56481, 56489, 5649, 5680, 56881, 56882, 56889, 5689, 56981, 56982, 56983, 56984, 56985, 56986, 56987, 56989, 5699, 5790, 5791, 5792, 5798, 5799, 7871, 7872, 78720, 78721, 78722, 78723, 78724, 78729, 7873, 7874, 7875, 7876, 78760, 78761, 78762, 78763, 7877, 7879, 78791, 78799, 7892, 7893, 78930, 78931, 78932, 78933, 78934, 78935, 78936, 78937, 78939, 7894, 78940, 78941, 78942, 78943, 78944, 78945, 78946, 78947, 78949, 7899, 7921, 7934, 7936, V127, V1270, V1279, V416, V441, V442, V443, V444, V453, V473, V535, V5350, V5351, V5359, V551, V552, V553, V554 |
| 156 | Nephritis; nephrosis; renal sclerosis | 5800, 5804, 58081, 58089, 5809, 5810, 5811, 5812, 5813, 58181, 58189, 5819, 5820, 5821, 5822, 5824, 58281, 58289, 5829, 5830, 5831, 5832, 5834, 5836, 5837, 58381, 58389, 5839, 587 |
| 157 | Acute and unspecified renal failure | 5845, 5846, 5847, 5848, 5849, 586 |
| 158 | Chronic kidney disease | 585, 5851, 5852, 5853, 5854, 5855, 5856, 5859, 7925, V420, V451, V4511, V4512, V560, V561, V562, V5631, V5632, V568 |
| 159 | Urinary tract infections | 03284, 59000, 59001, 59010, 59011, 5902, 5903, 59080, 59081, 5909, 5950, 5951, 5952, 5953, 5954, 59581, 59582, 59589, 5959, 5970, 59780, 59781, 59789, 59800, 59801, 5990 |
| 160 | Calculus of urinary tract | 5920, 5921, 5929, 5940, 5941, 5942, 5948, 5949, 7880, V1301 |
| 161 | Other diseases of kidney and ureters | 5880, 5881, 5888, 58881, 58889, 5889, 5890, 5891, 5899, 591, 5930, 5931, 5932, 5933, 5934, 5935, 5936, 5937, 59370, 59371, 59372, 59373, 59381, 59382, 59389, 5939 |
| 162 | Other diseases of bladder and urethra | 5960, 5961, 5962, 5963, 5964, 5965, 59651, 59652, 59653, 59654, 59655, 59659, 5966, 5967, 5968, 59689, 5969, 5981, 5982, 5988, 5989, 5991, 5992, 5993, 5994, 5995, 59981, 59982, 59983, 59984 |
| 163 | Genitourinary symptoms and ill-defined conditions | 5996, 59960, 59969, 5997, 59970, 59971, 59972, 5998, 59989, 5999, 7881, 7882, 78820, 78821, 78829, 7883, 78830, 78831, 78832, 78833, 78834, 78835, 78836, 78837, 78838, 78839, 7884, 78841, 78842, 78843, 7885, 7886, 78861, 78862, 78863, 78864, 78865, 78869, 7887, 7888, 7889, 78891, 78899, 7910, 7911, 7912, 7913, 7914, 7917, 7919, 7935, 7944, V130, V1300, V1302, V1303, V1309, V417, V435, V445, V4450, V4451, V4452, V4459, V446, V474, V475, V536, V555, V556 |
| 164 | Hyperplasia of prostate | 600, 6000, 60000, 60001, 6001, 60010, 60011, 6002, 60020, 60021, 6003, 6009, 60090, 60091 |
| 165 | Inflammatory conditions of male genital organs | 6010, 6011, 6012, 6013, 6014, 6018, 6019, 6031, 6040, 60490, 60491, 60499, 6071, 6072, 6080, 6084 |
| 166 | Other male genital disorders | 6020, 6021, 6022, 6023, 6028, 6029, 6030, 6038, 6039, 605, 6060, 6061, 6068, 6069, 6070, 6073, 60781, 60782, 60783, 60784, 60785, 60789, 6079, 6081, 6082, 60820, 60821, 60822, 60823, 60824, 6083, 60881, 60882, 60883, 60884, 60885, 60886, 60887, 60889, 6089, 7922 |
| 167 | Nonmalignant breast conditions | 6100, 6101, 6102, 6103, 6104, 6108, 6109, 6110, 6111, 6112, 6113, 6114, 6115, 6116, 61171, 61172, 61179, 6118, 61181, 61182, 61183, 61189, 6119, 6120, 6121, 7938, 79380, 79381, 79382, 79389 |
| 168 | Inflammatory diseases of female pelvic organs | 6140, 6141, 6142, 6143, 6144, 6145, 6146, 6147, 6148, 6149, 6150, 6151, 6159, 6160, 61610, 61611, 6162, 6163, 6164, 61650, 61651, 6168, 61681, 61689, 6169, 62571 |
| 169 | Endometriosis | 6170, 6171, 6172, 6173, 6174, 6175, 6176, 6178, 6179 |
| 170 | Prolapse of female genital organs | 6180, 61800, 61801, 61802, 61803, 61804, 61805, 61809, 6181, 6182, 6183, 6184, 6185, 6186, 6187, 6188, 61881, 61882, 61883, 61884, 61889, 6189 |
| 171 | Menstrual disorders | 6253, 6260, 6261, 6262, 6263, 6264, 6265, 6266, 6268, 6269 |
| 172 | Ovarian cyst | 6200, 6201, 6202 |
| 173 | Menopausal disorders | 25631, 25639, 6270, 6271, 6272, 6273, 6274, 6278, 6279, V074 |
| 174 | Female infertility | 6280, 6281, 6282, 6283, 6284, 6288, 6289 |
| 175 | Other female genital disorders | 6190, 6191, 6192, 6198, 6199, 6203, 6204, 6205, 6206, 6207, 6208, 6209, 6210, 6211, 6212, 6213, 62130, 62131, 62132, 62133, 62134, 62135, 6214, 6215, 6216, 6217, 6218, 6219, 6220, 6221, 62210, 62211, 62212, 6222, 6223, 6224, 6225, 6226, 6227, 6228, 6229, 6230, 6231, 6232, 6233, 6234, 6235, 6236, 6237, 6238, 6239, 6240, 62401, 62402, 62409, 6241, 6242, 6243, 6244, 6245, 6246, 6248, 6249, 6250, 6251, 6252, 6254, 6255, 6256, 62570, 62579, 6258, 6259, 6267, 6290, 6291, 62920, 62921, 62922, 62923, 62929, 6298, 62981, 62989, 6299, 79509, V132, V1321, V1322, V1323, V1324, V1329, V557, V723 |
| 176 | Contraceptive and procreative management | V157, V2501, V2502, V2503, V2504, V2509, V251, V2511, V2512, V2513, V252, V253, V2540, V2541, V2542, V2543, V2549, V255, V258, V259, V260, V261, V262, V2621, V2622, V2629, V263, V2631, V2632, V2633, V2634, V2635, V2639, V264, V2641, V2642, V2649, V2651, V2652, V268, V2681, V2682, V2689, V269, V455, V4551, V4552, V4559 |
| 177 | Spontaneous abortion | 63400, 63401, 63402, 63410, 63411, 63412, 63420, 63421, 63422, 63430, 63431, 63432, 63440, 63441, 63442, 63450, 63451, 63452, 63460, 63461, 63462, 63470, 63471, 63472, 63480, 63481, 63482, 63490, 63491, 63492 |
| 178 | Induced abortion | 63500, 63501, 63502, 63510, 63511, 63512, 63520, 63521, 63522, 63530, 63531, 63532, 63540, 63541, 63542, 63550, 63551, 63552, 63560, 63561, 63562, 63570, 63571, 63572, 63580, 63581, 63582, 63590, 63591, 63592, 63600, 63601, 63602, 63610, 63611, 63612, 63620, 63621, 63622, 63630, 63631, 63632, 63640, 63641, 63642, 63650, 63651, 63652, 63660, 63661, 63662, 63670, 63671, 63672, 63680, 63681, 63682, 63690, 63691, 63692, 63700, 63701, 63702, 63710, 63711, 63712, 63720, 63721, 63722, 63730, 63731, 63732, 63740, 63741, 63742, 63750, 63751, 63752, 63760, 63761, 63762, 63770, 63771, 63772, 63780, 63781, 63782, 63790, 63791, 63792, 6380, 6381, 6382, 6383, 6384, 6385, 6386, 6387, 6388, 6389 |
| 179 | Postabortion complications | 6390, 6391, 6392, 6393, 6394, 6395, 6396, 6398, 6399 |
| 180 | Ectopic pregnancy | 6330, 63300, 63301, 6331, 63310, 63311, 6332, 63320, 63321, 6338, 63380, 63381, 6339, 63390, 63391 |
| 181 | Other complications of pregnancy | 630, 631, 6310, 6318, 632, 64300, 64301, 64303, 64310, 64311, 64313, 64320, 64321, 64323, 64380, 64381, 64383, 64390, 64391, 64393, 64600, 64601, 64603, 64610, 64611, 64612, 64613, 64614, 64620, 64621, 64622, 64623, 64624, 64630, 64631, 64633, 64640, 64641, 64642, 64643, 64644, 64650, 64651, 64652, 64653, 64654, 64660, 64661, 64662, 64663, 64664, 64670, 64671, 64673, 64680, 64681, 64682, 64683, 64684, 64690, 64691, 64693, 64700, 64701, 64702, 64703, 64704, 64710, 64711, 64712, 64713, 64714, 64720, 64721, 64722, 64723, 64724, 64730, 64731, 64732, 64733, 64734, 64740, 64741, 64742, 64743, 64744, 64750, 64751, 64752, 64753, 64754, 64760, 64761, 64762, 64763, 64764, 64780, 64781, 64782, 64783, 64784, 64790, 64791, 64792, 64793, 64794, 64810, 64811, 64812, 64813, 64814, 64820, 64821, 64822, 64823, 64824, 64850, 64851, 64852, 64853, 64854, 64860, 64861, 64862, 64863, 64864, 64870, 64871, 64872, 64873, 64874, 64890, 64891, 64892, 64893, 64894, 64900, 64901, 64902, 64903, 64904, 64910, 64911, 64912, 64913, 64914, 64920, 64921, 64922, 64923, 64924, 64930, 64931, 64932, 64933, 64934, 64940, 64941, 64942, 64943, 64944, 64950, 64951, 64953, 64960, 64961, 64962, 64963, 64964, V2342, V2387, V271, V272, V273, V274, V275, V276, V277, V279 |
| 182 | Hemorrhage during pregnancy; abruptio placenta; placenta previa | 64000, 64001, 64003, 64080, 64081, 64083, 64090, 64091, 64093, 64100, 64101, 64103, 64110, 64111, 64113, 64120, 64121, 64123, 64130, 64131, 64133, 64180, 64181, 64183, 64190, 64191, 64193 |
| 183 | Hypertension complicating pregnancy; childbirth and the puerperium | 64200, 64201, 64202, 64203, 64204, 64210, 64211, 64212, 64213, 64214, 64220, 64221, 64222, 64223, 64224, 64230, 64231, 64232, 64233, 64234, 64240, 64241, 64242, 64243, 64244, 64250, 64251, 64252, 64253, 64254, 64260, 64261, 64262, 64263, 64264, 64270, 64271, 64272, 64273, 64274, 64290, 64291, 64292, 64293, 64294 |
| 184 | Early or threatened labor | 64400, 64403, 64410, 64413, 64420, 64421 |
| 185 | Prolonged pregnancy | 64500, 64501, 64503, 64510, 64511, 64513, 64520, 64521, 64523 |
| 186 | Diabetes or abnormal glucose tolerance complicating pregnancy; childbirth; or the puerperium | 64800, 64801, 64802, 64803, 64804, 64880, 64881, 64882, 64883, 64884 |
| 187 | Malposition; malpresentation | 65200, 65201, 65203, 65210, 65211, 65213, 65220, 65221, 65223, 65230, 65231, 65233, 65240, 65241, 65243, 65250, 65251, 65253, 65260, 65261, 65263, 65270, 65271, 65273, 65280, 65281, 65283, 65290, 65291, 65293, 66000, 66001, 66003 |
| 188 | Fetopelvic disproportion; obstruction | 65300, 65301, 65303, 65310, 65311, 65313, 65320, 65321, 65323, 65330, 65331, 65333, 65340, 65341, 65343, 65350, 65351, 65353, 65360, 65361, 65363, 65370, 65371, 65373, 65380, 65381, 65383, 65390, 65391, 65393, 66010, 66011, 66013, 66020, 66021, 66023, 66030, 66031, 66033, 66040, 66041, 66043, 66050, 66051, 66053, 66060, 66061, 66063, 66070, 66071, 66073, 66080, 66081, 66083, 66090, 66091, 66093, 67810, 67811, 67813 |
| 189 | Previous C-section | 65420, 65421, 65423 |
| 190 | Fetal distress and abnormal forces of labor | 65630, 65631, 65633, 66100, 66101, 66103, 66110, 66111, 66113, 66120, 66121, 66123, 66130, 66131, 66133, 66140, 66141, 66143, 66190, 66191, 66193, 66200, 66201, 66203, 66210, 66211, 66213, 66220, 66221, 66223, 66230, 66231, 66233 |
| 191 | Polyhydramnios and other problems of amniotic cavity | 65700, 65701, 65703, 65800, 65801, 65803, 65810, 65811, 65813, 65820, 65821, 65823, 65830, 65831, 65833, 65840, 65841, 65843, 65880, 65881, 65883, 65890, 65891, 65893, 7923 |
| 192 | Umbilical cord complication | 66300, 66301, 66303, 66310, 66311, 66313, 66320, 66321, 66323, 66330, 66331, 66333, 66340, 66341, 66343, 66350, 66351, 66353, 66360, 66361, 66363, 66380, 66381, 66383, 66390, 66391, 66393 |
| 193 | OB-related trauma to perineum and vulva | 66400, 66401, 66404, 66410, 66411, 66414, 66420, 66421, 66424, 66430, 66431, 66434, 66440, 66441, 66444, 66450, 66451, 66454, 66460, 66461, 66464, 66480, 66481, 66484, 66490, 66491, 66494 |
| 194 | Forceps delivery | 66950, 66951 |
| 195 | Other complications of birth; puerperium affecting management of mother | 64970, 64971, 64973, 64981, 64982, 65103, 65113, 65123, 65130, 65131, 65133, 65140, 65141, 65143, 65150, 65151, 65153, 65160, 65161, 65163, 65183, 65193, 65400, 65401, 65402, 65403, 65404, 65410, 65411, 65412, 65413, 65414, 65430, 65431, 65432, 65433, 65434, 65440, 65441, 65442, 65443, 65444, 65450, 65451, 65452, 65453, 65454, 65460, 65461, 65462, 65463, 65464, 65470, 65471, 65472, 65473, 65474, 65480, 65481, 65482, 65483, 65484, 65490, 65491, 65492, 65493, 65494, 65500, 65501, 65503, 65510, 65511, 65513, 65520, 65521, 65523, 65530, 65531, 65533, 65540, 65541, 65543, 65560, 65561, 65563, 65570, 65571, 65573, 65580, 65581, 65583, 65590, 65591, 65593, 65600, 65601, 65603, 65610, 65611, 65613, 65620, 65621, 65623, 65640, 65641, 65643, 65650, 65651, 65653, 65660, 65661, 65663, 65670, 65671, 65673, 65680, 65681, 65683, 65690, 65691, 65693, 65900, 65901, 65903, 65910, 65911, 65913, 65920, 65921, 65923, 65930, 65931, 65933, 65940, 65941, 65943, 65950, 65951, 65953, 65960, 65961, 65963, 65970, 65971, 65973, 65980, 65981, 65983, 65990, 65991, 65993, 66500, 66501, 66503, 66510, 66511, 66512, 66514, 66520, 66522, 66524, 66530, 66531, 66534, 66540, 66541, 66544, 66550, 66551, 66554, 66560, 66561, 66564, 66570, 66571, 66572, 66574, 66580, 66581, 66582, 66583, 66584, 66590, 66591, 66592, 66593, 66594, 66600, 66602, 66604, 66610, 66612, 66614, 66620, 66622, 66624, 66630, 66632, 66634, 66700, 66702, 66704, 66710, 66712, 66714, 66800, 66801, 66802, 66803, 66804, 66810, 66811, 66812, 66813, 66814, 66820, 66821, 66822, 66823, 66824, 66880, 66881, 66882, 66883, 66884, 66890, 66891, 66892, 66893, 66894, 66900, 66901, 66902, 66903, 66904, 66910, 66911, 66912, 66913, 66914, 66920, 66921, 66922, 66923, 66924, 66930, 66932, 66934, 66940, 66941, 66942, 66943, 66944, 66960, 66961, 66970, 66971, 66980, 66981, 66982, 66983, 66984, 66990, 66991, 66992, 66993, 66994, 67000, 67002, 67004, 67010, 67012, 67014, 67020, 67022, 67024, 67030, 67032, 67034, 67080, 67082, 67084, 67100, 67101, 67102, 67103, 67104, 67110, 67111, 67112, 67113, 67114, 67120, 67121, 67122, 67123, 67124, 67130, 67131, 67133, 67140, 67142, 67144, 67150, 67151, 67152, 67153, 67154, 67180, 67181, 67182, 67183, 67184, 67190, 67191, 67192, 67193, 67194, 67200, 67202, 67204, 67300, 67301, 67302, 67303, 67304, 67310, 67311, 67312, 67313, 67314, 67320, 67321, 67322, 67323, 67324, 67330, 67331, 67332, 67333, 67334, 67380, 67381, 67382, 67383, 67384, 67400, 67401, 67402, 67403, 67404, 67410, 67412, 67414, 67420, 67422, 67424, 67430, 67432, 67434, 67440, 67442, 67444, 67450, 67451, 67452, 67453, 67454, 67480, 67482, 67484, 67490, 67492, 67494, 67500, 67501, 67502, 67503, 67504, 67510, 67511, 67512, 67513, 67514, 67520, 67521, 67522, 67523, 67524, 67580, 67581, 67582, 67583, 67584, 67590, 67591, 67592, 67593, 67594, 67600, 67601, 67602, 67603, 67604, 67610, 67611, 67612, 67613, 67614, 67620, 67621, 67622, 67623, 67624, 67630, 67631, 67632, 67633, 67634, 67640, 67641, 67642, 67643, 67644, 67650, 67651, 67652, 67653, 67654, 67660, 67661, 67662, 67663, 67664, 67680, 67681, 67682, 67683, 67684, 67690, 67691, 67692, 67693, 67694, 677, 67800, 67801, 67803, 67900, 67901, 67902, 67903, 67904, 67910, 67911, 67912, 67913, 67914, V230, V231, V232, V233, V234, V2341, V2349, V235, V237, V238, V2381, V2382, V2383, V2384, V2385, V2386, V2389, V239 |
| 196 | Other pregnancy and delivery including normal | 650, 65100, 65101, 65110, 65111, 65120, 65121, 65170, 65171, 65173, 65180, 65181, 65190, 65191, V220, V221, V222, V240, V241, V242, V270, V724, V7242, V9100, V9101, V9102, V9103, V9109, V9110, V9111, V9112, V9119, V9120, V9121, V9122, V9129, V9190, V9191, V9192, V9199 |
| 197 | Skin and subcutaneous tissue infections | 0201, 0210, 0220, 0311, 03285, 035, 0390, 6800, 6801, 6802, 6803, 6804, 6805, 6806, 6807, 6808, 6809, 68100, 68101, 68102, 68110, 68111, 6819, 6820, 6821, 6822, 6823, 6824, 6825, 6826, 6827, 6828, 6829, 684, 6850, 6851, 6860, 68600, 68601, 68609, 6861, 6868, 6869 |
| 198 | Other inflammatory condition of skin | 690, 69010, 69011, 69012, 69018, 6908, 69276, 69277, 6940, 6941, 6942, 6943, 6944, 6945, 69460, 69461, 6948, 6949, 6950, 6951, 69510, 69511, 69512, 69513, 69514, 69515, 69519, 6952, 6953, 6954, 69550, 69551, 69552, 69553, 69554, 69555, 69556, 69557, 69558, 69559, 69581, 69589, 6959, 6960, 6961, 6962, 6963, 6964, 6965, 6968, 6970, 6971, 6978, 6979, 6980, 6981, 6982, 6983, 6984, 6988, 6989 |
| 199 | Chronic ulcer of skin | 7070, 70700, 70701, 70702, 70703, 70704, 70705, 70706, 70707, 70709, 7071, 70710, 70711, 70712, 70713, 70714, 70715, 70719, 70720, 70721, 70722, 70723, 70724, 70725, 7078, 7079 |
| 200 | Other skin disorders | 69275, 700, 7010, 7011, 7012, 7013, 7014, 7015, 7018, 7019, 702, 7020, 7021, 70211, 70219, 7028, 7030, 7038, 7039, 70400, 70401, 70402, 70409, 7041, 7042, 7043, 70441, 70442, 7048, 7049, 7050, 7051, 70521, 70522, 70581, 70582, 70583, 70589, 7059, 7060, 7061, 7062, 7063, 7068, 7069, 7090, 70900, 70901, 70909, 7091, 7092, 7093, 7094, 7098, 7099, 7808, 7821, 7822, V133, V423 |
| 201 | Infective arthritis and osteomyelitis (except that caused by tuberculosis or sexually transmitted disease) | 00323, 00324, 0261, 03682, 05671, 71100, 71101, 71102, 71103, 71104, 71105, 71106, 71107, 71108, 71109, 71110, 71111, 71112, 71113, 71114, 71115, 71116, 71117, 71118, 71119, 71120, 71121, 71122, 71123, 71124, 71125, 71126, 71127, 71128, 71129, 71130, 71131, 71132, 71133, 71134, 71135, 71136, 71137, 71138, 71139, 71140, 71141, 71142, 71143, 71144, 71145, 71146, 71147, 71148, 71149, 71150, 71151, 71152, 71153, 71154, 71155, 71156, 71157, 71158, 71159, 71160, 71161, 71162, 71163, 71164, 71165, 71166, 71167, 71168, 71169, 71170, 71171, 71172, 71173, 71174, 71175, 71176, 71177, 71178, 71179, 71180, 71181, 71182, 71183, 71184, 71185, 71186, 71187, 71188, 71189, 71190, 71191, 71192, 71193, 71194, 71195, 71196, 71197, 71198, 71199, 73000, 73001, 73002, 73003, 73004, 73005, 73006, 73007, 73008, 73009, 73010, 73011, 73012, 73013, 73014, 73015, 73016, 73017, 73018, 73019, 73020, 73021, 73022, 73023, 73024, 73025, 73026, 73027, 73028, 73029, 73030, 73031, 73032, 73033, 73034, 73035, 73036, 73037, 73038, 73039, 73070, 73071, 73072, 73073, 73074, 73075, 73076, 73077, 73078, 73079, 73080, 73081, 73082, 73083, 73084, 73085, 73086, 73087, 73088, 73089, 73090, 73091, 73092, 73093, 73094, 73095, 73096, 73097, 73098, 73099 |
| 202 | Rheumatoid arthritis and related disease | 7140, 7141, 7142, 71430, 71431, 71432, 71433, 7144, 71481, 71489, 7149, 7200 |
| 203 | Osteoarthritis | 71500, 71504, 71509, 71510, 71511, 71512, 71513, 71514, 71515, 71516, 71517, 71518, 71520, 71521, 71522, 71523, 71524, 71525, 71526, 71527, 71528, 71530, 71531, 71532, 71533, 71534, 71535, 71536, 71537, 71538, 71580, 71589, 71590, 71591, 71592, 71593, 71594, 71595, 71596, 71597, 71598, V134 |
| 204 | Other non-traumatic joint disorders | 7130, 7131, 7132, 7133, 7134, 7135, 7136, 7137, 7138, 71600, 71601, 71602, 71603, 71604, 71605, 71606, 71607, 71608, 71609, 71620, 71621, 71622, 71623, 71624, 71625, 71626, 71627, 71628, 71629, 71630, 71631, 71632, 71633, 71634, 71635, 71636, 71637, 71638, 71639, 71640, 71641, 71642, 71643, 71644, 71645, 71646, 71647, 71648, 71649, 71650, 71651, 71652, 71653, 71654, 71655, 71656, 71657, 71658, 71659, 71660, 71661, 71662, 71663, 71664, 71665, 71666, 71667, 71668, 71680, 71681, 71682, 71683, 71684, 71685, 71686, 71687, 71688, 71689, 71690, 71691, 71692, 71693, 71694, 71695, 71696, 71697, 71698, 71699, 71810, 71811, 71812, 71813, 71814, 71815, 71817, 71818, 71819, 71820, 71821, 71822, 71823, 71824, 71825, 71826, 71827, 71828, 71829, 71850, 71851, 71852, 71853, 71854, 71855, 71856, 71857, 71858, 71859, 71860, 71865, 71870, 71871, 71872, 71873, 71874, 71875, 71876, 71877, 71878, 71879, 71880, 71881, 71882, 71883, 71884, 71885, 71886, 71887, 71888, 71889, 71890, 71891, 71892, 71893, 71894, 71895, 71897, 71898, 71899, 71900, 71901, 71902, 71903, 71904, 71905, 71906, 71907, 71908, 71909, 71910, 71911, 71912, 71913, 71914, 71915, 71916, 71917, 71918, 71919, 71920, 71921, 71922, 71923, 71924, 71925, 71926, 71927, 71928, 71929, 71930, 71931, 71932, 71933, 71934, 71935, 71936, 71937, 71938, 71939, 71940, 71941, 71942, 71943, 71944, 71945, 71946, 71947, 71948, 71949, 71950, 71951, 71952, 71953, 71954, 71955, 71956, 71957, 71958, 71959, 71960, 71961, 71962, 71963, 71964, 71965, 71966, 71967, 71968, 71969, 7197, 71970, 71975, 71976, 71977, 71978, 71979, 71980, 71981, 71982, 71983, 71984, 71985, 71986, 71987, 71988, 71989, 71990, 71991, 71992, 71993, 71994, 71995, 71996, 71997, 71998, 71999 |
| 205 | Spondylosis; intervertebral disc disorders; other back problems | 7201, 7202, 72081, 72089, 7209, 7210, 7211, 7212, 7213, 72141, 72142, 7215, 7216, 7217, 7218, 72190, 72191, 7220, 72210, 72211, 7222, 72230, 72231, 72232, 72239, 7224, 72251, 72252, 7226, 72270, 72271, 72272, 72273, 72280, 72281, 72282, 72283, 72290, 72291, 72292, 72293, 7230, 7231, 7232, 7233, 7234, 7235, 7236, 7237, 7238, 7239, 72400, 72401, 72402, 72403, 72409, 7241, 7242, 7243, 7244, 7245, 7246, 72470, 72471, 72479, 7248, 7249 |
| 206 | Osteoporosis | 73300, 73301, 73302, 73303, 73309 |
| 207 | Pathological fracture | 7331, 73310, 73311, 73312, 73313, 73314, 73315, 73316, 73319, 73393, 73394, 73395, 73396, 73397, 73398, V1351 |
| 208 | Acquired foot deformities | 7271, 734, 7350, 7351, 7352, 7353, 7354, 7355, 7358, 7359, 73670, 73671, 73672, 73673, 73674, 73675, 73676, 73679 |
| 209 | Other acquired deformities | 71840, 71841, 71842, 71843, 71844, 71845, 71846, 71847, 71848, 71849, 73600, 73601, 73602, 73603, 73604, 73605, 73606, 73607, 73609, 7361, 73620, 73621, 73622, 73629, 73630, 73631, 73632, 73639, 73641, 73642, 7365, 7366, 73681, 73689, 7369, 7370, 73710, 73711, 73712, 73719, 73720, 73721, 73722, 73729, 73733, 73734, 73739, 73740, 73741, 73742, 73743, 7378, 7379, 7380, 7381, 73810, 73811, 73812, 73819, 7382, 7383, 7384, 7385, 7386, 7387, 7388, 7389 |
| 210 | Systemic lupus erythematosus and connective tissue disorders | 7100, 7101, 7102, 7103, 7104, 7108, 7109 |
| 211 | Other connective tissue disease | 32752, 56731, 7105, 725, 7260, 72610, 72611, 72612, 72613, 72619, 7262, 72630, 72631, 72632, 72633, 72639, 7264, 7265, 72660, 72661, 72662, 72663, 72664, 72665, 72669, 72670, 72671, 72672, 72673, 72679, 7268, 72690, 72691, 72700, 72701, 72702, 72703, 72704, 72705, 72706, 72709, 7272, 7273, 72740, 72741, 72742, 72743, 72749, 72750, 72751, 72759, 72760, 72761, 72762, 72763, 72764, 72765, 72766, 72767, 72768, 72769, 72781, 72782, 72783, 72789, 7279, 7280, 72810, 72811, 72812, 72813, 72819, 7282, 7283, 7284, 7285, 7286, 72871, 72879, 72881, 72882, 72883, 72884, 72885, 72886, 72887, 72888, 72889, 7289, 7290, 7291, 7292, 72930, 72931, 72939, 7294, 7295, 7296, 72971, 72972, 72973, 72979, 72981, 72982, 72989, 7299, 72990, 72991, 72992, 72999, 7819, 78191, 78192, 78194, 78199, 7937, V135, V1359, V436, V4360, V4361, V4362, V4363, V4364, V4365, V4366, V4369, V437, V454, V481, V482, V483, V490, V491, V492, V495, V4960, V4961, V4962, V4963, V4964, V4965, V4966, V4967, V4970, V4971, V4972, V4973, V4974, V4975, V4976, V4977, V537 |
| 212 | Other bone disease and musculoskeletal deformities | 7310, 7311, 7312, 7313, 7318, 7320, 7321, 7322, 7323, 7324, 7325, 7326, 7327, 7328, 7329, 73320, 73321, 73322, 73329, 7333, 73340, 73341, 73342, 73343, 73344, 73345, 73349, 7335, 7336, 7337, 73381, 73382, 73390, 73391, 73392, 73399, 73730, 73731, 73732, 7390, 7391, 7392, 7393, 7394, 7395, 7396, 7397, 7398, 7399, V424, V486, V487, V494, V8821, V8822, V8829 |
| 213 | Cardiac and circulatory congenital anomalies | 7450, 74510, 74511, 74512, 74519, 7452, 7453, 7454, 7455, 74560, 74561, 74569, 7457, 7458, 7459, 74600, 74601, 74602, 74609, 7461, 7462, 7463, 7464, 7465, 7466, 7467, 74681, 74682, 74683, 74684, 74685, 74686, 74687, 74689, 7469, 7470, 74710, 74711, 74720, 74721, 74722, 74729, 7473, 74731, 74732, 74739, 74740, 74741, 74742, 74749, 7475, 7476, 74760, 74761, 74762, 74763, 74764, 74769, 74781, 74782, 74783, 74789, 7479, V1365 |
| 214 | Digestive congenital anomalies | 7500, 75010, 75011, 75012, 75013, 75015, 75016, 75019, 75021, 75022, 75023, 75024, 75025, 75026, 75027, 75029, 7503, 7504, 7505, 7506, 7507, 7508, 7509, 7510, 7511, 7512, 7513, 7514, 7515, 75160, 75161, 75162, 75169, 7517, 7518, 7519, V1367 |
| 215 | Genitourinary congenital anomalies | 7520, 75210, 75211, 75219, 7522, 7523, 75231, 75232, 75233, 75234, 75235, 75236, 75239, 75240, 75241, 75242, 75243, 75244, 75245, 75246, 75247, 75249, 7525, 75251, 75252, 7526, 75261, 75262, 75263, 75264, 75265, 75269, 7527, 7528, 75281, 75289, 7529, 7530, 7531, 75310, 75311, 75312, 75313, 75314, 75315, 75316, 75317, 75319, 7532, 75320, 75321, 75322, 75323, 75329, 7533, 7534, 7535, 7536, 7537, 7538, 7539, V1361, V1362 |
| 216 | Nervous system congenital anomalies | 7400, 7401, 7402, 74100, 74101, 74102, 74103, 74190, 74191, 74192, 74193, 7420, 7421, 7422, 7423, 7424, 74251, 74253, 74259, 7428, 7429, V1363 |
| 217 | Other congenital anomalies | 74300, 74303, 74306, 74310, 74311, 74312, 74320, 74321, 74322, 74330, 74331, 74332, 74333, 74334, 74335, 74336, 74337, 74339, 74341, 74342, 74343, 74344, 74345, 74346, 74347, 74348, 74349, 74351, 74352, 74353, 74354, 74355, 74356, 74357, 74358, 74359, 74361, 74362, 74363, 74364, 74365, 74366, 74369, 7438, 7439, 74400, 74401, 74402, 74403, 74404, 74405, 74409, 7441, 74421, 74422, 74423, 74424, 74429, 7443, 74441, 74442, 74443, 74446, 74447, 74449, 7445, 74481, 74482, 74483, 74484, 74489, 7449, 7480, 7481, 7482, 7483, 7484, 7485, 74860, 74861, 74869, 7488, 7489, 74900, 74901, 74902, 74903, 74904, 74910, 74911, 74912, 74913, 74914, 74920, 74921, 74922, 74923, 74924, 74925, 7540, 7541, 7542, 75430, 75431, 75432, 75433, 75435, 75440, 75441, 75442, 75443, 75444, 75450, 75451, 75452, 75453, 75459, 75460, 75461, 75462, 75469, 75470, 75471, 75479, 75481, 75482, 75489, 75500, 75501, 75502, 75510, 75511, 75512, 75513, 75514, 75520, 75521, 75522, 75523, 75524, 75525, 75526, 75527, 75528, 75529, 75530, 75531, 75532, 75533, 75534, 75535, 75536, 75537, 75538, 75539, 7554, 75550, 75551, 75552, 75553, 75554, 75555, 75556, 75557, 75558, 75559, 75560, 75561, 75562, 75563, 75564, 75565, 75566, 75567, 75569, 7558, 7559, 7560, 75610, 75611, 75612, 75613, 75614, 75615, 75616, 75617, 75619, 7562, 7563, 7564, 75650, 75651, 75652, 75653, 75654, 75655, 75656, 75659, 7566, 7567, 75670, 75671, 75672, 75673, 75679, 75681, 75682, 75683, 75689, 7569, 7570, 7571, 7572, 75731, 75732, 75733, 75739, 7574, 7575, 7576, 7578, 7579, 7580, 7581, 7582, 7583, 75831, 75832, 75833, 75839, 7584, 7585, 7586, 7587, 7588, 75881, 75889, 7589, 7590, 7591, 7592, 7593, 7594, 7595, 7596, 7597, 7598, 75981, 75982, 75983, 75989, 7599, 7952, V136, V1364, V1366, V1368, V1369 |
| 218 | Liveborn | 76520, 76529, V300, V3000, V3001, V301, V302, V310, V3100, V3101, V311, V312, V320, V3200, V3201, V321, V322, V330, V3300, V3301, V331, V332, V340, V3400, V3401, V341, V342, V350, V3500, V3501, V351, V352, V360, V3600, V3601, V361, V362, V370, V3700, V3701, V371, V372, V390, V3900, V3901, V391, V392 |
| 219 | Short gestation; low birth weight; and fetal growth retardation | 7640, 76400, 76401, 76402, 76403, 76404, 76405, 76406, 76407, 76408, 76409, 7641, 76410, 76411, 76412, 76413, 76414, 76415, 76416, 76417, 76418, 76419, 7642, 76420, 76421, 76422, 76423, 76424, 76425, 76426, 76427, 76428, 76429, 7649, 76490, 76491, 76492, 76493, 76494, 76495, 76496, 76497, 76498, 76499, 7650, 76500, 76501, 76502, 76503, 76504, 76505, 76506, 76507, 76508, 76509, 7651, 76510, 76511, 76512, 76513, 76514, 76515, 76516, 76517, 76518, 76519, 76521, 76522, 76523, 76524, 76525, 76526, 76527, 76528, V2130, V2131, V2132, V2133, V2134, V2135 |
| 220 | Intrauterine hypoxia and birth asphyxia | 7680, 7681, 7682, 7683, 7684, 7685, 7686, 7687, 76870, 76871, 76872, 76873, 7689, 77088 |
| 221 | Respiratory distress syndrome | 769 |
| 222 | Hemolytic jaundice and perinatal jaundice | 7730, 7731, 7732, 7733, 7734, 7735, 7740, 7741, 7742, 77430, 77431, 77439, 7744, 7745, 7746, 7747 |
| 223 | Birth trauma | 7670, 7671, 76711, 76719, 7672, 7673, 7674, 7675, 7676, 7677, 7678, 7679 |
| 224 | Other perinatal conditions | 04041, 7600, 7601, 7602, 7603, 7604, 7605, 7606, 76061, 76062, 76063, 76064, 76070, 76074, 76076, 76077, 76078, 76079, 7608, 7609, 7610, 7611, 7612, 7613, 7614, 7615, 7616, 7617, 7618, 7619, 7620, 7621, 7622, 7623, 7624, 7625, 7626, 7627, 7628, 7629, 7630, 7631, 7632, 7633, 7634, 7635, 7636, 7637, 7638, 76381, 76382, 76383, 76384, 76389, 7639, 7660, 7661, 7662, 76621, 76622, 7700, 7701, 77010, 77011, 77012, 77013, 77014, 77015, 77016, 77017, 77018, 7702, 7703, 7704, 7705, 7706, 7707, 7708, 77081, 77082, 77083, 77084, 77085, 77086, 77087, 77089, 7709, 7710, 7711, 7712, 7713, 7714, 7715, 7716, 7717, 7718, 77182, 77183, 77189, 7720, 7721, 77210, 77211, 77212, 77213, 77214, 7722, 7723, 7724, 7725, 7726, 7728, 7729, 7750, 7751, 7752, 7753, 7754, 7755, 7756, 7757, 7758, 77581, 77589, 7759, 7760, 7761, 7762, 7763, 7764, 7765, 7766, 7767, 7768, 7769, 7771, 7772, 7773, 7774, 7775, 77750, 77751, 77752, 77753, 7776, 7778, 7779, 7780, 7781, 7782, 7783, 7784, 7785, 7786, 7787, 7788, 7789, 7790, 7791, 7792, 7793, 77931, 77932, 77933, 77934, 7794, 7796, 7797, 7798, 77981, 77982, 77983, 77984, 77985, 77989, 7799, 78091, 78092, 7897, V137, V502 |
| 225 | Joint disorders and dislocations; trauma-related | 71610, 71611, 71612, 71613, 71614, 71615, 71616, 71617, 71618, 71619, 7170, 7171, 7172, 7173, 71740, 71741, 71742, 71743, 71749, 7175, 7176, 7177, 71781, 71782, 71783, 71784, 71785, 71789, 7179, 71800, 71801, 71802, 71803, 71804, 71805, 71807, 71808, 71809, 71830, 71831, 71832, 71833, 71834, 71835, 71836, 71837, 71838, 71839, 8300, 8301, 83100, 83101, 83102, 83103, 83104, 83109, 83110, 83111, 83112, 83113, 83114, 83119, 83200, 83201, 83202, 83203, 83204, 83209, 83210, 83211, 83212, 83213, 83214, 83219, 8322, 83300, 83301, 83302, 83303, 83304, 83305, 83309, 83310, 83311, 83312, 83313, 83314, 83315, 83319, 83400, 83401, 83402, 83410, 83411, 83412, 83500, 83501, 83502, 83503, 83510, 83511, 83512, 83513, 8360, 8361, 8362, 8363, 8364, 83650, 83651, 83652, 83653, 83654, 83659, 83660, 83661, 83662, 83663, 83664, 83669, 8370, 8371, 83800, 83801, 83802, 83803, 83804, 83805, 83806, 83809, 83810, 83811, 83812, 83813, 83814, 83815, 83816, 83819, 83900, 83901, 83902, 83903, 83904, 83905, 83906, 83907, 83908, 83910, 83911, 83912, 83913, 83914, 83915, 83916, 83917, 83918, 83920, 83921, 83930, 83931, 83940, 83941, 83942, 83949, 83950, 83951, 83952, 83959, 83961, 83969, 83971, 83979, 8398, 8399, 9056 |
| 226 | Fracture of neck of femur (hip) | 82000, 82001, 82002, 82003, 82009, 82010, 82011, 82012, 82013, 82019, 82020, 82021, 82022, 82030, 82031, 82032, 8208, 8209, 9053, V5413, V5423 |
| 227 | Spinal cord injury | 34939, 80600, 80601, 80602, 80603, 80604, 80605, 80606, 80607, 80608, 80609, 80610, 80611, 80612, 80613, 80614, 80615, 80616, 80617, 80618, 80619, 80620, 80621, 80622, 80623, 80624, 80625, 80626, 80627, 80628, 80629, 80630, 80631, 80632, 80633, 80634, 80635, 80636, 80637, 80638, 80639, 8064, 8065, 80660, 80661, 80662, 80669, 80670, 80671, 80672, 80679, 8068, 8069, 9072, 95200, 95201, 95202, 95203, 95204, 95205, 95206, 95207, 95208, 95209, 95210, 95211, 95212, 95213, 95214, 95215, 95216, 95217, 95218, 95219, 9522, 9523, 9524, 9528, 9529 |
| 228 | Skull and face fractures | 80000, 80001, 80002, 80003, 80004, 80005, 80006, 80009, 80050, 80051, 80052, 80053, 80054, 80055, 80056, 80059, 80100, 80101, 80102, 80103, 80104, 80105, 80106, 80109, 80150, 80151, 80152, 80153, 80154, 80155, 80156, 80159, 8020, 8021, 80220, 80221, 80222, 80223, 80224, 80225, 80226, 80227, 80228, 80229, 80230, 80231, 80232, 80233, 80234, 80235, 80236, 80237, 80238, 80239, 8024, 8025, 8026, 8027, 8028, 8029, 80300, 80301, 80302, 80303, 80304, 80305, 80306, 80309, 80350, 80351, 80352, 80353, 80354, 80355, 80356, 80359, 80400, 80401, 80402, 80403, 80404, 80405, 80406, 80409, 80450, 80451, 80452, 80453, 80454, 80455, 80456, 80459, 9050 |
| 229 | Fracture of upper limb | 81000, 81001, 81002, 81003, 81010, 81011, 81012, 81013, 81100, 81101, 81102, 81103, 81109, 81110, 81111, 81112, 81113, 81119, 81200, 81201, 81202, 81203, 81209, 81210, 81211, 81212, 81213, 81219, 81220, 81221, 81230, 81231, 81240, 81241, 81242, 81243, 81244, 81249, 81250, 81251, 81252, 81253, 81254, 81259, 81300, 81301, 81302, 81303, 81304, 81305, 81306, 81307, 81308, 81310, 81311, 81312, 81313, 81314, 81315, 81316, 81317, 81318, 81320, 81321, 81322, 81323, 81330, 81331, 81332, 81333, 81340, 81341, 81342, 81343, 81344, 81345, 81346, 81347, 81350, 81351, 81352, 81353, 81354, 81380, 81381, 81382, 81383, 81390, 81391, 81392, 81393, 81400, 81401, 81402, 81403, 81404, 81405, 81406, 81407, 81408, 81409, 81410, 81411, 81412, 81413, 81414, 81415, 81416, 81417, 81418, 81419, 81500, 81501, 81502, 81503, 81504, 81509, 81510, 81511, 81512, 81513, 81514, 81519, 81600, 81601, 81602, 81603, 81610, 81611, 81612, 81613, 8170, 8171, 8180, 8181, 8190, 8191, 9052, V5410, V5411, V5412, V5420, V5421, V5422 |
| 230 | Fracture of lower limb | 82100, 82101, 82110, 82111, 82120, 82121, 82122, 82123, 82129, 82130, 82131, 82132, 82133, 82139, 8220, 8221, 82300, 82301, 82302, 82310, 82311, 82312, 82320, 82321, 82322, 82330, 82331, 82332, 82340, 82341, 82342, 82380, 82381, 82382, 82390, 82391, 82392, 8240, 8241, 8242, 8243, 8244, 8245, 8246, 8247, 8248, 8249, 8250, 8251, 82520, 82521, 82522, 82523, 82524, 82525, 82529, 82530, 82531, 82532, 82533, 82534, 82535, 82539, 8260, 8261, 8270, 8271, 9054, V5414, V5415, V5416, V5424, V5425, V5426 |
| 231 | Other fractures | 80500, 80501, 80502, 80503, 80504, 80505, 80506, 80507, 80508, 80510, 80511, 80512, 80513, 80514, 80515, 80516, 80517, 80518, 8052, 8053, 8054, 8055, 8056, 8057, 8058, 8059, 80700, 80701, 80702, 80703, 80704, 80705, 80706, 80707, 80708, 80709, 80710, 80711, 80712, 80713, 80714, 80715, 80716, 80717, 80718, 80719, 8072, 8073, 8074, 8075, 8076, 8080, 8081, 8082, 8083, 80841, 80842, 80843, 80844, 80849, 80851, 80852, 80853, 80854, 80859, 8088, 8089, 8090, 8091, 8280, 8281, 8290, 8291, 9051, 9055, V1352, V540, V5401, V5402, V5409, V5417, V5419, V5427, V5429, V664, V674 |
| 232 | Sprains and strains | 8400, 8401, 8402, 8403, 8404, 8405, 8406, 8407, 8408, 8409, 8410, 8411, 8412, 8413, 8418, 8419, 84200, 84201, 84202, 84209, 84210, 84211, 84212, 84213, 84219, 8430, 8431, 8438, 8439, 8440, 8441, 8442, 8443, 8448, 8449, 84500, 84501, 84502, 84503, 84509, 84510, 84511, 84512, 84513, 84519, 8460, 8461, 8462, 8463, 8468, 8469, 8470, 8471, 8472, 8473, 8474, 8479, 8480, 8481, 8482, 8483, 84840, 84841, 84842, 84849, 8485, 8488, 8489, 9057 |
| 233 | Intracranial injury | 80010, 80011, 80012, 80013, 80014, 80015, 80016, 80019, 80020, 80021, 80022, 80023, 80024, 80025, 80026, 80029, 80030, 80031, 80032, 80033, 80034, 80035, 80036, 80039, 80040, 80041, 80042, 80043, 80044, 80045, 80046, 80049, 80060, 80061, 80062, 80063, 80064, 80065, 80066, 80069, 80070, 80071, 80072, 80073, 80074, 80075, 80076, 80079, 80080, 80081, 80082, 80083, 80084, 80085, 80086, 80089, 80090, 80091, 80092, 80093, 80094, 80095, 80096, 80099, 80110, 80111, 80112, 80113, 80114, 80115, 80116, 80119, 80120, 80121, 80122, 80123, 80124, 80125, 80126, 80129, 80130, 80131, 80132, 80133, 80134, 80135, 80136, 80139, 80140, 80141, 80142, 80143, 80144, 80145, 80146, 80149, 80160, 80161, 80162, 80163, 80164, 80165, 80166, 80169, 80170, 80171, 80172, 80173, 80174, 80175, 80176, 80179, 80180, 80181, 80182, 80183, 80184, 80185, 80186, 80189, 80190, 80191, 80192, 80193, 80194, 80195, 80196, 80199, 80310, 80311, 80312, 80313, 80314, 80315, 80316, 80319, 80320, 80321, 80322, 80323, 80324, 80325, 80326, 80329, 80330, 80331, 80332, 80333, 80334, 80335, 80336, 80339, 80340, 80341, 80342, 80343, 80344, 80345, 80346, 80349, 80360, 80361, 80362, 80363, 80364, 80365, 80366, 80369, 80370, 80371, 80372, 80373, 80374, 80375, 80376, 80379, 80380, 80381, 80382, 80383, 80384, 80385, 80386, 80389, 80390, 80391, 80392, 80393, 80394, 80395, 80396, 80399, 80410, 80411, 80412, 80413, 80414, 80415, 80416, 80419, 80420, 80421, 80422, 80423, 80424, 80425, 80426, 80429, 80430, 80431, 80432, 80433, 80434, 80435, 80436, 80439, 80440, 80441, 80442, 80443, 80444, 80445, 80446, 80449, 80460, 80461, 80462, 80463, 80464, 80465, 80466, 80469, 80470, 80471, 80472, 80473, 80474, 80475, 80476, 80479, 80480, 80481, 80482, 80483, 80484, 80485, 80486, 80489, 80490, 80491, 80492, 80493, 80494, 80495, 80496, 80499, 8500, 8501, 85011, 85012, 8502, 8503, 8504, 8505, 8509, 85100, 85101, 85102, 85103, 85104, 85105, 85106, 85109, 85110, 85111, 85112, 85113, 85114, 85115, 85116, 85119, 85120, 85121, 85122, 85123, 85124, 85125, 85126, 85129, 85130, 85131, 85132, 85133, 85134, 85135, 85136, 85139, 85140, 85141, 85142, 85143, 85144, 85145, 85146, 85149, 85150, 85151, 85152, 85153, 85154, 85155, 85156, 85159, 85160, 85161, 85162, 85163, 85164, 85165, 85166, 85169, 85170, 85171, 85172, 85173, 85174, 85175, 85176, 85179, 85180, 85181, 85182, 85183, 85184, 85185, 85186, 85189, 85190, 85191, 85192, 85193, 85194, 85195, 85196, 85199, 85200, 85201, 85202, 85203, 85204, 85205, 85206, 85209, 85210, 85211, 85212, 85213, 85214, 85215, 85216, 85219, 85220, 85221, 85222, 85223, 85224, 85225, 85226, 85229, 85230, 85231, 85232, 85233, 85234, 85235, 85236, 85239, 85240, 85241, 85242, 85243, 85244, 85245, 85246, 85249, 85250, 85251, 85252, 85253, 85254, 85255, 85256, 85259, 85300, 85301, 85302, 85303, 85304, 85305, 85306, 85309, 85310, 85311, 85312, 85313, 85314, 85315, 85316, 85319, 85400, 85401, 85402, 85403, 85404, 85405, 85406, 85409, 85410, 85411, 85412, 85413, 85414, 85415, 85416, 85419, 9070, V1552 |
| 234 | Crushing injury or internal injury | 8600, 8601, 8602, 8603, 8604, 8605, 86100, 86101, 86102, 86103, 86110, 86111, 86112, 86113, 86120, 86121, 86122, 86130, 86131, 86132, 8620, 8621, 86221, 86222, 86229, 86231, 86232, 86239, 8628, 8629, 8630, 8631, 86320, 86321, 86329, 86330, 86331, 86339, 86340, 86341, 86342, 86343, 86344, 86345, 86346, 86349, 86350, 86351, 86352, 86353, 86354, 86355, 86356, 86359, 86380, 86381, 86382, 86383, 86384, 86385, 86389, 86390, 86391, 86392, 86393, 86394, 86395, 86399, 86400, 86401, 86402, 86403, 86404, 86405, 86409, 86410, 86411, 86412, 86413, 86414, 86415, 86419, 86500, 86501, 86502, 86503, 86504, 86509, 86510, 86511, 86512, 86513, 86514, 86519, 86600, 86601, 86602, 86603, 86610, 86611, 86612, 86613, 8670, 8671, 8672, 8673, 8674, 8675, 8676, 8677, 8678, 8679, 86800, 86801, 86802, 86803, 86804, 86809, 86810, 86811, 86812, 86813, 86814, 86819, 8690, 8691, 90000, 90001, 90002, 90003, 9001, 90081, 90082, 90089, 9009, 9010, 9011, 9012, 9013, 90140, 90141, 90142, 90181, 90182, 90183, 90189, 9019, 9020, 90210, 90211, 90219, 90220, 90221, 90222, 90223, 90224, 90225, 90226, 90227, 90229, 90231, 90232, 90233, 90234, 90239, 90240, 90241, 90242, 90249, 90250, 90251, 90252, 90253, 90254, 90255, 90256, 90259, 90281, 90282, 90287, 90289, 9029, 90300, 90301, 90302, 9031, 9032, 9033, 9034, 9035, 9038, 9039, 9040, 9041, 9042, 9043, 90440, 90441, 90442, 90450, 90451, 90452, 90453, 90454, 9046, 9047, 9048, 9049, 9064, 9080, 9081, 9082, 9083, 9084, 925, 9251, 9252, 9260, 92611, 92612, 92619, 9268, 9269, 92700, 92701, 92702, 92703, 92709, 92710, 92711, 92720, 92721, 9273, 9278, 9279, 92800, 92801, 92810, 92811, 92820, 92821, 9283, 9288, 9289, 9290, 9299 |
| 235 | Open wounds of head; neck; and trunk | 8700, 8701, 8702, 8703, 8704, 8708, 8709, 8710, 8711, 8712, 8713, 8714, 8715, 8716, 8717, 8719, 87200, 87201, 87202, 87210, 87211, 87212, 87261, 87262, 87263, 87264, 87269, 87271, 87272, 87273, 87274, 87279, 8728, 8729, 8730, 8731, 87320, 87321, 87322, 87323, 87329, 87330, 87331, 87332, 87333, 87339, 87340, 87341, 87342, 87343, 87344, 87349, 87350, 87351, 87352, 87353, 87354, 87359, 87360, 87361, 87362, 87363, 87364, 87365, 87369, 87370, 87371, 87372, 87373, 87374, 87375, 87379, 8738, 8739, 87400, 87401, 87402, 87410, 87411, 87412, 8742, 8743, 8744, 8745, 8748, 8749, 8750, 8751, 8760, 8761, 8770, 8771, 8780, 8781, 8782, 8783, 8784, 8785, 8786, 8787, 8788, 8789, 8790, 8791, 8792, 8793, 8794, 8795, 8796, 8797, 8798, 8799, 9060 |
| 236 | Open wounds of extremities | 88000, 88001, 88002, 88003, 88009, 88010, 88011, 88012, 88013, 88019, 88020, 88021, 88022, 88023, 88029, 88100, 88101, 88102, 88110, 88111, 88112, 88120, 88121, 88122, 8820, 8821, 8822, 8830, 8831, 8832, 8840, 8841, 8842, 8850, 8851, 8860, 8861, 8870, 8871, 8872, 8873, 8874, 8875, 8876, 8877, 8900, 8901, 8902, 8910, 8911, 8912, 8920, 8921, 8922, 8930, 8931, 8932, 8940, 8941, 8942, 8950, 8951, 8960, 8961, 8962, 8963, 8970, 8971, 8972, 8973, 8974, 8975, 8976, 8977, 9058, 9059, 9061 |
| 237 | Complication of device; implant or graft | 27950, 27951, 27952, 27953, 41402, 41403, 41404, 41405, 41407, 44030, 44031, 44032, 56960, 56961, 56969, 59682, 59683, 62931, 62932, 99600, 99601, 99602, 99603, 99604, 99609, 9961, 9962, 99630, 99631, 99632, 99639, 9964, 99640, 99641, 99642, 99643, 99644, 99645, 99646, 99647, 99649, 99651, 99652, 99653, 99654, 99655, 99656, 99657, 99659, 9966, 99660, 99661, 99662, 99663, 99664, 99665, 99666, 99667, 99668, 99669, 9967, 99670, 99671, 99672, 99673, 99674, 99675, 99676, 99677, 99678, 99679, 99680, 99681, 99682, 99683, 99684, 99685, 99686, 99687, 99688, 99689, 99690, 99691, 99692, 99693, 99694, 99695, 99696, 99699, 99931, 99932, 99933 |
| 238 | Complications of surgical procedures or medical care | 27661, 27783, 27788, 2853, 28741, 3490, 3491, 34931, 41511, 4294, 4582, 45821, 45829, 5121, 5122, 5187, 5190, 51900, 51901, 51902, 51909, 53086, 53087, 53640, 53641, 53642, 53649, 53901, 53909, 53981, 53989, 5642, 5643, 5644, 5696, 56962, 56971, 56979, 5793, 59681, 78062, 78063, 78066, 9093, 99524, 9954, 99586, 9970, 99700, 99701, 99702, 99709, 9971, 9972, 9973, 99731, 99732, 99739, 9974, 99741, 99749, 9975, 99760, 99761, 99762, 99769, 99771, 99772, 99779, 9979, 99791, 99799, 9980, 99800, 99801, 99802, 99809, 9981, 99811, 99812, 99813, 9982, 9983, 99830, 99831, 99832, 99833, 9984, 9985, 99851, 99859, 9986, 9987, 9988, 99881, 99882, 99883, 99889, 9989, 9990, 9991, 9992, 9993, 99934, 99939, 9994, 99941, 99942, 99949, 9995, 99951, 99952, 99959, 9996, 99960, 99961, 99962, 99963, 99969, 9997, 99970, 99971, 99972, 99973, 99974, 99975, 99976, 99977, 99978, 99979, 9998, 99980, 99981, 99982, 99983, 99984, 99985, 99988, 99989, 9999, V1553, V1580, V1583, V9001, V9009 |
| 239 | Superficial injury; contusion | 9062, 9063, 9100, 9101, 9102, 9103, 9104, 9105, 9106, 9107, 9108, 9109, 9110, 9111, 9112, 9113, 9114, 9115, 9116, 9117, 9118, 9119, 9120, 9121, 9122, 9123, 9124, 9125, 9126, 9127, 9128, 9129, 9130, 9131, 9132, 9133, 9134, 9135, 9136, 9137, 9138, 9139, 9140, 9141, 9142, 9143, 9144, 9145, 9146, 9147, 9148, 9149, 9150, 9151, 9152, 9153, 9154, 9155, 9156, 9157, 9158, 9159, 9160, 9161, 9162, 9163, 9164, 9165, 9166, 9167, 9168, 9169, 9170, 9171, 9172, 9173, 9174, 9175, 9176, 9177, 9178, 9179, 9180, 9181, 9182, 9189, 9190, 9191, 9192, 9193, 9194, 9195, 9196, 9197, 9198, 9199, 920, 9210, 9211, 9212, 9213, 9219, 9220, 9221, 9222, 9223, 92231, 92232, 92233, 9224, 9228, 9229, 92300, 92301, 92302, 92303, 92309, 92310, 92311, 92320, 92321, 9233, 9238, 9239, 92400, 92401, 92410, 92411, 92420, 92421, 9243, 9244, 9245, 9248, 9249 |
| 240 | Burns | 9065, 9066, 9067, 9068, 9069, 9400, 9401, 9402, 9403, 9404, 9405, 9409, 94100, 94101, 94102, 94103, 94104, 94105, 94106, 94107, 94108, 94109, 94110, 94111, 94112, 94113, 94114, 94115, 94116, 94117, 94118, 94119, 94120, 94121, 94122, 94123, 94124, 94125, 94126, 94127, 94128, 94129, 94130, 94131, 94132, 94133, 94134, 94135, 94136, 94137, 94138, 94139, 94140, 94141, 94142, 94143, 94144, 94145, 94146, 94147, 94148, 94149, 94150, 94151, 94152, 94153, 94154, 94155, 94156, 94157, 94158, 94159, 94200, 94201, 94202, 94203, 94204, 94205, 94209, 94210, 94211, 94212, 94213, 94214, 94215, 94219, 94220, 94221, 94222, 94223, 94224, 94225, 94229, 94230, 94231, 94232, 94233, 94234, 94235, 94239, 94240, 94241, 94242, 94243, 94244, 94245, 94249, 94250, 94251, 94252, 94253, 94254, 94255, 94259, 94300, 94301, 94302, 94303, 94304, 94305, 94306, 94309, 94310, 94311, 94312, 94313, 94314, 94315, 94316, 94319, 94320, 94321, 94322, 94323, 94324, 94325, 94326, 94329, 94330, 94331, 94332, 94333, 94334, 94335, 94336, 94339, 94340, 94341, 94342, 94343, 94344, 94345, 94346, 94349, 94350, 94351, 94352, 94353, 94354, 94355, 94356, 94359, 94400, 94401, 94402, 94403, 94404, 94405, 94406, 94407, 94408, 94410, 94411, 94412, 94413, 94414, 94415, 94416, 94417, 94418, 94420, 94421, 94422, 94423, 94424, 94425, 94426, 94427, 94428, 94430, 94431, 94432, 94433, 94434, 94435, 94436, 94437, 94438, 94440, 94441, 94442, 94443, 94444, 94445, 94446, 94447, 94448, 94450, 94451, 94452, 94453, 94454, 94455, 94456, 94457, 94458, 94500, 94501, 94502, 94503, 94504, 94505, 94506, 94509, 94510, 94511, 94512, 94513, 94514, 94515, 94516, 94519, 94520, 94521, 94522, 94523, 94524, 94525, 94526, 94529, 94530, 94531, 94532, 94533, 94534, 94535, 94536, 94539, 94540, 94541, 94542, 94543, 94544, 94545, 94546, 94549, 94550, 94551, 94552, 94553, 94554, 94555, 94556, 94559, 9460, 9461, 9462, 9463, 9464, 9465, 9470, 9471, 9472, 9473, 9474, 9478, 9479, 94800, 94810, 94811, 94820, 94821, 94822, 94830, 94831, 94832, 94833, 94840, 94841, 94842, 94843, 94844, 94850, 94851, 94852, 94853, 94854, 94855, 94860, 94861, 94862, 94863, 94864, 94865, 94866, 94870, 94871, 94872, 94873, 94874, 94875, 94876, 94877, 94880, 94881, 94882, 94883, 94884, 94885, 94886, 94887, 94888, 94890, 94891, 94892, 94893, 94894, 94895, 94896, 94897, 94898, 94899, 9490, 9491, 9492, 9493, 9494, 9495 |
| 241 | Poisoning by psychotropic agents | 9690, 96900, 96901, 96902, 96903, 96904, 96905, 96909, 9691, 9692, 9693, 9694, 9695, 9696, 9697, 96970, 96971, 96972, 96973, 96979, 9698, 9699 |
| 242 | Poisoning by other medications and drugs | 52801, 52802, 9090, 9095, 9600, 9601, 9602, 9603, 9604, 9605, 9606, 9607, 9608, 9609, 9610, 9611, 9612, 9613, 9614, 9615, 9616, 9617, 9618, 9619, 9620, 9621, 9622, 9623, 9624, 9625, 9626, 9627, 9628, 9629, 9630, 9631, 9632, 9633, 9634, 9635, 9638, 9639, 9640, 9641, 9642, 9643, 9644, 9645, 9646, 9647, 9648, 9649, 9651, 9654, 9655, 9656, 96561, 96569, 9657, 9658, 9659, 9660, 9661, 9662, 9663, 9664, 9670, 9671, 9672, 9673, 9674, 9675, 9676, 9678, 9679, 9680, 9681, 9682, 9683, 9684, 9685, 9686, 9687, 9689, 9700, 9701, 9708, 97081, 97089, 9709, 9710, 9711, 9712, 9713, 9719, 9720, 9721, 9722, 9723, 9724, 9725, 9726, 9727, 9728, 9729, 9730, 9731, 9732, 9733, 9734, 9735, 9736, 9738, 9739, 9740, 9741, 9742, 9743, 9744, 9745, 9746, 9747, 9750, 9751, 9752, 9753, 9754, 9755, 9756, 9757, 9758, 9760, 9761, 9762, 9763, 9764, 9765, 9766, 9767, 9768, 9769, 9770, 9771, 9772, 9773, 9774, 9778, 9779, 9780, 9781, 9782, 9783, 9784, 9785, 9786, 9788, 9789, 9790, 9791, 9792, 9793, 9794, 9795, 9796, 9797, 9799, 9952, 99520, 99521, 99522, 99523, 99527, 99529 |
| 243 | Poisoning by nonmedicinal substances | 9091, 9801, 9802, 9803, 9808, 9809, 981, 9820, 9821, 9822, 9823, 9824, 9828, 9830, 9831, 9832, 9839, 9840, 9841, 9848, 9849, 9850, 9851, 9852, 9853, 9854, 9855, 9856, 9858, 9859, 986, 9870, 9871, 9872, 9873, 9874, 9875, 9876, 9877, 9878, 9879, 9880, 9881, 9882, 9888, 9889, 9890, 9891, 9892, 9893, 9894, 9895, 9896, 9897, 9898, 98981, 98982, 98983, 98984, 98989, 9899 |
| 244 | Other injuries and conditions due to external causes | 7960, 7990, 79901, 79902, 9071, 9073, 9074, 9075, 9079, 9085, 9086, 9089, 9092, 9094, 9099, 9300, 9301, 9302, 9308, 9309, 931, 932, 9330, 9331, 9340, 9341, 9348, 9349, 9350, 9351, 9352, 936, 937, 938, 9390, 9391, 9392, 9393, 9399, 9500, 9501, 9502, 9503, 9509, 9510, 9511, 9512, 9513, 9514, 9515, 9516, 9517, 9518, 9519, 9530, 9531, 9532, 9533, 9534, 9535, 9538, 9539, 9540, 9541, 9548, 9549, 9550, 9551, 9552, 9553, 9554, 9555, 9556, 9557, 9558, 9559, 9560, 9561, 9562, 9563, 9564, 9565, 9568, 9569, 9570, 9571, 9578, 9579, 9580, 9581, 9582, 9583, 9584, 9585, 9586, 9587, 9588, 95890, 95891, 95892, 95893, 95899, 9590, 95901, 95909, 9591, 95911, 95912, 95913, 95914, 95919, 9592, 9593, 9594, 9595, 9596, 9597, 9598, 9599, 990, 9910, 9911, 9912, 9913, 9914, 9915, 9916, 9918, 9919, 9920, 9921, 9922, 9923, 9924, 9925, 9926, 9927, 9928, 9929, 9930, 9931, 9932, 9933, 9934, 9938, 9939, 9940, 9941, 9942, 9943, 9944, 9945, 9946, 9947, 9948, 9949, 9955, 99550, 99551, 99552, 99553, 99554, 99555, 99559, 99580, 99581, 99582, 99583, 99584, 99585, 99589, 99590, 99593, 99594, V155, V1551, V1559, V156, V1588, V713, V714, V716, V9010, V9011, V9012, V902, V9031, V9032, V9033, V9039, V9081, V9083, V9089, V909 |
| 245 | Syncope | 7802 |
| 246 | Fever of unknown origin | 7806, 78060, 78061 |
| 247 | Lymphadenitis | 2891, 2892, 2893, 683, 7856 |
| 248 | Gangrene | 44024, 7854 |
| 249 | Shock | 78550, 78551, 78552, 78559 |
| 250 | Nausea and vomiting | 7870, 78701, 78702, 78703, 78704 |
| 251 | Abdominal pain | 7890, 78900, 78901, 78902, 78903, 78904, 78905, 78906, 78907, 78909, 78960, 78961, 78962, 78963, 78964, 78965, 78966, 78967, 78969 |
| 252 | Malaise and fatigue | 7807, 78071, 78079 |
| 253 | Allergic reactions | 4771, 5186, 5583, 6910, 6918, 6920, 6921, 6922, 6923, 6924, 6925, 6926, 69270, 69271, 69272, 69273, 69274, 69279, 69281, 69282, 69283, 69284, 69289, 6929, 6930, 6931, 6938, 6939, 7080, 7081, 7082, 7083, 7084, 7085, 7088, 7089, 9950, 9953, 99560, 99561, 99562, 99563, 99564, 99565, 99566, 99567, 99568, 99569, 9957, V071, V1381, V140, V141, V142, V143, V144, V145, V146, V147, V148, V149, V150, V1501, V1502, V1503, V1504, V1505, V1506, V1507, V1508, V1509, V727 |
| 254 | Rehabilitation care; fitting of prostheses; and adjustment of devices | V520, V521, V524, V528, V529, V538, V570, V571, V572, V5721, V5722, V573, V574, V5781, V5789, V579, V5882 |
| 255 | Administrative/social admission | V200, V201, V202, V2031, V2032, V600, V601, V602, V603, V604, V605, V606, V608, V6081, V6089, V609, V610, V6101, V6102, V6103, V6104, V6105, V6106, V6107, V6108, V6109, V611, V6110, V6111, V6112, V6120, V6121, V6122, V6123, V6124, V6125, V6129, V613, V6141, V6142, V6149, V615, V616, V617, V618, V619, V620, V621, V622, V6221, V6222, V6229, V623, V624, V625, V626, V6281, V6282, V6283, V6289, V629, V630, V631, V632, V638, V639, V650, V651, V6511, V6519, V652, V653, V654, V6540, V6541, V6543, V6544, V6545, V6549, V655, V658, V659, V680, V681, V682, V6881, V6889, V689 |
| 256 | Medical examination/evaluation | V290, V291, V292, V293, V298, V299, V6801, V6809, V700, V703, V704, V705, V706, V707, V708, V709, V718, V719, V7231, V7232, V725, V726, V7260, V7261, V7262, V7263, V7269, V728, V7281, V7282, V7283, V7284, V7285, V7286, V729 |
| 257 | Other aftercare | V51, V510, V518, V539, V5390, V5399, V548, V5481, V5482, V5489, V549, V558, V559, V583, V5830, V5831, V5832, V584, V5841, V5842, V5843, V5844, V5849, V5861, V5862, V5863, V5864, V5865, V5866, V5867, V5868, V5869, V5871, V5872, V5873, V5874, V5875, V5876, V5877, V5878, V588, V5881, V5883, V5889, V589, V660, V665, V666, V667, V669, V670, V6700, V6701, V6709, V6751, V6759, V676, V679 |
| 258 | Other screening for suspected conditions (not mental disorders or infectious disease) | 7929, 79500, 79507, 79508, 79518, 7954, 79677, 79678, V280, V281, V282, V283, V284, V285, V288, V2881, V2882, V2889, V289, V715, V7181, V7189, V7240, V7241, V760, V761, V7610, V7611, V7612, V7619, V762, V763, V7641, V7642, V7643, V7644, V7645, V7646, V7647, V7649, V7650, V7651, V7652, V768, V7681, V7689, V769, V770, V771, V772, V773, V774, V775, V776, V777, V778, V779, V7791, V7799, V780, V781, V782, V783, V788, V789, V800, V8001, V8009, V801, V802, V803, V810, V811, V812, V813, V814, V815, V816, V820, V821, V822, V823, V824, V825, V826, V8271, V8279, V828, V8281, V8289, V829 |
| 259 | Residual codes; unclassified | 3020, 32700, 32701, 32709, 32710, 32711, 32712, 32713, 32714, 32719, 32720, 32721, 32722, 32723, 32724, 32725, 32726, 32727, 32729, 32740, 32741, 32742, 32743, 32744, 32749, 32751, 32759, 3278, 78002, 7801, 78050, 78051, 78052, 78053, 78054, 78055, 78056, 78057, 78058, 78059, 78064, 78065, 7809, 78093, 78094, 78095, 78096, 78097, 78099, 7815, 7816, 7823, 78261, 78262, 7828, 7829, 7830, 7836, 7842, 7901, 7906, 7909, 79091, 79092, 79093, 79094, 79095, 79099, 7932, 7939, 79399, 7949, 79581, 79582, 79589, 7963, 7964, 7965, 7966, 7969, 7980, 7981, 7982, 7989, 7992, 79921, 79922, 79923, 79924, 79925, 79929, 7993, 7998, 79981, 79982, 79989, 7999, V070, V072, V073, V0731, V0739, V0751, V0752, V0759, V078, V079, V131, V138, V1389, V139, V152, V1521, V1522, V1529, V153, V1581, V1584, V1585, V1586, V1587, V1589, V159, V160, V161, V162, V163, V164, V1640, V1641, V1642, V1643, V1649, V165, V1651, V1652, V1659, V166, V167, V168, V169, V170, V171, V172, V173, V174, V1741, V1749, V175, V176, V177, V178, V1781, V1789, V180, V181, V1811, V1819, V182, V183, V184, V185, V1851, V1859, V186, V1861, V1869, V187, V188, V189, V190, V191, V1911, V1919, V192, V193, V194, V195, V196, V197, V198, V210, V211, V212, V218, V219, V418, V419, V428, V4281, V4282, V4283, V4284, V4289, V429, V438, V4381, V4382, V4383, V4389, V447, V448, V449, V4571, V4572, V4573, V4574, V4575, V4576, V4577, V4578, V4579, V4583, V4584, V4586, V4587, V4588, V4589, V460, V463, V468, V469, V470, V471, V472, V479, V480, V488, V489, V498, V4981, V4982, V4983, V4984, V4986, V4987, V4989, V499, V500, V501, V503, V5041, V5042, V5049, V508, V509, V590, V5901, V5902, V5909, V591, V592, V593, V594, V595, V596, V5970, V5971, V5972, V5973, V5974, V598, V599, V640, V6400, V6401, V6402, V6403, V6404, V6405, V6406, V6407, V6408, V6409, V641, V642, V643, V644, V6441, V6442, V6443, V690, V691, V692, V693, V694, V695, V698, V699, V8301, V8302, V8381, V8389, V8401, V8402, V8403, V8404, V8409, V848, V8481, V8489, V851, V8552, V860, V861, V8701, V8702, V8709, V8711, V8712, V8719, V872, V8731, V8732, V8739, V8741, V8742, V8743, V8744, V8745, V8746, V8749, V8801, V8802, V8803, V8811, V8812, V8901, V8902, V8903, V8904, V8905, V8909 |
| 650 | Adjustment disorders | 3090, 3091, 30922, 30923, 30924, 30928, 30929, 3093, 3094, 30982, 30983, 30989, 3099 |
| 651 | Anxiety disorders | 29384, 30000, 30001, 30002, 30009, 30010, 30020, 30021, 30022, 30023, 30029, 3003, 3005, 30089, 3009, 3080, 3081, 3082, 3083, 3084, 3089, 30981, 3130, 3131, 31321, 31322, 3133, 31382, 31383 |
| 652 | Attention-deficit, conduct, and disruptive behavior disorders | 31200, 31201, 31202, 31203, 31210, 31211, 31212, 31213, 31220, 31221, 31222, 31223, 3124, 3128, 31281, 31282, 31289, 3129, 31381, 31400, 31401, 3141, 3142, 3148, 3149 |
| 653 | Delirium, dementia, and amnestic and other cognitive disorders | 2900, 29010, 29011, 29012, 29013, 29020, 29021, 2903, 29040, 29041, 29042, 29043, 2908, 2909, 2930, 2931, 2940, 2941, 29410, 29411, 29420, 29421, 2948, 2949, 3100, 3102, 3108, 31081, 31089, 3109, 3310, 3311, 33111, 33119, 3312, 33182, 797 |
| 654 | Developmental disorders | 3070, 3079, 31500, 31501, 31502, 31509, 3151, 3152, 31531, 31532, 31534, 31535, 31539, 3154, 3155, 3158, 3159, 317, 3180, 3181, 3182, 319, V400, V401 |
| 655 | Disorders usually diagnosed in infancy, childhood, or adolescence | 29900, 29901, 29910, 29911, 29980, 29981, 29990, 29991, 30720, 30721, 30722, 30723, 3073, 3076, 3077, 30921, 31323, 31389, 3139 |
| 656 | Impulse control disorders, NEC | 31230, 31231, 31232, 31233, 31234, 31235, 31239 |
| 657 | Mood disorders | 29383, 29600, 29601, 29602, 29603, 29604, 29605, 29606, 29610, 29611, 29612, 29613, 29614, 29615, 29616, 29620, 29621, 29622, 29623, 29624, 29625, 29626, 29630, 29631, 29632, 29633, 29634, 29635, 29636, 29640, 29641, 29642, 29643, 29644, 29645, 29646, 29650, 29651, 29652, 29653, 29654, 29655, 29656, 29660, 29661, 29662, 29663, 29664, 29665, 29666, 2967, 29680, 29681, 29682, 29689, 29690, 29699, 3004, 311 |
| 658 | Personality disorders | 3010, 30110, 30111, 30112, 30113, 30120, 30121, 30122, 3013, 3014, 30150, 30151, 30159, 3016, 3017, 30181, 30182, 30183, 30184, 30189, 3019 |
| 659 | Schizophrenia and other psychotic disorders | 29381, 29382, 29500, 29501, 29502, 29503, 29504, 29505, 29510, 29511, 29512, 29513, 29514, 29515, 29520, 29521, 29522, 29523, 29524, 29525, 29530, 29531, 29532, 29533, 29534, 29535, 29540, 29541, 29542, 29543, 29544, 29545, 29550, 29551, 29552, 29553, 29554, 29555, 29560, 29561, 29562, 29563, 29564, 29565, 29570, 29571, 29572, 29573, 29574, 29575, 29580, 29581, 29582, 29583, 29584, 29585, 29590, 29591, 29592, 29593, 29594, 29595, 2970, 2971, 2972, 2973, 2978, 2979, 2980, 2981, 2982, 2983, 2984, 2988, 2989 |
| 660 | Alcohol-related disorders | 2910, 2911, 2912, 2913, 2914, 2915, 2918, 29181, 29182, 29189, 2919, 30300, 30301, 30302, 30303, 30390, 30391, 30392, 30393, 30500, 30501, 30502, 30503, 3575, 4255, 5353, 53530, 53531, 5710, 5711, 5712, 5713, 76071, 9800 |
| 661 | Substance-related disorders | 2920, 29211, 29212, 2922, 29281, 29282, 29283, 29284, 29285, 29289, 2929, 30400, 30401, 30402, 30403, 30410, 30411, 30412, 30413, 30420, 30421, 30422, 30423, 30430, 30431, 30432, 30433, 30440, 30441, 30442, 30443, 30450, 30451, 30452, 30453, 30460, 30461, 30462, 30463, 30470, 30471, 30472, 30473, 30480, 30481, 30482, 30483, 30490, 30491, 30492, 30493, 30520, 30521, 30522, 30523, 30530, 30531, 30532, 30533, 30540, 30541, 30542, 30543, 30550, 30551, 30552, 30553, 30560, 30561, 30562, 30563, 30570, 30571, 30572, 30573, 30580, 30581, 30582, 30583, 30590, 30591, 30592, 30593, 64830, 64831, 64832, 64833, 64834, 65550, 65551, 65553, 76072, 76073, 76075, 7795, 96500, 96501, 96502, 96509, V6542 |
| 662 | Suicide and intentional self-inflicted injury | E9500, E9501, E9502, E9503, E9504, E9505, E9506, E9507, E9508, E9509, E9510, E9511, E9518, E9520, E9521, E9528, E9529, E9530, E9531, E9538, E9539, E954, E9550, E9551, E9552, E9553, E9554, E9555, E9556, E9557, E9559, E956, E9570, E9571, E9572, E9579, E9580, E9581, E9582, E9583, E9584, E9585, E9586, E9587, E9588, E9589, E959, V6284 |
| 663 | Screening and history of mental health and substance abuse codes | 3051, 30510, 30511, 30512, 30513, 33392, 7903, V110, V111, V112, V113, V114, V118, V119, V154, V1541, V1542, V1549, V1582, V6285, V663, V701, V702, V7101, V7102, V7109, V790, V791, V792, V793, V798, V799 |
| 670 | Miscellaneous mental health disorders | 29389, 2939, 30011, 30012, 30013, 30014, 30015, 30016, 30019, 3006, 3007, 30081, 30082, 3021, 3022, 3023, 3024, 30250, 30251, 30252, 30253, 3026, 30270, 30271, 30272, 30273, 30274, 30275, 30276, 30279, 30281, 30282, 30283, 30284, 30285, 30289, 3029, 3060, 3061, 3062, 3063, 3064, 30650, 30651, 30652, 30653, 30659, 3066, 3067, 3068, 3069, 3071, 30740, 30741, 30742, 30743, 30744, 30745, 30746, 30747, 30748, 30749, 30750, 30751, 30752, 30753, 30754, 30759, 30780, 30781, 30789, 3101, 316, 64840, 64841, 64842, 64843, 64844, V402, V403, V4031, V4039, V409, V673 |
| 2601 | E Codes: Cut/pierced | E9200, E9201, E9202, E9203, E9204, E9205, E9208, E9209, E966, E974, E986 |
| 2602 | E Codes: Drowning/submersion | E8300, E8301, E8302, E8303, E8304, E8305, E8306, E8307, E8308, E8309, E8317, E8320, E8321, E8322, E8323, E8324, E8325, E8326, E8327, E8328, E8329, E9100, E9101, E9102, E9103, E9104, E9108, E9109, E964, E984 |
| 2603 | E Codes: Fall | E8800, E8801, E8809, E8810, E8811, E882, E8830, E8831, E8832, E8839, E8840, E8841, E8842, E8843, E8844, E8845, E8846, E8849, E885, E8850, E8851, E8852, E8853, E8854, E8859, E8860, E8869, E888, E8880, E8881, E8888, E8889, E9681, E9870, E9871, E9872, E9879 |
| 2604 | E Codes: Fire/burn | E8900, E8901, E8902, E8903, E8908, E8909, E8910, E8911, E8912, E8913, E8918, E8919, E892, E8930, E8931, E8932, E8938, E8939, E894, E895, E896, E897, E8980, E8981, E899, E9240, E9241, E9242, E9248, E9249, E961, E9680, E9683, E9793, E9881, E9882, E9887 |
| 2605 | E Codes: Firearm | E9220, E9221, E9222, E9223, E9228, E9229, E9287, E9650, E9651, E9652, E9653, E9654, E970, E9794, E9850, E9851, E9852, E9853, E9854 |
| 2606 | E Codes: Machinery | E9190, E9191, E9192, E9193, E9194, E9195, E9196, E9197, E9198, E9199 |
| 2607 | E Codes: Motor vehicle traffic (MVT) | E8100, E8101, E8102, E8103, E8104, E8105, E8106, E8107, E8108, E8109, E8110, E8111, E8112, E8113, E8114, E8115, E8116, E8117, E8118, E8119, E8120, E8121, E8122, E8123, E8124, E8125, E8126, E8127, E8128, E8129, E8130, E8131, E8132, E8133, E8134, E8135, E8136, E8137, E8138, E8139, E8140, E8141, E8142, E8143, E8144, E8145, E8146, E8147, E8148, E8149, E8150, E8151, E8152, E8153, E8154, E8155, E8156, E8157, E8158, E8159, E8160, E8161, E8162, E8163, E8164, E8165, E8166, E8167, E8168, E8169, E8170, E8171, E8172, E8173, E8174, E8175, E8176, E8177, E8178, E8179, E8180, E8181, E8182, E8183, E8184, E8185, E8186, E8187, E8188, E8189, E8190, E8191, E8192, E8193, E8194, E8195, E8196, E8197, E8198, E8199, E9685, E9885 |
| 2608 | E Codes: Pedal cyclist; not MVT | E8003, E8013, E8023, E8033, E8043, E8053, E8063, E8073, E8206, E8216, E8226, E8236, E8246, E8256, E8261, E8269 |
| 2609 | E Codes: Pedestrian; not MVT | E8002, E8012, E8022, E8032, E8042, E8052, E8062, E8072, E8207, E8217, E8227, E8237, E8247, E8257, E8260, E8270, E8280, E8290 |
| 2610 | E Codes: Transport; not MVT | E8000, E8001, E8008, E8009, E8010, E8011, E8018, E8019, E8020, E8021, E8028, E8029, E8030, E8031, E8038, E8039, E8040, E8041, E8048, E8049, E8050, E8051, E8058, E8059, E8060, E8061, E8068, E8069, E8070, E8071, E8078, E8079, E8200, E8201, E8202, E8203, E8204, E8205, E8208, E8209, E8210, E8211, E8212, E8213, E8214, E8215, E8218, E8219, E8220, E8221, E8222, E8223, E8224, E8225, E8228, E8229, E8230, E8231, E8232, E8233, E8234, E8235, E8238, E8239, E8240, E8241, E8242, E8243, E8244, E8245, E8248, E8249, E8250, E8251, E8252, E8253, E8254, E8255, E8258, E8259, E8262, E8263, E8264, E8268, E8272, E8273, E8274, E8278, E8279, E8282, E8284, E8288, E8289, E8294, E8298, E8299, E8310, E8311, E8312, E8313, E8314, E8315, E8316, E8318, E8319, E8330, E8331, E8332, E8333, E8334, E8335, E8336, E8337, E8338, E8339, E8340, E8341, E8342, E8343, E8344, E8345, E8346, E8347, E8348, E8349, E8350, E8351, E8352, E8353, E8354, E8355, E8356, E8357, E8358, E8359, E8360, E8361, E8362, E8363, E8364, E8365, E8366, E8367, E8368, E8369, E8370, E8371, E8372, E8373, E8374, E8375, E8376, E8377, E8378, E8379, E8380, E8381, E8382, E8383, E8384, E8385, E8386, E8387, E8388, E8389, E8400, E8401, E8402, E8403, E8404, E8405, E8406, E8407, E8408, E8409, E8410, E8411, E8412, E8413, E8414, E8415, E8416, E8417, E8418, E8419, E8426, E8427, E8428, E8429, E8430, E8431, E8432, E8433, E8434, E8435, E8436, E8437, E8438, E8439, E8440, E8441, E8442, E8443, E8444, E8445, E8446, E8447, E8448, E8449, E8450, E8458, E8459, E9886 |
| 2611 | E Codes: Natural/environment | E9000, E9001, E9009, E9010, E9011, E9018, E9019, E9020, E9021, E9022, E9028, E9029, E903, E9040, E9041, E9042, E9043, E9049, E9050, E9051, E9052, E9053, E9054, E9055, E9056, E9057, E9058, E9059, E9060, E9061, E9062, E9063, E9064, E9065, E9068, E9069, E907, E908, E9080, E9081, E9082, E9083, E9084, E9088, E9089, E909, E9090, E9091, E9092, E9093, E9094, E9098, E9099, E9280, E9281, E9282, E9883 |
| 2612 | E Codes: Overexertion | E927, E9270, E9271, E9272, E9273, E9278, E9279 |
| 2613 | E Codes: Poisoning | E8500, E8501, E8502, E8503, E8504, E8505, E8506, E8507, E8508, E8509, E851, E8520, E8521, E8522, E8523, E8524, E8525, E8528, E8529, E8530, E8531, E8532, E8538, E8539, E8540, E8541, E8542, E8543, E8548, E8550, E8551, E8552, E8553, E8554, E8555, E8556, E8558, E8559, E856, E857, E8580, E8581, E8582, E8583, E8584, E8585, E8586, E8587, E8588, E8589, E8600, E8601, E8602, E8603, E8604, E8608, E8609, E8610, E8611, E8612, E8613, E8614, E8615, E8616, E8619, E8620, E8621, E8622, E8623, E8624, E8629, E8630, E8631, E8632, E8633, E8634, E8635, E8636, E8637, E8638, E8639, E8640, E8641, E8642, E8643, E8644, E8650, E8651, E8652, E8653, E8654, E8655, E8658, E8659, E8660, E8661, E8662, E8663, E8664, E8665, E8666, E8667, E8668, E8669, E867, E8680, E8681, E8682, E8683, E8688, E8689, E8690, E8691, E8692, E8693, E8694, E8698, E8699, E9620, E9621, E9622, E9629, E972, E9800, E9801, E9802, E9803, E9804, E9805, E9806, E9807, E9808, E9809, E9810, E9811, E9818, E9820, E9821, E9828, E9829 |
| 2614 | E Codes: Struck by; against | E916, E9170, E9171, E9172, E9173, E9174, E9175, E9176, E9177, E9178, E9179, E9274, E9600, E9682, E973, E975 |
| 2615 | E Codes: Suffocation | E911, E912, E9130, E9131, E9132, E9133, E9138, E9139, E9284, E9285, E963, E9830, E9831, E9838, E9839 |
| 2616 | E Codes: Adverse effects of medical care | E8700, E8701, E8702, E8703, E8704, E8705, E8706, E8707, E8708, E8709, E8710, E8711, E8712, E8713, E8714, E8715, E8716, E8717, E8718, E8719, E8720, E8721, E8722, E8723, E8724, E8725, E8726, E8728, E8729, E8730, E8731, E8732, E8733, E8734, E8735, E8736, E8738, E8739, E8740, E8741, E8742, E8743, E8744, E8745, E8748, E8749, E8750, E8751, E8752, E8758, E8759, E8760, E8761, E8762, E8763, E8764, E8765, E8766, E8767, E8768, E8769, E8780, E8781, E8782, E8783, E8784, E8785, E8786, E8788, E8789, E8790, E8791, E8792, E8793, E8794, E8795, E8796, E8797, E8798, E8799 |
| 2617 | E Codes: Adverse effects of medical drugs | E9300, E9301, E9302, E9303, E9304, E9305, E9306, E9307, E9308, E9309, E9310, E9311, E9312, E9313, E9314, E9315, E9316, E9317, E9318, E9319, E9320, E9321, E9322, E9323, E9324, E9325, E9326, E9327, E9328, E9329, E9330, E9331, E9332, E9333, E9334, E9335, E9336, E9337, E9338, E9339, E9340, E9341, E9342, E9343, E9344, E9345, E9346, E9347, E9348, E9349, E9350, E9351, E9352, E9353, E9354, E9355, E9356, E9357, E9358, E9359, E9360, E9361, E9362, E9363, E9364, E9370, E9371, E9372, E9373, E9374, E9375, E9376, E9378, E9379, E9380, E9381, E9382, E9383, E9384, E9385, E9386, E9387, E9389, E9390, E9391, E9392, E9393, E9394, E9395, E9396, E9397, E9398, E9399, E9400, E9401, E9408, E9409, E9410, E9411, E9412, E9413, E9419, E9420, E9421, E9422, E9423, E9424, E9425, E9426, E9427, E9428, E9429, E9430, E9431, E9432, E9433, E9434, E9435, E9436, E9438, E9439, E9440, E9441, E9442, E9443, E9444, E9445, E9446, E9447, E9450, E9451, E9452, E9453, E9454, E9455, E9456, E9457, E9458, E9460, E9461, E9462, E9463, E9464, E9465, E9466, E9467, E9468, E9469, E9470, E9471, E9472, E9473, E9474, E9478, E9479, E9480, E9481, E9482, E9483, E9484, E9485, E9486, E9488, E9489, E9490, E9491, E9492, E9493, E9494, E9495, E9496, E9497, E9499 |
| 2618 | E Codes: Other specified and classifiable | E846, E847, E848, E914, E915, E918, E9210, E9211, E9218, E9219, E9224, E9225, E9230, E9231, E9232, E9238, E9239, E9250, E9251, E9252, E9258, E9259, E9260, E9261, E9262, E9263, E9264, E9265, E9268, E9269, E9283, E9290, E9291, E9292, E9293, E9294, E9295, E9601, E9655, E9656, E9657, E9658, E9659, E9670, E9671, E9672, E9673, E9674, E9675, E9676, E9677, E9678, E9679, E9684, E9686, E9687, E971, E978, E9790, E9791, E9792, E9795, E9796, E9797, E9798, E9799, E9855, E9856, E9857, E9880, E9884, E9900, E9901, E9902, E9903, E9909, E9910, E9911, E9912, E9913, E9914, E9915, E9916, E9917, E9918, E9919, E992, E9920, E9921, E9922, E9923, E9928, E9929, E993, E9930, E9931, E9932, E9933, E9934, E9935, E9936, E9937, E9938, E9939, E994, E9940, E9941, E9942, E9943, E9948, E9949, E9950, E9951, E9952, E9953, E9954, E9958, E9959, E996, E9960, E9961, E9962, E9963, E9968, E9969, E9970, E9971, E9972, E9973, E9980, E9981, E9988, E9989, E9990, E9991 |
| 2619 | E Codes: Other specified; NEC | E9286, E9288, E9298, E9688, E969, E977, E9888, E989, E995, E9978, E998, E999 |
| 2620 | E Codes: Unspecified | E0000, E0001, E0002, E0008, E0009, E0010, E0011, E0020, E0021, E0022, E0023, E0024, E0025, E0026, E0027, E0028, E0029, E0030, E0031, E0032, E0033, E0039, E0040, E0041, E0042, E0043, E0044, E0049, E0050, E0051, E0052, E0053, E0054, E0059, E0060, E0061, E0062, E0063, E0064, E0065, E0066, E0069, E0070, E0071, E0072, E0073, E0074, E0075, E0076, E0077, E0078, E0079, E0080, E0081, E0082, E0083, E0084, E0089, E0090, E0091, E0092, E0093, E0094, E0095, E0099, E0100, E0101, E0102, E0103, E0109, E0110, E0111, E0119, E0120, E0121, E0122, E0129, E0130, E0131, E0132, E0133, E0134, E0135, E0138, E0139, E0140, E0141, E0149, E0150, E0151, E0152, E0159, E0160, E0161, E0162, E0169, E0170, E0179, E0180, E0181, E0182, E0183, E0190, E0191, E0192, E0199, E0290, E0291, E0292, E0299, E030, E887, E9289, E9299, E9689, E976, E9889, E9979 |
| 2621 | E Codes: Place of occurrence | E8490, E8491, E8492, E8493, E8494, E8495, E8496, E8497, E8498, E8499 |

**Supplementary Table 1.** Single-level CCS categories for diagnoses.

## **Procedure Categories**

| CCS Category | CCS Category Label | ICD-9 Codes | CPT Codes |
| --- | --- | --- | --- |
| 1 | Incision and excision of CNS | 0101, 0109, 0121, 0122, 0123, 0124, 0125, 0126, 0127, 0128, 0131, 0132, 0139, 0141, 0142, 0151, 0152, 0153, 0159 | 61000, 61001, 61020, 61026, 61050, 61055, 61105, 61108, 61120, 61150, 61151, 61154, 61156, 61250, 61253, 61304, 61305, 61312, 61313, 61314, 61315, 61320, 61321, 61322, 61323, 61340, 61345, 61510, 61512, 61514, 61516, 61518, 61519, 61520, 61521, 61522, 61524, 61526, 61530, 61534, 61535, 61536, 61537, 61538, 61539, 61540, 61541, 61543, 61544, 61545, 61556, 61557, 61570, 61571, 61575, 61576, 61582, 61583, 61584, 61585, 61586, 61590, 61591, 61592, 61595, 61596, 61598, 61600, 61601, 61605, 61606, 61607, 61608, 61615, 61616, 61720, 61735, 61736, 61737, 61880, 62161, 62162, 62164 |
| 2 | Insertion, replacement, or removal of extracranial ventricular shunt | 0231, 0232, 0233, 0234, 0235, 0239, 0242, 0243 | 61107, 61210, 62160, 62180, 62190, 62192, 62194, 62200, 62201, 62220, 62223, 62225, 62230, 62252, 62256, 62258 |
| 3 | Laminectomy, excision intervertebral disc | 0302, 0309, 805, 8050, 8051, 8059, 8459, 8460, 8461, 8462, 8463, 8464, 8465, 8466, 8467, 8468, 8469, 8480, 8481, 8482, 8483, 8484, 8485 | 0202T, 0274T, 0275T, 22856, 22858, 22861, 22864, 61343, 62287, 62351, 62380, 63001, 63003, 63005, 63011, 63012, 63015, 63016, 63017, 63020, 63030, 63035, 63040, 63042, 63043, 63044, 63045, 63046, 63047, 63048, 63050, 63051, 63052, 63053, 63055, 63056, 63057, 63064, 63066, 63075, 63076, 63077, 63078, 63081, 63082, 63085, 63086, 63087, 63088, 63090, 63091, 63101, 63102, 63103, 63170, 63172, 63173, 63185, 63190, 63191, 63197, 63200, 63250, 63251, 63252, 63655, 63709, 63740, C9757, G0276, S2348, S2350, S2351 |
| 4 | Diagnostic spinal tap | 0331 | 62270, 62272, 62328, 62329 |
| 5 | Insertion of catheter or spinal stimulator and injection into spinal canal | 038, 0390, 0391, 0392, 0393, 0394 | 62263, 62264, 62280, 62281, 62282, 62320, 62321, 62322, 62323, 62324, 62325, 62326, 62327, 62350, 62355, 63600, 63610, 63650, 63661, 63662, 63663, 63664, 63685, 63688, 64416, 64446, 64448, 64449, 64461, 64462, 64463, 64479, 64480, 64483, 64484 |
| 6 | Decompression peripheral nerve | 0443, 0444, 0449 | 28035, 29848, 64702, 64704, 64708, 64712, 64713, 64714, 64716, 64718, 64719, 64721, 64722, 64726, 64727 |
| 7 | Other diagnostic nervous system procedures | 0111, 0112, 0113, 0114, 0115, 0118, 0119, 0332, 0339, 0411, 0412, 0419, 0511, 0519 | 0106T, 0107T, 0108T, 0109T, 0110T, 0333T, 0434T, 0435T, 0436T, 0533T, 0534T, 0535T, 0536T, 0589T, 0590T, 0639T, 61140, 61750, 61751, 62267, 62269, 64795, 92516, 95782, 95783, 95800, 95801, 95803, 95805, 95806, 95807, 95808, 95810, 95811, 95836, 95857, 95860, 95861, 95863, 95864, 95865, 95866, 95867, 95868, 95869, 95870, 95872, 95874, 95875, 95885, 95886, 95887, 95905, 95907, 95908, 95909, 95910, 95911, 95912, 95913, 95921, 95922, 95923, 95924, 95925, 95926, 95927, 95928, 95929, 95930, 95933, 95937, 95938, 95939, 95940, 95941, 95965, 95966, 95967, 95970, 95971, 95972, 95976, 95977, 95980, 95981, 95982, 95983, 95984, 95999, 96000, 96001, 96002, 96003, 96004, 96020, 96112, 96113, G0255, G0398, G0399, G0400, G0453, S3900, S8040 |
| 8 | Other non-OR or closed therapeutic nervous system procedures | 0102, 0241, 0295, 0321, 0395, 0396, 0480, 0481, 0489, 0531, 0532, 0539 | 0278T, 0398T, 0440T, 0441T, 0442T, 0627T, 0628T, 0629T, 0630T, 22505, 61070, 61796, 61798, 62268, 62273, 62292, 62369, 62370, 63620, 64400, 64405, 64408, 64415, 64417, 64418, 64420, 64421, 64425, 64430, 64435, 64445, 64447, 64450, 64451, 64454, 64455, 64486, 64487, 64488, 64489, 64490, 64491, 64492, 64493, 64494, 64495, 64505, 64510, 64517, 64520, 64530, 64600, 64605, 64610, 64611, 64612, 64615, 64616, 64617, 64620, 64624, 64625, 64630, 64632, 64633, 64635, 64640, 64642, 64644, 64646, 64647, 64680, 64681, 64999, 95873, 95990, 95991, 95992, S9090 |
| 9 | Other OR therapeutic nervous system procedures | 0120, 0129, 016, 0201, 0202, 0203, 0204, 0205, 0206, 0207, 0211, 0212, 0213, 0214, 022, 0221, 0222, 0291, 0292, 0293, 0294, 0296, 0299, 0301, 031, 0329, 034, 0351, 0352, 0353, 0359, 036, 0371, 0372, 0379, 0397, 0398, 0399, 0401, 0402, 0403, 0404, 0405, 0406, 0407, 042, 043, 0441, 0442, 045, 046, 0471, 0472, 0473, 0474, 0475, 0476, 0479, 0491, 0492, 0493, 0499, 050, 0521, 0522, 0523, 0524, 0525, 0529, 0581, 0589, 059, 1761, 8053, 8054, 8458, 8694, 8695, 8696, 8697, 8698 | 0338T, 0339T, 0424T, 0425T, 0426T, 0427T, 0428T, 0429T, 0430T, 0431T, 0432T, 0433T, 0587T, 0588T, 0609T, 0610T, 0611T, 0612T, 22526, 22527, 27003, 27035, 27325, 27326, 28055, 28080, 32664, 61450, 61458, 61460, 61500, 61501, 61531, 61533, 61550, 61552, 61558, 61559, 61563, 61564, 61566, 61567, 61580, 61581, 61618, 61619, 61760, 61770, 61781, 61782, 61783, 61790, 61791, 61797, 61799, 61800, 61850, 61860, 61863, 61864, 61867, 61868, 62100, 62115, 62117, 62120, 62121, 62140, 62141, 62142, 62143, 62145, 62146, 62147, 62148, 62294, 62360, 62361, 62362, 62365, 63265, 63266, 63267, 63268, 63270, 63271, 63272, 63273, 63275, 63276, 63277, 63278, 63280, 63281, 63282, 63283, 63285, 63286, 63287, 63290, 63295, 63300, 63301, 63302, 63303, 63304, 63305, 63306, 63307, 63308, 63621, 63700, 63702, 63704, 63706, 63707, 63710, 63741, 63744, 63746, 64553, 64555, 64561, 64566, 64568, 64569, 64570, 64575, 64580, 64581, 64582, 64583, 64584, 64585, 64590, 64595, 64628, 64629, 64634, 64636, 64643, 64645, 64732, 64734, 64736, 64738, 64740, 64742, 64744, 64746, 64755, 64760, 64763, 64766, 64771, 64772, 64774, 64776, 64778, 64782, 64783, 64784, 64786, 64787, 64788, 64790, 64792, 64802, 64804, 64809, 64818, 64820, 64821, 64822, 64823, 64831, 64832, 64834, 64835, 64836, 64837, 64840, 64856, 64857, 64858, 64859, 64861, 64862, 64864, 64865, 64866, 64868, 64872, 64874, 64876, 64885, 64886, 64890, 64891, 64892, 64893, 64895, 64896, 64897, 64898, 64901, 64902, 64905, 64907, 64910, 64911, 64912, 64913, 67570, 69720, 69725, 69740, 69745, 69955, S2235 |
| 10 | Thyroidectomy, partial or complete | 062, 0631, 0639, 064, 0650, 0651, 0652, 066 | 60210, 60212, 60220, 60225, 60240, 60252, 60254, 60260, 60270, 60271 |
| 11 | Diagnostic endocrine procedures | 0611, 0612, 0613, 0619, 0700, 0701, 0702, 0711, 0712, 0713, 0714, 0715, 0716, 0717, 0719, 0751, 0771, 0791 | 0446T, 0447T, 0448T, 60100, 95249, 95250, 95251 |
| 12 | Other therapeutic endocrine procedures | 0601, 0602, 0609, 067, 0681, 0689, 0691, 0692, 0693, 0694, 0695, 0698, 0699, 0721, 0722, 0729, 073, 0741, 0742, 0743, 0744, 0745, 0749, 0752, 0753, 0754, 0759, 0761, 0762, 0763, 0764, 0765, 0768, 0769, 0772, 0779, 0780, 0781, 0782, 0783, 0784, 0792, 0793, 0794, 0795, 0798, 0799 | 0673T, 32673, 60000, 60200, 60280, 60281, 60300, 60500, 60502, 60505, 60512, 60520, 60521, 60522, 60540, 60545, 60650, 60659, 60699, 61546, 61548, 62165, 79005 |
| 13 | Corneal transplant | 1160, 1161, 1162, 1163, 1164, 1169 | 65710, 65730, 65750, 65755, 65756, 65757 |
| 14 | Glaucoma procedures | 1251, 1252, 1253, 1254, 1255, 1259, 1261, 1262, 1263, 1264, 1265, 1266, 1267, 1269, 1271, 1272, 1273, 1274, 1279 | 0253T, 0449T, 0450T, 0621T, 0622T, 0671T, 65820, 65850, 65855, 66150, 66155, 66160, 66170, 66172, 66174, 66175, 66179, 66180, 66183, 66184, 66185, 66700, 66710, 66711, 66720, 66740, 66761 |
| 15 | Lens and cataract procedures | 1311, 1319, 132, 133, 1341, 1342, 1343, 1351, 1359, 1361, 1362, 1363, 1364, 1365, 1366, 1369, 1370, 1371, 1372, 138, 139, 1390, 1391 | 0308T, 0617T, 0618T, 65920, 66820, 66821, 66825, 66830, 66840, 66850, 66852, 66920, 66930, 66940, 66982, 66983, 66984, 66985, 66986, 66987, 66988, 66989, 66991 |
| 16 | Repair of retinal tear, detachment | 1431, 1432, 1433, 1434, 1435, 1439, 1441, 1449, 1451, 1452, 1453, 1454, 1455, 1459 | 67039, 67040, 67101, 67105, 67107, 67108, 67110, 67113, 67141, 67145 |
| 17 | Destruction of lesion of retina and choroid | 1421, 1422, 1423, 1424, 1425, 1426, 1427, 1429 | 67208, 67210, 67218, 67220, 67221, 67225, 67227, 67228, 67229, G0186 |
| 18 | Diagnostic procedures on eye | 0811, 0819, 0911, 0912, 0919, 1021, 1029, 1121, 1122, 1129, 1221, 1222, 1229, 1411, 1419, 1501, 1509, 1621, 1622, 1623, 1629 | 0329T, 0330T, 0615T, 65410, 65430, 65800, 67346, 67400, 67415, 67450, 67810, 68100, 68510, 68525, 92145, S0620, S0621, S3000 |
| 19 | Other therapeutic procedures on eyelids, conjunctiva, cornea | 0801, 0802, 0809, 0820, 0821, 0822, 0823, 0824, 0825, 0831, 0832, 0833, 0834, 0835, 0836, 0837, 0838, 0841, 0842, 0843, 0844, 0849, 0851, 0852, 0859, 0861, 0862, 0863, 0864, 0869, 0870, 0871, 0872, 0873, 0874, 0881, 0882, 0883, 0884, 0885, 0886, 0887, 0889, 0891, 0892, 0893, 0899, 090, 0920, 0921, 0922, 0923, 093, 0941, 0942, 0943, 0944, 0949, 0951, 0952, 0953, 0959, 096, 0971, 0972, 0973, 0981, 0982, 0983, 0991, 0999, 100, 101, 1031, 1032, 1033, 1041, 1042, 1043, 1044, 1049, 105, 106, 1091, 1099, 110, 111, 1131, 1132, 1139, 1141, 1142, 1143, 1149, 1151, 1152, 1153, 1159, 1171, 1172, 1173, 1174, 1175, 1176, 1179, 1191, 1192, 1199, 9651, 9731 | 0207T, 0402T, 0563T, 13151, 13152, 13153, 15820, 15821, 15822, 15823, 65210, 65270, 65272, 65273, 65275, 65280, 65285, 65286, 65400, 65420, 65426, 65435, 65436, 65450, 65600, 65760, 65765, 65767, 65770, 65771, 65772, 65775, 65778, 65779, 65785, 66250, 67700, 67710, 67715, 67800, 67801, 67805, 67808, 67820, 67825, 67830, 67835, 67840, 67850, 67875, 67880, 67882, 67900, 67901, 67902, 67903, 67904, 67906, 67908, 67909, 67911, 67912, 67914, 67915, 67916, 67917, 67921, 67922, 67923, 67924, 67930, 67935, 67938, 67950, 67961, 67966, 67971, 67973, 67974, 67975, 67999, 68020, 68040, 68110, 68115, 68130, 68135, 68200, 68320, 68325, 68326, 68328, 68330, 68335, 68340, 68360, 68362, 68371, 68399, 68400, 68420, 68440, 68500, 68505, 68520, 68530, 68540, 68550, 68700, 68705, 68720, 68745, 68750, 68760, 68761, 68770, 68801, 68810, 68811, 68815, 68816, 68840, S0800, S0810, S0812 |
| 20 | Other intraocular therapeutic procedures | 1200, 1201, 1202, 1211, 1212, 1213, 1214, 1231, 1232, 1233, 1234, 1235, 1239, 1240, 1241, 1242, 1243, 1244, 1281, 1282, 1283, 1284, 1285, 1286, 1287, 1288, 1289, 1291, 1292, 1293, 1297, 1298, 1299, 1300, 1301, 1302, 1400, 1401, 1402, 146, 1471, 1472, 1473, 1474, 1475, 1479, 1481, 1482, 1483, 149 | 0100T, 0474T, 0616T, 0660T, 0661T, 0699T, 65265, 65810, 65815, 65860, 65865, 65870, 65875, 65880, 65900, 65930, 66020, 66030, 66130, 66225, 66500, 66505, 66600, 66605, 66625, 66630, 66635, 66680, 66682, 66762, 66770, 66990, 66999, 67005, 67010, 67015, 67025, 67027, 67028, 67030, 67031, 67036, 67041, 67042, 67043, 67115, 67120, 67121, 67250, 67255, 67299, C9770 |
| 21 | Other extraocular muscle and orbit therapeutic procedures | 1511, 1512, 1513, 1519, 1521, 1522, 1529, 153, 154, 155, 156, 157, 159, 1601, 1602, 1609, 161, 1631, 1639, 1641, 1642, 1649, 1651, 1652, 1659, 1661, 1662, 1663, 1664, 1665, 1666, 1669, 1671, 1672, 1681, 1682, 1689, 1691, 1692, 1693, 1698, 1699 | 0687T, 0688T, 21280, 21282, 61330, 61333, 65091, 65093, 65101, 65103, 65105, 65110, 65112, 65114, 65125, 65130, 65135, 65140, 65150, 65155, 65175, 65290, 67311, 67312, 67314, 67316, 67318, 67320, 67331, 67332, 67334, 67335, 67340, 67343, 67345, 67399, 67405, 67412, 67413, 67414, 67420, 67430, 67440, 67445, 67500, 67505, 67515, 67550, 67560, 67599, 68841, 68899 |
| 22 | Tympanoplasty | 194, 1952, 1953, 1954, 1955, 196 | 69604, 69610, 69620, 69631, 69632, 69633, 69635, 69636, 69637 |
| 23 | Myringotomy | 2001, 2009 | 0583T, 69420, 69421, 69433, 69436, 69440, S2225 |
| 24 | Mastoidectomy | 2041, 2042, 2049 | 69501, 69502, 69505, 69511, 69530, 69601, 69602, 69603, 69641, 69642, 69643, 69644, 69645, 69646, 69910 |
| 25 | Diagnostic procedures on ear | 1811, 1812, 1819, 2031, 2032, 2039 | 69100, 69105, 92626, 92627, 92640 |
| 26 | Other therapeutic ear procedures | 1801, 1802, 1809, 1821, 1829, 1831, 1839, 184, 185, 186, 1871, 1872, 1879, 189, 190, 1911, 1919, 1921, 1929, 193, 199, 201, 2021, 2022, 2023, 2051, 2059, 2061, 2062, 2071, 2072, 2079, 208, 2091, 2092, 2093, 2094, 2095, 2096, 2097, 2098, 2099, 9611, 9652 | 69000, 69005, 69020, 69090, 69110, 69120, 69140, 69145, 69150, 69155, 69205, 69209, 69210, 69300, 69310, 69320, 69399, 69424, 69450, 69535, 69540, 69550, 69552, 69554, 69650, 69660, 69661, 69662, 69666, 69667, 69670, 69676, 69700, 69705, 69706, 69710, 69711, 69714, 69716, 69717, 69719, 69726, 69727, 69799, 69801, 69805, 69806, 69905, 69915, 69930, 69949, 69950, 69960, 69979, 92630, 92633, G0268, S2230 |
| 27 | Control of epistaxis | 2100, 2101, 2102, 2103, 2104, 2105, 2106, 2107, 2109 | 30901, 30903, 30905, 30906, 30915, 30920, 31238, 31241, 42970 |
| 28 | Plastic procedures on nose | 215, 2181, 2182, 2183, 2184, 2185, 2186, 2187, 2188, 2189 | 30400, 30410, 30420, 30430, 30435, 30450, 30460, 30462, 30465, 30520, 30540, 30545, 30620, 30630 |
| 29 | Oral and Dental Services | 2301, 2309, 2311, 2319, 232, 233, 2341, 2342, 2343, 2349, 235, 236, 2370, 2371, 2372, 2373, 244, 245, 246, 247, 248, 2491, 2499, 9654, 9722, 9733, 9734, 9735, 9997 | 21110, 21497, 41800, 41805, 41806, 41821, 41822, 41823, 41825, 41826, 41827, 41828, 41830, 41850, 41874, 41899 |
| 30 | Tonsillectomy and/or adenoidectomy | 282, 283, 286, 287 | 42820, 42821, 42825, 42826, 42830, 42831, 42835, 42836, 42860, 42870 |
| 31 | Diagnostic procedures on nose, mouth and pharynx | 2121, 2122, 2129, 2211, 2212, 2219, 2411, 2412, 2419, 2501, 2502, 2509, 2611, 2612, 2619, 2721, 2722, 2723, 2724, 2729, 2811, 2819, 2911, 2912, 2919 | 30100, 31231, 31233, 31235, 31237, 40490, 40808, 41100, 41105, 41108, 42100, 42400, 42405, 42800, 42804, 42806, 42975, 92502, 92504, 92511, 92512, 92610, 92611, 92612, 92613, 92614, 92615, 92616, 92617, 92700 |
| 32 | Other non-OR therapeutic procedures on nose, mouth and pharynx | 211, 2130, 2131, 2132, 2171, 2191, 2200, 2201, 2202, 222, 240, 2431, 2432, 2439, 2551, 2591, 2592, 2593, 260, 2691, 2741, 2751, 2752, 2791, 2991, 9621, 9653, 9721, 9732 | 21076, 21077, 21079, 21080, 21081, 21082, 21083, 21084, 21085, 21086, 21087, 21088, 21089, 30020, 30118, 30200, 30210, 30220, 30310, 30801, 30802, 30999, 31000, 31002, 31299, 40799, 40806, 40820, 40830, 40831, 40899, 41010, 41115, 41250, 41251, 41252, 41530, 41599, 42000, 42160, 42280, 42299, 42650, 42660, 42699, 42960, 42999, 92526, 99188 |
| 33 | Other OR therapeutic procedures on nose, mouth and pharynx | 214, 2161, 2162, 2169, 2172, 2199, 2231, 2239, 2241, 2242, 2250, 2251, 2252, 2253, 2260, 2261, 2262, 2263, 2264, 2271, 2279, 229, 242, 251, 252, 253, 254, 2559, 2594, 2599, 2621, 2629, 2630, 2631, 2632, 2641, 2642, 2649, 2699, 270, 271, 2731, 2732, 2742, 2743, 2749, 2753, 2754, 2755, 2756, 2757, 2759, 2761, 2762, 2763, 2764, 2769, 2771, 2772, 2773, 2779, 2792, 2799, 280, 284, 285, 2891, 2892, 2899, 290, 292, 293, 2931, 2932, 2933, 2939, 294, 2951, 2952, 2953, 2954, 2959, 2992, 2999 | 20912, 21100, 30000, 30110, 30115, 30117, 30125, 30130, 30140, 30150, 30160, 30468, 30560, 30580, 30600, 31020, 31030, 31032, 31040, 31050, 31051, 31070, 31075, 31080, 31081, 31084, 31085, 31086, 31087, 31090, 31200, 31201, 31205, 31239, 31240, 31253, 31254, 31255, 31256, 31257, 31259, 31267, 31276, 31287, 31288, 31290, 31291, 31292, 31293, 31294, 31295, 31296, 31297, 31298, 31390, 31395, 40500, 40510, 40520, 40525, 40527, 40530, 40650, 40652, 40654, 40700, 40701, 40702, 40720, 40761, 40800, 40801, 40810, 40812, 40814, 40816, 40819, 40840, 40842, 40843, 40844, 40845, 41000, 41005, 41006, 41007, 41008, 41009, 41015, 41016, 41017, 41018, 41110, 41112, 41113, 41114, 41116, 41120, 41130, 41135, 41140, 41145, 41150, 41153, 41155, 41510, 41512, 41520, 41820, 41870, 41872, 42104, 42106, 42107, 42120, 42140, 42145, 42180, 42182, 42200, 42205, 42210, 42215, 42220, 42225, 42226, 42227, 42235, 42260, 42281, 42300, 42305, 42310, 42320, 42330, 42335, 42340, 42408, 42409, 42410, 42415, 42420, 42425, 42426, 42440, 42450, 42500, 42505, 42507, 42509, 42510, 42600, 42665, 42700, 42720, 42725, 42808, 42810, 42815, 42842, 42844, 42845, 42890, 42892, 42894, 42900, 42950, 42953, 42955, 42961, 42962, 42971, 42972, 43030, C9771, S2080, S2342 |
| 34 | Tracheostomy, temporary and permanent | 311, 3121, 3129 | 31600, 31601, 31603, 31605, 31610, 31613, 31614 |
| 35 | Tracheoscopy and laryngoscopy with biopsy | 3142, 3143, 3144 | 31505, 31510, 31515, 31520, 31525, 31526, 31535, 31536, 31575, 31576, 31579, 31615 |
| 36 | Lobectomy or pneumonectomy | 3220, 3221, 3222, 3223, 3224, 3225, 3226, 3227, 3229, 323, 3230, 3239, 324, 3241, 3249, 325, 3250, 3259 | 32440, 32442, 32445, 32480, 32482, 32484, 32486, 32488, 32491, 32501, 32505, 32506, 32663, 32666, 32667, 32668, 32669, 32670, 32671, 32672, 32850, G0302, G0303, G0304, G0305, S2061 |
| 37 | Diagnostic bronchoscopy and biopsy of bronchus | 3322, 3323, 3324, 3326, 3327, 3372 | 31622, 31623, 31624, 31625, 31626, 31627, 31628, 31629, 31632, 31633, 31652, 31653, 31717 |
| 38 | Other diagnostic procedures on lung and bronchus | 3320, 3325, 3328, 3329, 3402 | 32096, 32097, 32098, 32100, 32408, 32507, 32607, 32608, 32609, 94010, 94011, 94012, 94013, 94014, 94015, 94016, 94060, 94070, 94150, 94200, 94375, 94450, 94452, 94453, 94617, 94618, 94619, 94621, 94726, 94727, 94728, 94729 |
| 39 | Incision of pleura, thoracentesis, chest drainage | 3404, 3406, 3409, 3491 | 32035, 32036, 32550, 32551, 32554, 32555, 32556, 32557 |
| 40 | Other diagnostic procedures of respiratory tract and mediastinum | 3141, 3145, 3148, 3149, 3321, 3420, 3421, 3422, 3423, 3424, 3425, 3426, 3427, 3428, 3429 | 32400, 32601, 32606, 39401, 39402, 92520, 95012 |
| 41 | Other non-OR therapeutic procedures on respiratory system | 310, 3193, 3194, 3195, 3201, 3228, 3331, 3332, 3333, 3371, 3373, 3378, 3379, 3391, 3471, 3472, 3492, 9655, 9656, 9723, 9737, 9741, 9742, 9743, 9749 | 0683T, 0684T, 0685T, 31502, 31512, 31513, 31572, 31573, 31574, 31578, 31599, 31612, 31647, 31648, 31649, 31651, 31720, 31725, 31899, 32552, 32553, 32560, 32561, 32562, 32701, 32960, 32997, 32999, 39499, 39599, 94610, 94799 |
| 42 | Other OR therapeutic procedures on respiratory system | 3001, 3009, 301, 3021, 3022, 3029, 303, 304, 313, 315, 3161, 3162, 3163, 3164, 3169, 3171, 3172, 3173, 3174, 3175, 3179, 3191, 3192, 3198, 3199, 320, 3209, 321, 326, 329, 330, 331, 3334, 3339, 3341, 3342, 3343, 3348, 3349, 3392, 3393, 3398, 3399, 3401, 3403, 3405, 341, 343, 344, 3451, 3452, 3459, 346, 3473, 3474, 3479, 3481, 3482, 3483, 3484, 3485, 3489, 3493, 3499 | 0674T, 0675T, 0676T, 0677T, 0678T, 0679T, 0680T, 0681T, 0682T, 31300, 31360, 31365, 31367, 31368, 31370, 31375, 31380, 31382, 31400, 31420, 31527, 31528, 31529, 31530, 31531, 31540, 31541, 31545, 31546, 31551, 31552, 31553, 31554, 31560, 31561, 31570, 31571, 31580, 31584, 31587, 31590, 31591, 31592, 31611, 31630, 31631, 31634, 31636, 31637, 31638, 31640, 31641, 31643, 31645, 31646, 31660, 31661, 31730, 31750, 31755, 31760, 31766, 31770, 31775, 31780, 31781, 31785, 31786, 31800, 31805, 31820, 31825, 32110, 32120, 32124, 32140, 32141, 32150, 32151, 32200, 32215, 32220, 32225, 32310, 32320, 32503, 32504, 32540, 32650, 32651, 32652, 32653, 32654, 32655, 32656, 32662, 32800, 32810, 32815, 32820, 32905, 32906, 32940, 32994, 32998, 39000, 39010, 39200, 39220, 39501, 39545, 39560, 39561, C9751, S2340, S2341 |
| 43 | Heart valve procedures | 3500, 3501, 3502, 3503, 3504, 3505, 3506, 3507, 3508, 3509, 3510, 3511, 3512, 3513, 3514, 3520, 3521, 3522, 3523, 3524, 3525, 3526, 3527, 3528, 3596, 3597, 3599 | 0345T, 0483T, 0484T, 0543T, 0544T, 0545T, 0569T, 0570T, 0646T, 33361, 33362, 33363, 33364, 33365, 33366, 33367, 33368, 33369, 33370, 33390, 33391, 33404, 33405, 33406, 33410, 33411, 33412, 33413, 33414, 33415, 33416, 33417, 33418, 33419, 33420, 33422, 33425, 33426, 33427, 33430, 33440, 33460, 33463, 33464, 33465, 33468, 33471, 33474, 33475, 33476, 33477, 33478, 33496, 33600, 33602, 33684, 33732, 33920, 92986, 92987, 92990, 93590, 93591, 93592 |
| 44 | Coronary artery bypass graft (CABG) | 3610, 3611, 3612, 3613, 3614, 3615, 3616, 3617, 3619, 362, 363, 3631, 3632, 3633, 3634, 3639 | 33140, 33141, 33510, 33511, 33512, 33513, 33514, 33516, 33517, 33518, 33519, 33521, 33522, 33523, 33530, 33533, 33534, 33535, 33536, 35600, S2205, S2206, S2207, S2208, S2209 |
| 45 | Percutaneous transluminal coronary angioplasty (PTCA) | 0066, 1755, 3601, 3602, 3605 | 92920, 92921, 92924, 92925, 92928, 92929, 92933, 92934, 92937, 92938, 92941, 92943, 92944, 92973, C9600, C9601, C9602, C9603, C9604, C9605, C9606, C9607, C9608 |
| 46 | Coronary thrombolysis | 3604 | 92975, 92977 |
| 47 | Diagnostic cardiac catheterization, coronary arteriography | 3721, 3722, 3723, 8852, 8853, 8854, 8855, 8856, 8857 | 0523T, 36013, 75574, 75756, 93451, 93452, 93453, 93454, 93455, 93456, 93457, 93458, 93459, 93460, 93461, 93462, 93463, 93464, 93563, 93564, 93565, 93566, 93567, 93568, 93571, 93572, 93593, 93594, 93595, 93596, 93597, 93598 |
| 48 | Insertion, revision, replacement, removal of cardiac pacemaker or cardioverter/defibrillator | 0050, 0051, 0052, 0053, 0054, 0056, 0057, 1751, 1752, 3770, 3771, 3772, 3773, 3774, 3775, 3776, 3777, 3778, 3779, 3780, 3781, 3782, 3783, 3785, 3786, 3787, 3789, 3794, 3795, 3796, 3797, 3798 | 0408T, 0409T, 0410T, 0411T, 0412T, 0413T, 0414T, 0415T, 0416T, 0515T, 0516T, 0517T, 0518T, 0519T, 0520T, 0571T, 0572T, 0573T, 0574T, 0580T, 0614T, 33202, 33203, 33206, 33207, 33208, 33210, 33211, 33212, 33213, 33214, 33215, 33216, 33217, 33218, 33220, 33221, 33222, 33223, 33224, 33225, 33226, 33227, 33228, 33229, 33230, 33231, 33233, 33234, 33235, 33236, 33237, 33238, 33240, 33241, 33243, 33244, 33249, 33262, 33263, 33264, 33270, 33271, 33272, 33273, 33274, 33275, G0448 |
| 49 | Other OR heart procedures | 3531, 3532, 3533, 3534, 3535, 3539, 3541, 3542, 3550, 3551, 3552, 3553, 3554, 3555, 3560, 3561, 3562, 3563, 3570, 3571, 3572, 3573, 3581, 3582, 3583, 3584, 3591, 3592, 3593, 3594, 3595, 3598, 3600, 3603, 3609, 3691, 3699, 3710, 3711, 3712, 3731, 3732, 3733, 3734, 3735, 3736, 3737, 374, 3741, 3749, 3752, 3753, 3754, 3755, 3760, 3761, 3762, 3763, 3764, 3765, 3766, 3767, 3768, 3790, 3791, 3799 | 0525T, 0526T, 0527T, 0530T, 0531T, 0532T, 0613T, 0643T, 0644T, 32160, 32658, 32659, 32661, 33017, 33018, 33019, 33020, 33025, 33030, 33031, 33050, 33120, 33130, 33250, 33251, 33254, 33255, 33256, 33257, 33258, 33259, 33261, 33265, 33266, 33267, 33268, 33269, 33300, 33305, 33310, 33315, 33340, 33500, 33501, 33502, 33503, 33504, 33505, 33506, 33507, 33542, 33545, 33548, 33572, 33608, 33610, 33611, 33612, 33615, 33617, 33619, 33620, 33621, 33622, 33641, 33645, 33647, 33660, 33665, 33670, 33675, 33676, 33677, 33681, 33688, 33692, 33694, 33697, 33702, 33710, 33720, 33724, 33726, 33730, 33735, 33736, 33737, 33741, 33745, 33746, 33770, 33771, 33774, 33775, 33776, 33777, 33778, 33779, 33780, 33781, 33782, 33783, 33786, 33813, 33814, 33894, 33895, 33924, 33927, 33928, 33929, 33940, 33967, 33970, 33971, 33973, 33974, 33975, 33976, 33977, 33978, 33979, 33980, 33981, 33982, 33983, 33999, 93580, 93581, 93582, 93583, C9758, C9760, C9782, C9783 |
| 50 | Extracorporeal circulation auxiliary to open heart procedures | 3961, 3962, 3963, 3964, 3965, 3966 | 33946, 33947, 33948, 33949, 33951, 33952, 33953, 33954, 33955, 33956, 33957, 33958, 33959, 33962, 33963, 33964, 33965, 33966, 33969, 33984, 33985, 33986, 33987, 33988, 33989, 33990, 33991, 33992, 33993, 33995, 33997, 92970, 92971 |
| 51 | Endarterectomy, vessel of head and neck | 3811, 3812 | 35301, 35390, 35901 |
| 52 | Aortic resection, replacement or anastomosis | 3834, 3844, 3864, 3971, 3973, 3978 | 33330, 33335, 33606, 33802, 33803, 33820, 33822, 33824, 33840, 33845, 33851, 33852, 33853, 33858, 33859, 33863, 33864, 33866, 33871, 33875, 33877, 33880, 33881, 33883, 33884, 33886, 34705, 34830, 34831, 34832, 34839, 35081, 35082, 35091, 35092, 35102, 35103 |
| 53 | Varicose vein stripping, lower limb | 3859 | 37700, 37718, 37722, 37735, 37760, 37761, 37765, 37766, 37780, 37785 |
| 54 | Other vascular catheterization, not heart | 3891, 3892, 3893, 3897 | 36000, 36010, 36011, 36012, 36014, 36015, 36100, 36140, 36160, 36200, 36215, 36216, 36217, 36218, 36221, 36222, 36223, 36224, 36225, 36226, 36227, 36228, 36245, 36246, 36247, 36248, 36251, 36252, 36253, 36254, 36481, 36500, 36510, 36555, 36556, 36557, 36558, 36560, 36561, 36563, 36565, 36566, 36568, 36569, 36570, 36571, 36572, 36573, 36578, 36580, 36581, 36582, 36583, 36584, 36585, 36595, 36597, 36620, 36625, 36640, 36660, 36823, 37197, 37211, 61650, 61651, 92997, 92998, C9780, G0278, S2095 |
| 55 | Peripheral vascular bypass | 3925, 3929 | 34520, 35516, 35518, 35521, 35522, 35523, 35525, 35533, 35537, 35538, 35539, 35540, 35556, 35558, 35565, 35566, 35570, 35571, 35583, 35585, 35587, 35616, 35621, 35623, 35637, 35638, 35646, 35647, 35650, 35654, 35656, 35661, 35665, 35666, 35671, 35685, 35686 |
| 56 | Other vascular bypass and shunt, not heart | 390, 391, 3921, 3922, 3923, 3924, 3926, 3928 | 33750, 33755, 33762, 33764, 33766, 33767, 33768, 35501, 35506, 35508, 35509, 35510, 35511, 35512, 35515, 35526, 35531, 35535, 35536, 35560, 35563, 35601, 35606, 35612, 35626, 35631, 35632, 35633, 35634, 35636, 35642, 35645, 35663, 37140, 37145, 37160, 37180, 37181, 37182, 37183 |
| 57 | Creation, revision and removal of arteriovenous fistula or vessel-to-vessel cannula for dialysis | 3927, 3942, 3943, 3993 | 36800, 36810, 36815, 36818, 36819, 36820, 36821, 36825, 36830, 36832, 36833, 36835, 36838, 36901, 36902, 36903, 36904, 36905, 36906, 36907, 36908, 36909 |
| 58 | Hemodialysis | 3995 | 90935, 90937, 90940, G0257, G0491, G0492 |
| 59 | Other OR procedures on vessels of head and neck | 0061, 0062, 0063, 0064, 0065, 1753, 1754, 3801, 3802, 3831, 3832, 3841, 3842, 3851, 3852, 3861, 3862, 3881, 3882, 3972, 3974, 3975, 3976, 3981, 3982, 3983, 3984, 3985, 3986, 3987, 3988, 3989 | 0075T, 0076T, 0266T, 0267T, 0268T, 0269T, 0270T, 0271T, 33889, 33891, 34001, 34471, 35001, 35002, 35005, 35180, 35188, 35231, 35261, 35691, 35693, 35694, 35695, 35697, 35701, 35800, 37215, 37216, 37565, 37600, 37605, 37606, 37609, 37615, 60600, 60605, 61611, 61613, 61623, 61624, 61626, 61630, 61635, 61640, 61641, 61642, 61645, 61680, 61682, 61684, 61686, 61690, 61692, 61697, 61698, 61700, 61702, 61703, 61705, 61708, 61710, 61711 |
| 60 | Embolectomy and endarterectomy of lower limbs | 3808, 3818 | 34201, 34203, 34421, 34451, 35302, 35303, 35304, 35305, 35306, 35355, 35361, 35363, 35371, 35372, 35700, 35903 |
| 61 | Other OR procedures on vessels other than head and neck | 0040, 0041, 0042, 0043, 0044, 0045, 0046, 0047, 0048, 0055, 1756, 1771, 3800, 3803, 3804, 3805, 3806, 3807, 3809, 3810, 3813, 3814, 3815, 3816, 3830, 3833, 3835, 3836, 3837, 3838, 3839, 3840, 3843, 3845, 3846, 3847, 3848, 3849, 3850, 3853, 3855, 3857, 3860, 3863, 3865, 3866, 3867, 3868, 3869, 387, 3880, 3883, 3884, 3885, 3886, 3887, 3888, 3889, 3930, 3931, 3932, 3941, 3949, 3950, 3951, 3952, 3953, 3954, 3955, 3956, 3957, 3958, 3959, 397, 3977, 3979, 398, 3990, 3991, 3992, 3994, 3998, 3999, 6824, 6825 | 0234T, 0235T, 0236T, 0237T, 0238T, 0505T, 0524T, 0620T, 33320, 33321, 33322, 33508, 33509, 33690, 33788, 33800, 33897, 33910, 33915, 33916, 33917, 33922, 33925, 33926, 34051, 34101, 34111, 34151, 34401, 34490, 34501, 34502, 34510, 34530, 34701, 34702, 34703, 34704, 34706, 34707, 34708, 34709, 34710, 34711, 34712, 34713, 34714, 34715, 34716, 34717, 34718, 34808, 34812, 34813, 34820, 34833, 34834, 34841, 34842, 34843, 34844, 34845, 34846, 34847, 34848, 35011, 35013, 35021, 35022, 35045, 35111, 35112, 35121, 35122, 35131, 35132, 35141, 35142, 35151, 35152, 35182, 35184, 35189, 35190, 35201, 35206, 35207, 35211, 35216, 35221, 35226, 35236, 35241, 35246, 35251, 35256, 35266, 35271, 35276, 35281, 35286, 35311, 35321, 35331, 35341, 35351, 35500, 35572, 35681, 35682, 35683, 35702, 35703, 35820, 35840, 35860, 35870, 35875, 35876, 35879, 35881, 35883, 35884, 35905, 35907, 36260, 36261, 36262, 36483, 36831, 37184, 37185, 37186, 37187, 37188, 37191, 37192, 37193, 37217, 37218, 37220, 37221, 37222, 37223, 37224, 37225, 37226, 37227, 37228, 37229, 37230, 37231, 37232, 37233, 37234, 37235, 37236, 37237, 37238, 37239, 37241, 37242, 37243, 37244, 37246, 37247, 37248, 37249, 37500, 37607, 37616, 37617, 37618, 37619, 37650, 37660, 37788, 37790, 50100, C9764, C9765, C9766, C9767, C9772, C9773, C9774, C9775, G0269, G2170, G2171, M0301 |
| 62 | Other diagnostic cardiovascular procedures | 3720, 3724, 3725, 3726, 3727, 3728, 3729, 3821, 3822, 3826, 3829 | 0104U, 0272T, 0475T, 0476T, 0477T, 0478T, 0541T, 0542T, 0577T, 0623T, 0624T, 0625T, 0626T, 32604, 33285, 33286, 33289, 35400, 36005, 37200, 37501, 93024, 93050, 93260, 93261, 93505, 93600, 93602, 93603, 93609, 93610, 93612, 93613, 93615, 93616, 93618, 93619, 93620, 93621, 93622, 93623, 93624, 93631, 93640, 93641, 93642, 93644, 93701, 93740, 93750, 93770, 93784, 93786, 93788, 93790, 93799, 93922, 93923, 93924, 93998, S3902 |
| 63 | Other non-OR therapeutic cardiovascular procedures | 0049, 0060, 3606, 3607, 370, 3792, 3793, 3894, 3895, 3996, 3997, 9744 | 0273T, 0553T, 0632T, 0645T, 0659T, 33016, 33968, 36002, 36420, 36425, 36465, 36466, 36468, 36470, 36471, 36473, 36474, 36475, 36476, 36478, 36479, 36482, 36575, 36576, 36589, 36590, 36591, 36592, 36593, 36596, 36860, 36861, 37195, 37212, 37213, 37214, 37799, 92950, 92953, 92960, C9759, G0166, M0300 |
| 64 | Bone marrow transplant | 410, 4100, 4101, 4102, 4103, 4104, 4105, 4106, 4107, 4108, 4109 | 38205, 38206, 38207, 38208, 38209, 38210, 38211, 38212, 38213, 38214, 38215, 38240, 38241, 38242, 38243, S2140 |
| 65 | Bone marrow biopsy | 4131 | 38220, 38221, 38222 |
| 66 | Procedures on spleen | 411, 412, 4141, 4142, 4143, 415, 4193, 4195, 4199 | 38100, 38101, 38102, 38115, 38120, 38129 |
| 67 | Other therapeutic procedures, hemic and lymphatic system | 400, 4011, 4019, 4021, 4022, 4023, 4024, 4029, 403, 4040, 4041, 4042, 4050, 4051, 4052, 4053, 4054, 4059, 4061, 4062, 4063, 4064, 4069, 409, 4132, 4133, 4138, 4139, 4191, 4192, 4198 | 0265T, 32674, 36680, 38204, 38230, 38232, 38300, 38305, 38308, 38380, 38381, 38382, 38500, 38505, 38510, 38520, 38525, 38530, 38531, 38542, 38550, 38555, 38562, 38564, 38570, 38571, 38572, 38573, 38589, 38700, 38720, 38724, 38740, 38745, 38746, 38747, 38760, 38765, 38770, 38780, 38794, 38900, 38999, 49062, 49323, 92974 |
| 68 | Injection or ligation of esophageal varices | 4291 | 43243, 43244, 43400 |
| 69 | Esophageal dilatation | 4292 | 43195, 43196, 43213, 43214, 43220, 43226, 43233, 43248, 43249, 43450, 43453, 43460, 43510 |
| 70 | Upper gastrointestinal endoscopy, biopsy | 4223, 4224, 4413, 4414, 4513, 4514, 4516 | 0652T, 0653T, 0654T, 43180, 43191, 43192, 43193, 43197, 43198, 43200, 43201, 43202, 43204, 43205, 43206, 43210, 43211, 43212, 43217, 43229, 43231, 43232, 43235, 43236, 43237, 43238, 43239, 43240, 43241, 43242, 43247, 43250, 43251, 43252, 43253, 43254, 43255, 43257, 43259, 43266, 43270, 44360, 44361, 44376, 44377, 44379, 44380, 44381, 44382, 44384, 44385, 44386 |
| 71 | Gastrostomy, temporary and permanent | 431, 4311, 4319, 432, 4432 | 0647T, 43246, 43653, 43762, 43763, 43830, 43831, 43832, 44373, 49440, 49450, 49452 |
| 72 | Colostomy, temporary and permanent | 4610, 4611, 4612, 4613, 4614 | 44188, 44320, 44322, 44340, 44345, 44605, 45563, 45805, 45825, 57307 |
| 73 | Ileostomy and other enterostomy | 4620, 4621, 4622, 4623, 4624, 4631, 4632, 4639 | 44015, 44130, 44186, 44187, 44300, 44310, 44312, 44314, 44316, 47802, 48001, 48150, 48152, 48153, 48154, 49441, 49442, 49446, 49451 |
| 74 | Gastrectomy, partial and total | 435, 436, 437, 4381, 4382, 4389, 4391, 4399 | 43620, 43621, 43622, 43631, 43632, 43633, 43634 |
| 75 | Small bowel resection | 4561, 4562, 4563 | 43496, 44120, 44121, 44125, 44126, 44127, 44128, 44132, 44133, 44202, 44203, 44661 |
| 76 | Colonoscopy and biopsy | 4523, 4524, 4525 | 44388, 44389, 44391, 44392, 44394, 44401, 44402, 44403, 44404, 44405, 44406, 44407, 44408, 45378, 45380, 45381, 45382, 45384, 45385, 45386, 45388, 45389, 45390, 45391, 45392, 45393, 45398, 45500, 45505, C1749, G0105, G0121 |
| 77 | Proctoscopy and anorectal biopsy | 4823, 4824, 4826, 4921, 4922, 4923 | 45100, 45300, 45303, 45305, 45308, 45309, 45315, 45317, 45320, 45321, 45327, 45330, 45331, 45333, 45334, 45335, 45337, 45338, 45340, 45341, 45342, 45346, 45347, 45349, 45350, 46600, 46601, 46604, 46606, 46607, 46610, 46611, 46612, 46614, 46615, G0104, S0601 |
| 78 | Colorectal resection | 1731, 1732, 1733, 1734, 1735, 1736, 1739, 4571, 4572, 4573, 4574, 4575, 4576, 4579, 458, 4581, 4582, 4583, 4840, 4841, 4842, 4843, 4849, 485, 4850, 4851, 4852, 4859, 4861, 4862, 4863, 4864, 4865, 4866, 4869 | 44139, 44140, 44141, 44143, 44144, 44145, 44146, 44147, 44150, 44151, 44155, 44156, 44157, 44158, 44160, 44204, 44205, 44206, 44207, 44208, 44210, 44211, 44212, 44213, 45110, 45111, 45112, 45113, 45114, 45116, 45119, 45120, 45121, 45123, 45126, 45130, 45135, 45136, 45160, 45171, 45172, 45395, 45397, 45402, 45550 |
| 79 | Local excision of large intestine lesion (not endoscopic) | 4541 | 44110, 44111 |
| 80 | Appendectomy | 470, 4701, 4709, 471, 4711, 4719 | 44950, 44955, 44960, 44970, 44979 |
| 81 | Hemorrhoid procedures | 4941, 4942, 4943, 4944, 4945, 4946, 4947, 4949 | 46083, 46221, 46230, 46250, 46255, 46257, 46258, 46260, 46261, 46262, 46320, 46500, 46930, 46945, 46946, 46947, 46948 |
| 82 | Endoscopic retrograde cannulation of pancreas (ERCP) | 5110, 5111, 5197, 5213, 5291 | 0397T, 43260, 43261, 43262, 43263, 43264, 43265, 43273, 43274, 43275, 43276, 43277, 43278, 74328, 74329, 74330 |
| 83 | Biopsy of liver | 5011, 5012, 5013 | 47000, 47001, 47100 |
| 84 | Cholecystectomy and common duct exploration | 5121, 5122, 5123, 5124, 5141, 5142, 5143, 5149, 5151, 5159 | 47400, 47420, 47425, 47460, 47550, 47562, 47563, 47564, 47600, 47605, 47610, 47612, 47620 |
| 85 | Inguinal and femoral hernia repair | 1711, 1712, 1713, 1721, 1722, 1723, 1724, 5300, 5301, 5302, 5303, 5304, 5305, 5310, 5311, 5312, 5313, 5314, 5315, 5316, 5317, 5321, 5329, 5331, 5339 | 49491, 49492, 49495, 49496, 49500, 49501, 49505, 49507, 49520, 49521, 49525, 49550, 49553, 49555, 49557, 49650, 49651 |
| 86 | Other hernia repair | 5341, 5342, 5343, 5349, 5351, 5359, 5361, 5362, 5363, 5369, 537, 5371, 5372, 5375, 5380, 5381, 5382, 5383, 5384, 539 | 39503, 39540, 39541, 43281, 43282, 43332, 43333, 43334, 43335, 43336, 43337, 44346, 49540, 49560, 49561, 49565, 49566, 49568, 49570, 49572, 49580, 49582, 49585, 49587, 49590, 49600, 49605, 49606, 49610, 49611, 49652, 49653, 49654, 49655, 49656, 49657, 49659, 55540 |
| 87 | Laparoscopy | 5421 | 43289, 43659, 44238, 45499, 47579, 49320, 49321, 49322, 49324, 49325, 49326, 49327, 49329 |
| 88 | Abdominal paracentesis | 5491 | 49082, 49083, 49084 |
| 89 | Exploratory laparotomy | 5411 | 43605, 49000, 49002, 49010, 49013, 49014 |
| 90 | Excision, lysis peritoneal adhesions | 545, 5451, 5459 | 44005 |
| 91 | Peritoneal dialysis | 5498 | 90945, 90947, 90997, 90999 |
| 92 | Other bowel diagnostic procedures | 4411, 4412, 4415, 4419, 4511, 4512, 4515, 4519, 4521, 4522, 4526, 4527, 4528, 4529, 4821, 4822, 4825, 4829, 4929 | 44010, 44020, 44025, 44100 |
| 93 | Other non-OR upper GI therapeutic procedures | 4233, 4281, 4341, 4422, 4443, 4444, 4449, 4462, 4493, 4494, 9606, 9631, 9632, 9633, 9634, 9635, 9701, 9702, 9751 | 43216, 43227, 43245, 44378, M0100, S2083 |
| 94 | Other OR upper GI therapeutic procedures | 4201, 4209, 4210, 4211, 4212, 4219, 4231, 4232, 4239, 4240, 4241, 4242, 4251, 4252, 4253, 4254, 4255, 4256, 4258, 4259, 4261, 4262, 4263, 4264, 4265, 4266, 4268, 4269, 427, 4282, 4283, 4284, 4285, 4286, 4287, 4289, 4299, 430, 433, 4342, 4349, 4400, 4401, 4402, 4403, 442, 4421, 4429, 4431, 4438, 4439, 4440, 4441, 4442, 445, 4461, 4463, 4464, 4465, 4466, 4467, 4468, 4469, 4491, 4492, 4495, 4496, 4497, 4498, 4499 | 32665, 43020, 43045, 43100, 43101, 43107, 43108, 43112, 43113, 43116, 43117, 43118, 43121, 43122, 43123, 43124, 43130, 43135, 43279, 43280, 43283, 43284, 43285, 43286, 43287, 43288, 43300, 43305, 43310, 43312, 43313, 43314, 43320, 43325, 43327, 43328, 43330, 43331, 43338, 43340, 43341, 43351, 43352, 43360, 43361, 43405, 43410, 43415, 43420, 43425, 43497, 43500, 43501, 43502, 43520, 43610, 43611, 43635, 43640, 43641, 43647, 43648, 43651, 43652, 43800, 43810, 43820, 43825, 43840, 43860, 43865, 43870, 43880, 43881, 43882, 44615, 46924, S2079 |
| 95 | Other non-OR lower GI therapeutic procedures | 4530, 4542, 4543, 4685, 4686, 4687, 4695, 4696, 4831, 4832, 4833, 4834, 4836, 4903, 4931, 9609, 9619, 9622, 9623, 9626, 9629, 9637, 9638, 9639, 9703, 9704, 9752, 9753, 9993 | 43499, 43761, 43999, 44705, 44799, 44899, 45190, 45399, 45900, 45905, 45910, 45915, 45999, 46220, 46505, 46706, 46900, 46910, 46916, 46917, 46922, 46940, 46942, 46999, G0455 |
| 96 | Other OR lower GI therapeutic procedures | 4500, 4501, 4502, 4503, 4531, 4532, 4533, 4534, 4549, 4550, 4551, 4552, 4590, 4591, 4592, 4593, 4594, 4595, 4601, 4602, 4603, 4604, 4640, 4641, 4642, 4643, 4650, 4651, 4652, 4660, 4661, 4662, 4663, 4664, 4671, 4672, 4673, 4674, 4675, 4676, 4679, 4680, 4681, 4682, 4691, 4692, 4693, 4694, 4699, 472, 4791, 4792, 4799, 480, 481, 4835, 4871, 4872, 4873, 4874, 4875, 4876, 4879, 4881, 4882, 4891, 4892, 4893, 4899, 4901, 4902, 4904, 4911, 4912, 493, 4939, 4951, 4952, 4959, 496, 4971, 4972, 4973, 4974, 4975, 4976, 4979, 4991, 4992, 4993, 4994, 4995, 4999 | 0184T, 44021, 44050, 44055, 44180, 44227, 44370, 44372, 44602, 44603, 44604, 44620, 44625, 44626, 44640, 44650, 44660, 44680, 44700, 44701, 44800, 44820, 44850, 44900, 45000, 45005, 45020, 45108, 45150, 45400, 45540, 45541, 45562, 45800, 45820, 46020, 46030, 46040, 46045, 46050, 46060, 46070, 46080, 46200, 46270, 46275, 46280, 46285, 46288, 46700, 46705, 46707, 46710, 46712, 46715, 46716, 46730, 46735, 46740, 46742, 46750, 46751, 46753, 46754, 46760, 46761 |
| 97 | Other gastrointestinal diagnostic procedures | 4221, 4222, 4225, 4229, 5014, 5019, 5112, 5113, 5114, 5115, 5119, 5211, 5212, 5214, 5219, 5422, 5423, 5424, 5425, 5429 | 0651T, 45990, 47543, 47552, 47553, 47700, 48100, 48102, 48400, 49180, 49400, 49424, 49427, 91010, 91013, 91020, 91022, 91030, 91034, 91035, 91037, 91038, 91040, 91065, 91112, 91113, 91117, 91120, 91122, 91132, 91133, 91200, 91299, C9768, C9777 |
| 98 | Other non-OR gastrointestinal therapeutic procedures | 5091, 5092, 5093, 5094, 5099, 5101, 5164, 5184, 5185, 5186, 5187, 5188, 5196, 5198, 5221, 5293, 5294, 5297, 5298, 5496, 5497, 5499, 9608, 9624, 9627, 9628, 9636, 9641, 9642, 9643, 9705, 9754, 9755, 9756, 9759, 9782, 9786 | 0686T, 44500, 45520, 47133, 47399, 47536, 47537, 47541, 47999, 48550, 48999, 49405, 49406, 49407, 49411, 49423, 49460, 49999, 91110, 91111, 99175 |
| 99 | Other OR gastrointestinal therapeutic procedures | 1763, 500, 5021, 5022, 5023, 5024, 5025, 5026, 5029, 503, 504, 5061, 5069, 5102, 5103, 5104, 5131, 5132, 5133, 5134, 5135, 5136, 5137, 5139, 5161, 5162, 5163, 5169, 5171, 5172, 5179, 5181, 5182, 5183, 5189, 5191, 5192, 5193, 5194, 5195, 5199, 5201, 5209, 522, 5222, 523, 524, 5251, 5252, 5253, 5259, 526, 527, 5292, 5295, 5296, 5299, 540, 5412, 5419, 543, 544, 5461, 5462, 5463, 5464, 5471, 5472, 5473, 5474, 5475, 5492, 5493, 5494, 5495 | 0437T, 44137, 44364, 44365, 44366, 44369, 47010, 47015, 47120, 47122, 47125, 47130, 47300, 47350, 47360, 47361, 47362, 47370, 47371, 47379, 47380, 47381, 47382, 47383, 47480, 47490, 47533, 47534, 47535, 47538, 47539, 47540, 47542, 47544, 47554, 47555, 47556, 47570, 47701, 47711, 47712, 47715, 47720, 47721, 47740, 47741, 47760, 47765, 47780, 47785, 47800, 47801, 47900, 48000, 48020, 48105, 48120, 48140, 48145, 48146, 48148, 48155, 48500, 48510, 48520, 48540, 48545, 48547, 48548, 48556, 49020, 49040, 49060, 49185, 49203, 49204, 49205, 49215, 49250, 49255, 49402, 49412, 49418, 49419, 49421, 49422, 49425, 49426, 49428, 49429, 49435, 49436, 49900, 49904, 49905, 49906, C9779 |
| 100 | Endoscopy and endoscopic biopsy of the urinary tract | 5521, 5522, 5631, 5633, 5732, 5733, 5822, 5823 | 50551, 50553, 50555, 50570, 50574, 50951, 50955, 50970, 50974, 52000, 52007, 52010, 52204, 52276, 52351, 52354, 53200, C9738 |
| 101 | Transurethral excision, drainage, or removal urinary obstruction | 560, 570, 5741, 5749 | 50561, 50580, 50957, 50961, 50976, 50980, 52001, 52214, 52224, 52234, 52235, 52240, 52300, 52301, 52305, 52310, 52315, 52320, 52325, 52330, 52341, 52342, 52343, 52344, 52345, 52346, 52352, 52500, 52640, S2070 |
| 102 | Ureteral catheterization | 598 | 50572, 50605, 50693, 50694, 50695, 50947, 50948, 50953, 50972, 52005, 52332 |
| 103 | Nephrotomy and nephrostomy | 5501, 5502, 5503, 5504, 5511, 5512 | 50010, 50020, 50040, 50045, 50060, 50065, 50070, 50075, 50080, 50081, 50120, 50125, 50130, 50135, 50432, 50433, 50434, 50435, 50436, 50437, 52334 |
| 104 | Nephrectomy, partial or complete | 554, 5551, 5552, 5553, 5554 | 50220, 50225, 50230, 50234, 50236, 50240, 50300, 50320, 50323, 50325, 50327, 50328, 50329, 50340, 50370, 50543, 50545, 50546, 50547, 50548 |
| 105 | Kidney transplant | 5561, 5569 | 50360, 50365, 50380, S2065 |
| 106 | Genitourinary incontinence procedures | 593, 594, 595, 596, 5971, 5972, 5979 | 51715, 51840, 51841, 51845, 51990, 51992, 53440, 53442, 53445, 53447, 53448, 53449, 53451, 53452, 53453, 53454, 57220, 57287, 57288 |
| 107 | Extracorporeal lithotripsy, urinary | 5995, 5996, 9851 | 50590, 52353, 52356, S0400 |
| 108 | Indwelling catheter | 5794 | 51701, 51702, 51703 |
| 109 | Procedures on the urethra | 580, 581, 583, 5831, 5839, 5841, 5842, 5843, 5844, 5845, 5846, 5847, 5849, 585, 586, 5891, 5892, 5893, 5899 | 0499T, 0596T, 0597T, 52270, 52275, 52277, 52281, 52282, 52283, 52285, 52290, 52400, 52441, 52442, 53000, 53010, 53020, 53025, 53040, 53060, 53210, 53215, 53220, 53230, 53235, 53240, 53250, 53260, 53265, 53270, 53275, 53400, 53405, 53410, 53415, 53420, 53425, 53430, 53431, 53444, 53446, 53450, 53460, 53500, 53502, 53505, 53510, 53515, 53520, 53600, 53601, 53605, 53620, 53621, 53660, 53661, 53665, 53855, 53860, 54308, 54312, 54316, 54318, 57310, 57311 |
| 110 | Other diagnostic procedures of urinary tract | 5523, 5524, 5529, 5632, 5634, 5635, 5639, 5731, 5734, 5739, 5821, 5824, 5829, 5921, 5929 | 50200, 50205, 50549, 51999, C9761, P9612, P9615 |
| 111 | Other non-OR therapeutic procedures of urinary tract | 5592, 5593, 5594, 5595, 5596, 5691, 5711, 5717, 5792, 5795, 5993, 5994, 5999, 9625, 9645, 9646, 9647, 9648, 9649, 9761, 9762, 9763, 9764, 9765, 9769 | 50382, 50384, 50385, 50386, 50387, 50389, 50390, 50391, 50592, 50606, 50688, 50705, 50706, 51100, 51700, 51705, 51710, 52265, 53899 |
| 112 | Other OR therapeutic procedures of urinary tract | 5531, 5532, 5533, 5534, 5535, 5539, 557, 5581, 5582, 5583, 5584, 5585, 5586, 5587, 5589, 5591, 5597, 5598, 5599, 561, 562, 5640, 5641, 5642, 5651, 5652, 5661, 5662, 5671, 5672, 5673, 5674, 5675, 5679, 5681, 5682, 5683, 5684, 5685, 5686, 5689, 5692, 5693, 5694, 5695, 5699, 5712, 5718, 5719, 5721, 5722, 5751, 5759, 576, 5771, 5779, 5781, 5782, 5783, 5784, 5785, 5786, 5787, 5788, 5789, 5791, 5793, 5796, 5797, 5798, 5799, 5900, 5901, 5902, 5903, 5909, 5911, 5912, 5919, 5991, 5992 | 50250, 50280, 50290, 50400, 50405, 50500, 50520, 50525, 50526, 50540, 50541, 50542, 50544, 50557, 50562, 50575, 50576, 50593, 50600, 50610, 50620, 50630, 50650, 50660, 50700, 50715, 50722, 50725, 50727, 50728, 50740, 50750, 50760, 50770, 50780, 50782, 50783, 50785, 50800, 50810, 50815, 50820, 50825, 50830, 50840, 50845, 50860, 50900, 50920, 50930, 50940, 50945, 50949, 51020, 51030, 51040, 51045, 51050, 51060, 51065, 51080, 51101, 51102, 51500, 51520, 51525, 51530, 51535, 51550, 51555, 51565, 51570, 51575, 51580, 51585, 51590, 51595, 51596, 51597, 51800, 51820, 51860, 51865, 51880, 51900, 51920, 51940, 51960, 51980, 52250, 52260, 52287, 52317, 52318, 52327, 52355, 53080, 53085, 57320, 57330 |
| 113 | Transurethral resection of prostate (TURP) | 602, 6021, 6029, 6096, 6097 | 0421T, 0582T, 52601, 52630, 53850, 53852, 53854 |
| 114 | Open prostatectomy | 603, 604, 605, 6061, 6062, 6069 | 55801, 55810, 55812, 55815, 55821, 55831, 55840, 55842, 55845, 55866, 55873 |
| 115 | Circumcision | 640 | 54150, 54160, 54161, 54163 |
| 116 | Diagnostic procedures, male genital | 6011, 6012, 6013, 6014, 6015, 6018, 6019, 6111, 6119, 6211, 6212, 6219, 6301, 6309, 6411, 6419 | 0443T, 54100, 54105, 54231, 54235, 54240, 54250, 54500, 54505, 54699, 54800, 54865, 55110, 55700, 55705, 55706, G0102, G0416 |
| 117 | Other non-OR therapeutic procedures, male genital | 6071, 6091, 6092, 6095, 610, 613, 6141, 6191, 6291, 6292, 6352, 636, 6370, 6371, 6372, 6373, 6384, 6391, 6491, 6494, 9994, 9995, 9996 | 0619T, 54000, 54001, 54050, 54055, 54056, 54057, 54065, 54200, 54220, 54450, 55000, 55100, 55120, 55200, 55250, 55870, 55875, 55876, 55880, 55899, 55920 |
| 118 | Other OR therapeutic procedures, male genital | 600, 6072, 6073, 6079, 6081, 6082, 6093, 6094, 6099, 612, 6142, 6149, 6192, 6199, 620, 622, 623, 6241, 6242, 625, 6261, 6269, 627, 6299, 631, 632, 633, 634, 6351, 6353, 6359, 6381, 6382, 6383, 6385, 6389, 6392, 6393, 6394, 6395, 6399, 642, 643, 6441, 6442, 6443, 6444, 6445, 6449, 645, 6492, 6493, 6495, 6496, 6497, 6498, 6499 | 0655T, 52402, 52450, 52647, 52648, 52649, 52700, 54015, 54060, 54110, 54111, 54112, 54115, 54120, 54125, 54130, 54135, 54162, 54164, 54205, 54300, 54304, 54322, 54324, 54326, 54328, 54332, 54336, 54340, 54344, 54348, 54352, 54360, 54380, 54385, 54390, 54400, 54401, 54405, 54406, 54408, 54410, 54411, 54415, 54416, 54417, 54420, 54430, 54435, 54437, 54438, 54440, 54512, 54520, 54522, 54530, 54535, 54550, 54560, 54600, 54620, 54640, 54650, 54660, 54670, 54680, 54690, 54692, 54700, 54830, 54840, 54860, 54861, 54900, 54901, 55040, 55041, 55060, 55150, 55175, 55180, 55400, 55500, 55520, 55530, 55535, 55550, 55559, 55600, 55605, 55650, 55680, 55720, 55725, 55860, 55862, 55865, 55970, C9739, C9740, C9769 |
| 119 | Oophorectomy, unilateral and bilateral | 653, 6531, 6539, 654, 6541, 6549, 6551, 6552, 6553, 6554, 6561, 6562, 6563, 6564 | 58661, 58720, 58940, 58943, 58950, 58952 |
| 120 | Other operations on ovary | 650, 6501, 6509, 6521, 6522, 6523, 6524, 6525, 6529, 6571, 6572, 6573, 6574, 6575, 6576, 6579, 658, 6581, 6589, 6591, 6592, 6593, 6594, 6595, 6599 | 58740, 58800, 58805, 58820, 58822, 58825, 58920, 58925, 58970 |
| 121 | Ligation of fallopian tubes | 6621, 6622, 6629, 6631, 6632, 6639 | 0567T, 58600, 58605, 58611, 58615, 58670, 58671 |
| 122 | Removal of ectopic pregnancy | 6662, 6911, 743 | 59120, 59121, 59130, 59136, 59140, 59150, 59151 |
| 123 | Other operations on fallopian tubes | 664, 6651, 6652, 6661, 6663, 6669, 6671, 6672, 6673, 6674, 6679, 668, 6691, 6692, 6693, 6694, 6695, 6696, 6697, 6699 | 58345, 58350, 58565, 58672, 58700, 58750, 58752, 58760, 58770 |
| 124 | Hysterectomy, abdominal and vaginal | 683, 6831, 6839, 684, 6841, 6849, 685, 6851, 6859, 686, 6861, 6869, 687, 6871, 6879, 689 | 0664T, 0665T, 0666T, 0667T, 51925, 58150, 58152, 58180, 58200, 58210, 58240, 58260, 58262, 58263, 58267, 58270, 58275, 58280, 58285, 58290, 58291, 58292, 58294, 58541, 58542, 58543, 58544, 58548, 58550, 58552, 58553, 58554, 58570, 58571, 58572, 58573, 58575, 58951, 58953, 58954, 58956, 59525 |
| 125 | Other excision of cervix and uterus | 672, 6731, 6732, 6733, 6739, 674, 6821, 6822, 6823, 6829, 6919 | 57461, 57510, 57511, 57513, 57520, 57522, 57530, 57531, 57540, 57545, 57550, 57558, 58140, 58145, 58146, 58353, 58356, 58545, 58546, 58559, 58560, 58561, 58563 |
| 126 | Abortion (termination of pregnancy) | 6901, 6951, 7491, 750 | 59840, 59841, 59850, 59851, 59852, 59855, 59856, 59857, S0199, S2260, S2265, S2266, S2267, S8055 |
| 127 | Dilatation and curettage (D&C), aspiration after delivery or abortion | 6902, 6952 | 59160, 59812, 59820, 59821, 59830 |
| 128 | Diagnostic dilatation and curettage (D&C) | 6909 | 58120 |
| 129 | Repair of cystocele and rectocele, obliteration of vaginal vault | 7050, 7051, 7052, 7053, 7054, 7055, 708 | 45560, 57120, 57230, 57240, 57250, 57260, 57265, 57267, 57268, 57270, 57284, 57285, 57289, 57423, 57555, 57556 |
| 130 | Other diagnostic procedures, female organs | 6511, 6512, 6513, 6514, 6519, 6611, 6619, 6711, 6712, 6719, 6811, 6812, 6813, 6814, 6815, 6816, 6819, 7021, 7022, 7023, 7024, 7029, 7111, 7119 | 0487T, 0568T, 56605, 56606, 56820, 56821, 57000, 57100, 57105, 57410, 57420, 57421, 57452, 57454, 57455, 57456, 57460, 57465, 57500, 58100, 58110, 58340, 58555, 58558, 58578, 58579, 58679, 58900, 58960, G0101, G0123 |
| 131 | Other non-OR therapeutic procedures, female organs | 670, 6959, 696, 697, 6991, 6992, 6993, 6994, 6996, 700, 7011, 7121, 9614, 9615, 9616, 9617, 9618, 9644, 9724, 9725, 9726, 9771, 9772, 9773, 9774, 9775 | 0071T, 0072T, 0404T, 0672T, 56442, 56501, 56515, 57020, 57061, 57065, 57150, 57155, 57156, 57160, 57170, 57180, 57400, 57800, 58300, 58301, 58321, 58322, 58323, 58346, 58974, 58999, 59871 |
| 132 | Other OR therapeutic procedures, female organs | 660, 6601, 6602, 675, 6761, 6762, 6769, 680, 688, 6921, 6922, 6923, 6929, 693, 6941, 6942, 6949, 6995, 6997, 6998, 6999, 7012, 7013, 7014, 7031, 7032, 7033, 704, 7061, 7062, 7063, 7064, 7071, 7072, 7073, 7074, 7075, 7076, 7077, 7078, 7079, 7091, 7092, 7093, 7094, 7095, 7101, 7109, 7122, 7123, 7124, 7129, 713, 714, 715, 7161, 7162, 7171, 7172, 7179, 718, 719 | 0581T, 19105, 46744, 46746, 46748, 55980, 56405, 56420, 56440, 56441, 56620, 56625, 56630, 56631, 56632, 56633, 56634, 56637, 56640, 56700, 56740, 56800, 56805, 56810, 57010, 57023, 57106, 57107, 57109, 57110, 57111, 57130, 57135, 57200, 57210, 57280, 57282, 57283, 57291, 57292, 57295, 57296, 57300, 57305, 57308, 57335, 57425, 57426, 57505, 57700, 57720, 58400, 58410, 58520, 58540, 58660, 58662, 58673, 58674, 58957, 58958, 58976, 59100, 59870, C9778 |
| 133 | Episiotomy | 736 | - |
| 134 | Cesarean section | 740, 741, 742, 744, 7499 | 59510, 59514, 59515, 59618, 59620, 59622 |
| 135 | Forceps, vacuum, and breech delivery | 720, 721, 7221, 7229, 7231, 7239, 724, 7251, 7252, 7253, 7254, 726, 7271, 7279, 728, 729, 733 | 59409, 59410, 59610, 59612, 59614 |
| 136 | Artificial rupture of membranes to assist delivery | 7301, 7309 | - |
| 137 | Other procedures to assist delivery | 731, 7321, 7322, 734, 7351, 7359, 738, 7391, 7392, 7393, 7394, 7399 | 59412, 59414, 59899 |
| 138 | Diagnostic amniocentesis | 751 | 59000, Q0114 |
| 139 | Fetal monitoring | 7531, 7532, 7533, 7534, 7535, 7538 | 59012, 59015, 59020, 59025, 59030, 59050, 59051 |
| 140 | Repair of current obstetric laceration | 7550, 7551, 7552, 7561, 7562, 7569 | 59300, 59350 |
| 141 | Other therapeutic obstetrical procedures including antepartum and postpartum care | 6751, 6759, 752, 7536, 7537, 754, 757, 758, 7591, 7592, 7593, 7594, 7599 | 36460, 57022, 59001, 59070, 59072, 59074, 59076, 59200, 59320, 59325, 59400, 59425, 59426, 59430, 59866, 59897, 59898, S2400, S2401, S2402, S2403, S2404, S2405, S2409, S2411, S4005, S4981 |
| 142 | Partial excision bone | 7601, 762, 7631, 7639, 7760, 7761, 7762, 7763, 7764, 7765, 7766, 7767, 7768, 7769, 7770, 7771, 7772, 7773, 7774, 7775, 7776, 7777, 7778, 7779, 7780, 7781, 7782, 7783, 7784, 7785, 7786, 7787, 7788, 7789 | 15920, 15922, 15933, 15935, 15937, 15941, 15945, 15946, 15951, 15953, 15958, 20150, 21025, 21026, 21029, 21030, 21031, 21032, 21034, 21040, 21044, 21045, 21046, 21047, 21048, 21049, 21050, 21070, 21209, 21295, 21296, 21502, 21600, 21601, 21602, 21603, 21610, 21615, 21616, 21620, 21627, 21630, 21632, 21705, 22100, 22101, 22102, 22103, 22110, 22112, 22114, 22116, 22206, 22207, 22208, 22210, 22212, 22214, 22216, 22220, 22222, 22224, 22226, 22818, 22819, 23120, 23125, 23130, 23140, 23145, 23146, 23150, 23155, 23156, 23180, 23182, 23184, 23190, 23195, 23200, 23210, 23220, 24110, 24115, 24116, 24120, 24125, 24126, 24130, 24140, 24145, 24147, 24149, 24150, 24152, 25119, 25120, 25125, 25126, 25130, 25135, 25136, 25150, 25151, 25170, 25210, 25215, 25230, 25240, 26200, 26205, 26210, 26215, 26230, 26235, 26236, 26250, 26260, 26262, 27065, 27066, 27067, 27070, 27071, 27075, 27076, 27077, 27078, 27122, 27350, 27355, 27356, 27357, 27358, 27360, 27365, 27418, 27635, 27637, 27638, 27640, 27641, 27645, 27646, 27647, 28100, 28104, 28108, 28110, 28111, 28112, 28113, 28114, 28118, 28119, 28120, 28122, 28124, 28126, 28130, 28140, 28150, 28153, 28160, 28171, 28173, 28175, 28288, 29824, 31225, 31230, 32900, 61597, 69970 |
| 143 | Bunionectomy or repair of toe deformities | 7751, 7752, 7753, 7754, 7756, 7757, 7758, 7759 | 28285, 28286, 28289, 28291, 28292, 28295, 28296, 28297, 28298, 28299, 28313, 28340, 28341, 28345, 28755, 28760 |
| 144 | Treatment, facial fracture or dislocation | 7670, 7671, 7672, 7673, 7674, 7675, 7676, 7677, 7678, 7679, 7693, 7694, 7697, 7699 | 21315, 21320, 21325, 21330, 21335, 21336, 21337, 21338, 21339, 21340, 21343, 21344, 21345, 21346, 21347, 21348, 21355, 21356, 21360, 21365, 21366, 21385, 21386, 21387, 21390, 21395, 21400, 21401, 21406, 21407, 21408, 21421, 21422, 21423, 21431, 21432, 21433, 21435, 21436, 21440, 21445, 21450, 21451, 21452, 21453, 21454, 21461, 21462, 21465, 21470, 21480, 21485, 21490, 30930 |
| 145 | Treatment, fracture or dislocation of radius and ulna | 7853, 7863, 7902, 7912, 7922, 7932, 7942, 7952, 7962, 7972, 7973, 7982, 7983, 7992 | 24164, 24586, 24587, 24600, 24605, 24615, 24620, 24635, 24640, 24650, 24655, 24665, 24666, 24670, 24675, 24685, 25400, 25405, 25415, 25420, 25500, 25505, 25515, 25520, 25525, 25526, 25530, 25535, 25545, 25560, 25565, 25574, 25575, 25600, 25605, 25606, 25607, 25608, 25609, 25650, 25651, 25652, 25660, 25671, 25675, 25676, 29847 |
| 146 | Treatment, fracture or dislocation of hip and femur | 7855, 7865, 7905, 7915, 7925, 7935, 7945, 7955, 7965, 7975, 7985, 7995 | 27175, 27176, 27177, 27178, 27179, 27181, 27185, 27187, 27220, 27222, 27230, 27232, 27235, 27236, 27238, 27240, 27244, 27245, 27246, 27248, 27250, 27252, 27253, 27254, 27256, 27257, 27258, 27259, 27265, 27266, 27267, 27268, 27269, 27472, 27495, 27500, 27501, 27502, 27503, 27506, 27507, 27508, 27509, 27510, 27511, 27513, 27514, 27516, 27517, 27519 |
| 147 | Treatment, fracture or dislocation of lower extremity (other than hip or femur) | 7856, 7857, 7858, 7866, 7867, 7868, 7906, 7907, 7908, 7916, 7917, 7918, 7926, 7927, 7928, 7936, 7937, 7938, 7946, 7956, 7966, 7967, 7968, 7976, 7977, 7978, 7986, 7987, 7988, 7996, 7997, 7998 | 27520, 27524, 27530, 27532, 27535, 27536, 27538, 27540, 27550, 27552, 27556, 27557, 27558, 27560, 27562, 27566, 27720, 27722, 27724, 27725, 27726, 27750, 27752, 27756, 27758, 27759, 27760, 27762, 27766, 27767, 27768, 27769, 27780, 27781, 27784, 27786, 27788, 27792, 27808, 27810, 27814, 27816, 27818, 27822, 27823, 27824, 27825, 27826, 27827, 27828, 27829, 27830, 27831, 27832, 27840, 27842, 27846, 27848, 28320, 28322, 28400, 28405, 28406, 28415, 28420, 28430, 28435, 28436, 28445, 28446, 28450, 28455, 28456, 28465, 28470, 28475, 28476, 28485, 28490, 28495, 28496, 28505, 28510, 28515, 28525, 28530, 28531, 28540, 28545, 28546, 28555, 28570, 28575, 28576, 28585, 28600, 28605, 28606, 28615, 28630, 28635, 28636, 28645, 28660, 28665, 28666, 28675, 29850, 29851, 29855, 29856 |
| 148 | Other fracture and dislocation procedure | 7850, 7851, 7852, 7854, 7859, 7860, 7861, 7862, 7864, 7869, 7900, 7901, 7903, 7904, 7909, 7910, 7911, 7913, 7914, 7919, 7920, 7921, 7923, 7924, 7929, 7930, 7931, 7933, 7934, 7939, 7940, 7941, 7949, 7950, 7951, 7959, 7960, 7961, 7963, 7964, 7969, 7970, 7971, 7974, 7979, 7980, 7981, 7984, 7989, 7990, 7991, 7993, 7994, 7999, 8494 | 0200T, 0201T, 21811, 21812, 21813, 21820, 21825, 22310, 22315, 22318, 22319, 22325, 22326, 22327, 22328, 22510, 22511, 22512, 22513, 22514, 22515, 23500, 23505, 23515, 23520, 23525, 23530, 23532, 23540, 23545, 23550, 23552, 23570, 23575, 23585, 23600, 23605, 23615, 23616, 23620, 23625, 23630, 23650, 23655, 23660, 23665, 23670, 23675, 23680, 24160, 24430, 24498, 24500, 24505, 24515, 24516, 24530, 24535, 24538, 24545, 24546, 24560, 24565, 24566, 24575, 24576, 24577, 24579, 24582, 25431, 25440, 25622, 25624, 25628, 25630, 25635, 25645, 25670, 25680, 25685, 25690, 25695, 26600, 26605, 26607, 26608, 26615, 26641, 26645, 26650, 26665, 26670, 26675, 26676, 26685, 26686, 26700, 26705, 26706, 26715, 26720, 26725, 26727, 26735, 26740, 26742, 26746, 26750, 26755, 26756, 26765, 26770, 26775, 26776, 26785, 27197, 27198, 27200, 27202, 27215, 27216, 27217, 27218, 27226, 27227, 27228, 27860, 62000, 62005, 62010, G0412, G0413, G0414, G0415 |
| 149 | Arthroscopy | 8020, 8021, 8022, 8023, 8024, 8025, 8026, 8027, 8028, 8029, 8145 | 29800, 29804, 29805, 29819, 29830, 29834, 29840, 29843, 29860, 29870, 29871, 29888, 29889, 29900, 29999, G0289, S2300 |
| 150 | Division of joint capsule, ligament or cartilage | 8040, 8041, 8042, 8043, 8044, 8045, 8046, 8047, 8048, 8049 | 23415, 24006, 24102, 25085, 26520, 26525, 27036, 27425, 27435, 27612, 28260, 28261, 28262, 28264, 28270, 28272, 29825, 29873, 29884 |
| 151 | Excision of semilunar cartilage of knee | 806 | 27332, 27333, 27347, 27403, 27405, 27407, 27409, 29880, 29881, 29882, 29883 |
| 152 | Arthroplasty knee | 0080, 0081, 0082, 0083, 0084, 8141, 8142, 8143, 8144, 8146, 8147, 8154, 8155 | 27420, 27422, 27424, 27427, 27428, 27429, 27437, 27438, 27440, 27441, 27442, 27443, 27445, 27446, 27447, 27486, 27487, G0428 |
| 153 | Hip replacement, total and partial | 0070, 0071, 0072, 0073, 0074, 0075, 0076, 0077, 0085, 0086, 0087, 8151, 8152, 8153, 8169 | 27125, 27130, 27132, 27134, 27137, 27138, 29914, 29915, 29916, S2118 |
| 154 | Arthroplasty other than hip or knee | 8148, 8149, 8156, 8157, 8159, 8171, 8172, 8173, 8174, 8175, 8179, 8180, 8181, 8182, 8183, 8184, 8185, 8186, 8187, 8188, 8197 | 0098T, 21240, 21242, 21243, 23420, 23470, 23472, 23473, 23474, 24360, 24361, 24362, 24363, 24365, 24366, 24370, 24371, 25332, 25441, 25442, 25443, 25444, 25445, 25446, 25447, 25449, 26530, 26531, 26535, 26536, 27700, 27702, 27703, 29826, S2117 |
| 155 | Arthrocentesis | 8191 | 20600, 20604, 20605, 20606, 20610, 20611 |
| 156 | Injections and aspirations of muscles, tendons, bursa, joints and soft tissue | 8292, 8293, 8294, 8295, 8296, 8394, 8395, 8396, 8397, 8398 | 0263T, 0264T, 0566T, 20500, 20527, 20550, 20551, 20552, 20553, 20612, 64650, 64653, G0259, G0260, M0076 |
| 157 | Amputation of lower extremity | 8410, 8411, 8412, 8413, 8414, 8415, 8416, 8417, 8418, 8419 | 27290, 27295, 27590, 27591, 27592, 27594, 27596, 27598, 27880, 27881, 27882, 27884, 27886, 27888, 27889, 28800, 28805, 28810, 28820, 28825 |
| 158 | Spinal fusion | 8100, 8101, 8102, 8103, 8104, 8105, 8106, 8107, 8108, 8109, 8130, 8131, 8132, 8133, 8134, 8135, 8136, 8137, 8138, 8139, 8161, 8162, 8163, 8164, 8451 | 0163T, 0164T, 0165T, 22532, 22533, 22534, 22548, 22551, 22552, 22554, 22556, 22558, 22585, 22586, 22590, 22595, 22600, 22610, 22612, 22614, 22630, 22632, 22633, 22634, 22800, 22802, 22804, 22808, 22810, 22812, 22840, 22841, 22842, 22843, 22844, 22845, 22846, 22847, 22848, 22849, 22850, 22852, 22853, 22854, 22855, 22857, 22859, 22862, 22865 |
| 159 | Other diagnostic procedures on musculoskeletal system | 7611, 7619, 7740, 7741, 7742, 7743, 7744, 7745, 7746, 7747, 7748, 7749, 7880, 7881, 7882, 7883, 7884, 7885, 7886, 7887, 7888, 7889, 8030, 8031, 8032, 8033, 8034, 8035, 8036, 8037, 8038, 8039, 8321, 8329 | 0547T, 0554T, 0555T, 0556T, 0557T, 20200, 20205, 20206, 20220, 20225, 20240, 20245, 20250, 20251, 20501, 20950, 21550, 21920, 21925, 23065, 23066, 23100, 23101, 23107, 24065, 24066, 24100, 24101, 25040, 25065, 25066, 25100, 25101, 26100, 26105, 26110, 27040, 27041, 27050, 27052, 27323, 27324, 27330, 27613, 27614, 27620, 28050, 28052, 28054 |
| 160 | Other therapeutic procedures on muscles and tendons | 8201, 8202, 8203, 8204, 8209, 8211, 8212, 8219, 8221, 8222, 8229, 8231, 8232, 8233, 8234, 8235, 8236, 8239, 8241, 8242, 8243, 8244, 8245, 8246, 8251, 8252, 8253, 8254, 8255, 8256, 8257, 8258, 8259, 8261, 8269, 8271, 8272, 8279, 8281, 8282, 8283, 8284, 8285, 8286, 8289, 8291, 8299, 8301, 8302, 8303, 8309, 8311, 8312, 8313, 8314, 8319, 8331, 8332, 8339, 8341, 8342, 8343, 8344, 8345, 8349, 835, 8361, 8362, 8363, 8364, 8365, 8371, 8372, 8373, 8374, 8375, 8376, 8377, 8379, 8381, 8382, 8383, 8384, 8385, 8386, 8387, 8388, 8389, 8391, 8392, 8393, 8399 | 20520, 20525, 20920, 20922, 20924, 21013, 21014, 21015, 21016, 21554, 21556, 21557, 21558, 21700, 21720, 21725, 21743, 21930, 21932, 21933, 21935, 21936, 22900, 22901, 22904, 22905, 23000, 23020, 23030, 23073, 23078, 23395, 23397, 23405, 23406, 23410, 23412, 23430, 23440, 23930, 23931, 24073, 24076, 24077, 24079, 24105, 24201, 24301, 24305, 24310, 24320, 24330, 24331, 24332, 24340, 24341, 24342, 24495, 25000, 25001, 25020, 25023, 25024, 25025, 25028, 25031, 25073, 25076, 25077, 25078, 25109, 25110, 25111, 25112, 25115, 25116, 25248, 25260, 25263, 25265, 25270, 25272, 25274, 25275, 25280, 25290, 25295, 25300, 25301, 25310, 25312, 25315, 25316, 26020, 26025, 26030, 26035, 26037, 26040, 26045, 26055, 26060, 26113, 26116, 26117, 26118, 26121, 26123, 26125, 26160, 26170, 26180, 26350, 26352, 26356, 26357, 26358, 26370, 26372, 26373, 26390, 26392, 26410, 26412, 26415, 26416, 26418, 26420, 26426, 26428, 26432, 26433, 26434, 26437, 26440, 26442, 26445, 26449, 26450, 26455, 26460, 26471, 26474, 26476, 26477, 26478, 26479, 26480, 26483, 26485, 26489, 26490, 26492, 26494, 26496, 26497, 26498, 26499, 26500, 26502, 26508, 26510, 26550, 26580, 26591, 26593, 27000, 27001, 27005, 27006, 27025, 27027, 27045, 27048, 27049, 27057, 27059, 27060, 27062, 27087, 27097, 27098, 27100, 27105, 27110, 27111, 27301, 27305, 27306, 27307, 27328, 27329, 27331, 27339, 27340, 27345, 27364, 27372, 27380, 27381, 27385, 27386, 27390, 27391, 27392, 27393, 27394, 27395, 27396, 27397, 27400, 27430, 27496, 27497, 27498, 27499, 27600, 27601, 27602, 27603, 27604, 27605, 27606, 27615, 27616, 27619, 27626, 27630, 27634, 27650, 27652, 27654, 27656, 27658, 27659, 27664, 27665, 27675, 27676, 27680, 27681, 27685, 27686, 27687, 27690, 27691, 27692, 27892, 27893, 27894, 28001, 28002, 28003, 28008, 28010, 28011, 28041, 28045, 28046, 28047, 28060, 28062, 28086, 28088, 28090, 28092, 28192, 28193, 28200, 28202, 28208, 28210, 28220, 28222, 28225, 28226, 28230, 28232, 28234, 28238, 28240, 28250, 28360, 28737, 28890, 29827, 29828 |
| 161 | Other OR therapeutic procedures on bone | 7700, 7701, 7702, 7703, 7704, 7705, 7706, 7707, 7708, 7709, 7710, 7711, 7712, 7713, 7714, 7715, 7716, 7717, 7718, 7719, 7720, 7721, 7722, 7723, 7724, 7725, 7726, 7727, 7728, 7729, 7730, 7731, 7732, 7733, 7734, 7735, 7736, 7737, 7738, 7739, 7790, 7791, 7792, 7793, 7794, 7795, 7796, 7797, 7798, 7799, 7800, 7801, 7802, 7803, 7804, 7805, 7806, 7807, 7808, 7809, 7810, 7811, 7812, 7813, 7814, 7815, 7816, 7817, 7818, 7819, 7820, 7822, 7823, 7824, 7825, 7827, 7828, 7829, 7830, 7831, 7832, 7833, 7834, 7835, 7837, 7838, 7839, 7840, 7841, 7842, 7843, 7844, 7845, 7846, 7847, 7848, 7849, 7870, 7871, 7872, 7873, 7874, 7875, 7876, 7877, 7878, 7879, 7890, 7891, 7892, 7893, 7894, 7895, 7896, 7897, 7898, 7899, 8165, 8166, 8471, 8472, 8473 | 0594T, 0707T, 20615, 20670, 20680, 20690, 20692, 20693, 20694, 20696, 20697, 20900, 20902, 20930, 20931, 20936, 20937, 20938, 20939, 20955, 20956, 20957, 20962, 20969, 20970, 20972, 20973, 20975, 21120, 21121, 21122, 21123, 21125, 21127, 21137, 21138, 21139, 21141, 21142, 21143, 21145, 21146, 21147, 21150, 21151, 21154, 21155, 21159, 21160, 21172, 21175, 21179, 21180, 21181, 21182, 21183, 21184, 21188, 21193, 21194, 21195, 21196, 21198, 21199, 21206, 21208, 21210, 21215, 21244, 21245, 21246, 21247, 21248, 21249, 21255, 21256, 21260, 21261, 21263, 21267, 21268, 21270, 21275, 21510, 21750, 22830, 23035, 23170, 23172, 23174, 23400, 23480, 23485, 23490, 23491, 23935, 24134, 24136, 24138, 24400, 24410, 24420, 24435, 24470, 24935, 25035, 25145, 25350, 25355, 25360, 25365, 25370, 25375, 25390, 25391, 25392, 25393, 25394, 25425, 25426, 25430, 25450, 25455, 25490, 25491, 25492, 26034, 26185, 26546, 26551, 26553, 26554, 26555, 26565, 26567, 26568, 26992, 27080, 27140, 27151, 27161, 27165, 27170, 27303, 27448, 27450, 27454, 27455, 27457, 27465, 27466, 27468, 27475, 27477, 27479, 27485, 27607, 27705, 27707, 27709, 27712, 27715, 27727, 27730, 27732, 27734, 27740, 27742, 27745, 28005, 28102, 28103, 28106, 28107, 28116, 28300, 28302, 28304, 28305, 28306, 28307, 28308, 28309, 28310, 28312, 28315, 28344, 61316 |
| 162 | Other OR therapeutic procedures on joints | 8000, 8001, 8002, 8003, 8004, 8005, 8006, 8007, 8008, 8009, 8010, 8011, 8012, 8013, 8014, 8015, 8016, 8017, 8018, 8019, 8052, 8070, 8071, 8072, 8073, 8074, 8075, 8076, 8077, 8078, 8079, 8080, 8081, 8082, 8083, 8084, 8085, 8086, 8087, 8088, 8089, 8090, 8091, 8092, 8093, 8094, 8095, 8096, 8097, 8098, 8099, 8111, 8112, 8113, 8114, 8115, 8116, 8117, 8118, 8120, 8121, 8122, 8123, 8124, 8125, 8126, 8127, 8128, 8129, 8140, 8193, 8194, 8195, 8196, 8198, 8199 | 0095T, 0335T, 0510T, 0511T, 21010, 23031, 23040, 23044, 23105, 23106, 23334, 23335, 23450, 23455, 23460, 23462, 23465, 23466, 23800, 23802, 24000, 24155, 24343, 24344, 24345, 24346, 24357, 24358, 24359, 24800, 24802, 25105, 25107, 25118, 25250, 25251, 25320, 25335, 25337, 25800, 25805, 25810, 25820, 25825, 25830, 26070, 26075, 26080, 26130, 26135, 26140, 26145, 26516, 26517, 26518, 26540, 26541, 26542, 26545, 26548, 26556, 26820, 26841, 26842, 26843, 26844, 26850, 26852, 26860, 26861, 26862, 26863, 26990, 26991, 27030, 27033, 27054, 27090, 27091, 27120, 27146, 27147, 27156, 27158, 27279, 27280, 27282, 27284, 27286, 27310, 27334, 27335, 27412, 27415, 27416, 27488, 27570, 27580, 27610, 27625, 27695, 27696, 27698, 27704, 27870, 27871, 28020, 28022, 28024, 28070, 28072, 28705, 28715, 28725, 28730, 28735, 28740, 28750, 29806, 29807, 29820, 29821, 29822, 29823, 29835, 29836, 29837, 29838, 29844, 29845, 29846, 29861, 29862, 29863, 29866, 29867, 29868, 29874, 29875, 29876, 29877, 29879, 29885, 29886, 29887, 29891, 29892, 29894, 29895, 29897, 29898, 29899, 29901, 29902, 29904, 29905, 29906, 29907, C9781, S2115, S2325 |
| 163 | Other non-OR therapeutic procedures on musculoskeletal system | 7695, 7696, 8192, 8441, 8442, 8443, 8445, 8446, 8447, 8452, 8453, 8454, 8455, 8456, 8457, 9711, 9712, 9713, 9714, 9736, 9788 | 0101T, 0102T, 0481T, 0512T, 20974, 20979, 20982, 20983, 20999, 21073, 21299, 21499, 21899, 22899, 22999, 23700, 23929, 24300, 24999, 25259, 25999, 26340, 26341, 26989, 27275, 27299, 27599, 27899, 28899, 29700, 29705, 29710, 79440, 79445, 98940, 98941, 98942, 98943 |
| 164 | Other OR therapeutic procedures on musculoskeletal system | 7609, 7641, 7642, 7643, 7644, 7645, 7646, 765, 7661, 7662, 7663, 7664, 7665, 7666, 7667, 7668, 7669, 7691, 7692, 8400, 8401, 8402, 8403, 8404, 8405, 8406, 8407, 8408, 8409, 8421, 8422, 8423, 8424, 8425, 8426, 8427, 8428, 8429, 843, 8440, 8444, 8448, 8491, 8492, 8493, 8499 | 0219T, 0220T, 0221T, 0222T, 0513T, 0656T, 0657T, 20555, 20702, 20703, 20704, 20705, 20802, 20805, 20808, 20816, 20822, 20824, 20827, 20838, 20910, 20932, 20933, 20934, 20985, 21060, 21230, 21235, 21685, 21740, 21742, 22867, 22868, 22869, 22870, 23076, 23077, 23333, 23900, 23920, 23921, 24900, 24920, 24925, 24930, 24931, 24940, 25900, 25905, 25907, 25909, 25915, 25920, 25922, 25924, 25927, 25929, 25931, 26320, 26587, 26590, 26910, 26951, 26952, 27470 |
| 165 | Breast biopsy and other diagnostic procedures on breast | 8511, 8512, 8519 | 19000, 19001, 19030, 19081, 19082, 19083, 19084, 19085, 19086, 19100, 19101, 19281, 19282, 19283, 19284, 19285, 19286, 19287, 19288 |
| 166 | Lumpectomy, quadrantectomy of breast | 8520, 8521, 8522, 8523 | 19120, 19125, 19126, 19294, 19297, 19301, 19302 |
| 167 | Mastectomy | 8541, 8542, 8543, 8544, 8545, 8546, 8547, 8548 | 19300, 19303, 19305, 19306, 19307 |
| 168 | Incision and drainage, skin and subcutaneous tissue | 8604 | 10040, 10060, 10061, 10080, 10081, 10120, 10121, 10140, 10180, 11740, 21501, 22010, 22015, 26010, 26011 |
| 169 | Debridement of wound, infection or burn | 8622, 8628 | 11000, 11001, 11004, 11005, 11006, 11008, 11010, 11011, 11012, 11042, 11043, 11044, 11045, 11046, 11047, 16020, 16025, 16030, 69220, 69222 |
| 170 | Excision of skin lesion | 863, 864 | 0479T, 0480T, 10160, 11055, 11056, 11057, 11200, 11201, 11300, 11301, 11302, 11303, 11305, 11306, 11307, 11308, 11310, 11311, 11312, 11313, 11400, 11401, 11402, 11403, 11404, 11406, 11420, 11421, 11422, 11423, 11424, 11426, 11440, 11441, 11442, 11443, 11444, 11446, 11450, 11451, 11462, 11463, 11470, 11471, 11600, 11601, 11602, 11603, 11604, 11606, 11620, 11621, 11622, 11623, 11624, 11626, 11640, 11641, 11642, 11643, 11644, 11646, 15931, 15934, 15936, 15940, 15944, 15950, 15952, 15956, 15999, 17000, 17003, 17004, 17106, 17107, 17108, 17110, 17111, 17250, 17260, 17261, 17262, 17263, 17264, 17266, 17270, 17271, 17272, 17273, 17274, 17276, 17280, 17281, 17282, 17283, 17284, 17286, 17311, 17312, 17313, 17314, 17315, 17340, 17360, 17380, 21011, 21012, 21552, 21555, 21931, 22902, 22903, 23071, 23075, 24071, 24075, 25071, 25075, 26111, 26115, 27043, 27047, 27327, 27337, 27618, 27632, 28039, 28043, 29893, 31830 |
| 171 | Suture of skin and subcutaneous tissue | 8651, 8659 | 12001, 12002, 12004, 12005, 12006, 12007, 12011, 12013, 12014, 12015, 12016, 12017, 12018, 12020, 12021, 12031, 12032, 12034, 12035, 12036, 12037, 12041, 12042, 12044, 12045, 12046, 12047, 12051, 12052, 12053, 12054, 12055, 12056, 12057, 13100, 13101, 13102, 13120, 13121, 13122, 13131, 13132, 13133, 13160 |
| 172 | Skin graft | 8660, 8661, 8662, 8663, 8664, 8665, 8666, 8667, 8669, 8670, 8671, 8672, 8673, 8674, 8675 | 14000, 14001, 14020, 14021, 14040, 14041, 14060, 14061, 14301, 14302, 14350, 15002, 15003, 15004, 15005, 15040, 15050, 15100, 15101, 15110, 15111, 15115, 15116, 15120, 15121, 15130, 15131, 15135, 15136, 15150, 15151, 15152, 15155, 15156, 15157, 15200, 15201, 15220, 15221, 15240, 15241, 15260, 15261, 15271, 15272, 15273, 15274, 15275, 15276, 15277, 15278, 15570, 15572, 15574, 15576, 15600, 15610, 15620, 15630, 15650, 15730, 15731, 15733, 15734, 15736, 15738, 15750, 15756, 15757, 15758, 15760, 15770, 15775, 15776, 15840, 15841, 15842, 15845, 40818, C5271, C5272, C5273, C5274, C5275, C5276, C5277, C5278 |
| 173 | Other diagnostic procedures on skin and subcutaneous tissue | 8611, 8619 | 0493T, 0598T, 0599T, 0631T, 0640T, 0641T, 0642T, 0658T, 0700T, 0701T, 10004, 10005, 10006, 10007, 10008, 10009, 10010, 10011, 10012, 10021, 10035, 10036, 11102, 11103, 11104, 11105, 11106, 11107, 11755, 95004, 95017, 95018, 95024, 95027, 95028, 95044, 95052, 95056, 95060, 95065, 95070, 95076, 95079, 96931, 96932, 96933, 96934, 96935, 96936 |
| 174 | Other non-OR therapeutic procedures on skin and breast | 850, 8551, 8552, 8581, 8591, 8592, 8601, 8602, 8603, 8605, 8607, 8609, 8623, 8624, 8626, 8627, 8692, 8699 | 0470T, 0471T, 0489T, 0490T, 0491T, 0492T, 0546T, 0565T, 0662T, 0663T, 10030, 11719, 11720, 11721, 11730, 11732, 11750, 11765, 11900, 11901, 11920, 11921, 11922, 11950, 11951, 11952, 11954, 11976, 11980, 11981, 11982, 11983, 15780, 15781, 15782, 15783, 15786, 15788, 15789, 15792, 15793, 15860, 17999, 19396, 19499, 20700, 20701, 23330, 24200, 62367, 62368, 96567, 96570, 96571, 96573, 96574, 96900, 96904, 96910, 96912, 96913, 96920, 96921, 96922, 96999, G0127, G0168, G0247, G0281, G0282, G0283, G0295, G0329, G0516, G0517, G0518, S0390, S2067 |
| 175 | Other OR therapeutic procedures on skin and breast | 8524, 8525, 8531, 8532, 8533, 8534, 8535, 8536, 8550, 8553, 8554, 8555, 856, 857, 8570, 8571, 8572, 8573, 8574, 8575, 8576, 8579, 8582, 8583, 8584, 8585, 8586, 8587, 8589, 8593, 8594, 8595, 8596, 8599, 8606, 8621, 8625, 8681, 8682, 8683, 8684, 8685, 8686, 8687, 8689, 8690, 8691, 8693 | 0419T, 0420T, 11760, 11762, 11770, 11771, 11772, 11960, 11970, 11971, 15740, 15769, 15771, 15772, 15773, 15774, 15777, 15787, 15819, 15824, 15825, 15826, 15828, 15829, 15830, 15832, 15833, 15834, 15835, 15836, 15837, 15838, 15839, 15847, 15876, 15877, 15878, 15879, 16035, 16036, 19020, 19110, 19112, 19296, 19298, 19316, 19318, 19325, 19328, 19330, 19340, 19342, 19350, 19355, 19357, 19361, 19364, 19367, 19368, 19369, 19370, 19371, 19380, 20100, 20101, 20102, 20103, 26560, 26561, 26562, 26596, 27086, 28190, 28280, 30120, 30124, 61215, 61885, 61886, 61888, S2066, S2068 |
| 176 | Other organ transplantation | 335, 3350, 3351, 3352, 336, 375, 3751, 4194, 4697, 5051, 5059, 5280, 5281, 5282, 5283, 5284, 5285, 5286 | 0494T, 0495T, 0496T, 0584T, 0585T, 0586T, 0668T, 0669T, 0670T, 32851, 32852, 32853, 32854, 32855, 32856, 33930, 33933, 33935, 33944, 33945, 44135, 44136, 44715, 44720, 44721, 47135, 47140, 47141, 47142, 47143, 47144, 47145, 47146, 47147, 48160, 48551, 48552, 48554, 65780, 65781, 65782, G0341, G0342, G0343, S2053, S2054, S2055, S2060, S2102, S2103, S2152 |
| 177 | Computerized axial tomography (CT) scan head | 8703 | 70450, 70460, 70470, 70480, 70481, 70482, 70486, 70487, 70488, 70496 |
| 178 | CT scan chest | 8741 | 71250, 71260, 71270, 71271, 71275, 75571, 75572, 75573 |
| 179 | CT scan abdomen | 8801 | 72191, 72192, 72193, 72194, 74150, 74160, 74170, 74174, 74175, 74176, 74177, 74178, 74261, 74262, 74263 |
| 180 | Other CT scan | 0031, 8771, 8838 | 0042T, 0351T, 0352T, 0353T, 0354T, 0501T, 0502T, 0503T, 0504T, 0558T, 0633T, 0634T, 0635T, 0636T, 0637T, 0638T, 70490, 70491, 70492, 72125, 72126, 72127, 72128, 72129, 72130, 72131, 72132, 72133, 73200, 73201, 73202, 73206, 73700, 73701, 73702, 73706, 76380, 76497, 77011, 77012, 77013, 77014, 77078, S8092 |
| 181 | Myelogram | 8721 | 62284, 62302, 62303, 62304, 62305, 70010, 72240, 72255, 72265, 72270 |
| 182 | Mammography | 8736, 8737 | 77053, 77054, 77061, 77062, 77063, 77065, 77066, 77067, S8080 |
| 183 | Routine chest X-ray | 8744 | 0174T, 0175T, 71045, 71046, 71047, 71048 |
| 184 | Intraoperative cholangiogram | 8753 | 74300, 74301 |
| 185 | Upper gastrointestinal X-ray | 8761, 8762 | 74220, 74221, 74230, 74240, 74246, 74248, 74250, 74340 |
| 186 | Lower gastrointestinal X-ray | 8764 | 74251, 74270, 74280, 74283, G0106, G0120, G0122 |
| 187 | Intravenous pyelogram | 8773 | 74400, 74410 |
| 188 | Cerebral arteriogram | 8841 | - |
| 189 | Contrast aortogram | 8842 | 75600, 75605, 75625, 75630, 75635, G0288 |
| 190 | Contrast arteriogram of femoral and lower extremity arteries | 8848 | 75820, 75822 |
| 191 | Arterio- or venogram (not heart and head) | 8840, 8843, 8844, 8845, 8846, 8847, 8849, 8850, 8851, 8858, 8860, 8861, 8862, 8863, 8864, 8865, 8866, 8867, 8868 | 70498, 75705, 75710, 75716, 75726, 75731, 75733, 75736, 75741, 75743, 75746, 75774, 75810, 75825, 75827, 75831, 75833, 75840, 75842, 75860, 75870, 75872, 75880, 75885, 75887, 75889, 75891, 75894, 75898, 75956, 75957, 75958, 75959 |
| 192 | Diagnostic ultrasound of head and neck | 0021, 8871 | 76506, 76510, 76511, 76512, 76513, 76514, 76516, 76519, 76529, 76536, 93880, 93882, 93886, 93888, 93890, 93892, 93893, 93895, S9024 |
| 193 | Diagnostic ultrasound of heart (echocardiogram) | 0024, 8872 | 0439T, 76825, 76826, 76827, 76828, 76932, 92978, 92979, 93303, 93304, 93306, 93307, 93308, 93312, 93313, 93314, 93315, 93316, 93317, 93318, 93319, 93320, 93321, 93325, 93350, 93351, 93352, 93355, 93356, 93662, C8921, C8922, C8923, C8924, C8925, C8926, C8927, C8928, C8929, C8930 |
| 194 | Diagnostic ultrasound of gastrointestinal tract | 8874 | 76975 |
| 195 | Diagnostic ultrasound of urinary tract | 0025, 8875 | 76776 |
| 196 | Diagnostic ultrasound of abdomen or retroperitoneum | 8876 | 76700, 76705, 76706, 76770, 76775, 93975, 93976 |
| 197 | Other diagnostic ultrasound | 0022, 0023, 0028, 0029, 8873, 8877, 8878, 8879 | 0508T, 0689T, 0690T, 31654, 37252, 37253, 76604, 76641, 76642, 76800, 76801, 76802, 76805, 76810, 76811, 76812, 76813, 76814, 76815, 76816, 76817, 76818, 76819, 76820, 76821, 76830, 76831, 76856, 76857, 76870, 76872, 76873, 76881, 76882, 76885, 76886, 76936, 76937, 76940, 76941, 76942, 76945, 76946, 76948, 76977, 76978, 76979, 76981, 76982, 76983, 76998, 76999, 93925, 93926, 93930, 93931, 93970, 93971, 93978, 93979, 93980, 93981, 93985, 93986, 93990, 97610 |
| 198 | Magnetic resonance imaging | 0032, 8891, 8892, 8893, 8894, 8895, 8896, 8897, 8899 | 0648T, 0649T, 0697T, 0698T, 70336, 70540, 70542, 70543, 70544, 70545, 70546, 70547, 70548, 70549, 70551, 70552, 70553, 70554, 70555, 70557, 70558, 70559, 71550, 71551, 71552, 71555, 72141, 72142, 72146, 72147, 72148, 72149, 72156, 72157, 72158, 72159, 72195, 72196, 72197, 72198, 73218, 73219, 73220, 73221, 73222, 73223, 73225, 73718, 73719, 73720, 73721, 73722, 73723, 73725, 74181, 74182, 74183, 74185, 74712, 74713, 75557, 75559, 75561, 75563, 75565, 76391, 76498, 77021, 77022, 77046, 77047, 77048, 77049, 77084, C8900, C8901, C8902, C8903, C8905, C8906, C8908, C8909, C8910, C8911, C8912, C8913, C8914, C8918, C8919, C8920, C8931, C8932, C8933, C8934, C8935, C8936, C9762, C9763, S8035, S8037, S8042 |
| 199 | Electroencephalogram (EEG) | 8914 | 95700, 95705, 95706, 95707, 95708, 95709, 95710, 95711, 95712, 95713, 95714, 95715, 95716, 95717, 95718, 95719, 95720, 95721, 95722, 95723, 95724, 95725, 95726, 95812, 95813, 95816, 95819, 95822, 95824, 95829, 95830, 95954, 95955, 95957, 95958, 95961, 95962 |
| 200 | Nonoperative urinary system measurements | 8929 | 0602T, 0603T, 50396, 50684, 50686, 50690, 51600, 51605, 51610, 51725, 51726, 51727, 51728, 51729, 51736, 51741, 51784, 51785, 51792, 51797, 51798, 81002, 81003, 81005, 81025, 81050, 81099, 82042, 82131 |
| 201 | Cardiac stress tests | 8941, 8942, 8943, 8944 | 93015, 93016, 93017, 93018, Q0035, S3904 |
| 202 | Electrocardiogram | 8951, 8952 | 0695T, 0696T, 93000, 93005, 93010, 93025, 93040, 93041, 93042, G0403, G0404, G0405, S9025 |
| 203 | Electrographic cardiac monitoring | 8954 | 0417T, 0418T, 0497T, 0498T, 0521T, 0522T, 0528T, 0529T, 0575T, 0576T, 93224, 93225, 93226, 93227, 93241, 93242, 93243, 93244, 93245, 93246, 93247, 93248, 93278, 93279, 93280, 93281, 93282, 93283, 93284, 93285, 93286, 93287, 93288, 93289, 93290, 93291, 93292, 93660, 93724, 93745, G9157 |
| 204 | Swan-Ganz catheterization for monitoring | 8964 | 93503 |
| 205 | Arterial blood gases | 8960, 8965 | 82800, 82803, 82805, 82810 |
| 206 | Microscopic examination (bacterial smear, culture, toxicology) | 9001, 9002, 9003, 9004, 9005, 9006, 9009, 9011, 9012, 9013, 9014, 9015, 9016, 9019, 9021, 9022, 9023, 9024, 9025, 9026, 9029, 9031, 9032, 9033, 9034, 9035, 9036, 9039, 9041, 9042, 9043, 9044, 9045, 9046, 9049, 9051, 9052, 9053, 9054, 9055, 9056, 9059, 9061, 9062, 9063, 9064, 9065, 9066, 9069, 9071, 9072, 9073, 9074, 9075, 9076, 9079, 9081, 9082, 9083, 9084, 9085, 9086, 9089, 9091, 9092, 9093, 9094, 9095, 9096, 9099, 9101, 9102, 9103, 9104, 9105, 9106, 9109, 9111, 9112, 9113, 9114, 9115, 9116, 9119, 9121, 9122, 9123, 9124, 9125, 9126, 9129, 9131, 9132, 9133, 9134, 9135, 9136, 9139, 9141, 9142, 9143, 9144, 9145, 9146, 9149, 9151, 9152, 9153, 9154, 9155, 9156, 9159, 9161, 9162, 9163, 9164, 9165, 9166, 9169, 9171, 9172, 9173, 9174, 9175, 9176, 9179, 9181, 9182, 9183, 9184, 9185, 9186, 9189, 9191, 9192, 9193, 9194, 9195, 9196, 9199 | 0004U, 0008U, 0041U, 0042U, 0043U, 0044U, 0068U, 0096U, 0097U, 0107U, 0109U, 0115U, 0140U, 0141U, 0142U, 0151U, 0152U, 0202U, 0219U, 0223U, 0225U, 0240U, 0241U, 0301U, 0302U, 0311U, 0321U, 0500T, 81000, 81001, 81007, 81015, 81020, 82043, 82044, 82045, 82120, 82127, 82128, 82175, 83015, 83018, 83885, 85097, 86812, 86813, 86816, 86817, 86821, 86825, 86826, 87015, 87040, 87045, 87046, 87070, 87071, 87073, 87075, 87076, 87077, 87081, 87084, 87086, 87088, 87101, 87102, 87103, 87106, 87107, 87109, 87110, 87116, 87118, 87140, 87143, 87147, 87149, 87150, 87152, 87153, 87154, 87158, 87164, 87166, 87168, 87169, 87172, 87176, 87177, 87181, 87184, 87185, 87186, 87187, 87188, 87190, 87197, 87205, 87206, 87207, 87210, 87220, 87230, 87250, 87252, 87253, 87254, 87255, 87260, 87265, 87267, 87269, 87270, 87271, 87272, 87273, 87274, 87275, 87276, 87278, 87279, 87280, 87281, 87283, 87285, 87290, 87299, 87300, 87301, 87305, 87320, 87324, 87327, 87328, 87329, 87332, 87335, 87336, 87337, 87338, 87339, 87340, 87341, 87350, 87380, 87385, 87389, 87390, 87391, 87400, 87420, 87425, 87426, 87427, 87428, 87430, 87449, 87451, 87471, 87472, 87475, 87476, 87480, 87481, 87482, 87483, 87485, 87486, 87487, 87490, 87491, 87492, 87495, 87496, 87497, 87498, 87500, 87501, 87502, 87503, 87505, 87506, 87507, 87510, 87511, 87512, 87516, 87517, 87520, 87521, 87522, 87525, 87526, 87527, 87528, 87529, 87530, 87531, 87532, 87533, 87534, 87535, 87536, 87537, 87538, 87539, 87540, 87541, 87542, 87550, 87551, 87552, 87555, 87556, 87557, 87560, 87561, 87562, 87563, 87580, 87581, 87582, 87590, 87591, 87592, 87623, 87624, 87625, 87631, 87632, 87633, 87636, 87637, 87640, 87641, 87650, 87651, 87652, 87653, 87660, 87661, 87797, 87798, 87799, 87800, 87801, 87802, 87803, 87804, 87806, 87807, 87808, 87809, 87810, 87811, 87850, 87880, 87899, 87900, 87901, 87902, 87903, 87904, 87905, 87906, 87910, 87912, 87913, 87999, 88230, 88233, 88235, 88237, 88239, 88245, 88248, 88249, 88261, 88262, 88263, 88264, 88267, 88269, 88280, 88283, 88285, 88289, 88291, 88299, 89060, 89125, 89160, 89190, 89250, 89251, 89253, 89254, 89255, 89257, 89260, 89261, 89264, 89268, 89272, 89280, 89281, 89290, 89291, 89300, 89310, 89320, 89321, 89322, 89325, 89329, 89330, 89331, 96902, G0475, G0476, P7001, Q0112, Q0113, Q0115 |
| 207 | Radioisotope bone scan | 9214 | 78102, 78103, 78104, 78300, 78305, 78306, 78315, 78350, 78351, 78399 |
| 208 | Radioisotope pulmonary scan | 9215 | 78579, 78580, 78582, 78597, 78598, 78599 |
| 209 | Radioisotope scan and function studies | 9201, 9202, 9203, 9204, 9205, 9209 | 0106U, 0331T, 0332T, 70015, 72285, 72295, 78012, 78013, 78014, 78015, 78016, 78018, 78020, 78070, 78071, 78072, 78075, 78099, 78195, 78202, 78216, 78226, 78227, 78232, 78258, 78262, 78264, 78265, 78266, 78267, 78268, 78282, 78291, 78414, 78428, 78431, 78451, 78452, 78453, 78454, 78468, 78469, 78472, 78473, 78481, 78483, 78492, 78494, 78496, 78601, 78606, 78608, 78609, 78610, 78630, 78635, 78645, 78650, 78660, 78699, 78700, 78701, 78707, 78708, 78709, 78725, 78730, 78740, 78761, 78799, 78800, 78801, 78802, 78803, 78804, 78808, 78811, 78812, 78813, 78814, 78815, 78816, 78830, 78831, 78832, 78835, 78999, G0219, G0252, S8085 |
| 210 | Other radioisotope scan | 9211, 9212, 9213, 9216, 9217, 9218, 9219 | 78110, 78111, 78120, 78121, 78122, 78130, 78140, 78185, 78191, 78199, 78201, 78215, 78230, 78231, 78261, 78278, 78290, 78299, 78429, 78430, 78432, 78433, 78434, 78445, 78456, 78457, 78458, 78459, 78466, 78491, 78499, 78600, 78605, G0235 |
| 211 | Therapeutic radiology | 9220, 9221, 9222, 9223, 9224, 9225, 9226, 9227, 9228, 9229, 9241 | 0394T, 0395T, 41019, 55874, 74235, 76965, 77261, 77262, 77263, 77280, 77285, 77290, 77293, 77295, 77299, 77300, 77301, 77306, 77307, 77316, 77317, 77318, 77321, 77331, 77332, 77333, 77334, 77336, 77338, 77370, 77371, 77372, 77373, 77385, 77386, 77387, 77399, 77401, 77402, 77407, 77412, 77417, 77423, 77424, 77425, 77427, 77431, 77432, 77435, 77469, 77470, 77499, 77520, 77522, 77523, 77525, 77600, 77605, 77610, 77615, 77620, 77750, 77761, 77762, 77763, 77767, 77768, 77770, 77771, 77772, 77778, 77789, 77790, 77799, 79101, 79200, 79300, 79403, C2638, C2639, C2640, C2641, C2642, C2643, C2644, C2645, C2698, C2699, C9725, C9726, C9727, C9728, G0339, G0340, G0458, G6001, G6002, G6003, G6004, G6005, G6006, G6007, G6008, G6009, G6010, G6011, G6012, G6013, G6014, G6015, G6016, G6017, S8030 |
| 212 | Diagnostic physical, occupational, and speech therapy | 9301, 9302, 9303, 9304, 9305, 9306, 9307, 9308, 9309 | 92521, 92522, 92523, 92597, 92605, 92606, 92607, 92608, 92618, 95851, 95852, 96105, 96110, 97161, 97162, 97163, 97164, 97165, 97166, 97167, 97168, 97169, 97170, 97171, 97172, 97750, S9152 |
| 213 | Physical, occupational, and speech therapy exercises; manipulation; and other procedures | 9311, 9312, 9313, 9314, 9315, 9316, 9317, 9318, 9319, 9321, 9322, 9323, 9324, 9325, 9326, 9327, 9328, 9329, 9331, 9332, 9333, 9334, 9335, 9336, 9337, 9338, 9339 | 20560, 20561, 90901, 90912, 90913, 92507, 92508, 92609, 97010, 97014, 97016, 97018, 97022, 97024, 97026, 97028, 97032, 97033, 97034, 97035, 97110, 97112, 97113, 97116, 97124, 97140, 97150, 97530, 97545, 97546, 97810, 97811, 97813, 97814, G0129, G2168, G2169, S8930, S8950, S8990 |
| 214 | Traction, splints, and other wound care | 9341, 9342, 9343, 9344, 9345, 9346, 9351, 9352, 9353, 9354, 9355, 9356, 9357, 9358, 9359 | 15852, 16000, 20650, 20660, 20661, 20662, 20663, 20664, 20665, 29000, 29010, 29015, 29035, 29040, 29044, 29046, 29049, 29055, 29058, 29065, 29075, 29085, 29086, 29105, 29125, 29126, 29130, 29131, 29200, 29240, 29260, 29280, 29305, 29325, 29345, 29355, 29358, 29365, 29405, 29425, 29435, 29440, 29445, 29450, 29505, 29515, 29520, 29530, 29540, 29550, 29580, 29581, 29584, 29720, 29730, 29740, 29750, 29799, 97012, 97036, 97597, 97598, 97602, 97605, 97606, 97607, 97608 |
| 215 | Other physical, occupational, and speech therapy and rehabilitation | 9361, 9362, 9363, 9364, 9365, 9366, 9367, 9371, 9372, 9373, 9374, 9375, 9376, 9377, 9378, 9381, 9382, 9383, 9384, 9385, 9389 | 0552T, 93668, 93797, 93798, 97039, 97129, 97130, 97139, 97533, 97537, 97542, 97755, 97760, 97761, 97763, 97799, 98925, 98926, 98927, 98928, 98929, G0422, G0423, S8940, S9472, S9473, S9476, V5362, V5363 |
| 216 | Respiratory intubation and mechanical ventilation | 9390, 9392, 9601, 9602, 9603, 9604, 9605, 9670, 9671, 9672 | 31500, 94002, 94003, 94004, 94660, 94662 |
| 217 | Other respiratory therapy | 9391, 9394, 9396, 9399 | 94625, 94626, 94640, 94642, 94644, 94645, 94667, 94668, 94669, G0237, G0238, G0239 |
| 218 | Psychological and psychiatric evaluation and therapy | 9401, 9402, 9403, 9408, 9409, 9411, 9412, 9413, 9419, 9421, 9422, 9423, 9424, 9425, 9426, 9427, 9429, 9431, 9432, 9433, 9434, 9435, 9436, 9437, 9438, 9439, 9441, 9442, 9443, 9444, 9445, 9446, 9449, 9451, 9452, 9453, 9454, 9455, 9459 | 0362T, 0373T, 90785, 90791, 90792, 90832, 90833, 90834, 90836, 90837, 90838, 90839, 90840, 90845, 90846, 90847, 90849, 90853, 90863, 90865, 90867, 90868, 90869, 90870, 90875, 90876, 90880, 90882, 90885, 90887, 90889, 90899, 96116, 96121, 96125, 96127, 96130, 96131, 96132, 96133, 96136, 96137, 96138, 96139, 96146, 96156, 96158, 96159, 96160, 96161, 96164, 96165, 96167, 96168, 96170, 96171, 97151, 97152, 97153, 97154, 97155, 97156, 97157, 97158, 99354, 99355, 99484, 99492, 99493, 99494, G0409, G0410, G0411, G0444, G0469, G0470, G0512, G2214, H0031, H0032, H0035, H0036, H0037, H0046, H2013, H2017, H2018, H2027, H2028, H2029, H2030, H2031, H2032, H2033, H2037, H2038, S9480, S9484, S9485, T1040, T1041, T2048 |
| 219 | Alcohol and drug management, treatment, and rehabilitation | 9461, 9462, 9463, 9464, 9465, 9466, 9467, 9468, 9469 | 99408, 99409, G0396, G0397, G0442, G0443, G2011, G2067, G2068, G2069, G2070, G2071, G2072, G2073, G2074, G2075, G2080, G2086, G2087, G2088, H0001, H0002, H0003, H0004, H0005, H0006, H0007, H0008, H0009, H0010, H0011, H0012, H0013, H0014, H0015, H0016, H0017, H0018, H0019, H0020, H0021, H0022, H0023, H0024, H0025, H0026, H0027, H0028, H0029, H0030, H0033, H0034, H0038, H0039, H0040, H0045, H0047, H0048, H0049, H0050, H1000, H1001, H1002, H1003, H1004, H1005, H1010, H1011, H2000, H2001, H2010, H2011, H2012, H2014, H2015, H2016, H2019, H2020, H2025, H2026, H2034, H2035, H2036, S9475, T1006, T1007, T1009, T1010, T1012 |
| 220 | Ophthalmologic and otologic diagnosis and treatment | 9501, 9502, 9503, 9504, 9505, 9506, 9507, 9509, 9511, 9512, 9513, 9514, 9515, 9516, 9521, 9522, 9523, 9524, 9525, 9526, 9531, 9532, 9533, 9534, 9535, 9536, 9541, 9542, 9543, 9544, 9545, 9546, 9547, 9548, 9549 | 0198T, 0208T, 0209T, 0210T, 0211T, 0212T, 0464T, 0469T, 0472T, 0473T, 0485T, 0486T, 0506T, 0507T, 0509T, 0514T, 70030, 70371, 92002, 92004, 92012, 92014, 92015, 92018, 92019, 92020, 92025, 92060, 92065, 92071, 92072, 92081, 92082, 92083, 92100, 92132, 92133, 92134, 92136, 92201, 92202, 92227, 92228, 92229, 92230, 92235, 92240, 92242, 92250, 92260, 92265, 92270, 92273, 92274, 92283, 92284, 92285, 92286, 92287, 92310, 92311, 92312, 92313, 92314, 92315, 92316, 92317, 92325, 92326, 92340, 92341, 92342, 92352, 92353, 92354, 92355, 92358, 92370, 92371, 92499, 92517, 92518, 92519, 92524, 92531, 92532, 92533, 92534, 92537, 92538, 92540, 92541, 92542, 92544, 92545, 92546, 92547, 92548, 92549, 92550, 92551, 92552, 92553, 92555, 92556, 92557, 92558, 92562, 92563, 92565, 92567, 92568, 92570, 92571, 92572, 92575, 92576, 92577, 92579, 92582, 92583, 92584, 92587, 92588, 92590, 92591, 92592, 92593, 92594, 92595, 92596, 92601, 92602, 92603, 92604, 92620, 92621, 92625, 92650, 92651, 92652, 92653, 99172, 99173, 99174, 99177, G0117, G0118, S0618, V5008, V5010, V5020, V5299 |
| 221 | Nasogastric tube | 9607 | 43752, 43753, 43754, 43755, 43756, 43757 |
| 222 | Blood and blood product transfusion | 9900, 9901, 9902, 9903, 9904, 9905, 9906, 9907, 9908, 9909 | 0692T, 36430, 36440, 36450, 36455, 36456, 36511, 36512, 36513, 36514, 36516, 36522, C9507, G0465, P9010, P9011, P9012, P9016, P9017, P9019, P9020, P9021, P9022, P9023, P9025, P9026, P9031, P9032, P9033, P9034, P9035, P9036, P9037, P9038, P9039, P9040, P9041, P9043, P9045, P9046, P9047, P9048, P9050, P9051, P9052, P9053, P9054, P9055, P9056, P9057, P9058, P9059, P9060, P9070, P9071, P9073, P9099, P9100, S2142 |
| 223 | Enteral and parenteral nutrition | 966, 9915 | B4102, B4103, B4104, B4149, B4150, B4152, B4153, B4154, B4155, B4157, B4158, B4159, B4160, B4161, B4162, B4164, B4168, B4172, B4176, B4178, B4180, B4185, B4187, B4189, B4193, B4197, B4199, B4216, B4220, B4222, B4224, B5000, B5100, B5200, S9432, S9433 |
| 224 | Cancer chemotherapy | 0010, 0015, 1770, 9925, 9928 | 0708T, 0709T, 51720, 61517, 96401, 96402, 96405, 96406, 96409, 96411, 96413, 96415, 96416, 96417, 96420, 96422, 96423, 96425, 96440, 96446, 96450, 96542, 96549, C9087, G0498, Q0083, Q0084, Q0085, S2107 |
| 225 | Conversion of cardiac rhythm | 9960, 9961, 9962, 9963, 9964, 9969 | 92961, 93650, 93653, 93654, 93655, 93656, 93657 |
| 226 | Other diagnostic radiology and related techniques | 0033, 0034, 0035, 0039, 3823, 3824, 3825, 8701, 8702, 8704, 8705, 8706, 8707, 8708, 8709, 8711, 8712, 8713, 8714, 8715, 8716, 8717, 8722, 8723, 8724, 8729, 8731, 8732, 8733, 8734, 8735, 8738, 8739, 8742, 8743, 8749, 8751, 8752, 8754, 8759, 8763, 8765, 8766, 8769, 8772, 8774, 8775, 8776, 8777, 8778, 8779, 8781, 8782, 8783, 8784, 8785, 8789, 8791, 8792, 8793, 8794, 8795, 8799, 8802, 8803, 8804, 8809, 8811, 8812, 8813, 8814, 8815, 8816, 8819, 8821, 8822, 8823, 8824, 8825, 8826, 8827, 8828, 8829, 8831, 8832, 8833, 8834, 8835, 8836, 8837, 8839, 8859, 8881, 8882, 8883, 8884, 8885, 8886, 8889, 8890, 8898 | 0054T, 0055T, 0347T, 0348T, 0349T, 0350T, 0422T, 21116, 23350, 24220, 25246, 27093, 27095, 27096, 27369, 27648, 36598, 38200, 38790, 38792, 42550, 47531, 47532, 49465, 50430, 50431, 54230, 55300, 62290, 62291, 68850, 70100, 70110, 70120, 70130, 70134, 70140, 70150, 70160, 70170, 70190, 70200, 70210, 70220, 70240, 70250, 70260, 70300, 70310, 70320, 70328, 70330, 70332, 70350, 70355, 70360, 70370, 70380, 70390, 71100, 71101, 71110, 71111, 71120, 71130, 72020, 72040, 72050, 72052, 72070, 72072, 72074, 72080, 72081, 72082, 72083, 72084, 72100, 72110, 72114, 72120, 72170, 72190, 72200, 72202, 72220, 73000, 73010, 73020, 73030, 73040, 73050, 73060, 73070, 73080, 73085, 73090, 73092, 73100, 73110, 73115, 73120, 73130, 73140, 73501, 73502, 73503, 73521, 73522, 73523, 73525, 73551, 73552, 73560, 73562, 73564, 73565, 73580, 73590, 73592, 73600, 73610, 73615, 73620, 73630, 73650, 73660, 74018, 74019, 74021, 74022, 74190, 74210, 74290, 74355, 74360, 74363, 74415, 74420, 74425, 74430, 74440, 74445, 74450, 74455, 74470, 74485, 74710, 74740, 74742, 74775, 75801, 75803, 75805, 75807, 75809, 75893, 75901, 75902, 75970, 75984, 75989, 76000, 76010, 76080, 76098, 76100, 76120, 76125, 76140, 76145, 76376, 76377, 76390, 76496, 76499, 77001, 77002, 77003, 77071, 77072, 77073, 77074, 77075, 77076, 77077, 77080, 77081, 77085, 77086, 77089, 77090, 77091, 77092, 94772, C8937, C9733, C9734, C9756, C9776, G0130, Q0092 |
| 227 | Consultation, evaluation, and preventative care | 0058, 0059, 0067, 0068, 0069, 8901, 8902, 8903, 8904, 8905, 8906, 8907, 8908, 8909, 8910, 8911, 8912, 8913, 8915, 8916, 8917, 8918, 8919, 8921, 8922, 8923, 8924, 8925, 8926, 8931, 8932, 8933, 8934, 8935, 8936, 8937, 8938, 8939, 8945, 8946, 8947, 8948, 8949, 8950, 8953, 8955, 8956, 8957, 8958, 8959, 8961, 8962, 8963, 8966, 8967, 8968, 8969, 897, 898 | 0358T, 0403T, 0591T, 0592T, 0593T, 90989, 90993, 93793, 94780, 94781, 96040, 99024, 99026, 99027, 99050, 99051, 99053, 99056, 99058, 99060, 99078, 99091, 99170, 99202, 99203, 99204, 99205, 99211, 99212, 99213, 99214, 99215, 99217, 99218, 99219, 99220, 99221, 99222, 99223, 99224, 99225, 99226, 99231, 99232, 99233, 99234, 99235, 99236, 99238, 99239, 99241, 99242, 99243, 99244, 99245, 99251, 99252, 99253, 99254, 99255, 99281, 99282, 99283, 99284, 99285, 99288, 99291, 99292, 99356, 99357, 99358, 99359, 99360, 99366, 99367, 99368, 99381, 99382, 99383, 99384, 99385, 99386, 99387, 99391, 99392, 99393, 99394, 99395, 99396, 99397, 99401, 99402, 99403, 99404, 99406, 99407, 99411, 99412, 99415, 99416, 99417, 99424, 99425, 99426, 99427, 99429, 99437, 99439, 99450, 99455, 99456, 99460, 99461, 99462, 99463, 99464, 99465, 99466, 99467, 99468, 99469, 99471, 99472, 99475, 99476, 99477, 99478, 99479, 99480, 99485, 99486, 99487, 99489, 99490, 99491, 99497, 99498, 99499, G0108, G0109, G0177, G0245, G0246, G0250, G0270, G0271, G0296, G0372, G0378, G0379, G0380, G0381, G0382, G0383, G0384, G0390, G0402, G0420, G0421, G0438, G0439, G0445, G0446, G0447, G0451, G0454, G0463, G0466, G0467, G0468, G0473, G0506, G0511, G0513, G0514, G2012, G2076, G2077, G2082, G2083, G2212, G9156, S0220, S0221, S0250, S0260, S0265, S0280, S0281, S0302, S0311, S0315, S0316, S0317, S0353, S0354, S0610, S0612, S0613, S0622, S3005, S9117, S9140, S9141, T1015, V5364 |
| 228 | Prophylactic vaccinations and inoculations | 9931, 9932, 9933, 9934, 9935, 9936, 9937, 9938, 9939, 9941, 9942, 9943, 9944, 9945, 9946, 9947, 9948, 9951, 9952, 9953, 9954, 9955, 9956, 9957, 9958, 9959 | 0001A, 0002A, 0003A, 0004A, 0011A, 0012A, 0013A, 0021A, 0022A, 0031A, 0034A, 0041A, 0042A, 0051A, 0052A, 0053A, 0054A, 0064A, 0071A, 0072A, 0073A, 0094A, 90281, 90283, 90284, 90287, 90288, 90291, 90296, 90371, 90375, 90376, 90377, 90378, 90384, 90385, 90386, 90389, 90393, 90396, 90399, 90460, 90461, 90471, 90472, 90473, 90474, 90476, 90477, 90581, 90585, 90586, 90587, 90619, 90620, 90621, 90625, 90626, 90627, 90630, 90632, 90633, 90634, 90636, 90644, 90647, 90648, 90649, 90650, 90651, 90653, 90654, 90655, 90656, 90657, 90658, 90660, 90661, 90662, 90664, 90666, 90667, 90668, 90670, 90671, 90672, 90673, 90674, 90675, 90676, 90677, 90680, 90681, 90682, 90685, 90686, 90687, 90688, 90689, 90690, 90691, 90694, 90696, 90697, 90698, 90700, 90702, 90707, 90710, 90713, 90714, 90715, 90716, 90717, 90723, 90732, 90733, 90734, 90736, 90738, 90739, 90740, 90743, 90744, 90746, 90747, 90748, 90749, 90750, 90756, 90758, 90759, 91300, 91301, 91302, 91303, 91304, 91305, 91306, 91307, 91309, 95115, 95117, 95120, 95125, 95130, 95131, 95132, 95133, 95134, 95144, 95145, 95146, 95147, 95148, 95149, 95165, G0008, G0009, G0010, M0201, M0220, M0221, M0240, M0241, Q2034, Q2035, Q2036, Q2037, Q2038, Q2039 |
| 229 | Nonoperative removal of foreign body | 9801, 9802, 9803, 9804, 9805, 9811, 9812, 9813, 9814, 9815, 9816, 9817, 9818, 9819, 9820, 9821, 9822, 9823, 9824, 9825, 9826, 9827, 9828, 9829 | 30300, 30320, 31511, 31577, 31635, 40804, 40805, 42809, 43194, 43215, 44363, 44390, 45307, 45332, 45379, 46608, 57415, 58562, 65205, 65220, 65222, 65235, 65260, 69200 |
| 230 | Extracorporeal shock wave, other than urinary | 9852, 9859 | 872, 873, G0279, S9034 |
| 231 | Other therapeutic procedures | 0001, 0002, 0003, 0009, 0011, 0012, 0013, 0014, 0016, 0017, 0018, 0019, 0091, 0092, 0093, 0094, 0095, 0096, 0110, 0116, 0117, 1741, 1742, 1743, 1744, 1745, 1749, 1762, 1769, 1781, 3898, 3899, 923, 9230, 9231, 9232, 9233, 9239, 9393, 9395, 9397, 9398, 9657, 9658, 9659, 9715, 9716, 9729, 9738, 9739, 9779, 9781, 9783, 9784, 9785, 9787, 9789, 9910, 9911, 9912, 9913, 9914, 9916, 9917, 9918, 9919, 9920, 9921, 9922, 9923, 9924, 9926, 9927, 9929, 9971, 9972, 9973, 9974, 9975, 9976, 9977, 9978, 9979, 9981, 9982, 9983, 9984, 9985, 9986, 9988, 9991, 9992, 9998, 9999 | 0213T, 0214T, 0215T, 0216T, 0217T, 0218T, 0232T, 0342T, 0600T, 0601T, 15850, 15851, 20526, 36299, 69990, 90951, 90952, 90953, 90954, 90955, 90956, 90957, 90958, 90959, 90960, 90961, 90962, 90967, 90968, 90969, 90970, 95180, 95199, 96360, 96361, 96365, 96366, 96367, 96368, 96369, 96370, 96371, 96372, 96373, 96374, 96375, 96376, 96379, 99183, 99184, G0277, G0293, G0294, G0460, G0501, G2000, G2021, M0222, M0223, M0243, M0244, M0245, M0246, M0247, M0248, M0249, M0250, Q0081, S0630, S8948, S9056, S9474 |
| 232 | Anesthesia | - | 00100, 102, 103, 104, 00120, 00124, 00126, 00140, 00142, 144, 145, 147, 148, 00160, 00162, 00164, 00170, 00172, 00174, 00176, 00190, 00192, 210, 211, 212, 214, 215, 216, 00218, 00220, 00222, 00300, 00320, 00322, 00326, 00350, 00352, 00400, 00402, 00404, 00406, 00410, 00450, 00454, 00470, 00472, 00474, 00500, 00520, 00522, 00524, 528, 529, 00530, 00532, 00534, 00537, 00539, 540, 541, 542, 00546, 00548, 00550, 560, 561, 562, 563, 566, 567, 00580, 00600, 00604, 00620, 625, 626, 00630, 00632, 00635, 00640, 00670, 00700, 00702, 730, 731, 732, 00750, 00752, 00754, 00756, 00770, 00790, 00792, 00794, 796, 797, 00800, 00802, 811, 812, 813, 00820, 00830, 00832, 00834, 00836, 00840, 00842, 00844, 00846, 00848, 00851, 00860, 00862, 864, 865, 866, 00868, 00870, 00880, 00882, 00902, 00904, 00906, 00908, 00910, 00912, 00914, 00916, 00918, 920, 921, 922, 00924, 00926, 00928, 00930, 00932, 00934, 00936, 00938, 00940, 00942, 00944, 00948, 00950, 00952, 01112, 01120, 01130, 01140, 01150, 01160, 01170, 01173, 01200, 01202, 01210, 01212, 1214, 1215, 01220, 01230, 01232, 01234, 01250, 01260, 01270, 01272, 01274, 01320, 01340, 01360, 01380, 01382, 01390, 01392, 01400, 01402, 01404, 01420, 01430, 01432, 01440, 01442, 01444, 01462, 01464, 01470, 01472, 01474, 01480, 01482, 01484, 01486, 01490, 01500, 01502, 01520, 01522, 01610, 01620, 01622, 01630, 01634, 01636, 01638, 01650, 01652, 01654, 01656, 01670, 01680, 01710, 01712, 01714, 01716, 01730, 01732, 01740, 01742, 01744, 01756, 01758, 01760, 01770, 01772, 01780, 01782, 01810, 01820, 01829, 01830, 01832, 01840, 01842, 01844, 01850, 01852, 01860, 01916, 01920, 01922, 1924, 1925, 1926, 1930, 1931, 1932, 1933, 1937, 1938, 1939, 1940, 1941, 1942, 1951, 1952, 1953, 01958, 1960, 1961, 1962, 1963, 1965, 1966, 1967, 1968, 1969, 1990, 1991, 1992, 01996, 01999, 99100, 99116, 99135, 99140, 99151, 99152, 99153, 99155, 99156, 99157, G0500 |
| 233 | Laboratory - Chemistry and Hematology | - | 0002U, 0038U, 0052U, 0061U, 0066U, 0077U, 0095U, 0119U, 0268U, 0269U, 0270U, 0271U, 0272U, 0273U, 0274U, 0275U, 0276U, 0277U, 0278U, 0279U, 0280U, 0281U, 0303U, 0304U, 0305U, 0316U, 80047, 80048, 80050, 80051, 80053, 80055, 80069, 80081, 82009, 82010, 82013, 82016, 82017, 82024, 82030, 82040, 82075, 82077, 82085, 82088, 82103, 82104, 82105, 82106, 82107, 82108, 82135, 82136, 82139, 82140, 82143, 82150, 82154, 82157, 82160, 82163, 82164, 82172, 82180, 82190, 82239, 82240, 82247, 82248, 82252, 82261, 82270, 82274, 82286, 82300, 82306, 82308, 82310, 82330, 82331, 82340, 82355, 82360, 82365, 82370, 82373, 82374, 82375, 82376, 82378, 82379, 82380, 82382, 82383, 82384, 82387, 82390, 82397, 82415, 82435, 82436, 82438, 82441, 82465, 82480, 82482, 82485, 82495, 82507, 82523, 82525, 82528, 82530, 82533, 82540, 82542, 82550, 82552, 82553, 82554, 82565, 82570, 82575, 82585, 82595, 82600, 82607, 82608, 82610, 82615, 82626, 82627, 82633, 82634, 82638, 82642, 82652, 82657, 82658, 82664, 82668, 82670, 82671, 82672, 82677, 82679, 82681, 82693, 82696, 82705, 82710, 82715, 82725, 82726, 82728, 82731, 82735, 82746, 82747, 82757, 82759, 82760, 82775, 82776, 82777, 82784, 82785, 82787, 82820, 82930, 82938, 82941, 82943, 82945, 82946, 82947, 82948, 82950, 82951, 82952, 82955, 82960, 82962, 82963, 82965, 82977, 82978, 82979, 82985, 83001, 83002, 83003, 83006, 83009, 83010, 83012, 83013, 83014, 83020, 83021, 83026, 83030, 83033, 83036, 83037, 83045, 83050, 83051, 83060, 83065, 83068, 83069, 83070, 83080, 83088, 83090, 83150, 83491, 83497, 83498, 83500, 83505, 83516, 83518, 83519, 83520, 83521, 83525, 83527, 83528, 83529, 83540, 83550, 83570, 83582, 83586, 83593, 83605, 83615, 83625, 83630, 83631, 83632, 83633, 83655, 83661, 83662, 83663, 83664, 83670, 83690, 83695, 83698, 83700, 83701, 83704, 83718, 83719, 83721, 83722, 83727, 83735, 83775, 83785, 83789, 83825, 83835, 83857, 83861, 83864, 83872, 83873, 83874, 83876, 83880, 83883, 83915, 83916, 83918, 83919, 83921, 83930, 83935, 83937, 83945, 83950, 83951, 83970, 83986, 83987, 83992, 83993, 84030, 84035, 84060, 84066, 84075, 84078, 84080, 84081, 84085, 84087, 84100, 84105, 84106, 84110, 84112, 84119, 84120, 84126, 84132, 84133, 84134, 84135, 84138, 84140, 84143, 84144, 84145, 84146, 84150, 84152, 84153, 84154, 84155, 84156, 84157, 84160, 84163, 84165, 84166, 84181, 84182, 84202, 84203, 84206, 84207, 84210, 84220, 84228, 84233, 84234, 84235, 84238, 84244, 84252, 84255, 84260, 84270, 84275, 84285, 84295, 84300, 84302, 84305, 84307, 84311, 84315, 84375, 84376, 84377, 84378, 84379, 84392, 84402, 84403, 84410, 84425, 84430, 84431, 84432, 84436, 84437, 84439, 84442, 84443, 84445, 84446, 84449, 84450, 84460, 84466, 84478, 84479, 84480, 84481, 84482, 84484, 84485, 84488, 84490, 84510, 84512, 84520, 84525, 84540, 84545, 84550, 84560, 84577, 84578, 84580, 84583, 84585, 84586, 84588, 84590, 84591, 84597, 84600, 84620, 84630, 84681, 84702, 84703, 84704, 84830, 84999, 85002, 85004, 85007, 85008, 85009, 85013, 85014, 85018, 85025, 85027, 85032, 85041, 85044, 85045, 85046, 85048, 85049, 85055, 85060, 85130, 85170, 85175, 85210, 85220, 85230, 85240, 85244, 85245, 85246, 85247, 85250, 85260, 85270, 85280, 85290, 85291, 85292, 85293, 85300, 85301, 85302, 85303, 85305, 85306, 85307, 85335, 85337, 85345, 85347, 85348, 85360, 85362, 85366, 85370, 85378, 85379, 85380, 85384, 85385, 85390, 85396, 85397, 85400, 85410, 85415, 85420, 85421, 85441, 85445, 85460, 85461, 85475, 85520, 85525, 85530, 85536, 85540, 85547, 85549, 85555, 85557, 85576, 85597, 85598, 85610, 85611, 85612, 85613, 85635, 85651, 85652, 85660, 85670, 85675, 85705, 85730, 85732, 85810, 85999, 86015, 88319, 88738, 88740, 88741, 94664, 94680, 94681, 94690, 94760, 94761, 94762, E0620, G0103, G0306, G0307, G0432, G0433, G0435, P2028, P2033, P2038, S2120, S2150, S3620, S3630, S3655 |
| 234 | Pathology | - | 0001U, 0002M, 0003M, 0003U, 0004M, 0005U, 0006M, 0007M, 0009M, 0009U, 0010U, 0011M, 0012M, 0012U, 0013M, 0013U, 0014M, 0014U, 0015M, 0015U, 0016M, 0016U, 0017M, 0017U, 0018M, 0018U, 0019U, 0021U, 0022U, 0023U, 0026U, 0027U, 0028U, 0029U, 0030U, 0031U, 0032U, 0033U, 0034U, 0035U, 0036U, 0037U, 0040U, 0045U, 0046U, 0047U, 0048U, 0049U, 0050U, 0053U, 0056U, 0057U, 0060U, 0062U, 0063U, 0067U, 0069U, 0070U, 0071U, 0072U, 0073U, 0074U, 0075U, 0076U, 0078U, 0079U, 0080U, 0081U, 0083U, 0086U, 0087U, 0088U, 0089U, 0090U, 0091U, 0092U, 0094U, 0101U, 0102U, 0103U, 0105U, 0108U, 0111U, 0112U, 0113U, 0114U, 0116U, 0117U, 0118U, 0120U, 0129U, 0130U, 0131U, 0132U, 0133U, 0134U, 0135U, 0136U, 0137U, 0138U, 0153U, 0154U, 0155U, 0156U, 0157U, 0158U, 0159U, 0160U, 0161U, 0162U, 0163U, 0164U, 0165U, 0166U, 0167U, 0169U, 0170U, 0171U, 0172U, 0173U, 0174U, 0175U, 0176U, 0177U, 0178U, 0179U, 0180U, 0181U, 0182U, 0183U, 0184U, 0185U, 0186U, 0187U, 0188U, 0189U, 0190U, 0191U, 0192U, 0193U, 0194U, 0195U, 0196U, 0197U, 0198U, 0199U, 0200U, 0201U, 0203U, 0204U, 0205U, 0206U, 0207U, 0208U, 0209U, 0210U, 0211U, 0212U, 0213U, 0214U, 0215U, 0216U, 0217U, 0218U, 0220U, 0221U, 0222U, 0228U, 0229U, 0230U, 0231U, 0232U, 0233U, 0234U, 0235U, 0236U, 0237U, 0238U, 0239U, 0242U, 0243U, 0244U, 0245U, 0246U, 0247U, 0248U, 0249U, 0250U, 0251U, 0252U, 0253U, 0254U, 0255U, 0256U, 0257U, 0258U, 0259U, 0260U, 0261U, 0262U, 0263U, 0264U, 0265U, 0266U, 0267U, 0282U, 0283U, 0284U, 0285U, 0286U, 0287U, 0288U, 0289U, 0290U, 0291U, 0292U, 0293U, 0294U, 0295U, 0296U, 0297U, 0298U, 0299U, 0300U, 0306U, 0307U, 0308U, 0309U, 0310U, 0312U, 0313U, 0314U, 0315U, 0317U, 0318U, 0319U, 0320U, 0322U, 0537T, 0540T, 80503, 80504, 80505, 80506, 81105, 81106, 81107, 81108, 81109, 81110, 81111, 81112, 81120, 81121, 81161, 81162, 81163, 81164, 81165, 81166, 81167, 81168, 81170, 81171, 81172, 81173, 81174, 81175, 81176, 81177, 81178, 81179, 81180, 81181, 81182, 81183, 81184, 81185, 81186, 81187, 81188, 81189, 81190, 81191, 81192, 81193, 81194, 81200, 81201, 81202, 81203, 81204, 81205, 81206, 81207, 81208, 81209, 81210, 81212, 81215, 81216, 81217, 81218, 81219, 81220, 81221, 81222, 81223, 81224, 81225, 81226, 81227, 81228, 81229, 81230, 81231, 81232, 81233, 81234, 81235, 81236, 81237, 81238, 81239, 81240, 81241, 81242, 81243, 81244, 81245, 81246, 81247, 81248, 81249, 81250, 81251, 81252, 81253, 81254, 81255, 81256, 81257, 81258, 81259, 81260, 81261, 81262, 81263, 81264, 81265, 81266, 81267, 81268, 81269, 81270, 81271, 81272, 81273, 81274, 81275, 81276, 81277, 81278, 81279, 81283, 81284, 81285, 81286, 81287, 81288, 81289, 81290, 81291, 81292, 81293, 81294, 81295, 81296, 81297, 81298, 81299, 81300, 81301, 81302, 81303, 81304, 81305, 81306, 81307, 81308, 81309, 81310, 81311, 81312, 81313, 81314, 81315, 81316, 81317, 81318, 81319, 81320, 81321, 81322, 81323, 81324, 81325, 81326, 81327, 81328, 81329, 81330, 81331, 81332, 81333, 81334, 81335, 81336, 81337, 81338, 81339, 81340, 81341, 81342, 81343, 81344, 81345, 81346, 81347, 81348, 81349, 81350, 81351, 81352, 81353, 81355, 81357, 81360, 81361, 81362, 81363, 81364, 81370, 81371, 81372, 81373, 81374, 81375, 81376, 81377, 81378, 81379, 81380, 81381, 81382, 81383, 81400, 81401, 81402, 81403, 81404, 81405, 81406, 81407, 81408, 81410, 81411, 81412, 81413, 81414, 81415, 81416, 81417, 81419, 81420, 81422, 81425, 81426, 81427, 81430, 81431, 81432, 81433, 81434, 81435, 81436, 81437, 81438, 81439, 81440, 81442, 81443, 81445, 81448, 81450, 81455, 81460, 81465, 81470, 81471, 81479, 81490, 81493, 81500, 81503, 81504, 81506, 81507, 81508, 81509, 81510, 81511, 81512, 81513, 81514, 81518, 81519, 81520, 81521, 81522, 81523, 81525, 81528, 81529, 81535, 81536, 81538, 81539, 81540, 81541, 81542, 81546, 81551, 81552, 81554, 81560, 81595, 81596, 81599, 86077, 86078, 86079, 88000, 88005, 88007, 88012, 88014, 88016, 88020, 88025, 88027, 88028, 88029, 88036, 88037, 88040, 88045, 88099, 88104, 88106, 88108, 88112, 88120, 88121, 88125, 88130, 88140, 88141, 88142, 88143, 88147, 88148, 88150, 88152, 88153, 88155, 88160, 88161, 88162, 88164, 88165, 88166, 88167, 88172, 88173, 88174, 88175, 88177, 88182, 88184, 88185, 88187, 88188, 88189, 88199, 88300, 88302, 88304, 88305, 88307, 88309, 88311, 88312, 88313, 88314, 88321, 88323, 88325, 88329, 88331, 88332, 88333, 88334, 88341, 88342, 88344, 88346, 88348, 88350, 88355, 88356, 88358, 88360, 88361, 88362, 88363, 88364, 88365, 88366, 88367, 88368, 88369, 88371, 88372, 88373, 88374, 88375, 88377, 88380, 88381, 88387, 88388, 88399, 89240, G0124, G0141, G0143, G0144, G0145, G0147, G0148, G0452, P3000, P3001, Q0091, Q0111 |
| 235 | Other Laboratory | - | 0007U, 0011U, 0020U, 0024U, 0025U, 0039U, 0051U, 0054U, 0055U, 0058U, 0059U, 0064U, 0065U, 0082U, 0084U, 0093U, 0110U, 0121U, 0122U, 0123U, 0143U, 0144U, 0145U, 0146U, 0147U, 0148U, 0149U, 0150U, 0224U, 0226U, 0227U, 0538T, 0539T, 0564T, 36400, 36405, 36406, 36410, 36415, 36416, 36600, 80061, 80074, 80076, 80143, 80145, 80150, 80151, 80155, 80156, 80157, 80158, 80159, 80161, 80162, 80163, 80164, 80165, 80167, 80168, 80169, 80170, 80171, 80173, 80175, 80176, 80177, 80178, 80179, 80180, 80181, 80183, 80184, 80185, 80186, 80187, 80188, 80189, 80190, 80192, 80193, 80194, 80195, 80197, 80198, 80199, 80200, 80201, 80202, 80203, 80204, 80210, 80220, 80230, 80235, 80280, 80285, 80299, 80305, 80306, 80307, 80320, 80321, 80322, 80323, 80324, 80325, 80326, 80327, 80328, 80329, 80330, 80331, 80332, 80333, 80334, 80335, 80336, 80337, 80338, 80339, 80340, 80341, 80342, 80343, 80344, 80345, 80346, 80347, 80348, 80349, 80350, 80351, 80352, 80353, 80354, 80355, 80356, 80357, 80358, 80359, 80360, 80361, 80362, 80363, 80364, 80365, 80366, 80367, 80368, 80369, 80370, 80371, 80372, 80373, 80374, 80375, 80376, 80377, 80400, 80402, 80406, 80408, 80410, 80412, 80414, 80415, 80416, 80417, 80418, 80420, 80422, 80424, 80426, 80428, 80430, 80432, 80434, 80435, 80436, 80438, 80439, 82232, 82271, 82272, 82653, 82656, 86000, 86001, 86003, 86005, 86008, 86021, 86022, 86023, 86036, 86037, 86038, 86039, 86051, 86052, 86053, 86060, 86063, 86140, 86141, 86146, 86147, 86148, 86152, 86153, 86155, 86156, 86157, 86160, 86161, 86162, 86171, 86200, 86215, 86225, 86226, 86231, 86235, 86255, 86256, 86258, 86277, 86280, 86294, 86300, 86301, 86304, 86305, 86308, 86309, 86310, 86316, 86317, 86318, 86320, 86325, 86327, 86328, 86329, 86331, 86332, 86334, 86335, 86336, 86337, 86340, 86341, 86343, 86344, 86352, 86353, 86355, 86356, 86357, 86359, 86360, 86361, 86362, 86363, 86364, 86367, 86376, 86381, 86382, 86384, 86386, 86403, 86406, 86408, 86409, 86413, 86430, 86431, 86480, 86481, 86485, 86486, 86490, 86510, 86580, 86590, 86592, 86593, 86596, 86602, 86603, 86606, 86609, 86611, 86612, 86615, 86617, 86618, 86619, 86622, 86625, 86628, 86631, 86632, 86635, 86638, 86641, 86644, 86645, 86648, 86651, 86652, 86653, 86654, 86658, 86663, 86664, 86665, 86666, 86668, 86671, 86674, 86677, 86682, 86684, 86687, 86688, 86689, 86692, 86694, 86695, 86696, 86698, 86701, 86702, 86703, 86704, 86705, 86706, 86707, 86708, 86709, 86710, 86711, 86713, 86717, 86720, 86723, 86727, 86732, 86735, 86738, 86741, 86744, 86747, 86750, 86753, 86756, 86757, 86759, 86762, 86765, 86768, 86769, 86771, 86774, 86777, 86778, 86780, 86784, 86787, 86788, 86789, 86790, 86793, 86794, 86800, 86803, 86804, 86805, 86806, 86807, 86808, 86828, 86829, 86830, 86831, 86832, 86833, 86834, 86835, 86849, 86850, 86860, 86870, 86880, 86885, 86886, 86890, 86891, 86900, 86901, 86902, 86904, 86905, 86906, 86910, 86911, 86920, 86921, 86922, 86923, 86927, 86930, 86931, 86932, 86940, 86941, 86945, 86950, 86960, 86965, 86970, 86971, 86972, 86975, 86976, 86977, 86978, 86985, 86999, 87003, 87209, 87493, 87634, 87635, 87662, 88240, 88241, 88271, 88272, 88273, 88274, 88275, 88720, 88749, 89049, 89050, 89051, 89055, 89220, 89230, 89258, 89259, 89335, 89337, 89342, 89343, 89344, 89346, 89352, 89353, 89354, 89356, 89398, 93702, 99195, C9803, G0027, G0327, G0328, G0471, G0472, G0480, G0481, G0482, G0483, G0499, G0659, G2023, G2024, G9143, P2029, P2031, Q3031, S3645, S3650, S3652, S3708, S3800, S3840, S3841, S3842, S3844, S3845, S3846, S3849, S3850, S3852, S3853, S3854, S3861, S3865, S3866, S3870, S4027, S4030, S4031, S9529, U0001, U0002, U0003, U0004, U0005 |
| 236 | Nonhospital-based care (e.g., home health care, hospice) | - | 90963, 90964, 90965, 90966, 93792, 94005, 94774, 94775, 94776, 94777, 97535, 99304, 99305, 99306, 99307, 99308, 99309, 99310, 99315, 99316, 99318, 99324, 99325, 99326, 99327, 99328, 99334, 99335, 99336, 99337, 99339, 99340, 99341, 99342, 99343, 99344, 99345, 99347, 99348, 99349, 99350, 99374, 99375, 99377, 99378, 99379, 99380, 99483, 99500, 99501, 99502, 99503, 99504, 99505, 99506, 99507, 99509, 99510, 99511, 99512, 99600, 99601, 99602, G0068, G0069, G0070, G0076, G0077, G0078, G0079, G0080, G0081, G0082, G0083, G0084, G0085, G0086, G0087, G0151, G0152, G0153, G0155, G0156, G0157, G0158, G0159, G0160, G0161, G0162, G0179, G0180, G0181, G0248, G0249, G0299, G0300, G0337, G0490, G0493, G0494, G0495, G0496, G9477, G9478, G9479, G9481, G9482, G9483, G9484, G9485, G9486, G9487, G9488, G9489, G9978, G9979, G9980, G9981, G9982, G9983, G9984, G9985, G9986, Q5001, Q5002, Q5003, Q5004, Q5005, Q5006, Q5007, Q5008, Q5009, Q5010, Q9001, Q9002, Q9003, Q9004, S0255, S0270, S0271, S0272, S0273, S0274, S3601, S5035, S5036, S5108, S5109, S5110, S5111, S5115, S5116, S5120, S5121, S5125, S5126, S5130, S5131, S5135, S5136, S5150, S5151, S5170, S5175, S5180, S5181, S5185, S5199, S5497, S5498, S5501, S5502, S5517, S5518, S5520, S5521, S5522, S5523, S9061, S9097, S9098, S9122, S9123, S9124, S9125, S9126, S9128, S9129, S9131, S9208, S9209, S9211, S9212, S9213, S9214, S9325, S9326, S9327, S9328, S9329, S9330, S9331, S9335, S9336, S9338, S9339, S9340, S9341, S9342, S9343, S9345, S9346, S9347, S9348, S9349, S9351, S9353, S9355, S9357, S9359, S9361, S9363, S9364, S9365, S9366, S9367, S9368, S9370, S9372, S9373, S9374, S9375, S9376, S9377, S9379, S9490, S9494, S9497, S9500, S9501, S9502, S9503, S9504, S9537, S9538, S9542, S9558, S9559, S9560, S9562, S9590, S9810, T1005, T1019, T1020, T1021, T1022, T1028, T1029, T1030, T1031, T2042, T2043, T2044, T2045, T2046, T2047 |
| 237 | Ancillary Services | - | 0559T, 0560T, 0561T, 0562T, 0691T, 0693T, 0694T, 0710T, 0711T, 0712T, 0713T, 79999, 95170, 96521, 96522, 96523, 97802, 97803, 97804, 98960, 98961, 98962, 99000, 99001, 99002, 99071, 99075, 99080, 99190, 99191, 99192, 99199, 99605, 99606, 99607, G0128, G0175, G0176, G0182, G9473, G9474, G9475, G9476, H0041, H0042, H0043, H0044, H2021, H2022, H2023, H2024, S0201, S0207, S0208, S0257, S0310, S0340, S0341, S0342, S0395, S2900, S5100, S5101, S5102, S5105, S5140, S5141, S5145, S5146, S5190, S9083, S9088, S9127, S9145, S9401, S9430, S9436, S9437, S9438, S9439, S9441, S9442, S9443, S9444, S9445, S9446, S9447, S9449, S9451, S9452, S9453, S9454, S9455, S9460, S9465, S9470, S9482, S9900, S9901, S9970, S9976, S9977, S9981, S9982, S9986, S9988, S9989, S9990, S9991, S9994, S9996, S9999, T1000, T1001, T1002, T1003, T1004, T1013, T1016, T1017, T1018, T1023, T1024, T1025, T1026, T1027, T2010, T2011, T2012, T2013, T2014, T2015, T2016, T2017, T2018, T2019, T2020, T2021, T2022, T2023, T2024, T2025, T2026, T2027, T2030, T2031, T2032, T2033, T2034, T2035, T2036, T2037, T2038, T2040, T2041 |
| 238 | Infertility Services | - | S4011, S4013, S4014, S4015, S4016, S4017, S4018, S4020, S4021, S4022, S4023, S4025, S4026, S4028, S4035, S4037, S4040, S4042 |
| 239 | Transportation - patient, provider, equipment | - | 99082, A0021, A0080, A0090, A0100, A0110, A0120, A0130, A0140, A0160, A0170, A0180, A0190, A0200, A0210, A0225, A0380, A0382, A0384, A0390, A0392, A0394, A0396, A0398, A0420, A0422, A0424, A0425, A0426, A0427, A0428, A0429, A0430, A0431, A0432, A0433, A0434, A0435, A0436, A0888, A0998, A0999, A9901, P9603, P9604, R0070, R0075, R0076, S0209, S0215, S9381, S9960, S9961, S9975, S9992, T2001, T2002, T2003, T2004, T2005, T2007, T2049 |
| 240 | Medications (Injections, infusions and other forms) | - | 0444T, 0445T, 0465T, 96377, A4802, A9150, A9152, A9153, A9513, A9575, A9581, A9582, A9583, A9589, A9604, C8957, C9046, C9047, C9084, C9085, C9086, C9088, C9089, C9090, C9091, C9092, C9093, C9113, C9248, C9254, C9257, C9285, C9290, C9293, C9399, C9460, C9462, C9482, C9483, C9488, G0333, G0429, G2078, G2079, G3001, G9147, J0120, J0121, J0122, J0129, J0130, J0131, J0132, J0133, J0135, J0153, J0171, J0172, J0178, J0179, J0180, J0185, J0190, J0200, J0202, J0205, J0207, J0210, J0215, J0219, J0220, J0221, J0222, J0223, J0224, J0248, J0256, J0257, J0270, J0275, J0278, J0280, J0282, J0285, J0287, J0288, J0289, J0290, J0291, J0295, J0300, J0330, J0348, J0350, J0360, J0364, J0365, J0380, J0390, J0395, J0400, J0401, J0456, J0461, J0470, J0475, J0476, J0480, J0485, J0490, J0491, J0500, J0515, J0517, J0520, J0558, J0561, J0565, J0567, J0570, J0571, J0572, J0573, J0574, J0575, J0583, J0584, J0585, J0586, J0587, J0588, J0591, J0592, J0593, J0594, J0595, J0596, J0597, J0598, J0599, J0600, J0604, J0606, J0610, J0620, J0630, J0636, J0637, J0638, J0640, J0641, J0642, J0670, J0690, J0691, J0692, J0694, J0695, J0696, J0697, J0698, J0699, J0702, J0706, J0710, J0712, J0713, J0714, J0715, J0716, J0717, J0720, J0725, J0735, J0740, J0741, J0742, J0743, J0744, J0745, J0770, J0775, J0780, J0791, J0795, J0800, J0834, J0840, J0841, J0850, J0875, J0878, J0879, J0881, J0882, J0883, J0884, J0885, J0887, J0888, J0890, J0894, J0895, J0896, J0897, J0945, J1000, J1020, J1030, J1040, J1050, J1071, J1094, J1095, J1096, J1097, J1100, J1110, J1120, J1130, J1160, J1162, J1165, J1170, J1180, J1190, J1200, J1201, J1205, J1212, J1230, J1240, J1245, J1250, J1260, J1265, J1267, J1270, J1290, J1300, J1301, J1303, J1305, J1320, J1322, J1324, J1325, J1327, J1330, J1335, J1364, J1380, J1410, J1426, J1427, J1428, J1429, J1430, J1435, J1436, J1437, J1438, J1439, J1442, J1443, J1444, J1445, J1447, J1448, J1450, J1451, J1452, J1453, J1454, J1455, J1457, J1458, J1459, J1460, J1554, J1555, J1556, J1557, J1558, J1559, J1560, J1561, J1562, J1566, J1568, J1569, J1570, J1571, J1572, J1573, J1575, J1580, J1595, J1599, J1600, J1602, J1610, J1620, J1626, J1627, J1628, J1630, J1631, J1632, J1640, J1642, J1644, J1645, J1650, J1652, J1655, J1670, J1675, J1700, J1710, J1720, J1726, J1729, J1730, J1738, J1740, J1741, J1742, J1743, J1744, J1745, J1746, J1750, J1756, J1786, J1790, J1800, J1810, J1815, J1817, J1823, J1826, J1830, J1833, J1835, J1840, J1850, J1885, J1890, J1930, J1931, J1940, J1943, J1944, J1945, J1950, J1951, J1952, J1953, J1955, J1956, J1960, J1980, J1990, J2001, J2010, J2020, J2060, J2062, J2150, J2170, J2175, J2180, J2182, J2185, J2186, J2210, J2212, J2248, J2250, J2260, J2265, J2270, J2274, J2278, J2280, J2300, J2310, J2315, J2320, J2323, J2325, J2326, J2350, J2353, J2354, J2355, J2357, J2358, J2360, J2370, J2400, J2405, J2406, J2407, J2410, J2425, J2426, J2430, J2440, J2460, J2469, J2501, J2502, J2503, J2504, J2506, J2507, J2510, J2513, J2515, J2540, J2543, J2545, J2547, J2550, J2560, J2562, J2590, J2597, J2650, J2670, J2675, J2680, J2690, J2700, J2704, J2710, J2720, J2724, J2725, J2730, J2760, J2765, J2770, J2778, J2780, J2783, J2785, J2786, J2787, J2788, J2790, J2791, J2792, J2793, J2794, J2795, J2796, J2797, J2798, J2800, J2805, J2810, J2820, J2840, J2850, J2860, J2910, J2916, J2920, J2930, J2940, J2941, J2950, J2993, J2995, J2997, J3000, J3010, J3030, J3031, J3032, J3060, J3070, J3090, J3095, J3101, J3105, J3110, J3111, J3121, J3145, J3230, J3240, J3241, J3243, J3245, J3246, J3250, J3260, J3262, J3265, J3280, J3285, J3300, J3301, J3302, J3303, J3304, J3305, J3310, J3315, J3316, J3320, J3350, J3355, J3357, J3358, J3360, J3364, J3365, J3370, J3380, J3385, J3396, J3397, J3398, J3399, J3400, J3410, J3411, J3415, J3420, J3430, J3465, J3470, J3471, J3472, J3473, J3475, J3480, J3485, J3486, J3489, J3490, J3520, J3530, J3535, J3570, J3590, J3591, J7030, J7040, J7042, J7050, J7060, J7070, J7100, J7110, J7120, J7121, J7131, J7168, J7169, J7170, J7175, J7177, J7178, J7179, J7180, J7181, J7182, J7183, J7185, J7186, J7187, J7188, J7189, J7190, J7191, J7192, J7193, J7194, J7195, J7196, J7197, J7198, J7199, J7200, J7201, J7202, J7203, J7204, J7205, J7207, J7208, J7209, J7210, J7211, J7212, J7294, J7295, J7296, J7297, J7298, J7300, J7301, J7304, J7306, J7307, J7308, J7309, J7310, J7311, J7312, J7313, J7314, J7315, J7316, J7318, J7320, J7321, J7322, J7323, J7324, J7325, J7326, J7327, J7328, J7329, J7330, J7331, J7332, J7336, J7340, J7342, J7345, J7351, J7352, J7402, J7500, J7501, J7502, J7503, J7504, J7505, J7507, J7508, J7509, J7510, J7511, J7512, J7513, J7515, J7516, J7517, J7518, J7520, J7525, J7527, J7599, J7604, J7605, J7606, J7607, J7608, J7609, J7610, J7611, J7612, J7613, J7614, J7615, J7620, J7622, J7624, J7626, J7627, J7628, J7629, J7631, J7632, J7633, J7634, J7635, J7636, J7637, J7638, J7639, J7640, J7641, J7642, J7643, J7644, J7645, J7647, J7648, J7649, J7650, J7657, J7658, J7659, J7660, J7665, J7667, J7668, J7669, J7670, J7674, J7676, J7677, J7680, J7681, J7682, J7683, J7684, J7685, J7686, J7699, J7799, J7999, J8498, J8499, J8501, J8510, J8515, J8520, J8521, J8530, J8540, J8560, J8562, J8565, J8597, J8600, J8610, J8650, J8655, J8670, J8700, J8705, J8999, J9000, J9015, J9017, J9019, J9020, J9021, J9022, J9023, J9025, J9027, J9030, J9032, J9033, J9034, J9035, J9036, J9037, J9039, J9040, J9041, J9042, J9043, J9044, J9045, J9047, J9050, J9055, J9057, J9060, J9061, J9065, J9070, J9071, J9098, J9100, J9118, J9119, J9120, J9130, J9144, J9145, J9150, J9151, J9153, J9155, J9160, J9165, J9171, J9173, J9175, J9176, J9177, J9178, J9179, J9181, J9185, J9190, J9198, J9200, J9201, J9202, J9203, J9204, J9205, J9206, J9207, J9208, J9209, J9210, J9211, J9212, J9213, J9214, J9215, J9216, J9217, J9218, J9219, J9223, J9225, J9226, J9227, J9228, J9229, J9230, J9245, J9246, J9247, J9250, J9260, J9261, J9262, J9263, J9264, J9266, J9267, J9268, J9269, J9270, J9271, J9272, J9273, J9280, J9281, J9285, J9293, J9295, J9299, J9301, J9302, J9303, J9304, J9305, J9306, J9307, J9308, J9309, J9311, J9312, J9313, J9316, J9317, J9318, J9319, J9320, J9325, J9328, J9330, J9340, J9348, J9349, J9351, J9352, J9353, J9354, J9355, J9356, J9357, J9358, J9359, J9360, J9370, J9371, J9390, J9395, J9400, J9600, J9999, K0672, M0075, Q0138, Q0139, Q0144, Q0161, Q0162, Q0163, Q0164, Q0166, Q0167, Q0169, Q0173, Q0174, Q0175, Q0177, Q0180, Q0181, Q0220, Q0221, Q0222, Q0240, Q0243, Q0244, Q0245, Q0247, Q0249, Q0510, Q0511, Q0512, Q0513, Q0514, Q0515, Q2009, Q2017, Q2026, Q2028, Q2041, Q2042, Q2043, Q2049, Q2050, Q2052, Q2053, Q2054, Q2055, Q3027, Q3028, Q4074, Q4081, Q4082, Q5101, Q5103, Q5104, Q5105, Q5106, Q5107, Q5108, Q5109, Q5110, Q5111, Q5112, Q5113, Q5114, Q5115, Q5116, Q5117, Q5118, Q5119, Q5120, Q5121, Q5122, Q5123, Q5124, Q9950, Q9953, Q9955, Q9956, Q9957, Q9991, Q9992, S0012, S0013, S0014, S0017, S0020, S0021, S0023, S0028, S0030, S0032, S0034, S0039, S0040, S0073, S0074, S0077, S0078, S0080, S0081, S0088, S0090, S0091, S0092, S0093, S0104, S0106, S0108, S0109, S0117, S0119, S0122, S0126, S0128, S0132, S0136, S0137, S0138, S0139, S0140, S0142, S0145, S0148, S0156, S0157, S0160, S0164, S0166, S0169, S0170, S0171, S0172, S0174, S0175, S0176, S0177, S0178, S0179, S0182, S0183, S0187, S0189, S0190, S0191, S0194, S0197, S2112, S2202, S3722, S4990, S4991, S4993, S4995, S5000, S5001, S5012, S5014, S5550, S5551, S5552, S5553, T1502, T1503 |
| 241 | Visual aids and other optical supplies | - | Q1004, Q1005, S0500, S0504, S0506, S0508, S0510, S0512, S0514, S0515, S0516, S0518, S0580, S0581, S0590, S0592, S0595, S0596, S9150, V2020, V2025, V2100, V2101, V2102, V2103, V2104, V2105, V2106, V2107, V2108, V2109, V2110, V2111, V2112, V2113, V2114, V2115, V2118, V2121, V2199, V2200, V2201, V2202, V2203, V2204, V2205, V2206, V2207, V2208, V2209, V2210, V2211, V2212, V2213, V2214, V2215, V2218, V2219, V2220, V2221, V2299, V2300, V2301, V2302, V2303, V2304, V2305, V2306, V2307, V2308, V2309, V2310, V2311, V2312, V2313, V2314, V2315, V2318, V2319, V2320, V2321, V2399, V2410, V2430, V2499, V2500, V2501, V2502, V2503, V2510, V2511, V2512, V2513, V2520, V2521, V2522, V2523, V2524, V2525, V2530, V2531, V2599, V2600, V2610, V2615, V2623, V2624, V2625, V2626, V2627, V2628, V2629, V2630, V2631, V2632, V2700, V2702, V2710, V2715, V2718, V2730, V2744, V2745, V2750, V2755, V2756, V2760, V2761, V2762, V2770, V2780, V2781, V2782, V2783, V2784, V2785, V2786, V2787, V2788, V2790, V2797, V2799 |
| 242 | Hearing devices and audiology supplies | - | L8690, L8691, V5011, V5014, V5030, V5040, V5050, V5060, V5070, V5080, V5090, V5095, V5100, V5110, V5120, V5130, V5140, V5150, V5160, V5171, V5172, V5181, V5190, V5200, V5211, V5212, V5213, V5214, V5215, V5221, V5230, V5240, V5241, V5242, V5243, V5244, V5245, V5246, V5247, V5248, V5249, V5250, V5251, V5252, V5253, V5254, V5255, V5256, V5257, V5258, V5259, V5260, V5261, V5262, V5263, V5264, V5265, V5266, V5267, V5268, V5269, V5270, V5271, V5272, V5273, V5274, V5275, V5281, V5282, V5283, V5284, V5285, V5286, V5287, V5288, V5289, V5290, V5298 |
| 243 | DME and supplies | - | 99070, 99072, A2001, A2002, A2003, A2004, A2005, A2006, A2007, A2008, A2009, A2010, A2011, A2012, A2013, A4100, A4206, A4207, A4208, A4209, A4210, A4211, A4212, A4213, A4215, A4216, A4217, A4218, A4220, A4221, A4222, A4223, A4224, A4225, A4226, A4230, A4231, A4232, A4233, A4234, A4235, A4236, A4238, A4244, A4245, A4246, A4247, A4248, A4250, A4252, A4253, A4255, A4256, A4257, A4258, A4259, A4261, A4262, A4263, A4264, A4265, A4266, A4267, A4268, A4269, A4270, A4280, A4281, A4282, A4283, A4284, A4285, A4286, A4290, A4300, A4301, A4305, A4306, A4310, A4311, A4312, A4313, A4314, A4315, A4316, A4320, A4321, A4322, A4326, A4327, A4328, A4330, A4331, A4332, A4333, A4334, A4335, A4336, A4337, A4338, A4340, A4344, A4346, A4349, A4351, A4352, A4353, A4354, A4355, A4356, A4357, A4358, A4360, A4361, A4362, A4363, A4364, A4366, A4367, A4368, A4369, A4371, A4372, A4373, A4375, A4376, A4377, A4378, A4379, A4380, A4381, A4382, A4383, A4384, A4385, A4387, A4388, A4389, A4390, A4391, A4392, A4393, A4394, A4395, A4396, A4398, A4399, A4400, A4402, A4404, A4405, A4406, A4407, A4408, A4409, A4410, A4411, A4412, A4413, A4414, A4415, A4416, A4417, A4418, A4419, A4420, A4421, A4422, A4423, A4424, A4425, A4426, A4427, A4428, A4429, A4430, A4431, A4432, A4433, A4434, A4435, A4436, A4437, A4450, A4452, A4453, A4455, A4456, A4458, A4459, A4461, A4463, A4465, A4467, A4470, A4480, A4481, A4483, A4490, A4495, A4500, A4510, A4520, A4550, A4553, A4554, A4555, A4556, A4557, A4558, A4559, A4561, A4562, A4563, A4565, A4566, A4570, A4575, A4580, A4590, A4595, A4600, A4601, A4602, A4604, A4605, A4606, A4608, A4611, A4612, A4613, A4614, A4615, A4616, A4617, A4618, A4619, A4620, A4623, A4624, A4625, A4626, A4627, A4628, A4629, A4630, A4633, A4634, A4635, A4636, A4637, A4638, A4639, A4640, A4641, A4642, A4648, A4649, A4650, A4651, A4652, A4653, A4657, A4660, A4663, A4670, A4671, A4672, A4673, A4674, A4680, A4690, A4706, A4707, A4708, A4709, A4714, A4719, A4720, A4721, A4722, A4723, A4724, A4725, A4726, A4728, A4730, A4736, A4737, A4740, A4750, A4755, A4760, A4765, A4766, A4770, A4771, A4772, A4773, A4774, A4860, A4870, A4890, A4911, A4913, A4918, A4927, A4928, A4929, A4930, A4931, A4932, A5051, A5052, A5053, A5054, A5055, A5056, A5057, A5061, A5062, A5063, A5071, A5072, A5073, A5081, A5082, A5083, A5093, A5102, A5105, A5112, A5113, A5114, A5120, A5121, A5122, A5126, A5131, A5200, A5500, A5501, A5503, A5504, A5505, A5506, A5507, A5508, A5510, A5512, A5513, A5514, A6000, A6010, A6011, A6021, A6022, A6023, A6024, A6025, A6154, A6196, A6197, A6198, A6199, A6203, A6204, A6205, A6206, A6207, A6208, A6209, A6210, A6211, A6212, A6213, A6214, A6215, A6216, A6217, A6218, A6219, A6220, A6221, A6222, A6223, A6224, A6228, A6229, A6230, A6231, A6232, A6233, A6234, A6235, A6236, A6237, A6238, A6239, A6240, A6241, A6242, A6243, A6244, A6245, A6246, A6247, A6248, A6250, A6251, A6252, A6253, A6254, A6255, A6256, A6257, A6258, A6259, A6260, A6261, A6262, A6266, A6402, A6403, A6404, A6407, A6410, A6411, A6412, A6413, A6441, A6442, A6443, A6444, A6445, A6446, A6447, A6448, A6449, A6450, A6451, A6452, A6453, A6454, A6455, A6456, A6457, A6460, A6461, A6501, A6502, A6503, A6504, A6505, A6506, A6507, A6508, A6509, A6510, A6511, A6512, A6513, A6530, A6531, A6532, A6533, A6534, A6535, A6536, A6537, A6538, A6539, A6540, A6541, A6544, A6545, A6549, A6550, A7000, A7001, A7002, A7003, A7004, A7005, A7006, A7007, A7008, A7009, A7010, A7012, A7013, A7014, A7015, A7016, A7017, A7018, A7020, A7025, A7026, A7027, A7028, A7029, A7030, A7031, A7032, A7033, A7034, A7035, A7036, A7037, A7038, A7039, A7040, A7041, A7044, A7045, A7046, A7047, A7048, A7501, A7502, A7503, A7504, A7505, A7506, A7507, A7508, A7509, A7520, A7521, A7522, A7523, A7524, A7525, A7526, A7527, A8000, A8001, A8002, A8003, A8004, A9155, A9180, A9270, A9272, A9273, A9274, A9275, A9276, A9277, A9278, A9279, A9280, A9281, A9282, A9283, A9284, A9285, A9286, A9291, A9300, A9500, A9501, A9502, A9503, A9504, A9505, A9507, A9508, A9509, A9510, A9512, A9515, A9516, A9517, A9520, A9521, A9524, A9526, A9527, A9528, A9529, A9530, A9531, A9532, A9536, A9537, A9538, A9539, A9540, A9541, A9542, A9543, A9546, A9547, A9548, A9550, A9551, A9552, A9553, A9554, A9555, A9556, A9557, A9558, A9559, A9560, A9561, A9562, A9563, A9564, A9566, A9567, A9568, A9569, A9570, A9571, A9572, A9574, A9576, A9577, A9578, A9579, A9580, A9584, A9585, A9586, A9587, A9588, A9590, A9591, A9592, A9593, A9594, A9595, A9597, A9598, A9600, A9606, A9698, A9699, A9700, A9900, A9999, B4034, B4035, B4036, B4081, B4082, B4083, B4087, B4088, B4100, B4105, B9002, B9004, B9006, B9998, B9999, C1052, C1062, C1713, C1714, C1715, C1716, C1717, C1719, C1721, C1722, C1724, C1725, C1726, C1727, C1728, C1729, C1730, C1731, C1732, C1733, C1734, C1748, C1750, C1751, C1752, C1753, C1754, C1755, C1756, C1757, C1758, C1759, C1760, C1761, C1762, C1763, C1764, C1765, C1766, C1767, C1768, C1769, C1770, C1771, C1772, C1773, C1776, C1777, C1778, C1779, C1780, C1781, C1782, C1783, C1784, C1785, C1786, C1787, C1788, C1789, C1813, C1814, C1815, C1816, C1817, C1818, C1819, C1820, C1821, C1822, C1823, C1824, C1825, C1830, C1831, C1832, C1833, C1839, C1840, C1841, C1842, C1849, C1874, C1875, C1876, C1877, C1878, C1880, C1881, C1882, C1883, C1884, C1885, C1886, C1887, C1888, C1889, C1891, C1892, C1893, C1894, C1895, C1896, C1897, C1898, C1899, C1900, C1982, C2596, C2613, C2614, C2615, C2616, C2617, C2618, C2619, C2620, C2621, C2622, C2623, C2624, C2625, C2626, C2627, C2628, C2629, C2630, C2631, C2634, C2635, C2636, C2637, C9067, C9250, C9352, C9353, C9354, C9355, C9356, C9358, C9359, C9360, C9361, C9362, C9363, C9364, C9898, C9899, E0100, E0105, E0110, E0111, E0112, E0113, E0114, E0116, E0117, E0118, E0130, E0135, E0140, E0141, E0143, E0144, E0147, E0148, E0149, E0153, E0154, E0155, E0156, E0157, E0158, E0159, E0160, E0161, E0162, E0163, E0165, E0167, E0168, E0170, E0171, E0172, E0175, E0181, E0182, E0184, E0185, E0186, E0187, E0188, E0189, E0190, E0191, E0193, E0194, E0196, E0197, E0198, E0199, E0200, E0202, E0203, E0205, E0210, E0215, E0217, E0218, E0221, E0225, E0231, E0232, E0235, E0236, E0239, E0240, E0241, E0242, E0243, E0244, E0245, E0246, E0247, E0248, E0249, E0250, E0251, E0255, E0256, E0260, E0261, E0265, E0266, E0270, E0271, E0272, E0273, E0274, E0275, E0276, E0277, E0280, E0290, E0291, E0292, E0293, E0294, E0295, E0296, E0297, E0300, E0301, E0302, E0303, E0304, E0305, E0310, E0315, E0316, E0325, E0326, E0328, E0329, E0350, E0352, E0370, E0371, E0372, E0373, E0424, E0425, E0430, E0431, E0433, E0434, E0435, E0439, E0440, E0441, E0442, E0443, E0444, E0445, E0446, E0447, E0455, E0457, E0459, E0462, E0465, E0466, E0467, E0470, E0471, E0472, E0480, E0481, E0482, E0483, E0484, E0485, E0486, E0487, E0500, E0550, E0555, E0560, E0561, E0562, E0565, E0570, E0572, E0574, E0575, E0580, E0585, E0600, E0601, E0602, E0603, E0604, E0605, E0606, E0607, E0610, E0615, E0616, E0617, E0618, E0619, E0621, E0625, E0627, E0629, E0630, E0635, E0636, E0637, E0638, E0639, E0640, E0641, E0642, E0650, E0651, E0652, E0655, E0656, E0657, E0660, E0665, E0666, E0667, E0668, E0669, E0670, E0671, E0672, E0673, E0675, E0676, E0691, E0692, E0693, E0694, E0700, E0705, E0710, E0720, E0730, E0731, E0740, E0744, E0745, E0746, E0747, E0748, E0749, E0755, E0760, E0761, E0762, E0764, E0765, E0766, E0769, E0770, E0776, E0779, E0780, E0781, E0782, E0783, E0784, E0785, E0786, E0787, E0791, E0830, E0840, E0849, E0850, E0855, E0856, E0860, E0870, E0890, E0900, E0910, E0911, E0912, E0920, E0930, E0935, E0936, E0940, E0941, E0942, E0944, E0945, E0946, E0947, E0948, E0950, E0951, E0952, E0953, E0954, E0955, E0956, E0957, E0958, E0959, E0960, E0961, E0966, E0967, E0968, E0969, E0970, E0971, E0973, E0974, E0978, E0980, E0981, E0982, E0983, E0984, E0985, E0986, E0988, E0990, E0992, E0994, E0995, E1002, E1003, E1004, E1005, E1006, E1007, E1008, E1009, E1010, E1011, E1012, E1014, E1015, E1016, E1017, E1018, E1020, E1028, E1029, E1030, E1031, E1035, E1036, E1037, E1038, E1039, E1050, E1060, E1070, E1083, E1084, E1085, E1086, E1087, E1088, E1089, E1090, E1092, E1093, E1100, E1110, E1130, E1140, E1150, E1160, E1161, E1170, E1171, E1172, E1180, E1190, E1195, E1200, E1220, E1221, E1222, E1223, E1224, E1225, E1226, E1227, E1228, E1229, E1230, E1231, E1232, E1233, E1234, E1235, E1236, E1237, E1238, E1239, E1240, E1250, E1260, E1270, E1280, E1285, E1290, E1295, E1296, E1297, E1298, E1300, E1310, E1352, E1353, E1354, E1355, E1356, E1357, E1358, E1372, E1390, E1391, E1392, E1399, E1405, E1406, E1500, E1510, E1520, E1530, E1540, E1550, E1560, E1570, E1575, E1580, E1590, E1592, E1594, E1600, E1610, E1615, E1620, E1625, E1629, E1630, E1632, E1634, E1635, E1636, E1637, E1639, E1699, E1700, E1701, E1702, E1800, E1801, E1802, E1805, E1806, E1810, E1811, E1812, E1815, E1816, E1818, E1820, E1821, E1825, E1830, E1831, E1840, E1841, E1902, E2000, E2100, E2101, E2102, E2120, E2201, E2202, E2203, E2204, E2205, E2206, E2207, E2208, E2209, E2210, E2211, E2212, E2213, E2214, E2215, E2216, E2217, E2218, E2219, E2220, E2221, E2222, E2224, E2225, E2226, E2227, E2228, E2230, E2231, E2291, E2292, E2293, E2294, E2295, E2300, E2301, E2310, E2311, E2312, E2313, E2321, E2322, E2323, E2324, E2325, E2326, E2327, E2328, E2329, E2330, E2331, E2340, E2341, E2342, E2343, E2351, E2358, E2359, E2360, E2361, E2362, E2363, E2364, E2365, E2366, E2367, E2368, E2369, E2370, E2371, E2372, E2373, E2374, E2375, E2376, E2377, E2378, E2381, E2382, E2383, E2384, E2385, E2386, E2387, E2388, E2389, E2390, E2391, E2392, E2394, E2395, E2396, E2397, E2398, E2402, E2500, E2502, E2504, E2506, E2508, E2510, E2511, E2512, E2599, E2601, E2602, E2603, E2604, E2605, E2606, E2607, E2608, E2609, E2610, E2611, E2612, E2613, E2614, E2615, E2616, E2617, E2619, E2620, E2621, E2622, E2623, E2624, E2625, E2626, E2627, E2628, E2629, E2630, E2631, E2632, E2633, E8000, E8001, E8002, K0001, K0002, K0003, K0004, K0005, K0006, K0007, K0008, K0009, K0010, K0011, K0012, K0013, K0014, K0015, K0017, K0018, K0019, K0020, K0037, K0038, K0039, K0040, K0041, K0042, K0043, K0044, K0045, K0046, K0047, K0050, K0051, K0052, K0053, K0056, K0065, K0069, K0070, K0071, K0072, K0073, K0077, K0098, K0105, K0108, K0195, K0455, K0462, K0552, K0553, K0554, K0601, K0602, K0603, K0604, K0605, K0606, K0607, K0608, K0609, K0669, K0730, K0733, K0738, K0739, K0740, K0743, K0744, K0745, K0746, K0800, K0801, K0802, K0806, K0807, K0808, K0812, K0813, K0814, K0815, K0816, K0820, K0821, K0822, K0823, K0824, K0825, K0826, K0827, K0828, K0829, K0830, K0831, K0835, K0836, K0837, K0838, K0839, K0840, K0841, K0842, K0843, K0848, K0849, K0850, K0851, K0852, K0853, K0854, K0855, K0856, K0857, K0858, K0859, K0860, K0861, K0862, K0863, K0864, K0868, K0869, K0870, K0871, K0877, K0878, K0879, K0880, K0884, K0885, K0886, K0890, K0891, K0898, K0899, K0900, K1001, K1002, K1003, K1004, K1005, K1006, K1007, K1009, K1013, K1014, K1015, K1016, K1017, K1018, K1019, K1020, K1021, K1022, K1023, K1024, K1025, K1026, K1027, K1028, K1029, K1030, K1031, K1032, K1033, L0112, L0113, L0120, L0130, L0140, L0150, L0160, L0170, L0172, L0174, L0180, L0190, L0200, L0220, L0450, L0452, L0454, L0455, L0456, L0457, L0458, L0460, L0462, L0464, L0466, L0467, L0468, L0469, L0470, L0472, L0480, L0482, L0484, L0486, L0488, L0490, L0491, L0492, L0621, L0622, L0623, L0624, L0625, L0626, L0627, L0628, L0629, L0630, L0631, L0632, L0633, L0634, L0635, L0636, L0637, L0638, L0639, L0640, L0641, L0642, L0643, L0648, L0649, L0650, L0651, L0700, L0710, L0810, L0820, L0830, L0859, L0861, L0970, L0972, L0974, L0976, L0978, L0980, L0982, L0984, L0999, L1000, L1001, L1005, L1010, L1020, L1025, L1030, L1040, L1050, L1060, L1070, L1080, L1085, L1090, L1100, L1110, L1120, L1200, L1210, L1220, L1230, L1240, L1250, L1260, L1270, L1280, L1290, L1300, L1310, L1499, L1600, L1610, L1620, L1630, L1640, L1650, L1652, L1660, L1680, L1685, L1686, L1690, L1700, L1710, L1720, L1730, L1755, L1810, L1812, L1820, L1830, L1831, L1832, L1833, L1834, L1836, L1840, L1843, L1844, L1845, L1846, L1847, L1848, L1850, L1851, L1852, L1860, L1900, L1902, L1904, L1906, L1907, L1910, L1920, L1930, L1932, L1940, L1945, L1950, L1951, L1960, L1970, L1971, L1980, L1990, L2000, L2005, L2006, L2010, L2020, L2030, L2034, L2035, L2036, L2037, L2038, L2040, L2050, L2060, L2070, L2080, L2090, L2106, L2108, L2112, L2114, L2116, L2126, L2128, L2132, L2134, L2136, L2180, L2182, L2184, L2186, L2188, L2190, L2192, L2200, L2210, L2220, L2230, L2232, L2240, L2250, L2260, L2265, L2270, L2275, L2280, L2300, L2310, L2320, L2330, L2335, L2340, L2350, L2360, L2370, L2375, L2380, L2385, L2387, L2390, L2395, L2397, L2405, L2415, L2425, L2430, L2492, L2500, L2510, L2520, L2525, L2526, L2530, L2540, L2550, L2570, L2580, L2600, L2610, L2620, L2622, L2624, L2627, L2628, L2630, L2640, L2650, L2660, L2670, L2680, L2750, L2755, L2760, L2768, L2780, L2785, L2795, L2800, L2810, L2820, L2830, L2840, L2850, L2861, L2999, L3000, L3001, L3002, L3003, L3010, L3020, L3030, L3031, L3040, L3050, L3060, L3070, L3080, L3090, L3100, L3140, L3150, L3160, L3170, L3201, L3202, L3203, L3204, L3206, L3207, L3208, L3209, L3211, L3212, L3213, L3214, L3215, L3216, L3217, L3219, L3221, L3222, L3224, L3225, L3230, L3250, L3251, L3252, L3253, L3254, L3255, L3257, L3260, L3265, L3300, L3310, L3320, L3330, L3332, L3334, L3340, L3350, L3360, L3370, L3380, L3390, L3400, L3410, L3420, L3430, L3440, L3450, L3455, L3460, L3465, L3470, L3480, L3485, L3500, L3510, L3520, L3530, L3540, L3550, L3560, L3570, L3580, L3590, L3595, L3600, L3610, L3620, L3630, L3640, L3649, L3650, L3660, L3670, L3671, L3674, L3675, L3677, L3678, L3702, L3710, L3720, L3730, L3740, L3760, L3761, L3762, L3763, L3764, L3765, L3766, L3806, L3807, L3808, L3809, L3891, L3900, L3901, L3904, L3905, L3906, L3908, L3912, L3913, L3915, L3916, L3917, L3918, L3919, L3921, L3923, L3924, L3925, L3927, L3929, L3930, L3931, L3933, L3935, L3956, L3960, L3961, L3962, L3967, L3971, L3973, L3975, L3976, L3977, L3978, L3980, L3981, L3982, L3984, L3995, L3999, L4000, L4002, L4010, L4020, L4030, L4040, L4045, L4050, L4055, L4060, L4070, L4080, L4090, L4100, L4110, L4130, L4205, L4210, L4350, L4360, L4361, L4370, L4386, L4387, L4392, L4394, L4396, L4397, L4398, L4631, L5000, L5010, L5020, L5050, L5060, L5100, L5105, L5150, L5160, L5200, L5210, L5220, L5230, L5250, L5270, L5280, L5301, L5312, L5321, L5331, L5341, L5400, L5410, L5420, L5430, L5450, L5460, L5500, L5505, L5510, L5520, L5530, L5535, L5540, L5560, L5570, L5580, L5585, L5590, L5595, L5600, L5610, L5611, L5613, L5614, L5616, L5617, L5618, L5620, L5622, L5624, L5626, L5628, L5629, L5630, L5631, L5632, L5634, L5636, L5637, L5638, L5639, L5640, L5642, L5643, L5644, L5645, L5646, L5647, L5648, L5649, L5650, L5651, L5652, L5653, L5654, L5655, L5656, L5658, L5661, L5665, L5666, L5668, L5670, L5671, L5672, L5673, L5676, L5677, L5678, L5679, L5680, L5681, L5682, L5683, L5684, L5685, L5686, L5688, L5690, L5692, L5694, L5695, L5696, L5697, L5698, L5699, L5700, L5701, L5702, L5703, L5704, L5705, L5706, L5707, L5710, L5711, L5712, L5714, L5716, L5718, L5722, L5724, L5726, L5728, L5780, L5781, L5782, L5785, L5790, L5795, L5810, L5811, L5812, L5814, L5816, L5818, L5822, L5824, L5826, L5828, L5830, L5840, L5845, L5848, L5850, L5855, L5856, L5857, L5858, L5859, L5910, L5920, L5925, L5930, L5940, L5950, L5960, L5961, L5962, L5964, L5966, L5968, L5969, L5970, L5971, L5972, L5973, L5974, L5975, L5976, L5978, L5979, L5980, L5981, L5982, L5984, L5985, L5986, L5987, L5988, L5990, L5999, L6000, L6010, L6020, L6026, L6050, L6055, L6100, L6110, L6120, L6130, L6200, L6205, L6250, L6300, L6310, L6320, L6350, L6360, L6370, L6380, L6382, L6384, L6386, L6388, L6400, L6450, L6500, L6550, L6570, L6580, L6582, L6584, L6586, L6588, L6590, L6600, L6605, L6610, L6611, L6615, L6616, L6620, L6621, L6623, L6624, L6625, L6628, L6629, L6630, L6632, L6635, L6637, L6638, L6640, L6641, L6642, L6645, L6646, L6647, L6648, L6650, L6655, L6660, L6665, L6670, L6672, L6675, L6676, L6677, L6680, L6682, L6684, L6686, L6687, L6688, L6689, L6690, L6691, L6692, L6693, L6694, L6695, L6696, L6697, L6698, L6703, L6704, L6706, L6707, L6708, L6709, L6711, L6712, L6713, L6714, L6715, L6721, L6722, L6805, L6810, L6880, L6881, L6882, L6883, L6884, L6885, L6890, L6895, L6900, L6905, L6910, L6915, L6920, L6925, L6930, L6935, L6940, L6945, L6950, L6955, L6960, L6965, L6970, L6975, L7007, L7008, L7009, L7040, L7045, L7170, L7180, L7181, L7185, L7186, L7190, L7191, L7259, L7360, L7362, L7364, L7366, L7367, L7368, L7400, L7401, L7402, L7403, L7404, L7405, L7499, L7510, L7520, L7600, L7700, L7900, L7902, L8000, L8001, L8002, L8010, L8015, L8020, L8030, L8031, L8032, L8033, L8035, L8039, L8040, L8041, L8042, L8043, L8044, L8045, L8046, L8047, L8048, L8049, L8300, L8310, L8320, L8330, L8400, L8410, L8415, L8417, L8420, L8430, L8435, L8440, L8460, L8465, L8470, L8480, L8485, L8499, L8500, L8501, L8505, L8507, L8509, L8510, L8511, L8512, L8513, L8514, L8515, L8600, L8603, L8604, L8605, L8606, L8607, L8608, L8609, L8610, L8612, L8613, L8614, L8615, L8616, L8617, L8618, L8619, L8621, L8622, L8623, L8624, L8625, L8627, L8628, L8629, L8630, L8631, L8641, L8642, L8658, L8659, L8670, L8679, L8680, L8681, L8682, L8683, L8684, L8685, L8686, L8687, L8688, L8689, L8692, L8693, L8694, L8695, L8696, L8698, L8699, L8701, L8702, L9900, P9044, Q0477, Q0478, Q0479, Q0480, Q0481, Q0482, Q0483, Q0484, Q0485, Q0486, Q0487, Q0488, Q0489, Q0490, Q0491, Q0492, Q0493, Q0494, Q0495, Q0496, Q0497, Q0498, Q0499, Q0500, Q0501, Q0502, Q0503, Q0504, Q0506, Q0507, Q0508, Q0509, Q2004, Q3001, Q4001, Q4002, Q4003, Q4004, Q4005, Q4006, Q4007, Q4008, Q4009, Q4010, Q4011, Q4012, Q4013, Q4014, Q4015, Q4016, Q4017, Q4018, Q4019, Q4020, Q4021, Q4022, Q4023, Q4024, Q4025, Q4026, Q4027, Q4028, Q4029, Q4030, Q4031, Q4032, Q4033, Q4034, Q4035, Q4036, Q4037, Q4038, Q4039, Q4040, Q4041, Q4042, Q4043, Q4044, Q4045, Q4046, Q4047, Q4048, Q4049, Q4050, Q4051, Q4100, Q4101, Q4102, Q4103, Q4104, Q4105, Q4106, Q4107, Q4108, Q4110, Q4111, Q4112, Q4113, Q4114, Q4115, Q4116, Q4117, Q4118, Q4121, Q4122, Q4123, Q4124, Q4125, Q4126, Q4127, Q4128, Q4130, Q4132, Q4133, Q4134, Q4135, Q4136, Q4137, Q4138, Q4139, Q4140, Q4141, Q4142, Q4143, Q4145, Q4146, Q4147, Q4148, Q4149, Q4150, Q4151, Q4152, Q4153, Q4154, Q4155, Q4156, Q4157, Q4158, Q4159, Q4160, Q4161, Q4162, Q4163, Q4164, Q4165, Q4166, Q4167, Q4168, Q4169, Q4170, Q4171, Q4173, Q4174, Q4175, Q4176, Q4177, Q4178, Q4179, Q4180, Q4181, Q4182, Q4183, Q4184, Q4185, Q4186, Q4187, Q4188, Q4189, Q4190, Q4191, Q4192, Q4193, Q4194, Q4195, Q4196, Q4197, Q4198, Q4199, Q4200, Q4201, Q4202, Q4203, Q4204, Q4205, Q4206, Q4208, Q4209, Q4210, Q4211, Q4212, Q4213, Q4214, Q4215, Q4216, Q4217, Q4218, Q4219, Q4220, Q4221, Q4222, Q4224, Q4225, Q4226, Q4227, Q4229, Q4230, Q4231, Q4232, Q4233, Q4234, Q4235, Q4237, Q4238, Q4239, Q4240, Q4241, Q4242, Q4244, Q4245, Q4246, Q4247, Q4248, Q4249, Q4250, Q4251, Q4252, Q4253, Q4254, Q4255, Q4256, Q4257, Q4258, Q9951, Q9954, Q9958, Q9959, Q9960, Q9961, Q9962, Q9963, Q9964, Q9965, Q9966, Q9967, Q9968, Q9969, Q9982, Q9983, S0155, S1001, S1002, S1015, S1016, S1030, S1031, S1034, S1035, S1036, S1037, S1040, S1091, S3600, S4989, S5010, S5013, S5160, S5161, S5162, S5165, S5560, S5561, S5565, S5566, S5570, S5571, S8096, S8097, S8100, S8101, S8110, S8120, S8121, S8130, S8131, S8185, S8186, S8189, S8210, S8265, S8270, S8301, S8415, S8420, S8421, S8422, S8423, S8424, S8425, S8426, S8427, S8428, S8429, S8430, S8431, S8450, S8451, S8452, S8460, S8490, S8999, S9001, S9007, S9055, S9434, S9435, T1505, T1999, T2028, T2029, T2039, T2101, T4521, T4522, T4523, T4524, T4525, T4526, T4527, T4528, T4529, T4530, T4531, T4532, T4533, T4534, T4535, T4536, T4537, T4538, T4539, T4540, T4541, T4542, T4543, T4544, T4545, T5001, T5999, V5336 |
| 244 | Gastric bypass and volume reduction | - | 0312T, 0313T, 0314T, 0315T, 0316T, 0317T, 43644, 43645, 43770, 43771, 43772, 43773, 43774, 43775, 43842, 43843, 43845, 43846, 43847, 43848, 43886, 43887, 43888 |
| 245 | Telehealth (includes telephone calls, online communication, remote monitoring and surveillance) | - | 0378T, 0379T, 0488T, 0578T, 0579T, 0604T, 0605T, 0606T, 0607T, 0608T, 0650T, 0702T, 0703T, 0704T, 0705T, 0706T, 93228, 93229, 93264, 93268, 93270, 93271, 93272, 93293, 93294, 93295, 93296, 93297, 93298, 98966, 98967, 98968, 98970, 98971, 98972, 98975, 98976, 98977, 98980, 98981, 99421, 99422, 99423, 99441, 99442, 99443, 99446, 99447, 99448, 99449, 99451, 99452, 99453, 99454, 99457, 99458, 99473, 99474, 99495, 99496, G0071, G0406, G0407, G0408, G0425, G0426, G0427, G0459, G0508, G0509, G2010, G2025, G2066, G2250, Q3014, S0320, S9110, T1014 |
| 997 | Current Dental Terminology (CDT) codes (codes starting with D) | - | D0120, D0140, D0145, D0150, D0160, D0170, D0171, D0180, D0190, D0191, D0210, D0220, D0230, D0240, D0250, D0251, D0270, D0272, D0273, D0274, D0277, D0310, D0320, D0321, D0322, D0330, D0340, D0350, D0351, D0364, D0365, D0366, D0367, D0368, D0369, D0370, D0371, D0380, D0381, D0382, D0383, D0384, D0385, D0386, D0391, D0393, D0394, D0395, D0411, D0412, D0414, D0415, D0416, D0417, D0418, D0419, D0422, D0423, D0425, D0431, D0460, D0470, D0472, D0473, D0474, D0475, D0476, D0477, D0478, D0479, D0480, D0481, D0482, D0483, D0484, D0485, D0486, D0502, D0600, D0601, D0602, D0603, D0604, D0605, D0606, D0701, D0702, D0703, D0704, D0705, D0706, D0707, D0708, D0709, D0999, D1110, D1120, D1206, D1208, D1310, D1320, D1321, D1330, D1351, D1352, D1353, D1354, D1355, D1510, D1516, D1517, D1520, D1526, D1527, D1551, D1552, D1553, D1556, D1557, D1558, D1575, D1701, D1702, D1703, D1704, D1705, D1706, D1707, D1999, D2140, D2150, D2160, D2161, D2330, D2331, D2332, D2335, D2390, D2391, D2392, D2393, D2394, D2410, D2420, D2430, D2510, D2520, D2530, D2542, D2543, D2544, D2610, D2620, D2630, D2642, D2643, D2644, D2650, D2651, D2652, D2662, D2663, D2664, D2710, D2712, D2720, D2721, D2722, D2740, D2750, D2751, D2752, D2753, D2780, D2781, D2782, D2783, D2790, D2791, D2792, D2794, D2799, D2910, D2915, D2920, D2921, D2928, D2929, D2930, D2931, D2932, D2933, D2934, D2940, D2941, D2949, D2950, D2951, D2952, D2953, D2954, D2955, D2957, D2960, D2961, D2962, D2971, D2975, D2980, D2981, D2982, D2983, D2990, D2999, D3110, D3120, D3220, D3221, D3222, D3230, D3240, D3310, D3320, D3330, D3331, D3332, D3333, D3346, D3347, D3348, D3351, D3352, D3353, D3355, D3356, D3357, D3410, D3421, D3425, D3426, D3428, D3429, D3430, D3431, D3432, D3450, D3460, D3470, D3471, D3472, D3473, D3501, D3502, D3503, D3910, D3911, D3920, D3921, D3950, D3999, D4210, D4211, D4212, D4230, D4231, D4240, D4241, D4245, D4249, D4260, D4261, D4263, D4264, D4265, D4266, D4267, D4268, D4270, D4273, D4274, D4275, D4276, D4277, D4278, D4283, D4285, D4322, D4323, D4341, D4342, D4346, D4355, D4381, D4910, D4920, D4921, D4999, D5110, D5120, D5130, D5140, D5211, D5212, D5213, D5214, D5221, D5222, D5223, D5224, D5225, D5226, D5227, D5228, D5282, D5283, D5284, D5286, D5410, D5411, D5421, D5422, D5511, D5512, D5520, D5611, D5612, D5621, D5622, D5630, D5640, D5650, D5660, D5670, D5671, D5710, D5711, D5720, D5721, D5725, D5730, D5731, D5740, D5741, D5750, D5751, D5760, D5761, D5765, D5810, D5811, D5820, D5821, D5850, D5851, D5862, D5863, D5864, D5865, D5866, D5867, D5875, D5876, D5899, D5911, D5912, D5913, D5914, D5915, D5916, D5919, D5922, D5923, D5924, D5925, D5926, D5927, D5928, D5929, D5931, D5932, D5933, D5934, D5935, D5936, D5937, D5951, D5952, D5953, D5954, D5955, D5958, D5959, D5960, D5982, D5983, D5984, D5985, D5986, D5987, D5988, D5991, D5992, D5993, D5995, D5996, D5999, D6010, D6011, D6012, D6013, D6040, D6050, D6051, D6055, D6056, D6057, D6058, D6059, D6060, D6061, D6062, D6063, D6064, D6065, D6066, D6067, D6068, D6069, D6070, D6071, D6072, D6073, D6074, D6075, D6076, D6077, D6080, D6081, D6082, D6083, D6084, D6085, D6086, D6087, D6088, D6090, D6091, D6092, D6093, D6094, D6095, D6096, D6097, D6098, D6099, D6100, D6101, D6102, D6103, D6104, D6110, D6111, D6112, D6113, D6114, D6115, D6116, D6117, D6118, D6119, D6120, D6121, D6122, D6123, D6190, D6191, D6192, D6194, D6195, D6198, D6199, D6205, D6210, D6211, D6212, D6214, D6240, D6241, D6242, D6243, D6245, D6250, D6251, D6252, D6253, D6545, D6548, D6549, D6600, D6601, D6602, D6603, D6604, D6605, D6606, D6607, D6608, D6609, D6610, D6611, D6612, D6613, D6614, D6615, D6624, D6634, D6710, D6720, D6721, D6722, D6740, D6750, D6751, D6752, D6753, D6780, D6781, D6782, D6783, D6784, D6790, D6791, D6792, D6793, D6794, D6920, D6930, D6940, D6950, D6980, D6985, D6999, D7111, D7140, D7210, D7220, D7230, D7240, D7241, D7250, D7251, D7260, D7261, D7270, D7272, D7280, D7282, D7283, D7285, D7286, D7287, D7288, D7290, D7291, D7292, D7293, D7294, D7295, D7296, D7297, D7298, D7299, D7300, D7310, D7311, D7320, D7321, D7340, D7350, D7410, D7411, D7412, D7413, D7414, D7415, D7440, D7441, D7450, D7451, D7460, D7461, D7465, D7471, D7472, D7473, D7485, D7490, D7510, D7511, D7520, D7521, D7530, D7540, D7550, D7560, D7610, D7620, D7630, D7640, D7650, D7660, D7670, D7671, D7680, D7710, D7720, D7730, D7740, D7750, D7760, D7770, D7771, D7780, D7810, D7820, D7830, D7840, D7850, D7852, D7854, D7856, D7858, D7860, D7865, D7870, D7871, D7872, D7873, D7874, D7875, D7876, D7877, D7880, D7881, D7899, D7910, D7911, D7912, D7920, D7921, D7922, D7940, D7941, D7943, D7944, D7945, D7946, D7947, D7948, D7949, D7950, D7951, D7952, D7953, D7955, D7961, D7962, D7963, D7970, D7971, D7972, D7979, D7980, D7981, D7982, D7983, D7990, D7991, D7993, D7994, D7995, D7996, D7997, D7998, D7999, D8010, D8020, D8030, D8040, D8070, D8080, D8090, D8210, D8220, D8660, D8670, D8680, D8681, D8695, D8696, D8697, D8698, D8699, D8701, D8702, D8703, D8704, D8999, D9110, D9120, D9130, D9210, D9211, D9212, D9215, D9219, D9222, D9223, D9230, D9239, D9243, D9248, D9310, D9311, D9410, D9420, D9430, D9440, D9450, D9610, D9612, D9613, D9630, D9910, D9911, D9912, D9920, D9930, D9932, D9933, D9934, D9935, D9941, D9942, D9943, D9944, D9945, D9946, D9947, D9948, D9949, D9950, D9951, D9952, D9961, D9970, D9971, D9972, D9973, D9974, D9975, D9985, D9986, D9987, D9990, D9991, D9992, D9993, D9994, D9995, D9996, D9997, D9999 |
| 998 | CPT codes not classified (codes ending in F) | - | 0001F, 0005F, 0012F, 0014F, 0015F, 0500F, 0501F, 0502F, 0503F, 0505F, 0507F, 0509F, 0513F, 0514F, 0516F, 0517F, 0518F, 0519F, 0520F, 0521F, 0525F, 0526F, 0528F, 0529F, 0535F, 0540F, 0545F, 0550F, 0551F, 0555F, 0556F, 0557F, 0575F, 0580F, 0581F, 0582F, 0583F, 0584F, 1000F, 1002F, 1003F, 1004F, 1005F, 1006F, 1007F, 1008F, 1010F, 1011F, 1012F, 1015F, 1018F, 1019F, 1022F, 1026F, 1030F, 1031F, 1032F, 1033F, 1034F, 1035F, 1036F, 1038F, 1039F, 1040F, 1050F, 1052F, 1055F, 1060F, 1061F, 1065F, 1066F, 1070F, 1071F, 1090F, 1091F, 1100F, 1101F, 1110F, 1111F, 1116F, 1118F, 1119F, 1121F, 1123F, 1124F, 1125F, 1126F, 1127F, 1128F, 1130F, 1134F, 1135F, 1136F, 1137F, 1150F, 1151F, 1152F, 1153F, 1157F, 1158F, 1159F, 1160F, 1170F, 1175F, 1180F, 1181F, 1182F, 1183F, 1200F, 1205F, 1220F, 1400F, 1450F, 1451F, 1460F, 1461F, 1490F, 1491F, 1493F, 1494F, 1500F, 1501F, 1502F, 1503F, 1504F, 1505F, 2000F, 2001F, 2002F, 2004F, 2010F, 2014F, 2015F, 2016F, 2018F, 2019F, 2020F, 2021F, 2022F, 2023F, 2024F, 2025F, 2026F, 2027F, 2028F, 2029F, 2030F, 2031F, 2033F, 2035F, 2040F, 2044F, 2050F, 2060F, 3006F, 3008F, 3011F, 3014F, 3015F, 3016F, 3017F, 3018F, 3019F, 3020F, 3021F, 3022F, 3023F, 3025F, 3027F, 3028F, 3035F, 3037F, 3038F, 3040F, 3042F, 3044F, 3046F, 3048F, 3049F, 3050F, 3051F, 3052F, 3055F, 3056F, 3060F, 3061F, 3062F, 3066F, 3072F, 3073F, 3074F, 3075F, 3077F, 3078F, 3079F, 3080F, 3082F, 3083F, 3084F, 3085F, 3088F, 3089F, 3090F, 3091F, 3092F, 3093F, 3095F, 3096F, 3100F, 3110F, 3111F, 3112F, 3115F, 3117F, 3118F, 3119F, 3120F, 3126F, 3130F, 3132F, 3140F, 3141F, 3142F, 3150F, 3155F, 3160F, 3170F, 3200F, 3210F, 3215F, 3216F, 3218F, 3220F, 3230F, 3250F, 3260F, 3265F, 3266F, 3267F, 3268F, 3269F, 3270F, 3271F, 3272F, 3273F, 3274F, 3278F, 3279F, 3280F, 3281F, 3284F, 3285F, 3288F, 3290F, 3291F, 3292F, 3293F, 3294F, 3300F, 3301F, 3315F, 3316F, 3317F, 3318F, 3319F, 3320F, 3321F, 3322F, 3323F, 3324F, 3325F, 3328F, 3330F, 3331F, 3340F, 3341F, 3342F, 3343F, 3344F, 3345F, 3350F, 3351F, 3352F, 3353F, 3354F, 3370F, 3372F, 3374F, 3376F, 3378F, 3380F, 3382F, 3384F, 3386F, 3388F, 3390F, 3394F, 3395F, 3450F, 3451F, 3452F, 3455F, 3470F, 3471F, 3472F, 3475F, 3476F, 3490F, 3491F, 3492F, 3493F, 3494F, 3495F, 3496F, 3497F, 3498F, 3500F, 3502F, 3503F, 3510F, 3511F, 3512F, 3513F, 3514F, 3515F, 3517F, 3520F, 3550F, 3551F, 3552F, 3555F, 3570F, 3572F, 3573F, 3650F, 3700F, 3720F, 3725F, 3750F, 3751F, 3752F, 3753F, 3754F, 3755F, 3756F, 3757F, 3758F, 3759F, 3760F, 3761F, 3762F, 3763F, 3775F, 3776F, 4000F, 4001F, 4003F, 4004F, 4005F, 4008F, 4010F, 4011F, 4012F, 4013F, 4014F, 4015F, 4016F, 4017F, 4018F, 4019F, 4025F, 4030F, 4033F, 4035F, 4037F, 4040F, 4041F, 4042F, 4043F, 4044F, 4045F, 4046F, 4047F, 4048F, 4049F, 4050F, 4051F, 4052F, 4053F, 4054F, 4055F, 4056F, 4058F, 4060F, 4062F, 4063F, 4064F, 4065F, 4066F, 4067F, 4069F, 4070F, 4073F, 4075F, 4077F, 4079F, 4084F, 4086F, 4090F, 4095F, 4100F, 4110F, 4115F, 4120F, 4124F, 4130F, 4131F, 4132F, 4133F, 4134F, 4135F, 4136F, 4140F, 4142F, 4144F, 4145F, 4148F, 4149F, 4150F, 4151F, 4153F, 4155F, 4157F, 4158F, 4159F, 4163F, 4164F, 4165F, 4167F, 4168F, 4169F, 4171F, 4172F, 4174F, 4175F, 4176F, 4177F, 4178F, 4179F, 4180F, 4181F, 4182F, 4185F, 4186F, 4187F, 4188F, 4189F, 4190F, 4191F, 4192F, 4193F, 4194F, 4195F, 4196F, 4200F, 4201F, 4210F, 4220F, 4221F, 4230F, 4240F, 4242F, 4245F, 4248F, 4250F, 4255F, 4256F, 4260F, 4261F, 4265F, 4266F, 4267F, 4268F, 4269F, 4270F, 4271F, 4274F, 4276F, 4279F, 4280F, 4290F, 4293F, 4300F, 4301F, 4305F, 4306F, 4320F, 4322F, 4324F, 4325F, 4326F, 4328F, 4330F, 4340F, 4350F, 4400F, 4450F, 4470F, 4480F, 4481F, 4500F, 4510F, 4525F, 4526F, 4540F, 4541F, 4550F, 4551F, 4552F, 4553F, 4554F, 4555F, 4556F, 4557F, 4558F, 4559F, 4560F, 4561F, 4562F, 4563F, 5005F, 5010F, 5015F, 5020F, 5050F, 5060F, 5062F, 5100F, 5200F, 5250F, 6005F, 6010F, 6015F, 6020F, 6030F, 6040F, 6045F, 6070F, 6080F, 6090F, 6100F, 6101F, 6102F, 6110F, 6150F, 7010F, 7020F, 7025F, 9001F, 9002F, 9003F, 9004F, 9005F, 9006F, 9007F |
| 999 | HCPCS Level II codes not classified (codes starting with M1; some codes starting with G) | - | C1890, G0028, G0029, G0030, G0031, G0032, G0033, G0034, G0035, G0036, G0037, G0038, G0039, G0040, G0041, G0042, G0043, G0044, G0045, G0046, G0047, G0048, G0049, G0050, G0051, G0052, G0053, G0054, G0055, G0056, G0057, G0058, G0059, G0060, G0061, G0062, G0063, G0064, G0065, G0066, G0067, G0913, G0914, G0915, G0916, G0917, G0918, G1001, G1002, G1003, G1004, G1007, G1008, G1009, G1010, G1011, G1012, G1013, G1014, G1015, G1016, G1017, G1018, G1019, G1020, G1021, G1022, G1023, G1024, G1025, G1026, G1027, G1028, G2001, G2002, G2003, G2004, G2005, G2006, G2007, G2008, G2009, G2013, G2014, G2015, G2020, G2022, G2081, G2090, G2091, G2092, G2093, G2094, G2095, G2096, G2097, G2098, G2099, G2100, G2101, G2105, G2106, G2107, G2108, G2109, G2110, G2112, G2113, G2115, G2116, G2118, G2121, G2122, G2125, G2126, G2127, G2128, G2129, G2136, G2137, G2138, G2139, G2140, G2141, G2142, G2143, G2144, G2145, G2146, G2147, G2148, G2149, G2150, G2151, G2152, G2167, G2172, G2173, G2174, G2175, G2176, G2177, G2178, G2179, G2180, G2181, G2182, G2183, G2184, G2185, G2186, G2187, G2188, G2189, G2190, G2191, G2192, G2193, G2194, G2195, G2196, G2197, G2198, G2199, G2200, G2201, G2202, G2203, G2204, G2205, G2206, G2207, G2208, G2209, G2210, G2211, G2213, G2215, G2216, G2251, G2252, G4000, G4001, G4002, G4003, G4004, G4005, G4006, G4007, G4008, G4009, G4010, G4011, G4012, G4013, G4014, G4015, G4016, G4017, G4018, G4019, G4020, G4021, G4022, G4023, G4024, G4025, G4026, G4027, G4028, G4029, G4030, G4031, G4032, G4033, G4034, G4035, G4036, G4037, G4038, G8395, G8396, G8397, G8399, G8400, G8404, G8405, G8410, G8415, G8416, G8417, G8418, G8419, G8420, G8421, G8427, G8428, G8430, G8431, G8432, G8433, G8450, G8451, G8452, G8465, G8473, G8474, G8475, G8476, G8477, G8478, G8482, G8483, G8484, G8506, G8510, G8511, G8535, G8536, G8539, G8540, G8541, G8542, G8543, G8559, G8560, G8561, G8562, G8563, G8564, G8565, G8566, G8567, G8568, G8569, G8570, G8575, G8576, G8577, G8578, G8598, G8599, G8600, G8601, G8602, G8633, G8635, G8647, G8648, G8650, G8651, G8652, G8654, G8655, G8656, G8658, G8659, G8660, G8661, G8662, G8663, G8664, G8666, G8667, G8668, G8669, G8670, G8694, G8708, G8709, G8710, G8711, G8712, G8721, G8722, G8723, G8724, G8733, G8734, G8735, G8749, G8752, G8753, G8754, G8755, G8756, G8783, G8785, G8797, G8798, G8806, G8807, G8808, G8815, G8816, G8817, G8818, G8825, G8826, G8833, G8834, G8838, G8839, G8840, G8841, G8842, G8843, G8844, G8845, G8846, G8849, G8850, G8851, G8852, G8854, G8855, G8856, G8857, G8858, G8863, G8864, G8865, G8866, G8867, G8869, G8875, G8876, G8877, G8878, G8880, G8881, G8882, G8883, G8884, G8885, G8907, G8908, G8909, G8910, G8911, G8912, G8913, G8914, G8915, G8916, G8917, G8918, G8923, G8924, G8934, G8935, G8936, G8937, G8941, G8942, G8944, G8946, G8950, G8952, G8955, G8956, G8958, G8961, G8962, G8963, G8964, G8965, G8966, G8967, G8968, G8969, G9001, G9002, G9003, G9004, G9005, G9006, G9007, G9008, G9009, G9010, G9011, G9012, G9013, G9014, G9016, G9050, G9051, G9052, G9053, G9054, G9055, G9056, G9057, G9058, G9059, G9060, G9061, G9062, G9063, G9064, G9065, G9066, G9067, G9068, G9069, G9070, G9071, G9072, G9073, G9074, G9075, G9077, G9078, G9079, G9080, G9083, G9084, G9085, G9086, G9087, G9088, G9089, G9090, G9091, G9092, G9093, G9094, G9095, G9096, G9097, G9098, G9099, G9100, G9101, G9102, G9103, G9104, G9105, G9106, G9107, G9108, G9109, G9110, G9111, G9112, G9113, G9114, G9115, G9116, G9117, G9123, G9124, G9125, G9126, G9128, G9129, G9130, G9131, G9132, G9133, G9134, G9135, G9136, G9137, G9138, G9139, G9140, G9148, G9149, G9150, G9151, G9152, G9153, G9187, G9188, G9189, G9190, G9191, G9192, G9196, G9197, G9198, G9212, G9213, G9223, G9225, G9226, G9227, G9228, G9229, G9230, G9231, G9242, G9243, G9246, G9247, G9250, G9251, G9254, G9255, G9273, G9274, G9275, G9276, G9277, G9278, G9279, G9280, G9281, G9282, G9283, G9284, G9285, G9286, G9287, G9288, G9289, G9290, G9291, G9292, G9293, G9294, G9295, G9296, G9297, G9298, G9299, G9305, G9306, G9307, G9308, G9309, G9310, G9311, G9312, G9313, G9314, G9315, G9316, G9317, G9318, G9319, G9321, G9322, G9341, G9342, G9344, G9345, G9347, G9351, G9352, G9353, G9354, G9355, G9356, G9357, G9358, G9359, G9360, G9361, G9364, G9367, G9368, G9380, G9382, G9383, G9384, G9385, G9386, G9393, G9394, G9395, G9396, G9402, G9403, G9404, G9405, G9406, G9407, G9408, G9409, G9410, G9411, G9412, G9413, G9414, G9415, G9416, G9417, G9418, G9419, G9420, G9421, G9422, G9423, G9424, G9425, G9426, G9427, G9428, G9429, G9430, G9431, G9432, G9434, G9451, G9452, G9453, G9454, G9455, G9456, G9457, G9458, G9459, G9460, G9468, G9470, G9471, G9480, G9490, G9497, G9498, G9500, G9501, G9502, G9504, G9505, G9506, G9507, G9508, G9509, G9510, G9511, G9512, G9513, G9514, G9515, G9516, G9517, G9518, G9519, G9520, G9521, G9522, G9529, G9530, G9531, G9533, G9537, G9539, G9540, G9541, G9542, G9543, G9544, G9547, G9548, G9549, G9550, G9551, G9552, G9553, G9554, G9555, G9556, G9557, G9580, G9582, G9593, G9594, G9595, G9596, G9597, G9598, G9599, G9603, G9604, G9605, G9606, G9607, G9608, G9609, G9610, G9611, G9612, G9613, G9614, G9618, G9620, G9621, G9622, G9623, G9624, G9625, G9626, G9627, G9628, G9629, G9630, G9631, G9632, G9633, G9637, G9638, G9642, G9643, G9644, G9645, G9646, G9648, G9649, G9651, G9654, G9655, G9656, G9658, G9659, G9660, G9661, G9662, G9663, G9664, G9665, G9674, G9675, G9676, G9677, G9678, G9679, G9680, G9681, G9682, G9683, G9684, G9685, G9687, G9688, G9689, G9690, G9691, G9692, G9693, G9694, G9695, G9696, G9697, G9698, G9699, G9700, G9702, G9703, G9704, G9705, G9706, G9707, G9708, G9709, G9710, G9711, G9712, G9713, G9714, G9715, G9716, G9717, G9718, G9719, G9720, G9721, G9722, G9723, G9724, G9725, G9726, G9727, G9728, G9729, G9730, G9731, G9732, G9733, G9734, G9735, G9736, G9737, G9740, G9741, G9744, G9745, G9746, G9751, G9752, G9753, G9754, G9755, G9756, G9757, G9758, G9760, G9761, G9762, G9763, G9764, G9765, G9766, G9767, G9768, G9769, G9770, G9771, G9772, G9773, G9774, G9775, G9776, G9777, G9778, G9779, G9780, G9781, G9782, G9784, G9785, G9786, G9787, G9788, G9789, G9790, G9791, G9792, G9793, G9794, G9795, G9796, G9797, G9805, G9806, G9807, G9808, G9809, G9810, G9811, G9812, G9813, G9818, G9819, G9820, G9821, G9822, G9823, G9824, G9830, G9831, G9832, G9838, G9839, G9840, G9841, G9842, G9843, G9844, G9845, G9846, G9847, G9848, G9852, G9853, G9854, G9858, G9859, G9860, G9861, G9862, G9868, G9869, G9870, G9873, G9874, G9875, G9876, G9877, G9878, G9879, G9880, G9881, G9882, G9883, G9884, G9885, G9890, G9891, G9892, G9893, G9894, G9895, G9896, G9897, G9898, G9899, G9900, G9901, G9902, G9903, G9904, G9905, G9906, G9907, G9908, G9909, G9910, G9911, G9912, G9913, G9914, G9915, G9916, G9917, G9918, G9919, G9920, G9921, G9922, G9923, G9925, G9926, G9927, G9928, G9929, G9930, G9931, G9932, G9938, G9939, G9940, G9942, G9943, G9945, G9946, G9948, G9949, G9954, G9955, G9956, G9957, G9958, G9959, G9960, G9961, G9962, G9963, G9964, G9965, G9968, G9969, G9970, G9974, G9975, G9976, G9977, G9987, G9988, G9989, G9990, G9991, G9992, G9993, G9994, G9995, G9996, G9997, G9998, G9999, M1003, M1004, M1005, M1006, M1007, M1008, M1009, M1010, M1011, M1012, M1013, M1014, M1016, M1017, M1018, M1019, M1020, M1021, M1027, M1028, M1029, M1032, M1034, M1035, M1036, M1037, M1038, M1039, M1040, M1041, M1043, M1045, M1046, M1049, M1051, M1052, M1054, M1055, M1056, M1057, M1058, M1059, M1060, M1067, M1068, M1069, M1070, M1071, M1072, M1073, M1074, M1075, M1076, M1077, M1078, M1079, M1080, M1081, M1082, M1083, M1084, M1085, M1086, M1087, M1088, M1089, M1094, M1095, M1096, M1097, M1098, M1099, M1100, M1101, M1102, M1103, M1104, M1105, M1106, M1107, M1108, M1109, M1110, M1111, M1112, M1113, M1114, M1115, M1116, M1117, M1118, M1119, M1120, M1121, M1122, M1123, M1124, M1125, M1126, M1127, M1128, M1129, M1130, M1131, M1132, M1133, M1134, M1135, M1141, M1142, M1143, M1145, M1146, M1147, M1148, M1149, S0285, T2050, T2051 |

**Supplementary Table 2.** Single-label CCS categories for procedures.

## **Medication Categories**

| **Central Nervous System Medication Category** | **VA Class** |
| --- | --- |
| Opioid analgesics | CN101, CN102 |
| Non-opioid analgesics | CN103, CN104 |
| Other analgesics | CN100, IN500 |
| Antimigraine agents | CN105 |
| Anesthetics | CN200, CN201, CN202, CN203, CN204, CN205 |
| Sedative/hypnotics | CN300, CN301, CN302, CN309 |
| Other sedatives/hypnotics | IN520, IN570 |
| Anticonvulsants | CN400 |
| Other anticonvulsants | IN530 |
| Antiparkinson agents | CN500 |
| Antivertigo agents | CN550 |
| Antidepressants | |
| Tricyclic antidepressants | CN601 |
| Monoamine oxidase inhibitor antidepressants | CN602 |
| Other antidepressants | CN600, CN609, IN550 |
| Antipsychotics | CN700, CN701, CN709 |
| Lithium salts | CN750 |
| Other antipsychotics | IN580 |
| Stimulants | CN800, CN801, CN802, CN809, CN850 |
| Other central nervous system medications | CN900, IN590 |
| Other antidotes/deterrents | IN595 |
| Central nervous system investigational drugs | IN500, IN520, IN530, IN550, IN570, IN580, IN590, IN595 |

**Supplementary Table 3.** Central nervous system medication categories, adopted from Kessler et al.^1^

# **Supplementary Note 2: Combining Structured and NLP-extracted SBDH**

We combined structured and NLP-extracted SBDH to have 13 distinct SBDH categories. 5 SBDH were represented in both sets – ‘social problems’ (social or familial problems [structured] + social isolation [NLP-extracted]), ‘financial problems’ (employment or financial problems [structured] + job or financial insecurity [NLP-extracted]), ‘housing instability’, ‘legal problems’, and ‘violence’. NLP-extracted-only SBDH includes 7 categories – ‘barriers to care’, ‘transition of care’, ‘food insecurity’, ‘substance abuse’, ‘psychiatric symptoms’, ‘pain’, and ‘patient disability’ whereas ‘non-specific psychosocial needs’ was available only in the structured SBDH. Descriptions of the SBDH categories along with used ICD-9 and VA stop codes (for structured SBDH) are available at Mitra et al. ^3^

# **Supplementary Note 3: Data Statistics**

For the 7-day prediction cohort we have 17,210,996 discharges of which 2,877,495 discharges are from FY 2015 (Test split). Similarly, we have 17,023,781; 16,539,445 and 15,807,523 discharges for the 30, 90 and 180-day prediction cohorts respectively of which 2,690,280; 2,205,944 and 1,474,022 discharges are from FY 2015. We provide summary statistics of the four cohorts in Supplementary Tables 4a-d.

|  | **Participants, No. (%)** | | | |
| --- | --- | --- | --- | --- |
|  | **Training Split** | | **Test Split** | |
|  | **Case**  **(N=698)** | **Control (N=3,524)** | **Case**  **(N=151)** | **Control**  **(N=721)** |
| **Race** | | | | |
| White | 585 (83.81) | 2,452 (69.58) | 127 (84.11) | 525 (72.82) |
| Black | 37 (5.30) | 820 (23.27) | 7 (4.64) | 160 (22.19) |
| Asian | 2 (0.29) | 13 (0.37) | 2 (1.32) | 1 (0.14) |
| Native Hawaiian or Other Pacific Islander | 6 (0.86) | 27 (0.77) | 0 (0) | 1 (0.14) |
| American Indian | 5 (0.72) | 32 (0.91) | 2 (1.32) | 2 (0.28) |
| Unknown | 63 (9.03) | 180 (5.11) | 13 (8.61) | 32 (4.44) |
| **Sex** | | | | |
| Male | 669 (95.85) | 3,251 (92.25) | 139 (92.05) | 669 (92.79) |
| Female | 29 (4.15) | 273 (7.75) | 12 (7.95) | 52 (7.21) |
| **Age** | | | | |
| 18-29 | 28 (4.01) | 169 (4.80) | 6 (3.97) | 20 (2.77) |
| 30-39 | 59 (8.45) | 218 (6.19) | 14 (9.27) | 57 (7.91) |
| 40-49 | 77 (11.03) | 372 (10.56) | 12 (7.95) | 60 (8.32) |
| 50-59 | 132 (18.91) | 946 (26.84) | 25 (16.56) | 158 (21.91) |
| 60-69 | 206 (29.51) | 1120 (31.78) | 48 (31.79) | 258 (35.78) |
| 70-79 | 89 (12.75) | 356 (10.10) | 21 (13.91) | 84 (11.65) |
| >=80 | 107 (15.33) | 343 (9.73) | 25 (16.56) | 84 (11.65) |
| **Marital Status** | | | | |
| Married | 240 (34.38) | 1,262 (35.81) | 53 (35.10) | 252 (34.95) |
| Single | 129 (18.48) | 601 (17.05) | 22 (14.57) | 131 (18.17) |
| Divorced | 278 (39.83) | 1,344 (38.14) | 65 (43.05) | 287 (39.81) |
| Widowed | 44 (6.30) | 289 (8.20) | 10 (6.62) | 49 (6.80) |
| Unknown | 7 (1.00) | 28 (0.79) | 1 (0.66) | 2 (0.28) |
| **Previous suicide attempt** | 125 (17.91) | 308 (8.74) | 50 (33.11) | 62 (8.6) |
| **Previous suicide ideation** | 232 (33.24) | 616 (17.48) | 71 (47.02) | 143 (19.83) |
| **Discharge Type** | | | | |
| Outpatient ED | 394 (56.44) | 1,979 (56.15) | 84 (55.63) | 411 (57.00) |
| Inpatient | 304 (43.56) | 1,545 (43.85) | 67 (44.38) | 310 (42.99) |
| **Suicide Rate in the Entire Sample (%)** | 0.005 | | 0.005 | |

**Supplementary Table 4a.** Summary Statistics for the 7-day prediction cohort

|  | **Participants, No. (%)** | | | |
| --- | --- | --- | --- | --- |
|  | **Training Split** | | **Test Split** | |
|  | **Case**  **(N=1,980)** | **Control (N=9,976)** | **Case**  **(N=396)** | **Control**  **(N=1,904)** |
| **Race** | | | | |
| White | 1,634 (82.53) | 6,937 (69.54) | 317 (80.05) | 1,312 (68.91) |
| Black | 127 (6.41) | 2,297 (23.03) | 32 (8.08) | 471 (24.74) |
| Asian | 8 (0.40) | 43 (0.43) | 3 (0.76) | 5 (0.26) |
| Native Hawaiian or Other Pacific Islander | 16 (0.81) | 76 (0.76) | 3 (0.76) | 12 (0.63) |
| American Indian | 12 (0.61) | 95 (0.95) | 3 (0.76) | 11 (0.58) |
| Unknown | 183 (9.24) | 528 (5.29) | 38 (9.60) | 93 (4.88) |
| **Sex** | | | | |
| Male | 1,914 (96.67) | 9,229 (92.51) | 368 (92.93) | 1,756 (92.23) |
| Female | 66 (3.33) | 747 (7.49) | 28 (7.07) | 148 (7.77) |
| **Age** | | | | |
| 18-29 | 113 (5.71) | 489 (4.90) | 28 (7.07) | 73 (3.83) |
| 30-39 | 168 (8.48) | 627 (6.29) | 39 (9.85) | 186 (9.77) |
| 40-49 | 222 (11.21) | 1,067 (10.70) | 51 (12.88) | 169 (8.88) |
| 50-59 | 366 (18.48) | 2,567 (25.73) | 56 (14.14) | 441 (23.16) |
| 60-69 | 582 (29.39) | 3,154 (31.62) | 115 (29.04) | 602 (31.62) |
| 70-79 | 226 (11.41) | 1,114 (11.17) | 57 (14.39) | 234 (12.29) |
| >=80 | 303 (15.30) | 958 (9.60) | 50 (12.63) | 199 (10.45) |
| **Marital Status** | | | | |
| Married | 672 (33.94) | 3,582 (35.91) | 135 (34.09) | 666 (34.98) |
| Single | 386 (19.49) | 1,617 (16.21) | 79 (19.95) | 351 (18.43) |
| Divorced | 773 (39.04) | 3,871 (38.80) | 162 (40.91) | 729 (38.29) |
| Widowed | 129 (6.52) | 808 (8.10) | 17 (4.29) | 144 (7.56) |
| Unknown | 20 (1.01) | 98 (0.98) | 3 (0.76) | 14 (0.74) |
| **Previous suicide attempt** | 365 (18.43) | 792 (7.94) | 107 (27.72) | 185(9.72) |
| **Previous suicide ideation** | 686 (34.65) | 1762 (17.66) | 176 (44.44) | 397 (20.85) |
| **Discharge Type** | | | | |
| Outpatient ED | 1,218 (61.52) | 6,132 (61.47) | 241 (60.86) | 1,163 (61.08) |
| Inpatient | 762 (38.49) | 3,844 (38.53) | 155 (39.15) | 741 (38.92) |
| **Suicide Rate in the Entire Sample (%)** | 0.014 | | 0.015 | |

**Supplementary Table 4b.** Summary Statistics for the 30-day prediction cohort

|  | **Participants, No. (%)** | | | |
| --- | --- | --- | --- | --- |
|  | **Training Split** | | **Test Split** | |
|  | **Case**  **(N=4,289)** | **Control (N=21,350)** | **Case**  **(N=641)** | **Control**  **(N=3,300)** |
| **Race** | | | | |
| White | 3,517 (82.00) | 14,637 (68.56) | 521 (81.28) | 2,204 (66.79) |
| Black | 277 (6.46) | 5,070 (23.75) | 40 (6.24) | 862 (26.12) |
| Asian | 27 (0.63) | 92 (0.43) | 3 (0.47) | 13 (0.39) |
| Native Hawaiian or Other Pacific Islander | 31 (0.72) | 162 (0.76) | 2 (0.31) | 26 (0.79) |
| American Indian | 36 (0.84) | 199 (0.93) | 4 (0.62) | 19 (0.58) |
| Unknown | 401 (9.35) | 1,190 (5.57) | 71 (11.08) | 176 (5.33) |
| **Sex** | | | | |
| Male | 4,102 (95.64) | 19,764 (92.57) | 586 (91.42) | 3,015 (91.36) |
| Female | 187 (4.36) | 1,586 (7.43) | 55 (8.58) | 285 (8.64) |
| **Age** | | | | |
| 18-29 | 287 (6.69) | 1,053 (4.93) | 44 (6.86) | 159 (4.82) |
| 30-39 | 394 (9.19) | 1,349 (6.32) | 86 (13.42) | 314 (9.52) |
| 40-49 | 499 (11.63) | 2,329 (10.91) | 82 (12.79) | 318 (9.64) |
| 50-59 | 811 (18.91) | 5,405 (25.32) | 97 (15.13) | 772 (23.39) |
| 60-69 | 1,253 (29.21) | 6,758 (31.65) | 186 (29.02) | 1,044 (31.64) |
| 70-79 | 494 (11.52) | 2,359 (11.05) | 83 (12.95) | 389 (11.79) |
| >=80 | 551 (12.85) | 2,097 (9.82) | 63 (9.83) | 304 (9.21) |
| **Marital Status** | | | | |
| Married | 1,373 (32.01) | 7,698 (36.06) | 222 (34.63) | 1,137 (34.45) |
| Single | 843 (19.65) | 3,523 (16.50) | 129 (20.12) | 592 (17.94) |
| Divorced | 1,751 (40.83) | 8,135 (38.10) | 251 (39.16) | 1,287 (39.00) |
| Widowed | 273 (6.37) | 1,678 (7.86) | 36 (5.62) | 233 (7.06) |
| Unknown | 49 (1.14) | 316 (1.48) | 3 (0.47) | 51 (1.55) |
| **Previous suicide attempt** | 792 (18.47) | 1,673 (7.84) | 168 (26.21) | 335 (10.15) |
| **Previous suicide ideation** | 1,501 (35.00) | 3,760 (17.61) | 277 (43.21) | 696 (21.09) |
| **Discharge Type** | | | | |
| Outpatient ED | 2,762 (64.40) | 13,772 (64.51) | 421 (65.68) | 2,143 (64.94) |
| Inpatient | 1,527 (35.61) | 7,578 (35.49) | 220 (34.32) | 1,157 (35.06) |
| **Suicide Rate in the Entire Sample (%)** | 0.03 | | 0.03 | |

**Supplementary Table 4c.** Summary Statistics for the 90-day prediction cohort

|  | **Participants, No. (%)** | | | |
| --- | --- | --- | --- | --- |
|  | **Training Split** | | **Test Split** | |
|  | **Case**  **(N=7,038)** | **Control (N=35,141)** | **Case**  **(N=740)** | **Control**  **(N=3,749)** |
| **Race** | | | | |
| White | 5,756 (81.78) | 23,860 (67.90) | 619 (83.65) | 2,478 (66.10) |
| Black | 507 (7.20) | 8,453 (24.05) | 63 (8.51) | 942 (25.13) |
| Asian | 31 (0.44) | 159 (0.45) | 3 (0.41) | 13 (0.35) |
| Native Hawaiian or Other Pacific Islander | 57 (0.81) | 254 (0.72) | 1 (0.14) | 29(0.77) |
| American Indian | 71 (1.01) | 332 (0.94) | 4 (0.54) | 26 (0.69) |
| Unknown | 616 (8.75) | 2,083 (5.93) | 50 (6.76) | 261 (6.96) |
| **Sex** | | | | |
| Male | 6,662 (94.66) | 32,528 (92.56) | 680 (91.89) | 3,435 (91.62) |
| Female | 376 (5.34) | 1,613 (7.44) | 60 (8.11) | 314 (8.38) |
| **Age** | | | | |
| 18-29 | 536 (7.62) | 1,756 (5.00) | 49 (6.62) | 183 (4.88) |
| 30-39 | 685 (9.73) | 2,330 (6.63) | 104 (14.05) | 341 (9.10) |
| 40-49 | 868 (12.33) | 3,894 (11.08) | 72 (9.73) | 379 (10.11) |
| 50-59 | 1,448 (20.57) | 8,825 (25.11) | 114 (15.41) | 848 (22.62) |
| 60-69 | 1,986 (28.22) | 11,003 (31.31) | 241 (32.57) | 1,183 (31.56) |
| 70-79 | 733 (10.41) | 3,912 (11.13) | 98 (13.24) | 457 (12.19) |
| >=80 | 782 (11.11) | 3,421 (9.74) | 62 (8.38) | 358 (9.55) |
| **Marital Status** | | | | |
| Married | 2,227 (31.64) | 12,674 (36.07) | 260 (35.14) | 1,329 (35.45) |
| Single | 1,454 (20.6) | 5,753 (16.37) | 157 (21.22) | 651 (17.36) |
| Divorced | 2,866 (40.72) | 13,319 (37.90) | 287 (38.78) | 1,408 (37.560 |
| Widowed | 401 (5.70) | 2,733 (7.78) | 35 (4.73) | 248 (6.62) |
| Unknown | 90 (1.28) | 1662 (1.88) | 1 (0.14) | 113 (3.01) |
| **Previous suicide attempt** | 1,377 (19.57) | 2,855 (8.12) | 184 (24.86) | 376 (10.03) |
| **Previous suicide ideation** | 2,435 (34.60) | 6,185 (17.60) | 292 (39.46) | 811 (21.63) |
| **Discharge Type** | | | | |
| Outpatient ED | 4,577 (65.03) | 12,207 (34.73) | 500 (67.57) | 2,451 (65.37) |
| Inpatient | 2,461 (34.97) | 22,934 (65.27) | 240 (32.43) | 1,298 (34.62) |
| **Suicide Rate in the Entire Sample (%)** | 0.05 | | 0.05 | |

**Supplementary Table 4d.** Summary Statistics for the 180-day prediction cohort

# **Supplementary Note 4: Results**

| **Model** | **Predictor Configuration** | **ROC AUC (SD), %** | | | **PR AUC (SD), %** | | |
| --- | --- | --- | --- | --- | --- | --- | --- |
|  |  | **Outpatient ED** | **Inpatient** | **All Discharges** | **Outpatient ED** | **Inpatient** | **All Discharges** |
| **Prediction Window = 7** | | | | | | | |
| ENL | Demo | 50.32 (0.79) | 48.66 (0.64) | 61.34 (0.43) | 16.64 (0.27) | 17.29 (0.24) | 22.22 (0.28) |
|  | Demo + Codes | 66.15 (0.60) | 64.25 (0.42) | 71.09 (0.45) | 30.12 (1.00) | 28.33 (0.87) | 32.80 (0.71) |
|  | Demo + Codes + SB | 74.01 (0.48) | 70.98 (0.31) | 77.28 (0.27) | 37.91 (0.92) | 32.57 (0.89) | 40.38 (0.57) |
|  | Demo + Codes + SB + SBDH (NLP) | 74.63 (0.53) | 70.66 (0.44) | 77.65 (0.34) | 39.11 (0.86) | 32.54 (0.86) | 41.50 (0.48) |
|  | Demo + Codes + SB + SBDH (Best) | 74.63 (0.53) | 70.66 (0.44) | 77.65 (0.34) | 39.11 (0.86) | 32.54 (0.86) | 41.50 (0.48) |
| RF | Demo | 50.47 (1.01) | 50.11 (0.84) | 60.11 (0.59) | 16.06 (0.25) | 17.17 (0.70) | 24.61 (0.76) |
|  | Demo + Codes | 65.93 (0.84) | 60.97 (0.64) | 71.46 (0.54) | 26.84 (0.54) | 24.36 (0.35) | 30.16 (0.72) |
|  | Demo + Codes + SB | 72.68 (0.60) | 69.00 (0.64) | 76.51 (0.41) | 32.00 (1.07) | 30.73 (1.51) | 34.89 (0.61) |
|  | Demo + Codes + SB + SBDH (NLP) | 74.80 (0.84) | 71.19 (0.79) | 76.34 (0.68) | 38.26 (1.60) | 35.18 (1.93) | 37.94 (0.82) |
|  | Demo + Codes + SB + SBDH (Best) | 74.80 (0.84) | 71.19 (0.79) | 76.34 (0.68) | 38.26 (1.60) | 35.18 (1.93) | 37.94 (0.82) |
| MLP | Demo | 51.03 (0.93) | 50.20 (1.56) | 60.19 (0.97) | 17.35 (0.44) | 18.57 (0.43) | 24.71 (1.31) |
|  | Demo + Codes | 63.16 (1.25) | 60.48 (1.24) | 69.54 (0.81) | 27.93 (2.56) | 26.19 (2.89) | 33.15 (1.47) |
|  | Demo + Codes + SB | 69.42 (2.05) | 66.43 (1.68) | 74.21 (0.92) | 31.85 (2.44) | 29.07 (1.87) | 41.12 (1.58) |
|  | Demo + Codes + SB + SBDH (NLP) | 70.22 (2.31) | 67.88 (2.03) | 74.99 (1.58) | 34.40 (3.49) | 30.35 (2.82) | 41.39 (2.05) |
|  | Demo + Codes + SB + SBDH (Best) | 70.49 (1.08) | 67.37 (1.07) | 74.44 (0.77) | 34.32 (1.10) | 29.02 (0.85) | 42.26 (1.26) |
| **Prediction Window = 30** | | | | | | | |
| ENL | Demo | 58.20 (0.22) | 56.35 (0.24) | 62.96 (0.13) | 21.49 (0.37) | 19.48 (0.21) | 24.32 (0.25) |
|  | Demo + Codes | 70.48 (0.19) | 64.64 (0.18) | 75.39 (0.15) | 31.70 (0.25) | 26.31 (0.21) | 41.36 (0.33) |
|  | Demo + Codes + SB | 73.75 (0.15) | 65.81 (0.17) | 78.87 (0.15) | 33.20 (0.17) | 25.17 (0.29) | 46.38 (0.21) |
|  | Demo + Codes + SB + SBDH (NLP) | 73.73 (0.17) | 65.82 (0.18) | 78.87 (0.15) | 33.74 (0.18) | 25.08 (0.27) | 46.90 (0.28) |
|  | Demo + Codes + SB + SBDH (Best) | 73.73 (0.17) | 65.82 (0.18) | 78.87 (0.15) | 33.74 (0.18) | 25.08 (0.27) | 46.90 (0.28) |
| RF | Demo | 56.59 (0.29) | 56.54 (0.76) | 60.54 (0.16) | 19.54 (0.32) | 19.38 (0.63) | 22.27 (0.20) |
|  | Demo + Codes | 72.05 (0.55) | 65.26 (0.68) | 75.54 (0.27) | 31.05 (0.75) | 26.54 (0.64) | 36.32 (0.48) |
|  | Demo + Codes + SB | 74.58 (0.30) | 67.45 (0.33) | 78.49 (0.10) | 31.56 (0.38) | 26.81 (0.43) | 40.46 (0.53) |
|  | Demo + Codes + SB + SBDH (NLP) | 74.89 (0.28) | 67.49 (0.37) | 78.59 (0.21) | 33.07 (0.59) | 27.15 (0.45) | 42.82 (0.56) |
|  | Demo + Codes + SB + SBDH (Best) | 74.85 (0.31) | 68.13 (0.23) | 78.17 (0.22) | 33.29 (0.52) | 28.35 (0.41) | 43.11 (0.67) |
| MLP | Demo | 58.37 (0.87) | 58.70 (1.29) | 61.51 (0.66) | 20.47 (0.57) | 20.36 (0.38) | 23.35 (0.67) |
|  | Demo + Codes | 65.15 (0.69) | 61.73 (0.78) | 66.43 (0.39) | 26.92 (0.72) | 23.77 (0.44) | 29.95 (1.03) |
|  | Demo + Codes + SB | 67.74 (2.33) | 62.35 (1.47) | 70.84 (2.67) | 27.56 (1.64) | 23.06 (0.37) | 35.12 (2.41) |
|  | Demo + Codes + SB + SBDH (NLP) | 68.91 (0.77) | 63.24 (0.71) | 71.52 (0.58) | 29.15 (0.75) | 23.75 (0.52) | 37.08 (0.92) |
|  | Demo + Codes + SB + SBDH (Best) | 67.92 (1.12) | 62.27 (0.55) | 71.46 (1.33) | 30.37 (1.52) | 23.67 (0.20) | 39.05 (1.68) |
| **Prediction Window = 90** | | | | | | | |
| ENL | Demo | 60.73 (0.18) | 54.99 (0.28) | 64.17 (0.13) | 22.11 (0.25) | 17.82 (0.14) | 24.98 (0.17) |
|  | Demo + Codes | 75.86 (0.15) | 68.51 (0.18) | 79.73 (0.11) | 39.25 (0.21) | 28.10 (0.27) | 49.35 (0.11) |
|  | Demo + Codes + SB | 76.84 (0.15) | 68.44 (0.22) | 81.05 (0.12) | 38.74 (0.21) | 25.96 (0.20) | 51.87 (0.14) |
|  | Demo + Codes + SB + SBDH (NLP) | 76.68 (0.19) | 68.21 (0.22) | 81.01 (0.14) | 39.23 (0.27) | 25.58 (0.23) | 52.50 (0.15) |
|  | Demo + Codes + SB + SBDH (Best) | 77.09 (0.14) | 69.40 (0.20) | 80.94 (0.14) | 39.86 (0.25) | 26.55 (0.23) | 52.73 (0.16) |
| RF | Demo | 60.45 (0.37) | 56.14 (0.84) | 63.65 (0.23) | 21.47 (0.34) | 17.65 (0.43) | 23.50 (0.31) |
|  | Demo + Codes | 77.49 (0.27) | 69.58 (0.30) | 81.05 (0.22) | 39.01 (0.39) | 26.69 (0.73) | 51.01 (0.53) |
|  | Demo + Codes + SB | 78.62 (0.16) | 69.82 (0.40) | 82.51 (0.16) | 38.61 (0.40) | 25.67 (0.40) | 52.34 (0.50) |
|  | Demo + Codes + SB + SBDH (NLP) | 78.84 (0.32) | 70.56 (0.41) | 82.92 (0.26) | 40.09 (0.38) | 26.74 (0.70) | 55.28 (0.41) |
|  | Demo + Codes + SB + SBDH (Best) | 78.84 (0.32) | 70.56 (0.41) | 82.92 (0.26) | 40.09 (0.38) | 26.74 (0.70) | 55.28 (0.41) |
| MLP | Demo | 59.44 (0.11) | 56.18 (0.13) | 63.30 (0.13) | 20.96 (0.34) | 17.59 (0.09) | 23.18 (0.84) |
|  | Demo + Codes | 71.49 (1.24) | 65.19 (0.92) | 74.39 (1.24) | 34.54 (0.95) | 24.07 (0.99) | 41.25 (1.71) |
|  | Demo + Codes + SB | 70.05 (0.85) | 63.08(1.19) | 74.91 (0.51) | 32.91 (1.33) | 22.78 (1.08) | 42.85 (0.99) |
|  | Demo + Codes + SB + SBDH (NLP) | 73.16 (1.46) | 66.34 (1.47) | 77.61 (1.61) | 35.27 (1.68) | 24.97 (0.87) | 46.38 (1.77) |
|  | Demo + Codes + SB + SBDH (Best) | 73.16 (1.46) | 66.34 (1.47) | 77.61 (1.61) | 35.27 (1.68) | 24.97 (0.87) | 46.38 (1.77) |
| **Prediction Window = 180** | | | | | | | |
| ENL | Demo | 60.52 (0.21) | 53.37 (0.47) | 62.61 (0.12) | 22.83 (0.51) | 16.83 (0.23) | 22.79 (0.17) |
|  | Demo + Codes | 77.99 (0.08) | 69.74 (0.14) | 80.81 (0.05) | 43.05 (0.11) | 27.00 (0.15) | 51.19 (0.08) |
|  | Demo + Codes + SB | 78.95 (0.07) | 70.63 (0.13) | 81.70 (0.05) | 43.41 (0.10) | 26.39 (0.14) | 52.58 (0.07) |
|  | Demo + Codes + SB + NLP SBDH | 78.97 (0.07) | 70.64 (0.12) | 81.73 (0.04) | 43.68 (0.07) | 26.43 (0.11) | 52.95 (0.08) |
|  | Demo + Codes + SB + SBDH | 79.52 (0.08) | 70.94 (0.15) | 82.04 (0.05) | 44.36 (0.11) | 26.37 (0.14) | 53.47 (0.09) |
| RF | Demo | 62.81 (0.40) | 55.00 (0.76) | 63.98 (0.23) | 23.84 (0.29) | 17.21 (0.47) | 22.98 (0.13) |
|  | Demo + Codes | 80.89 (0.30) | 71.14 (0.45) | 83.26 (0.25) | 45.07 (0.77) | 27.52 (0.74) | 56.37 (0.49) |
|  | Demo + Codes + SB | 81.24 (0.27) | 71.56 (0.29) | 83.57 (0.21) | 44.95 (0.66) | 27.18 (0.57) | 57.38 (0.31) |
|  | Demo + Codes + SB + SBDH (NLP) | 81.60 (0.19) | 71.63 (0.29) | 84.25 (0.20) | 46.65 (0.50) | 27.28 (0.52) | 59.87 (0.35) |
|  | Demo + Codes + SB + SBDH (Best) | 81.70 (0.32) | 71.81 (0.48) | 83.94 (0.17) | 47.07 (0.64) | 27.55 (0.33) | 59.95 (0.37) |
| MLP | Demo | 61.40 (0.14) | 51.84 (0.71) | 63.52 (0.08) | 23.33 (0.77) | 15.86 (0.55) | 23.17 (0.22) |
|  | Demo + Codes | 72.37 (1.97) | 64.01 (1.76) | 74.68 (1.86) | 34.72 (0.69) | 22.85 (0.52) | 41.89 (1.68) |
|  | Demo + Codes + SB | 74.48 (0.11) | 65.12 (0.58) | 76.20 (0.30) | 36.77 (0.17) | 23.43 (0.56) | 44.85 (0.33) |
|  | Demo + Codes + SB + SBDH (NLP) | 74.72 (1.25) | 65.59 (1.15) | 77.39 (0.84) | 37.25 (2.44) | 23.44 (1.72) | 46.53 (2.25) |
|  | Demo + Codes + SB + SBDH (Best) | 74.72 (1.25) | 65.59 (1.15) | 77.39 (0.84) | 37.25 (2.44) | 23.44 (1.72) | 46.53 (2.25) |

**Supplementary Table 5.** Performance of different predictive models across different prediction windows. eTable8 outlines different SBDH settings. ROC AUC: area under the receiver operator characteristic curve; PR AUC: area under the precision recall curve; SD: standard deviation; Demo: demographic variables; SB: suicidal behaviors – attempt and ideation; Codes: diagnosis, procedure, and medication codes; SBDH: social and behavioral determinants of health; ENL: Elastic Net Logistic Regression; RF: Random Forest; MLP: Multilayer Perceptron.

| **Model** | **Predictor Configuration** | **ROC AUC (SD), %** | **PR AUC (SD), %** |
| --- | --- | --- | --- |
| **Prediction Window = 7** | | | |
| ENL | Demo | 61.35 (0.42) | 22.34 (0.28) |
|  | Demo + Codes | 71.19 (0.44) | 33.25 (0.74) |
|  | Demo + Codes + SB | 77.36 (0.27) | 41.06 (0.60) |
|  | Demo + Codes + SB + SBDH (NLP) | 77.73 (0.33) | 42.08 (0.49) |
|  | Demo + Codes + SB + SBDH (Best) | 77.73 (0.33) | 42.08 (0.49) |
| RF | Demo | 60.13 (0.58) | 24.76 (0.75) |
|  | Demo + Codes | 71.55 (0.54) | 30.34 (0.72) |
|  | Demo + Codes + SB | 76.61 (0.42) | 35.10 (0.61) |
|  | Demo + Codes + SB + SBDH (NLP) | 76.41 (0.67) | 38.11 (0.80) |
|  | Demo + Codes + SB + SBDH (Best) | 76.41 (0.67) | 38.11 (0.80) |
| MLP | Demo | 60.22 (0.97) | 24.84 (1.29) |
|  | Demo + Codes | 69.58 (0.78) | 33.27 (1.66) |
|  | Demo + Codes + SB | 74.29 (0.92) | 41.32 (1.67) |
|  | Demo + Codes + SB + SBDH (NLP) | 75.04 (1.56) | 41.54 (2.00) |
|  | Demo + Codes + SB + SBDH (Best) | 74.44 (0.76) | 42.32 (1.23) |
| **Prediction Window = 30** | | | |
| ENL | Demo | 63.43 (0.13) | 24.57 (0.24) |
|  | Demo + Codes | 76.13 (0.15) | 42.37 (0.37) |
|  | Demo + Codes + SB | 79.33 (0.15) | 47.35 (0.22) |
|  | Demo + Codes + SB + SBDH (NLP) | 79.26 (0.15) | 47.80 (0.30) |
|  | Demo + Codes + SB + SBDH (Best) | 79.26 (0.15) | 47.80 (0.30) |
| RF | Demo | 60.28 (0.16) | 21.86 (0.19) |
|  | Demo + Codes | 75.76 (0.23) | 36.25 (0.38) |
|  | Demo + Codes + SB | 78.73 (0.08) | 40.08 (0.42) |
|  | Demo + Codes + SB + SBDH (NLP) | 78.81 (0.20) | 42.62 (0.53) |
|  | Demo + Codes + SB + SBDH (Best) | 78.25 (0.20) | 42.42 (0.59) |
| MLP | Demo | 61.65 (0.57) | 23.36 (0.82) |
|  | Demo + Codes | 66.59 (0.37) | 30.23 (1.18) |
|  | Demo + Codes + SB | 70.84 (2.77) | 35.22 (2.48) |
|  | Demo + Codes + SB + SBDH (NLP) | 71.41 (0.57) | 37.21 (0.97) |
|  | Demo + Codes + SB + SBDH (Best) | 71.51 (1.37) | 39.25 (1.85) |
| **Prediction Window = 90** | | | |
| ENL | Demo | 65.44 (0.12) | 23.86 (0.23) |
|  | Demo + Codes | 80.51 (0.12) | 48.30 (0.13) |
|  | Demo + Codes + SB | 81.56 (0.13) | 50.53 (0.16) |
|  | Demo + Codes + SB + SBDH (NLP) | 81.41 (0.15) | 50.81 (0.15) |
|  | Demo + Codes + SB + SBDH (Best) | 81.37 (0.16) | 51.43 (0.20) |
| RF | Demo | 63.54 (0.23) | 21.62 (0.34) |
|  | Demo + Codes | 79.67 (0.23) | 42.78 (0.63) |
|  | Demo + Codes + SB | 81.50 (0.20) | 45.92 (0.57) |
|  | Demo + Codes + SB + SBDH (NLP) | 81.72 (0.27) | 47.41 (0.45) |
|  | Demo + Codes + SB + SBDH (Best) | 81.72 (0.27) | 47.41 (0.45) |
| MLP | Demo | 63.52 (0.58) | 21.36 (0.22) |
|  | Demo + Codes | 73.53 (1.40) | 38.20 (1.98) |
|  | Demo + Codes + SB | 73.86 (0.76) | 39.26 (1.20) |
|  | Demo + Codes + SB + SBDH (NLP) | 75.57 (1.80) | 41.16 (2.26) |
|  | Demo + Codes + SB + SBDH (Best) | 75.57 (1.80) | 41.16 (2.26) |
| **Prediction Window = 180** | | | |
| ENL | Demo | 62.76 (0.13) | 17.71 (0.16) |
|  | Demo + Codes | 80.27 (0.04) | 43.98 (0.08) |
|  | Demo + Codes + SB | 81.05 (0.03) | 45.12 (0.10) |
|  | Demo + Codes + SB + SBDH (NLP) | 81.02 (0.02) | 45.27 (0.10) |
|  | Demo + Codes + SB + SBDH (Best) | 81.39 (0.03) | 45.75 (0.11) |
| RF | Demo | 62.99 (0.14) | 17.43 (0.11) |
|  | Demo + Codes | 79.83 (0.25) | 39.54 (0.47) |
|  | Demo + Codes + SB | 80.23 (0.28) | 40.15 (0.49) |
|  | Demo + Codes + SB + SBDH (NLP) | 80.76 (0.19) | 42.14 (0.50) |
|  | Demo + Codes + SB + SBDH (Best) | 80.47 (0.21) | 42.94 (0.48) |
| MLP | Demo | 63.30 (0.32) | 18.08 (0.24) |
|  | Demo + Codes | 71.56 (2.15) | 28.56 (2.41) |
|  | Demo + Codes + SB | 72.91 (0.90) | 31.63 (0.27) |
|  | Demo + Codes + SB + SBDH (NLP) | 74.66 (1.09) | 34.44 (2.13) |
|  | Demo + Codes + SB + SBDH (Best) | 74.66 (1.09) | 34.44 (2.13) |

**Supplementary Table 6.** Performance of different predictive models across different prediction windows on discharges with patients unique to the test set. ROC AUC: area under the receiver operator characteristic curve; PR AUC: area under the precision recall curve; SD: standard deviation; Demo: demographic variables; SB: suicidal behaviors – attempt and ideation; Codes: diagnosis, procedure, and medication codes; SBDH: social and behavioral determinants of health; ENL: Elastic Net Logistic Regression; RF: Random Forest; MLP: Multilayer Perceptron.

| **Model** | **Risk Group Size, P** | **Sensitivity (SD), (%)** | **Specificity (SD), %** | **PPV (SD), %** | **Adjusted PPV (SD), %** |
| --- | --- | --- | --- | --- | --- |
| **Prediction Window = 7** | | | | | |
| ENL | 0.05 | 16.69 (0.71) | 97.52 (0.15) | 58.60 (2.50) | 0.03 (0.00) |
|  | 0.10 | 29.27 (0.83) | 94.17 (0.17) | 51.40 (1.45) | 0.02 (0.00) |
|  | 0.20 | 48.74 (1.85) | 86.14 (0.39) | 42.54 (1.62) | 0.02 (0.00) |
|  | 0.60 | 88.68 (0.46) | 46.15 (0.10) | 25.75 (0.13) | 0.01 (0.00) |
| RF | 0.05 | 12.98 (1.03) | 96.74 (0.22) | 45.58 (3.63) | 0.02 (0.00) |
|  | 0.10 | 24.90 (0.74) | 93.25 (0.16) | 43.72 (1.29) | 0.02 (0.00) |
|  | 0.20 | 47.28 (1.52) | 85.83 (0.32) | 41.27 (1.32) | 0.02 (0.00) |
|  | 0.60 | 88.41 (1.27) | 46.09 (0.27) | 25.67 (0.37) | 0.01 (0.00) |
| MLP | 0.05 | 16.11 (0.62) | 97.40 (0.13) | 56.59 (2.19) | 0.03 (0.00) |
|  | 0.10 | 26.93 (1.74) | 93.68 (0.37) | 47.29 (3.05) | 0.02 (0.00) |
|  | 0.20 | 48.34 (3.38) | 86.05 (0.71) | 42.20 (2.95) | 0.02 (0.00) |
|  | 0.60 | 82.56 (1.56) | 44.86 (0.33) | 23.97 (0.45) | 0.01 (0.00) |
| **Prediction Window = 30** | | | | | |
| ENL | 0.05 | 18.94 (0.48) | 97.89 (0.10) | 65.04 (1.64) | 0.13 (0.01) |
|  | 0.10 | 33.30 (0.59) | 94.83 (0.12) | 57.17 (1.01) | 0.09 (0.00) |
|  | 0.20 | 54.56 (0.67) | 87.18 (0.14) | 46.84 (0.58) | 0.06 (0.00) |
|  | 0.60 | 89.38 (0.32) | 46.09 (0.07) | 25.56 (0.09) | 0.02 (0.00) |
| RF | 0.05 | 15.10 (0.75) | 97.10 (0.16) | 51.86 (2.57) | 0.07 (0.01) |
|  | 0.10 | 27.96 (0.83) | 93.73 (0.17) | 48.01 (1.42) | 0.06 (0.00) |
|  | 0.20 | 51.70 (1.13) | 86.58 (0.23) | 44.38 (0.97) | 0.05 (0.00) |
|  | 0.60 | 88.14 (0.86) | 45.84 (0.18) | 25.20 (0.25) | 0.02 (0.00) |
| MLP | 0.05 | 16.15 (0.64) | 97.31 (0.13) | 55.46 (2.21) | 0.08 (0.01) |
|  | 0.10 | 28.87 (0.76) | 93.92 (0.16) | 49.56 (1.30) | 0.07 (0.00) |
|  | 0.20 | 43.13 (3.22) | 84.81 (0.67) | 37.02 (2.77) | 0.04 (0.00) |
|  | 0.60 | 81.36 (1.43) | 44.43 (0.30) | 23.26 (0.41) | 0.02 (0.00) |
| **Prediction Window = 90** | | | | | |
| ENL | 0.05 | 23.38 (0.28) | 98.24 (0.05) | 69.89 (0.83) | 0.39 (0.02) |
|  | 0.10 | 39.47 (0.49) | 95.17 (0.09) | 58.84 (0.73) | 0.24 (0.01) |
|  | 0.20 | 57.73 (0.47) | 86.62 (0.08) | 43.03 (0.35) | 0.13 (0.00) |
|  | 0.60 | 91.13 (0.59) | 45.46 (0.10) | 22.63 (0.15) | 0.05 (0.00) |
| RF | 0.05 | 22.48 (0.40) | 98.08 (0.07) | 67.20 (1.21) | 0.35 (0.02) |
|  | 0.10 | 37.12 (0.48) | 94.75 (0.08) | 55.33 (0.71) | 0.21 (0.01) |
|  | 0.20 | 56.74 (0.53) | 86.45 (0.09) | 42.30 (0.40) | 0.12 (0.00) |
|  | 0.60 | 92.07 (0.60) | 45.62 (0.11) | 22.87 (0.15) | 0.05 (0.00) |
| MLP | 0.05 | 19.88 (1.03) | 97.62 (0.18) | 59.44 (3.06) | 0.25 (0.03) |
|  | 0.10 | 32.09 (1.09) | 93.87 (0.19) | 47.85 (1.63) | 0.16 (0.01) |
|  | 0.20 | 49.62 (2.24) | 85.20 (0.39) | 36.98 (1.67) | 0.10 (0.01) |
|  | 0.60 | 86.55 (2.01) | 44.65 (0.35) | 21.49 (0.50) | 0.05 (0.00) |
| **Prediction Window = 180** | | | | | |
| ENL | 0.05 | 24.30 (0.36) | 97.86 (0.05) | 62.56 (0.92) | 0.56 (0.02) |
|  | 0.10 | 38.54 (0.38) | 94.22 (0.06) | 49.49 (0.48) | 0.33 (0.01) |
|  | 0.20 | 59.32 (0.33) | 85.80 (0.05) | 38.05 (0.21) | 0.21 (0.00) |
|  | 0.60 | 92.48 (0.51) | 44.80 (0.07) | 19.76 (0.11) | 0.08 (0.00) |
| RF | 0.05 | 23.04 (0.28) | 97.68 (0.04) | 59.32 (0.71) | 0.49 (0.01) |
|  | 0.10 | 38.18 (0.75) | 94.17 (0.11) | 49.04 (0.97) | 0.32 (0.01) |
|  | 0.20 | 57.39 (0.68) | 85.51 (0.10) | 36.81 (0.44) | 0.19 (0.00) |
|  | 0.60 | 91.31 (0.77) | 44.63 (0.11) | 19.51 (0.16) | 0.08 (0.00) |
| MLP | 0.05 | 19.07 (1.65) | 97.09 (0.24) | 49.11 (4.25) | 0.33 (0.06) |
|  | 0.10 | 30.64 (2.09) | 93.06 (0.31) | 39.36 (2.69) | 0.22 (0.03) |
|  | 0.20 | 47.72 (1.94) | 84.09 (0.29) | 30.61 (1.24) | 0.15 (0.01) |
|  | 0.60 | 86.49 (0.55) | 43.92 (0.08) | 18.48 (0.12) | 0.08 (0.00) |

**Supplementary Table 7.** Performance of different predictive models with the best predictor configuration on discharges with patients unique to the test set. PPV: positive predictive value; ENL: Elastic Net Logistic Regression; RF: Random Forest; MLP: Multilayer Perceptron.

# **Supplementary Note 5: Impact of NLP-extracted Predictors**

| **Prediction Window** | **Model** | **SBDH Type** | **Best performing SBDH setting** | **SBDH Extraction Timeframe** |
| --- | --- | --- | --- | --- |
| 7 | ENL | NLP | NLP-extracted SBDH | 7 Days |
|  |  | Best | NLP-extracted SBDH | 7 Days |
|  | RF | NLP | NLP-extracted SBDH + ADI | 7 Days |
|  |  | Best | NLP-extracted SBDH + ADI | 7 Days |
|  | MLP | NLP | NLP-extracted SBDH | 7 Days |
|  |  | Best | Combined SBDH | 365 Days |
| 30 | ENL | NLP | NLP-extracted SBDH + ADI | 7 Days |
|  |  | Best | NLP-extracted SBDH + ADI | 7 Days |
|  | RF | NLP | NLP-extracted SBDH + ADI | 7 Days |
|  |  | Best | Structured SBDH + ADI | All |
|  | MLP | NLP | NLP-extracted SBDH + ADI | 730 Days |
|  |  | Best | Combined SBDH + ADI | All |
| 90 | ENL | NLP | NLP-extracted SBDH + ADI | All |
|  |  | Best | Structured SBDH + ADI | All |
|  | RF | NLP | NLP-extracted SBDH + ADI | 365 Days |
|  |  | Best | NLP-extracted SBDH + ADI | 365 Days |
|  | MLP | NLP | NLP-extracted SBDH + ADI | 7 Days |
|  |  | Best | NLP-extracted SBDH + ADI | 7 Days |
| 180 | ENL | NLP | NLP-extracted SBDH + ADI | 7 Days |
|  |  | Best | Combined SBDH + ADI | All |
|  | RF | NLP | NLP-extracted SBDH + ADI | 730 Days |
|  |  | Best | Structured SBDH + ADI | 730 Days |
|  | MLP | NLP | NLP-extracted SBDH + ADI | All |
|  |  | Best | NLP-extracted SBDH + ADI | All |

**Supplementary Table 8.** Best performing SBDH settings for different models across different prediction windows. We show the best settings for both NLP-extracted SBDH configurations and all types of SBDH (structured, NLP-extracted and combined) configurations. SBDH Extraction timeframe indicates the period before discharge during which SBDH predictors were extracted (excluding ADI). ‘All’ indicates SBDH predictors were extracted from all six time windows (7, 30, 90, 180, 365, and 730 days).

# **Supplementary Note 6: Calibration and Predictor Importance**

R version 4.4.1 was used to calculate ICI statistics following the implementation in the ‘gmish’ library.


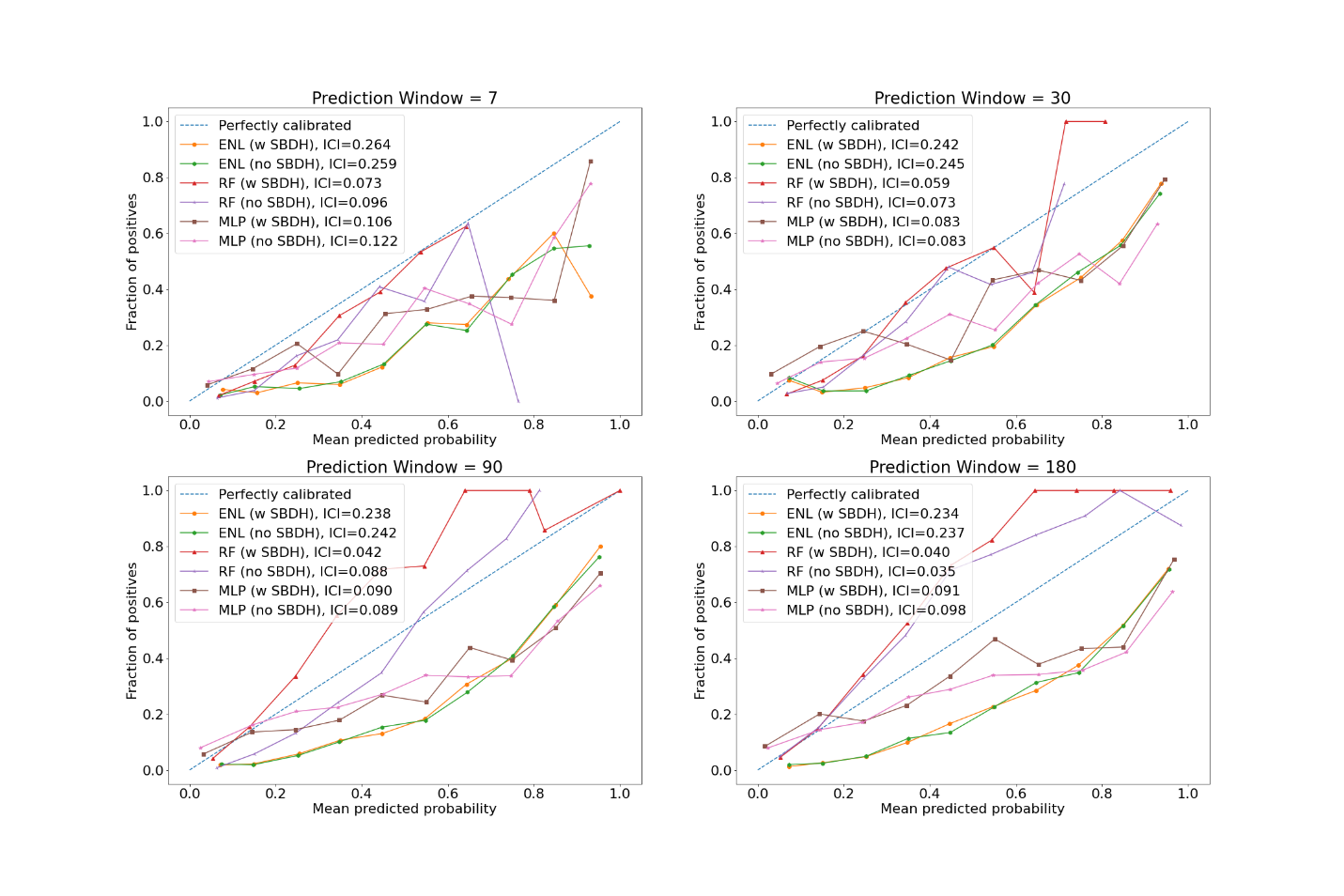


**Supplementary Figure 1.** Calibration analysis of all models on the test set. ENL: elastic net logistic regression; RF: random forest; MLP: multilayer perceptron.


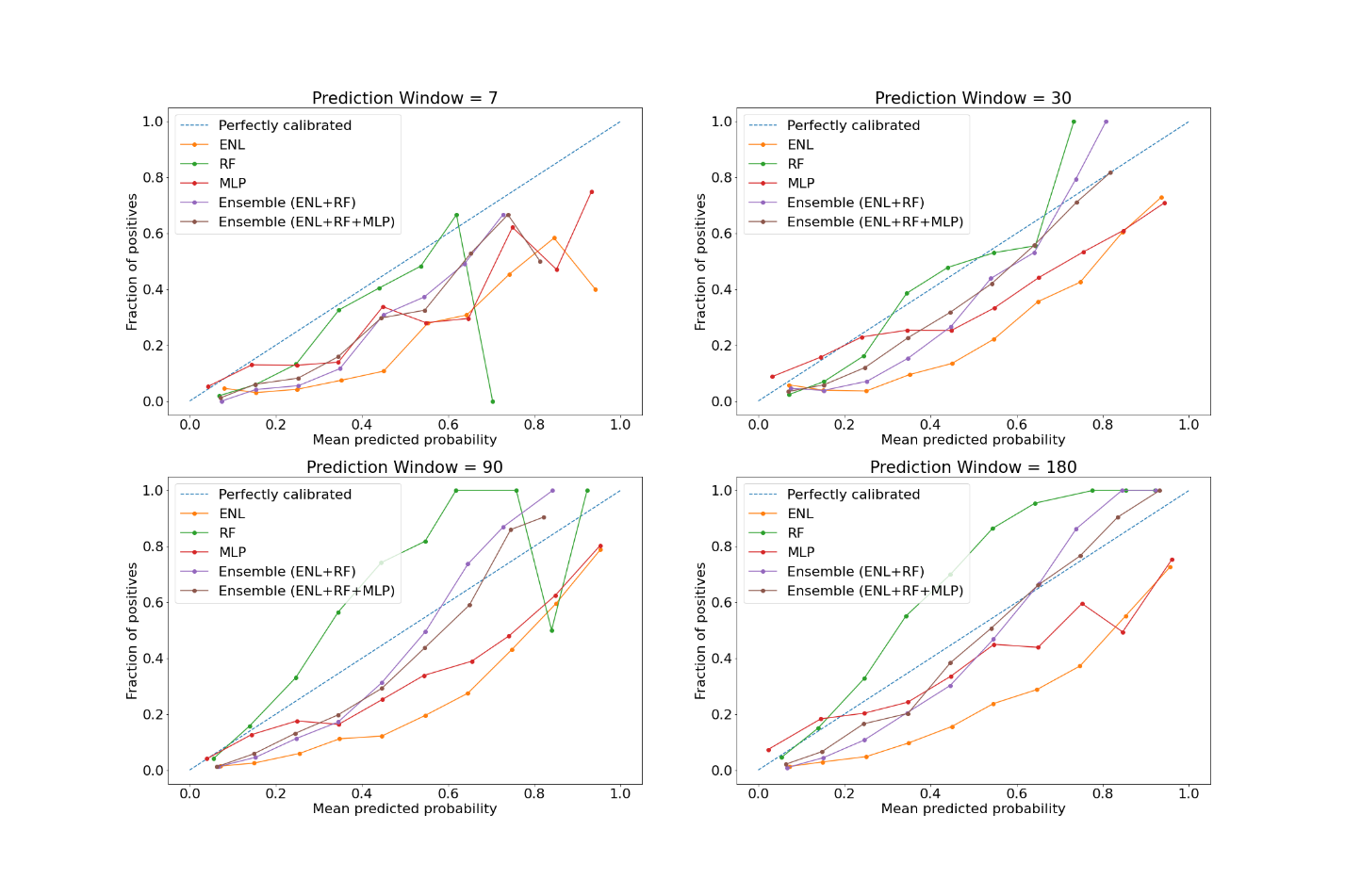


**Supplementary Figure 2.** Calibration analysis of all models on the test set, including the ensembled versions. ENL: elastic net logistic regression; RF: random forest; MLP: multilayer perceptron.


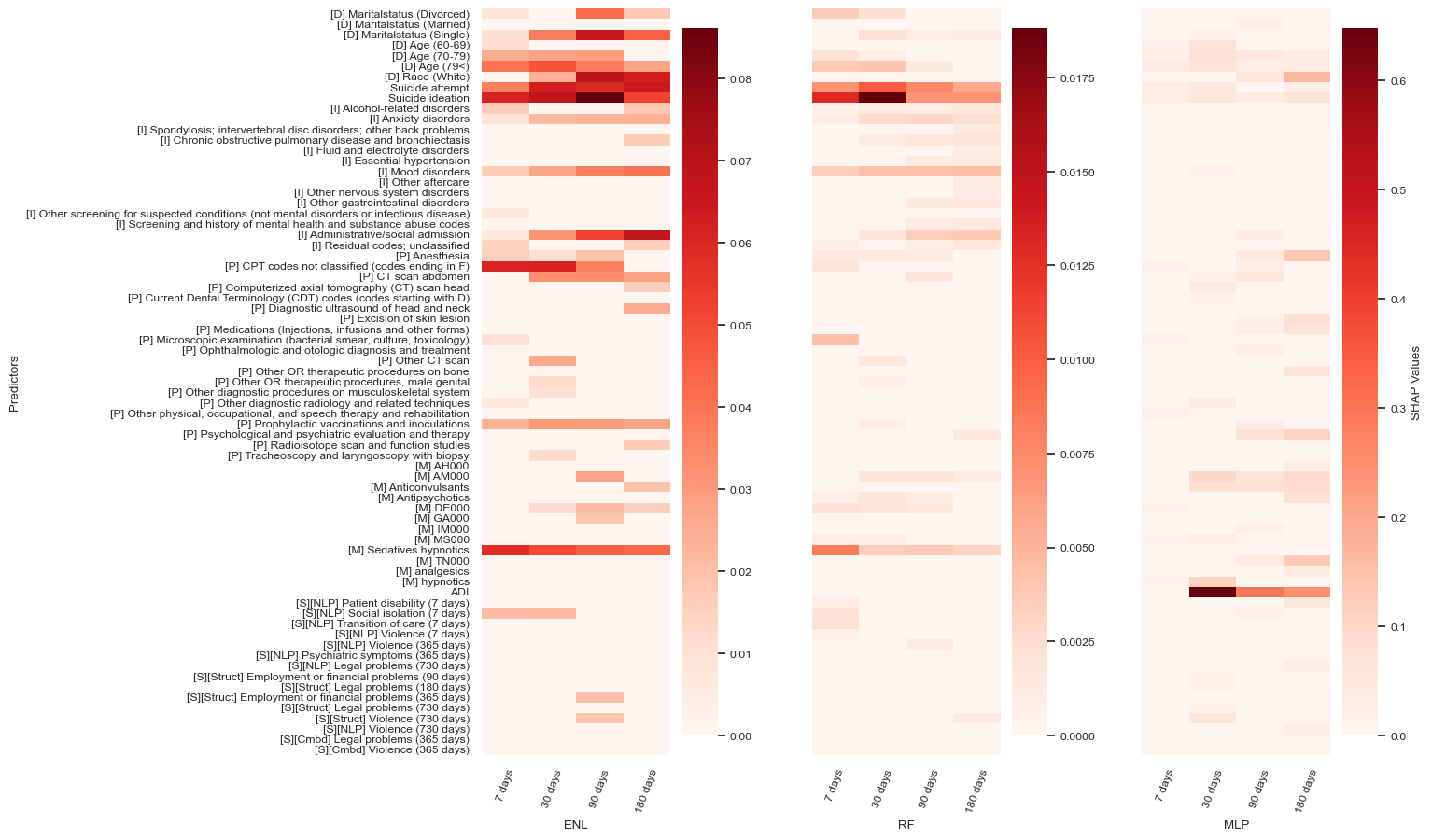


(a)


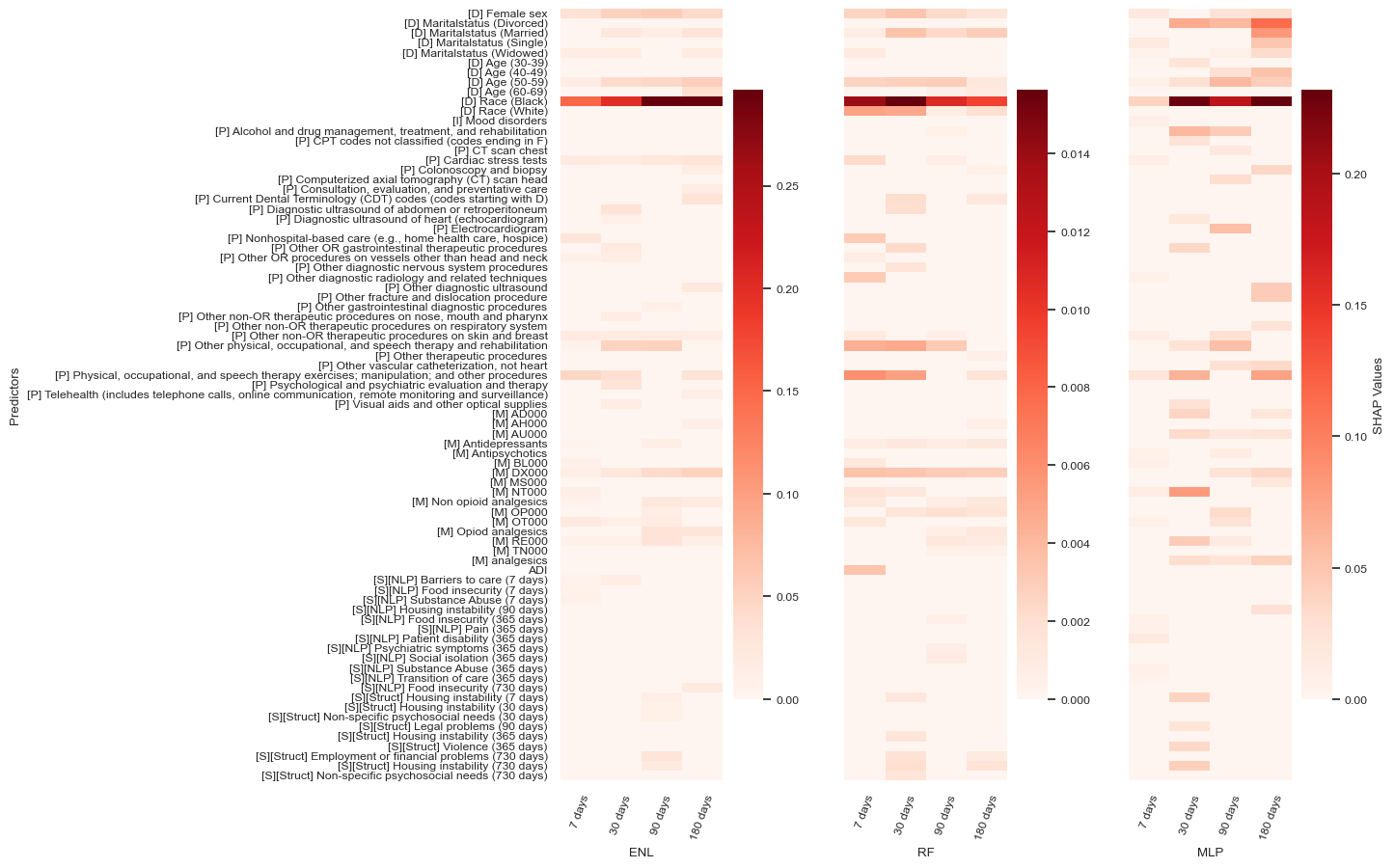


(b)

**Supplementary Figure 3.** Predictor importance using SHAP (Shapley Additive Explanations) values in the test set. Top 20 predictors were considered for each model-prediction-window combination. Predictors not in the top 20 for a specific model-prediction-window have been assigned SHAP values of zeros. (a) Top 20 positive predictors, (b) Top 20 negative predictors. [D]: Demographics; [I]: Diagnosis codes group; [P]: Procedure codes group; [M]: Medication codes group; [S]: SBDH; [NLP]: NLP-extracted SBDH; [Struct]: Structured SBDH; [Cmbd]: Combined SBDH

# References

1. Kessler RC, Bauer MS, Bishop TM, et al. Using Administrative Data to Predict Suicide After Psychiatric Hospitalization in the Veterans Health Administration System. *Front Psychiatry*. 2020;11:390. doi:10.3389/fpsyt.2020.00390

2. US Department of Veteran Services. VA National Formulary - Pharmacy Benefits Management Services. Accessed February 12, 2023. https://www.pbm.va.gov/nationalformulary.asp

3. Mitra A, Pradhan R, Melamed RD, et al. Associations Between Natural Language Processing–Enriched Social Determinants of Health and Suicide Death Among US Veterans. *JAMA Netw Open*. 2023;6(3):e233079-e233079. doi:10.1001/JAMANETWORKOPEN.2023.3079

4. Benjamini Y, Hochberg Y. Controlling the False Discovery Rate: A Practical and Powerful Approach to Multiple Testing. *Journal of the Royal Statistical Society: Series B (Methodological)*. 1995;57(1):289-300. doi:10.1111/J.2517-6161.1995.TB02031.X

1. <https://www.hcup-us.ahrq.gov/toolssoftware/ccs/ccs.jsp> [↑](#footnote-ref-1)
